# The evolution of identity signals for co-ordination in diverse societies

**DOI:** 10.1017/ehs.2026.10037

**Published:** 2026-03-30

**Authors:** Nathan Gabriel, Adrian Bell, Paul Smaldino

**Affiliations:** 1 Department of Cognitive & Information Sciences, University of California, Merced, California, USA; 2 Department of Anthropology, University of Utah, Utah, USA; 3 Santa Fe Institute, New Mexico, USA

**Keywords:** Bach or Stravinsky, co-ordination, cultural evolution, intersectionality, identity signals

## Abstract

Individual social identities indicate group affiliations and are typically associated with group-typical preferences, signals that indicate group membership, and the propensity to condition actions on the social signals of others, resulting in group-differentiated interaction norms. Past work modelling identity signalling and co-ordination has typically assumed that individuals belong to one of a discrete set of groups. Yet individuals can simultaneously belong to multiple groups, which may be nested within larger groupings. Here, we introduce the *generalized Bach or Stravinsky game*, a co-ordination game with ordered preferences, which allows us to construct a model that captures the overlapping and hierarchical nature of social identity. Our model unifies several prior results into a single framework, including results related to co-ordination, minority disadvantage, and cross-cultural competence. Our model also allows agents to express complex social identities through multidimensional signalling, which we use to explore a variety of complex group structures. Our consideration of intersectional identities exposes flaws in naive measures of group structure, illustrating how empirical studies may overlook some social identities if they do not consider the behaviours that those identities function to afford.

## Graphical abstract

**Figure:**
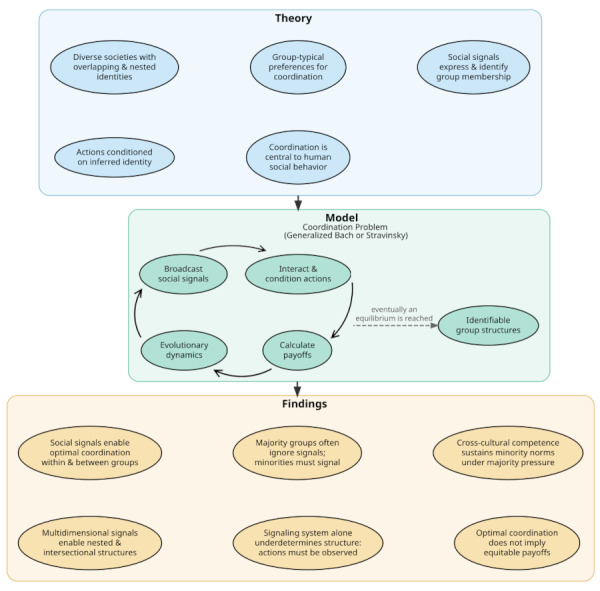

## Social media summary

We develop a formal model of the evolution of multidimensional social identity signals, allowing us to explore the emergence of complex group structures, cross-cultural competence, and intersectional identity in diverse societies.

## 1. Introduction

As early as the Third Dynasty of Ur (2100 BCE) in ancient Mesopotamia, we have evidence of complex social identities; cities as far apart as Ur and Mari, well over 100 miles apart, evidence a body of literature indicative of a shared Sumerian identity, while literature concerned with each city’s patron gods indicate more localized social identities (Delnero, 2016). The layered and overlapping identities of this case are indicative of similar stories in human history, as ethnographic and archaeological evidence suggests that coalitions, warfare, fusion–fission dynamics, and migration are features of early human groups (Singh & Glowacki, 2022). As a result, humans likely used a large variety of markers of group identity to facilitate co-ordination within and between groups (Cohen, 2012; Hodder, 1982; Johnson et al., 2024; Smaldino, 2019), especially in groups too large for a group member to know every individual in the group (Moffett, 2013). In today’s large, diverse, and globally connected societies, complex group identities are commonplace (Roccas & Brewer, 2002).

Some social groupings follow straightforward principles of organization. For example, all astrophysicists are physicists and all physicists are scientists. As such, you can assume that all astrophysicists share things in common with all physicists and all physicists share things in common with all scientists. But there are also ways in which social identities break from the contours of simple set membership relations. For example, the mere conjunction of dominant narratives in Black liberation and in mainstream feminism does not yield important narratives of Black feminists (Combahee River Collective, 1977; Crenshaw, 1991).

To get at this richer understanding of identity and power dynamics, we will consider how people come to optimize interactions in populations with diverse and multifaceted sets of preferences. Some prior literature, considering the power dynamics of how people are related to each other, has referred to such sets of identity relationships as group topologies (Bourdieu, 1985; Burt, 1977; Ioannides, 2006). We use the more generic term ‘group structures’ to refer to the relationships between preferences, signals, and interpersonal behaviours that emerge in populations of interacting individuals. This paper provides a formal model of how a variety of social identity structures can obtain in a population of agents trying to co-ordinate behaviour and preferentially interact with ingroup members.

### 1.1. Preferences, signals, and actions

Conceptually, there are three aspects of social group identities that we model: (i) the *preferences* of individuals, which correlate with social group membership; (ii) the emergence of *social signals* used to express and identify group membership; and (iii) the use of those social signals to *co-ordinate actions*. In high school cliques, Goths and Emos may have different values and music preferences; they use differences in hair and clothing style to identify group members, which help them decide who to join at recess. Swingers might use upside-down pineapples to signal their preferences for ‘co-ordinating behaviour’ with other couples (Pelzer, 2023). Similar empirical examples abound (Barth, 1969; Berger & Heath, 2008; Lin et al., 2024). But, these examples still lack the complexity that we often exhibit in our social identities, in which preferences for how we behave and interact are weighed against the availability of suitable partners, leading those aspects of expressed identity to be highly context-dependent (Bunce, 2020; McCluney et al., 2021; Roccas & Brewer, 2002; Smaldino, 2019). Our model is intended to create continuity between prior work on identity signalling and co-ordination that has dealt with simple discrete identities (Boyd & Richerson, 1987; Castro & Toro, 2007; Goodman et al., 2023; Macanovic et al., 2024; McElreath et al., 2003; Nettle & Dunbar, 1997; Smaldino, 2022; Smaldino & Turner, 2022; Smaldino et al., 2018) and the need to capture these more complex, context-dependent identity-mediated interactions.

Our model assigns agents different preferences reflective of a complex society and illustrates how these preferences can lead to social signalling of complex social identities as well as norms for co-ordination that are conditioned on the signals that agents perceive. For convenience, we hold agent preferences fixed, and allow strategies for social signals and co-ordinative actions to evolve. In reality, preferences may also evolve; this aspect of our model coheres with much of the prior literature and is a convenient simplifying assumption. In Appendix B, we discuss an alternative learning dynamic for the model that can facilitate future research adding dynamically evolving preferences to the model. We use the term ‘group structure’ to refer to the union of agents’ preferences, signalling strategies, and behavioural strategies for interacting with others contingent upon the receipt of particular signals. Group structures are an emergent feature of our model. When referring to a subset of the population that shares the same preferences, we use the terms ‘preference type’ or ‘preference group’ interchangeably.

### 1.2. The Mesopotamian approach

Our approach diverges from most prior models in two important ways. First, we allow agents to broadcast social signals in multiple dimensions rather than a single dimension. Second, we allow a broad range of pay-offs that can be associated with successful co-ordination, based on the preferences of the individuals involved. Consider a hypothetical example in which Catholics prefer to greet others with handshakes while Protestants prefer hugs. If a Catholic and a Protestant end up hugging upon meeting, the Protestant receives a higher pay-off than the Catholic, but both receive higher payoffs than if they had entirely failed to co-ordinate on a greeting. We will continue to use the example of greetings as a way of describing co-ordinating behaviour, though our model can represent any co-ordinative activity.

**Figure 1.**
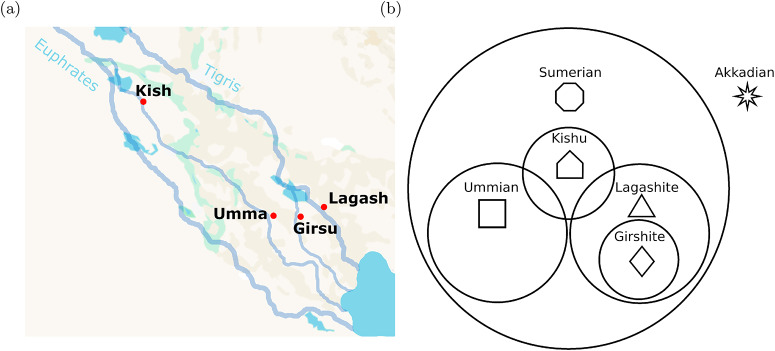
(a) Ancient Sumerian cities used throughout the paper as a motivating example. (b) Diagram of relationships between the different Mesopotamian identities along with shapes used to represent each of them throughout the paper.

To avoid having to construct artificial identity structures to motivate our analysis, we will anchor our discussion of the model with an example of social groups from ancient Mesopotamia (Fig. 1). This is a narrative device; the specific preferences and actions we assign to each group of agents are not necessarily reflective of historical reality. In ancient Mesopotamia there were two rival Sumerian cities, Umma and Lagash, who disagreed continually over who would control the fertile land between them. Let us assume that members of each group preferred the greeting of their own city over the greeting of the other. Despite their rivalry, both were Sumerian-speaking cities, and so we assume that members of both groups preferred a Sumerian greeting over an Akkadian greeting. The Akkadians were a Semitic language-speaking people living in and around northern Sumeria, who eventually conquered the region in 2334 BCE. Prior to that, Lagash established the city of Girsu in the middle of the disputed territory between themselves and Umma, where the temple of Lagash’s patron deity Ningirsu was housed. We can therefore think of Girshites as being a rather zealous subgroup of the broader Lagash community. Finally, let us consider the Sumerian city of Kish. Around 2550 BCE, the king of Kish negotiated a treaty between Umma and Lagash. Thus, we can treat Kishus as having some overlapping identity with Ummians, and perhaps with the Lagashites as well.

**Figure 2.**
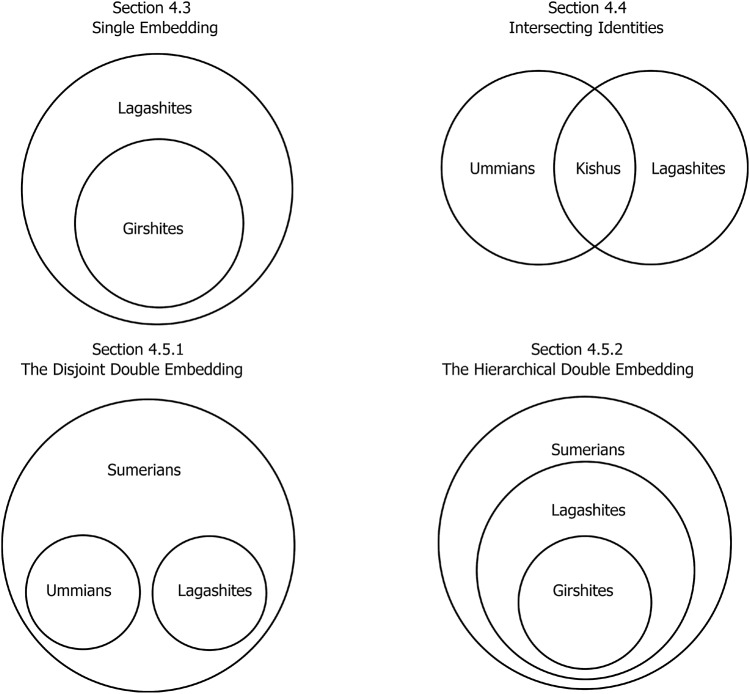
A rough sketch of some group structures that can be produced with our model.

In our analyses, a large population of agents plays a correlative co-ordination game that allows agents to co-ordinate despite having different preferences. Section 2 introduces the baseline model, including the generalized Bach or Stravinsky (BoS) game, the learning dynamics that allow strategies to evolve, and the results of simulations considering how co-ordinating behaviours evolve in the absence of signals. Section 3 explains how (potentially costly) social identity signals are incorporated into the model, and presents simulation results for simulations in which agents can learn to use unidimensional signals to condition their behaviour upon. These simulations establish a few properties of the model that are carried forward as more complex (multidimensional) signalling is added. First, we find that larger communities who share the same preferences are privileged over smaller preference groups; specifically, the population as a whole is more likely to settle on the preferences of a larger preference group than a smaller preference group. Second, we find that the presence of a larger preference group incentivizes other smaller preference groups to adopt social identity signals that they might not otherwise use. Finally, in Section 4, we consider the emergence of complex group structures involving co-ordination among nested or overlapping preference types (Fig. 2), made possible by allowing agents to signal in multiple dimensions simultaneously.

## 2. Base model description and signal-free results

Our model is complex, but it has simple roots. In this section, we introduce the base model, which includes agent interaction and learning but does not yet include identity signalling or signal-contingent behaviour. We then present simulation results indicating the model outcomes in the absence of signalling. Python code for all model versions presented in this paper is available at: https://github.com/nathanlgabriel/social_identity_signaling.

### 2.1. Generalizing the BoS game

The traditional BoS game is a one-shot, two-option co-ordination game between two players. It was introduced to the literature by Luce and Raiffa (1957) as the ‘battle of the sexes’ game, but has since been rebranded the ‘Bach or Stravinsky’ game to dissociate it from undesirable prejudices and stereotypes while maintaining the same abbreviation, BoS (Dekker & Van Rooy, 2000). In this game, each player prefers to co-ordinate on a different behaviour – one would rather go to the Bach concert, the other would rather hear Stravinsky – but both players prefer *some* co-ordination to non-co-ordination. The pay-off matrix (Table 1) is as follows:

**Table 1.** Traditional Bach or Stravinsky game; 
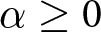

		**Player 2**	
		Bach	Stravinsky
**Player 1**	Bach	1+ 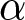 , 1	0, 0
	Stravinsky	0, 0	1, 1+ 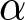

*Note:* Each cell lists the pay-offs to players 1 and 2, respectively.

The game captures a broad class of scenarios in which individuals have different preferences for co-ordination. We co-ordinate when deciding to shake hands or high-five, or when choosing music for a party. Such co-ordination problems are commonplace in communication; e.g. the same thing might be called ‘battle of the sexes’ or ‘Bach or Stravinsky’, it might be called ‘the morning star’, ‘the evening star’, or ‘Venus’; it might be called ‘the electric slide’ or ‘the wobble’.

To generalize the BoS game, we first consider the scenario in which the game is played repeatedly among intermixing agents in a population, such that all three dyadic preference combinations are possible. Agents’ pay-offs in the game are given by their preference type: Bachites prefer Bach, Stravinskians prefer Stravinsky. Thus, the game is characterized by three pay-off matrices (Table 2), representing each possible pair-up:

**Table 2.** Generalized Bach or Stravinsky for a population of two types; 
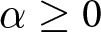

		**Bachite**	
		Bach	Stravinsky
**Bachite**	Bach	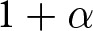 , 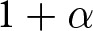	0, 0
	Stravinsky	0, 0	1, 1
		**Stravinskian**	
		Bach	Stravinsky
**Bachite**	Bach	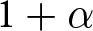 , 1	0, 0
	Stravinsky	0, 0	1, 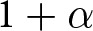
		**Stravinskian**	
		Bach	Stravinsky
**Stravinskian**	Bach	1, 1	0, 0
	Stravinsky	0, 0	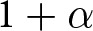 , 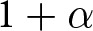

*Note:* Each cell lists the pay-offs to each player: first the row player, then the column player.

This generalization of the BoS game is equivalent to Neary (2012)’s Language Game, at least when restricted to two types of players with two actions and no social signals.

Since agents always get a pay-off of 0 when they fail to co-ordinate, we can more succinctly present agents’ pay-offs (Table 3) in terms of their preferences for successfully co-ordinating on each possible behaviour:

**Table 3.** co-ordination preferences: generalized BoS for a population of two types, 
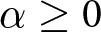

**Co-ordination preferences**	Bach	Stravinsky
Bachite	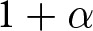	1
Stravinskian	1	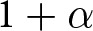

Table 3 expresses all of the information in Table 2, but does so much more concisely. This concise format will be particularly useful when we consider populations with more than just two preference types and more than two actions.

### 2.2. Evolutionary dynamics

In our first simulations, we initialized a population of 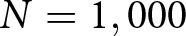 agents with a fixed distribution of preference types and randomly assigned a behaviour strategy to each (for the two-option BoS game, each player was initially assigned a strategy of playing either BoS without regard for preference). In all extensions of the model, we always begin simulations with agents whose strategy profiles are randomly assigned (Appendix H explores a version of the model with non-random initial conditions and finds similar results to the simulations with random initial conditions). The model dynamics proceeded in discrete time steps, using replicator dynamics under the assumption of a large number of interactions in a well-mixed population, such that agents who acquired higher pay-offs were more likely to transmit their strategies. These generic replicator dynamics allow us to remain uncommitted to the nature of the transmission mechanism or the timescale thereof – it could represent vertical parent-to-offspring transmission through genes or teaching, or success-biased social learning of older or same-age individuals (Smaldino, 2023). As detailed below, we think it is most intuitive to view this as pay-off-biased oblique social transmission.

On each time step, the prevalence of each strategy profile among a given preference type 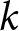 is adjusted according to a discrete replicator equation:

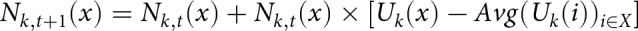
where, for a given preference type, 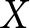 is the set of all strategy profiles, 
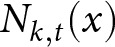
 is the number of agents of type 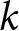 with strategy profile 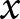 at time 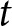, 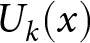 is the utility (expected pay-off) of strategy profile 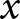, and 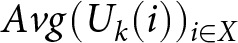 is the average utility of all the strategy profiles present among the given type.

The utility of strategy profile 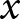 for preference type 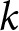 is calculated as

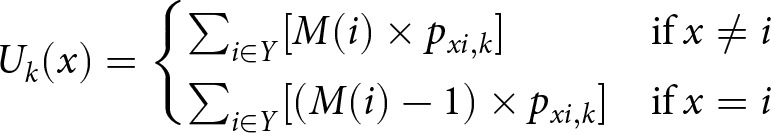
where 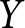 is the set of all strategy profiles present in the population, 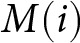 is the number of agents (of any preference type) in the population who play strategy 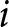, and 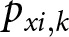 is the pay-off to an agent of preference type 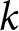 for playing strategy 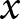 when paired with an agent who plays strategy 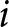. After adjusting the prevalence of strategy profiles, their quantity is re-normalized so that the number of agents of each preference type remains constant throughout a simulation. Thus, utility is calculated based on the assumption that an agent interacts with a large representative sample of all the other agents in the population.

The replicator dynamics perhaps most intuitively approximate agents inheriting their strategy profiles from among the highest performing agents of the previous generation. This does not require that agents can observe everyone’s preference type or strategy profile. Rather, it is justified by basic life history assumptions. Individuals are likely to learn their behavioural strategies during childhood, during which time most of the observed elders will be part of their local family groups and affiliative communities, and therefore likely to share their preference type. Evolutionary modelling suggests that such intragroup inheritance is often adaptive in diverse populations (Bisin et al., 2011; Smaldino & Velilla, 2025), and as implemented here only requires that pay-offs (or indicators thereof, such as prestige) are observable. Meanwhile, pay-offs are accrued during adulthood, during which time individuals are assumed to interact more widely with members of the entire population. The effects of more structured interaction networks are postponed for future research. In Appendix B, we present analyses in which individuals agents learn their strategy profiles via reinforcement learning rather than having those strategies evolve via replicator dynamics. We obtain qualitatively similar results, further adding to the robustness of our analyses.

We also allow for the possibility that an agent’s strategy profile is not copied faithfully, or ‘mutates.’ This is governed by two parameters, 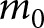 and 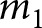. Each agent is selected for mutation with probability 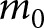. If selected, each element in the string expression of the agent’s strategy profile is assigned a random value from among the allowable values with probability 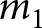. In the model described thus far, agents’ strategy profiles are represented by strings of length 1, and can either be 
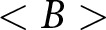
 (play Bach) or 
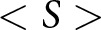
 (play Stravinsky). As the model is extended to allow agents to broadcast social signals and to choose their action based on the signal of a paired agent, the string expression of agents’ strategy profiles will have length greater than 1. Basic model parameters used in our simulation results are given in Table 4.

**Table 4.** Simulation parameter values used for the results presented throughout this paper

	Number of simulations	Simulation length, 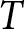	Population size, 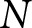	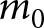	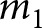	Signal cost, 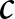
Figure 3	10,000	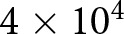	1,000	0.01	0.1	n/a
Figure 4	10,000	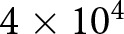	1,000	0.01	0.1	n/a
Figures 5 & 6	1,000	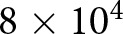	1,000	0.01	0.1	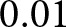
Figure 9	1,000	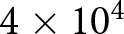	1,000	0.01	0.1	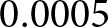
Figure 11	1,000	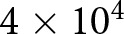	1,000	0.01	0.1	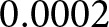
Figure 12	100	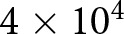	1,000	0.05	0.2	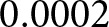
Figure 13	100	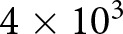	1,000	0.05	0.2	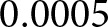

### 2.3. Simulation results: majority groups have power to make their preference the norm

We present our results using the Mesopotamian framing introduced earlier. We consider a population with two types of agents, Ummians 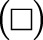 and Kishus 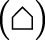. Ummians 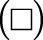 make up a proportion 
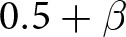
, with Kishus 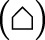 constituting the remainder. co-ordination preferences follow the generalized BoS game, such that members of each preference group receive a pay-off of 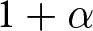 for co-ordinating on their preferred greeting, 1 for coordinating on their non-preferred greeting, and 0 for failing to co-ordinate (Table 5).

**Table 5.** Pay-offs for the baseline model with Ummians and Kishus

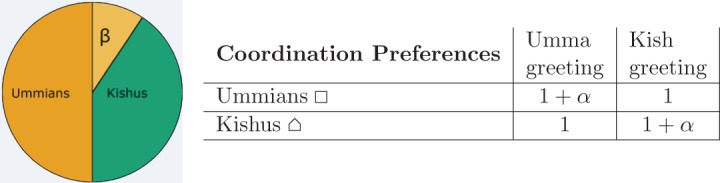

**Figure 3.**
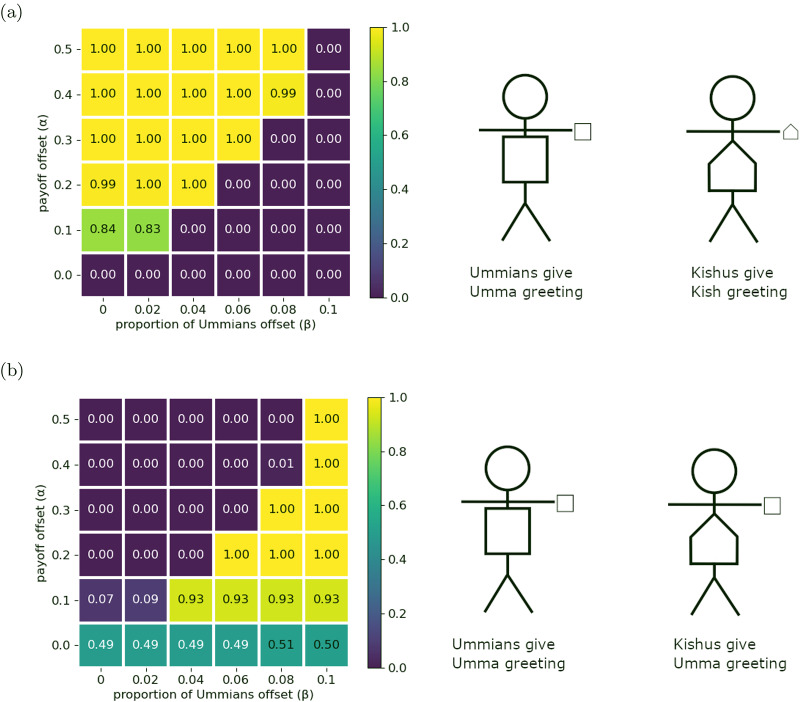
(a) Proportion of simulations in which agents always give their preferred greeting. (b) Proportion of simulations in which everyone gives Ummians’ preferred greeting.

Our simulations results are illustrated in Figure 3. In the absence of social signalling, agents will evolve to simply use whatever strategy yields the highest expected pay-off. Consequently, there are only two prominent types of outcomes in the simulations. When preferences are weak (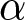 is small), everyone converges to using the same greeting because the benefit of always co-ordinating outweighs the benefit of always giving one’s preferred greeting but frequently failing to co-ordinate behaviour. It is important to note that our model is a stochastic system that can exhibit some path dependency. So a particular set of parameters, say 
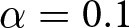
 and 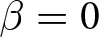, might result in 84% of simulations reaching the outcome depicted in Figure 3a, while other simulations with those same parameters resulted in different outcomes.

If the two preference groups are of unequal size, the equilibrium greeting will typically be that the majority preference group (the Ummians in this case). Correspondingly, when 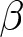 is large, the population will converge to all use the Ummian greeting for larger values of 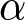, because for the Kishus, the higher pay-off for co-ordinating on their preferred greeting is outweighed by the higher probability of interacting with an Ummian (see also Neary, 2012, O’Connor, 2017). On the other hand, when preference groups are more evenly sized (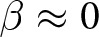) or co-ordinating on one’s preferred greeting is strongly incentivized (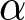 is large), each preference group will evolve to use their preferred greeting. When 
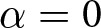
, the population evolves to co-ordinate on either greeting about half the time, as either co-ordinative equilibrium is equally good. In Appendix E, we show how these results can be derived analytically.

It may be helpful to think of this model as having three basins of attraction, one for each of the three equilibria. These equilibria are as follows: one in which each type plays their preferred greeting (Fig. 3a), one in which everyone gives the Ummian greeting (Fig. 3b), and one in which everyone gives the Kishu greeting (not depicted, but can be inferred as occurring whenever the other two equilibria do not obtain). Because each simulation was initialized initialized with randomly assigned strategy profiles, the proportion of simulations runs leading to each equilibrium can be interpreted as the relative size of each basin of attraction.

It is important to notice just how undesireable this behaviour is for agents. When agents of each type evolve to use their preferred greeting, they fail to co-ordinate (and receive a pay-off of 0) every time they interact with an agent of another type. And when agents all evolve to use the greeting of the majority preference group (the Ummians), interactions between two members of the minority preference group (the Kishus) always yield a lower pay-off than would occur if both individuals were to use their preferred greeting. This is why, in part, cultures have evolved to use social signals and ethnic markers (McElreath et al., 2003; Smaldino, 2019, 2025). As we will show in the following section, social signals allow people to condition their actions on those signals such that they successfully co-ordinate actions when interacting with individuals with differing preferences and co-ordinate on their preferred action when interacting with individuals who share their preferences. Even when a majority preference group makes their preference the norm, minority preference groups can still establish their own practices to use within their preference group.

Once a preference group becomes correlated with a distinct social signal (or combination of social signals) as well as a distinct way of acting in response to social signals, it makes sense to refer to the members of the preference group as a social group in the ordinary sense. Indeed, we think it would make sense to call the Ummians and Kishus separate groups in this section’s model when they are identifiable by their actions, i.e. when the outcome depicted in Figure 3a arises. However, when Ummians and Kishus look and act the same, as in the outcome depicted in Figure 3b, it seems odd to refer to them as separate groups. In this case, Ummians and Kishus are only differentiable from the omniscient modeller’s perspective. From the agent’s perspective, everyone looks and acts the same, and no one can tell that not everyone shares the same preferences.

## 3. Signals, signalling costs, and attention

### 3.1. Adding signals to the generalized BoS game

We next allow agents to broadcast social signals at the start of an interaction and to condition their action in the game upon their partner’s signal. Agents’ strategy profiles can be expressed as strings of length 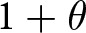, where 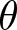 is the number of potential signals that can be received. The first element in this string represents the social signal that an agent broadcasts and the subsequent elements represent the action that is played when paired with an agent who broadcasts the corresponding signal. For example, suppose that there are two signals 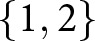 in the two-option BoS game. Then there are 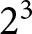 strategy profiles: 
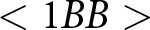
, 
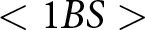
, 
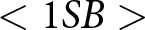
, 
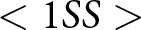
, 
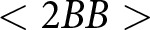
, 
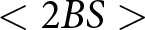
, 
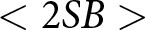
, and 
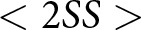
. The strategy profile 
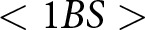
 corresponds to an agent broadcasting social signal 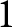, playing Bach when paired with an agent who broadcasts 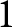, and playing Stravinsky when paired with an agent who broadcasts 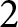. Note that individuals in this model broadcast their signals overtly without first knowing anything about their audience; we do not take into account covert or strategic signalling (Smaldino and Turner, 2022; Smaldino et al., 2018). Successful individuals in this extension of the model transmit their entire strategy profile, with mutation as described in Section 2.2.

### 3.2. Simulation results: social signals allow optimal behaviour and cross-cultural competence

We continue with the example of Ummians 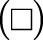 and Kishus 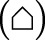 introduced in the previous section (Table 5), with the difference that agents now broadcast one of two social signals (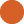 or 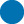) and choose their action based on the signal they observe from a partner. We continue with the assumption that agents are paired at random and use signals to condition their behaviours. Agents do not use signals assortatively to find interaction partners as in some previous models (e.g., Castro & Toro, 2007; McElreath et al., 2003; Smaldino et al., 2018). We view this assumption as reasonable, as individuals must often condition their behaviours on information received after an interaction has already begun. We analyse a version of our model with this type of assortment in Appendix A, and show that the results are qualitatively similar.

**Figure 4.**
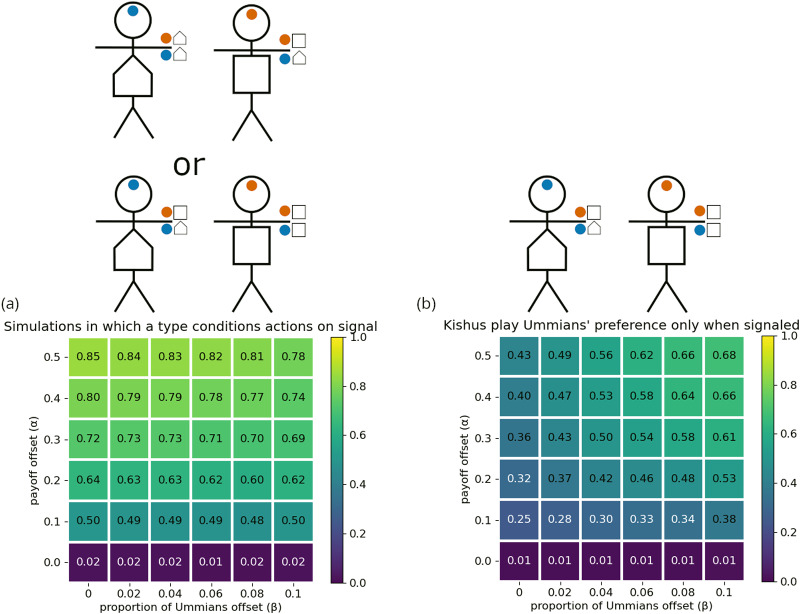
(a) Proportion of outcomes that are one of the two optimal outcomes. (b) Proportion of outcomes that are the optimal outcome favouring the Ummians.

Roughly speaking, an optimal outcome occurs when the highest net pay-offs possible, given the distribution of players’ incentives, have evolved. In these initial extensions of the model, we see that social signals allow agents to successfully co-ordinate in contexts where they frequently failed to co-ordinate in the absence of social signals (e.g. Fig. 3a). Social signals also allow agents to co-ordinate on their most preferred actions instead of on a less-preferred action (e.g. Fig. 3b). After we have fully described all components of the model, Section 4.2 gives explicit criteria for what we designate as an optimal outcome; this later definition is consistent with what we call optimal in this section. For now, we emphasize that our use of ‘optimal’ is not reflective of anything like an ethically prescribed group structure. For example, we sometimes find that the preferences of majority groups become normative for all intergroup interactions (e.g. Figs. 4b and 6). We do not think this is optimal in any prescriptive sense.

Broadly described, our analyses show that individuals almost always learn to use signals for optimal co-ordination, illustrating an often theorized use for identity signals (Boyd & Richerson, 1987; McElreath et al., 2003; Smaldino, 2019, 2025). When Ummians interact with other Ummians, they signal their identity and give the Umma greeting. Likewise, when Kishus interact with other Kishus, they signal their identity give the Kish greeting. But when Ummians interact with Kishus, the population still has to settle on whether to co-ordinate on the Umma greeting or the Kish greeting. The majority population is still advantaged in this case. If the Ummians outnumber the Kishus, then the population is more likely to settle on the Umma greeting as the norm when people of different types interact.

There are two types of optimal outcomes in this model, each of which involves agents successfully co-ordinating on their preferred greeting when interacting with partners of the same type, and otherwise settling on one of the two available greetings when interacting with partners of another type. Figure 4a shows the proportion of simulations that resulted in optimal outcomes of either type, and Figure 4b shows the proportion of simulations resulting in the subset of these outcomes favouring Ummians 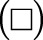. In Figure 4a, we see that simulations more reliably converged to optimal outcomes when agents were increasingly incentivized to co-ordinate on their preferred greetings (larger 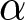). In Figure 4b, we see that increasing 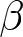 (the proportion of Ummians) made outcomes favouring Ummians more frequent, just as in the no-signal case. When both types were present in equal proportions (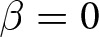), each optimal outcome was equally likely to occur. In the remaining proportion of simulations (1 – the value in Fig. 4a), meaningful signals did not evolve and either all agents in the population always gave the Ummian greeting irrespective of the social signal seen or all agents in the population always gave the Kish greeting irrespective of the social signal seen. No simulation resulted in each type of agent always giving their preferred greeting and then failing to co-ordinate when interacting with agents of a different type, as was frequently the case in the base model.

These results support similar findings from earlier models. Prior modelling work (Bunce & McElreath, 2018; O’Connor, 2019) has suggested that the ability of minority groups to maintain their cultural norms is tenuous at best, which accords with our results from the no-signal model in the previous section. Using a more complex model than Bunce and McElreath (2018), however, Bunce (2021) demonstrated that a minority group can nevertheless maintain their cultural norms if they are able to competently adopt either the majority or minority norms depending on the context, which Bunce calls cross-cultural competence. Our model also demonstrates the emergence of this sort of cross-cultural competence when agents are able to condition their actions on identity signals (Fig. 4b). Our model compliments Bunce (2021)’s account of cross-cultural competence by allowing an extra degree of freedom where social signals and conditional actions can evolve independently rather than assuming the existence of a single phenotype that packages each of these components together.

While our simulations frequently resulted in optimal outcomes, as shown in Figure 4a, they did not *always* result in optimal outcomes. When a simulation did not result in an optimal outcome, it was always the case that every agent in the population was giving the same greeting all of the time. This helps illustrate two important points. First, our simulations are stochastic. Our model shows that under certain conditions – e.g. when there are two different preferred ways to co-ordinate and there is an available means of signalling identity – agents are *likely* but not guaranteed to adopt social signals to facilitate co-ordinating in their preferred way when they are among others who share their preferences. Second, it shows clearly how group structure is not hard-coded but rather emerges in our model.

### 3.3. Adding attention and signal costs to the generalized BoS game

Thus far, we have assumed that the production of signals and the ability to attend to and condition actions upon those signals is trivial and costless. However, it is often the case that social signals require some effort to broadcast or attend to. In such cases, only those groups for whom the social information is relevant should be expected to invest the effort to engage with the signals. For example, individuals holding fringe or extreme political opinions may invest much more effort to differentiate themselves from the mainstream, while those in the mainstream pay little heed to those same distinguishing characteristics (van der Does et al., 2022). To capture this, the model is extended to include a special signal, 0, which indicates an agent is not attending to signals, and a signal cost, 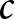, that an agent incurs if he/she broadcasts any signal other than 0. When an agent does not attend to signals (i.e. when he/she broadcasts 0), he/she interacts with all other agents as if they had also broadcast 0. In other words, he/she ignores all signals, so that actions cannot be chosen based on the social signal that was broadcast. When an agent uses a non-zero signal, its pay-off from an interaction is decreased by 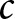.

As formalized here, an agent that does not condition his/her actions on signals will necessarily also fail to send a signal, and vice versa. In the real world, these may be independent dispositions. A person might abstain from broadcasting a social signal but still use social signals to determine what action to perform. Conversely, they might broadcast a social signal but ignore signals from others when choosing what action to perform. Combining these two dispositions so that they always co-occur is computationally convenient, plausible for at least some social contexts, and produces some interesting and novel social outcomes. We leave investigation of a model with independence between signalling and attention for future research.

For this extension, we introduce a third type of agent into the population: the Akkadians 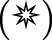, who by default make up a third (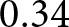) of the population. Ummians then 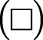 make up a proportion 
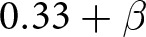
 of the population, with Kishus 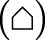 constituting the remainder. We will compare these to simulations without the presence of Akkadians. Recall from our introduction that Akkadians are from a different ethnolinguistic group than the Ummians and Kishus, who are both Sumerian. We therefore model their preferences so that they receive no pay-off from co-ordinating on greetings other than their own. The Kishus and Ummians, however, being threatened by the military might of the Akkadians, receive some pay-off from co-ordinating on their greeting, though not as much as co-ordinating on another Sumerian greeting (Table 6). Our intention in introducing this third preference type is to consider scenarios in which group identities are nested within one another. For example, Akkadians might perceive the world as divided into two groups – themselves and the Sumerians – whereas the Ummians and Kishus would take umbrage with that delineation, highlighting important differences between their two groups and insisting that the world is instead divided into *three* groups.

**Table 6.** co-ordination pay-offs for the model with three preference types

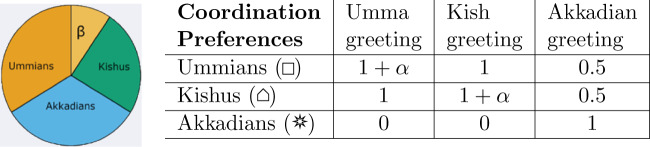

### 3.4. Simulation results: majority groups ignore social signals

**Figure 5.**
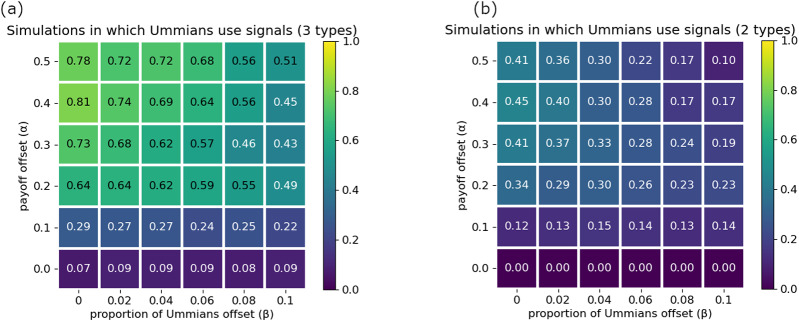
(a) Proportion of outcomes in which Ummians 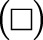 broadcast a social signal, for the model with three types of agents (see Table 6). (b) Proportion of outcomes in which Ummians 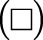 broadcast a social signal, for the model with two types of agents, in which Ummians constitute 
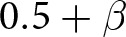
 of the population and Kishus constitute the remainder. In all cases, 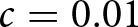.

Our analyses show that the presence of the Akkadians 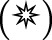 can increase the likelihood that the Ummians 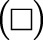 and Kishus 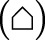 use social signals for co-ordination. Figure 5 shows the proportion of simulations in which Ummians reliably broadcast and attend to signals. When only Ummians and Kishus are present, the Ummians tend to ignore signals and simply use their preferred greeting, particularly when they are in the majority or there is a weaker incentive to co-ordinate on the Umma rather than the Kish greeting (Fig. 5b). In contrast, when Akkadians are present, Ummians are much more strongly incentivized to signal, both because they constitute a smaller proportion of the population, and because the Akkadians are less willing to adopt the Ummian greeting, making it important for the Ummians to condition their actions on their interaction partner. As elsewhere, it is the group that is least willing to budge that drives the rest of the population dynamics (Bergstrom & Lachmann, 2003; O’Connor, 2017).

**Figure 6.**
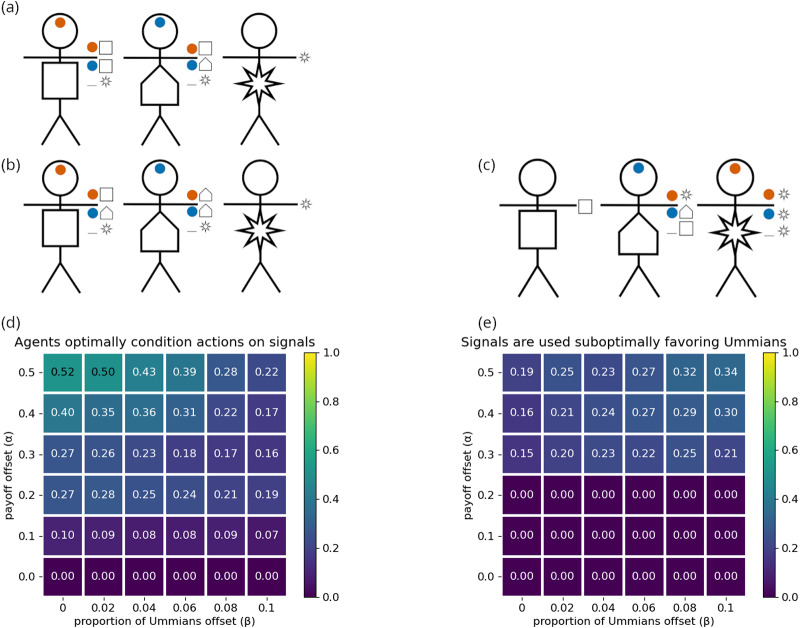
Majority groups ignore social signals. (a) and (b) depict the only two optimal outcomes, while (c) depicts a prominent suboptimal outcome. (d) Proportion of simulations that resulted in either of the two optimal outcomes. (e) Proportion of simulations that resulted in the prominent suboptimal outcome in which Ummians do not use signals.

With three preference types, three action options, and three signalling options, there were many possible outcomes for our simulations. While over half of these rarely occurred, our stochastic simulations yielded a total of 13 different outcomes that occurred at least 0.5% of the time. All of these outcomes are given a detailed description in Appendix G. Here we focus on the three most prominent outcomes. The first of these is suboptimal, in which Ummians 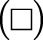 and Akkadians 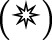 always played their respective preference, and Kishus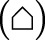 played the Umma greeting with Ummians, the Kish greeting with other Kishus, and the Akkadian greeting with Akkadians. This scenario is illustrated in Figure 6c, in which an agent’s signal is depicted in their head, and their actions conditioned on signals received are depicted to the right of their hand. The null signal (0) is represented as an underscore. The prevalence of this outcome is shown in Figure 6e. It is characterized by Ummians 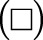 signalling 0 and Kishus 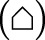 and Akkadians 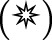 attending to signals that reliably identified their signaler’s type (but with only Kishus conditioning their actions on the signals they received). One can check that this is a Nash equilibrium. It is suboptimal in the sense that when Ummians 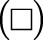 are paired with Akkadians, there is a failure to co-ordinate. We observed that there was some path dependency that locked in this outcome. Once sufficient numbers of Ummians evolved to ignore signals, Akkadians faced increased selection to signal in order to allow the Kishus to condition their responses on the Akkadians’ signals in order to co-ordinate more effectively.

The remaining two outcomes highlighted here are optimal in the sense that there were never failures of co-ordination and agents played their most preferred greeting among themselves, illustrated in Figure 6a and b. In both cases, Akkadians ignored signals and always used their preferred greeting, where as both of the Sumerian types successfully used signals to co-ordinate with Akkadians on the Akkadian greeting, co-ordinate with their own type on their preferred greeting, and to co-ordinate with the other Sumerian type with either the Umma or the Kish greeting. The combined frequency of these outcomes is shown in Figure 6d. This scenario was most likely to occur when the Ummians constituted a smaller proportion of the population and when Sumerians had a stronger incentive to co-ordinate on their own preferred greetings when possible.

Overall, these analysis indicate that larger or more stubborn groups are more often able to ignore social information and simply adopt their preferred actions, while members of minority groups have more incentive to use social signals and to condition their actions on social information. This coheres with the empirical evidence that majority groups, such as whites or heterosexuals in the United States, often do not think of themselves as having any specific social identity and take their norms to be ubiquitous (Devos and Banaji, 2005; Simoni & Walters, 2001). Although this behavioural outcome is optimal in the context of the model, it translates to substantive inequality as minorities face disadvantage in the cognitive load of code switching between norms (Johnson et al., 2022) in addition to the need to often co-ordinate on their non-preferred norms. If, for the ‘optimal’ outcome favouring Ummians (Fig. 6b), we assume each agent type is an equal proportion of the population and 
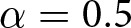
, then we can calculate the inequality between Ummians, who have an expected pay-off of 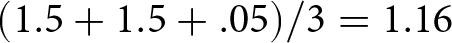, and Kishus, who have an expected pay-off of 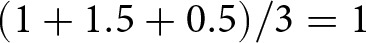. In other words, an optimal allocation of co-ordinative behaviour does not imply equity.

### 3.5. Signalling systems indicate affiliations; actions capture power structures

At this point, we wish to introduce a formal distinction between a *signalling system* and a *group structure*. By ‘signalling system’, we refer to the system of social signals that agents use to co-ordinate their actions. A signalling system is fully described when each social signal present in the population has been paired with the subset of all agents in the population that use the social signal. For example, Figure 6a depicts a signalling system in which all Ummians broadcast the red signal and all Kishus broadcast the blue signal. Figure 6b exhibits the exact same signalling system. Signalling systems are often a good first indicator of what groups are present in a population. However, knowing the signalling system used in a population may be insufficient to identify all the groups in the population, as signals may require context or specific knowledge to identify (Smaldino & Turner, 2022; Smaldino et al., 2018). Moreover, knowledge of signalling systems may reveal little about the power dynamics between groups. For example, they do not tell us whether a minority group yields to the norms of the majority. For this, we need information about the actions agents take in response to observing social signals.

We refer to the ‘structure’ of a population to include not only the signalling system but also agents’ full strategy profiles – both the social signals broadcast and the actions they take in response to observing various social signals. For example, while Figure 6a and b shows the same signalling system, they show different structures. In Figure 6a, an Ummian gives the Umma greeting when interacting with a Kishu. In Figure 6b, they give the Kish greeting. We can give the same treatment to Figure 4a, which depicts two different structures with the same signalling system. A focus on structures highlights that identity is more than the signals people use, it is also the actions they take (and expect) in response to those signals. These structures capture what particular signals imply for co-ordination and power between different groups of people. In some sense, we are arguing against a type of essentialist thinking regarding identity (Smaldino, 2025). While we use preferences types as labels to describe the distributions of agents in our model, we also try to take the perspective of the agents themselves and consider how they would categorize the agents in their world. For example, a person who had Kish preferences but the signalling/behaviour strategy profile of a typical Ummian would be would be indistinguishable from other Ummians. This perspective will become especially important in the next section, in which we focus on more complex structures requiring more complex signalling systems.

## 4. Multidimensional signalling and complex group structures

In previous sections, our analysis was restricted to one-dimensional, discrete signals of identity. Here we extend the model to allow multiple aspects of identity to be signaled simultaneously to allow for nested or intersectional identities. Individuals can therefore signal multiple identities because they have access to multiple modes of signalling. We will explore the implications of this sort of multidimensional signalling and show how it affords more complex identity structures.

We will continue to use cities and ethnicities from ancient Mesopotamia to designate the different preference types we consider in our model. Here we briefly review our six Mesopotamian groups and summarize their relationships with one another. Each of the four cities depicted in Figure 1 are Sumerian 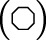 cities, united by a common language and religious pantheon. Umma 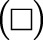 and Lagash 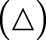 are rivals; they have different local gods and regularly feud over the fertile farmland between the two cities. The temple of the Lagash deity Ningirsu is in the city of Girsu, and we can think of the Girshites 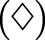 as a particular devout and zealous offshoot of the Lagashites. Meanwhile, the more distant city of Kish 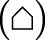 traded with both the Ummians and the Lagashites, and so can be considered a neutral third party, sharing features with both of the other Sumerian communities. We will use the Kishus alternately to illustrate (1) a group whose members are simply the conjunction of the people who like the Ummian greeting and those who like the Lagashite greeting, and (2) a group whose members share some preferences with both Ummians and Lagashites but retain distinct preferences of their own. Finally, the Akkadians 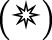 were a non-Sumerian civilization who would eventually go on to conquer all of Mesopotamia. Due to their military superiority, we treat the Akkadians as highly intolerant of the norms of other groups, while Sumerians would tend to be at least somewhat tolerant of the Akkadians.

### 4.1. The model with multiple signalling dimensions

We extend the model to allow agents to signal in up to 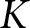 dimensions. signalling in each dimension is an independent decision, and for each dimension agents can choose to signal and attend to the signals of others, or else refrain from signalling and attending to signals in that dimension. A person can ignore sports iconography but still be attuned to symbols of religious affiliation. We assume the signal cost 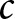 is incurred for each dimension attended to. Adding even one more signalling dimension is sufficient to add considerable complexity. For example, if there are three *de facto* signals (two real signals plus the null signal) in each of two dimensions, then there are nine different social signals an agent can broadcast. Strategy profiles must therefore include contingent actions for each of the signal combinations an agent can perceive; thus, strategy profiles are now strings of length 
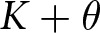
, where 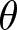 is the number of possible signals combinations. To keep things as simple as possible, we will focus primarily on scenarios with 
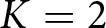
, where each dimension has only one signal plus the null signal, for a total of four possible social signals, though we will at times allow for more than one signal possibility in each dimension. Otherwise, the basic replicator dynamics and utility function of the model are the same as described in the previous sections.

**Figure 7.**
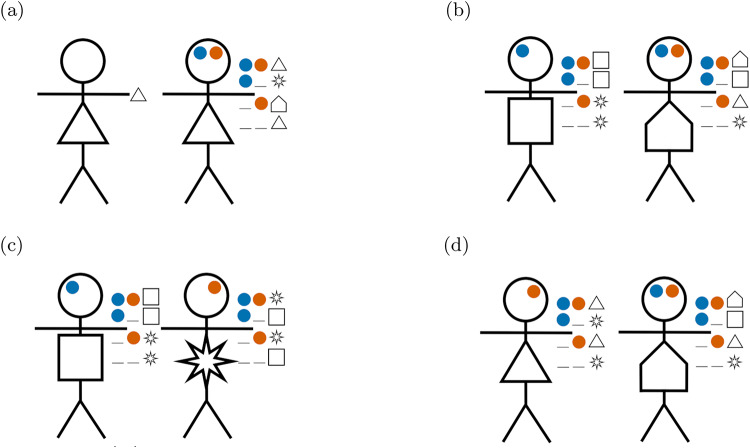
Diagram examples of interactions with multidimensional signals. (a) Successful co-ordination, both agents give the Lagash greeting 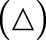. (b) Successful co-ordination, both agents give the Umma greeting 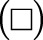. (c) Agents fail to co-ordinate, as each attends to a different signalling dimension and the agents have incompatible strategy profiles. (d) Successful co-ordination, both agents give the Lagash 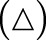 greeting.

Figure 7 illustrates how the stick figure diagrams are extended to accommodate multidimensional signals. Agents’ preference types are indicated by the shape on their body, though these are not observable to others. During an interaction, each agent perceives the social signal(s) that their partner broadcasts, depicted on each stick figure’s forehead. If an agent does not pay attention to a dimension of social signalling, then no signal for that dimension is shown on the agents’ forehead. Finally, an agent’s action in response to a given signal is depicted adjacent the given signal near the stick figure’s hand. If an agent does not pay the cost of attending to a signalling dimension, then he/she neither signals in that dimension nor conditions his/her actions on signals in that dimension. Figure 7c illustrates a scenario where agents fail to co-ordinate, because each agent is signalling using a dimension that their interaction partner does not attend to. The other subfigures all display scenarios in which the interaction leads to successful co-ordination.

In the remainder of this section, we will consider how the multidimensional signalling model can represent the identity structures discussed in Section 1 and depicted in Figure 2. Our analysis in this section is more illustrative than thorough, but it will highlight the importance of sufficient modelling complexity in capturing these identity structures and suggest how empirical research using signalling behaviour alone may fail to discover deeper identity structures.

### 4.2. The single embedding structure and the benefit of multiple dimensions

We will begin with a case inspired by the idea of one group being embedded within a larger group, whose members interact with a third distinct group. Of course, we do not model this embedding explicitly; instead, we construct three types of individuals whose preferences are *indicative* of this embedding. Consider the co-ordination preferences shown in Table 7. Recall that the Girshites can be viewed as a particularly zealous sect of Lagashites. The Girshites have their own distinct greeting on which they most prefer to co-ordinate. Additionally, given the affinity between Girsu and Lagash, Girshites consider the Lagash greeting the next best thing if they cannot co-ordinate on the Girsu greeting. Both Girshites and Lagashites will grudgingly adopt the Akkadian greeting if no other option is available, while the Akkadians do not benefit from co-ordinating on any greeting but their own. We will use this example to show that allowing agents multiple dimensions of social signals makes it much easier for them to find optimal methods of co-ordination.

**Table 7.** Co-ordination preferences for the single embedding subsection

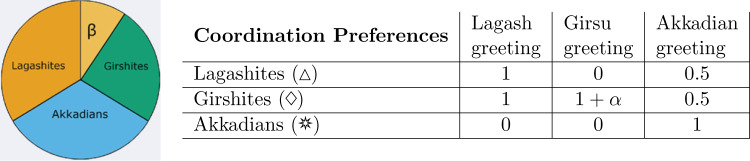

We designate a group structure as optimal if the following three conditions are met:

- Given any two agents, if there is some action for which they both receive non-zero pay-off, then they always successfully co-ordinate on an action for which they both have non-zero pay-off.
- Given (i), that action provides the maximal possible sum of the two agents’ pay-offs.
- There are no other group structures that meet criteria (i) and (ii) while also allowing agents to pay a lower attention cost.

This definition is tailored to the specific group structures that we investigate. Circumstances not involving co-ordination among large groups would require different metrics. The scope of our analysis is therefore restricted to dyadic co-ordination games. It is likely that most or all of the optimal outcomes discussed in Section 4 are Pareto optimal (See Neary, 2012), but our definition is not fully equivalent since there are scenarios in which an outcome that benefits one player without providing a benefit to the other would fail to evolve. For example, suppose there are two preference types such that no action exists for which both types receive non-zero pay-off. Then, in such a scenario, our model would produce outcomes in which agents of the two preference types fail to co-ordinate, given they have no incentive to do so. Such an outcome is not Pareto optimal because you can make a Pareto improvement. This can be done by changing the agents actions such that the two given preference types co-ordinate on an action for which one type has a non-zero pay-off despite the other type having zero pay-off for the action. In this case, one type would improve their pay-off while the other’s pay-off would not decrease. However, our definition optimality allows us to consider as optimal an outcome in which the two preference types fail to co-ordinate, given there is no action for which they *both* have non-zero pay-offs, and therefore neither type has any incentive to unilaterally change their actions.

In Appendix C, we explore an alternative optimality criterion (ii) requiring that an action is considered optimal only if there is no other action for which both agents receive a strictly greater pay-off. There are a small number of conditions for this the two definitions give slightly different results. However, for the cases we consider in this paper, either criterion is sufficient for identifying, from among the outcomes that are produced in simulations, those outcomes with the highest overall net pay-off.

**Figure 8.**
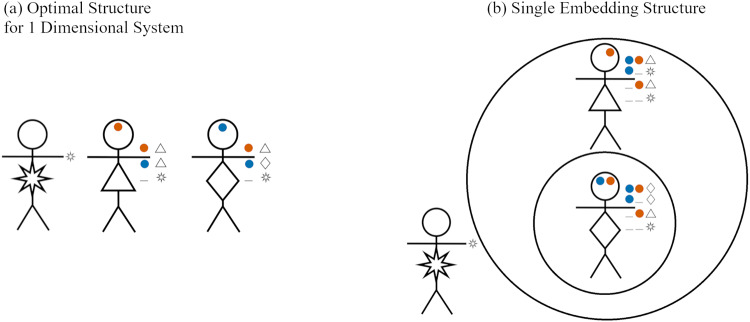
Optimal signalling systems and actions for the co-ordination preferences shown in Table 7, when agents have access to either (a) one or (b) two signalling dimensions.

Figure 8 depicts optimal group structures for the co-ordination preferences given in Table 7 for agents afforded either (a) one signalling dimension with two non-null options or (b) two signalling dimensions with one non-null option in each dimension. By inspection, each of these structures lead to the exact same outcomes for co-ordination between the possible dyads. Moreover, with two dimensions, the Girshites ending up paying higher attentional costs. Nevertheless, this outcome can be favoured over the one-dimensional system. The reason is because it is much more likely that the optimal group structure evolves if agents have access to more than one signalling dimension.

**Figure 9.**
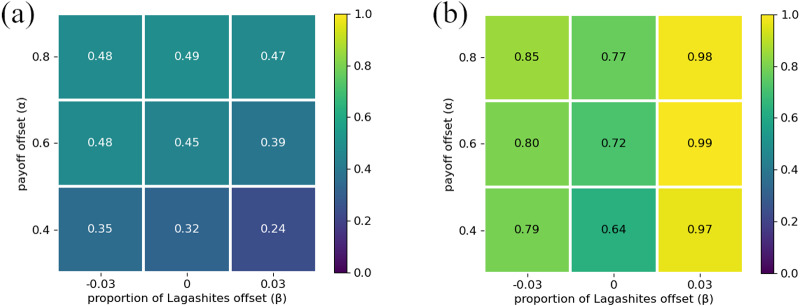
The proportion of simulations that resulted in the optimal outcome when agents had access to either (a) one or (b) two signalling dimensions.

Figure 9 shows the proportion of simulation runs in which agents evolved optimal group structures for (a) one or (b) two signalling dimensions. Our results show that it is substantially easier for agents with multiple signalling dimensions to arrive at an optimal structure. When only allowed one signalling dimension, the Lagashites 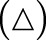 must learn responses to two different social signals: red (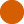) and blue (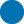). When agents are allowed two signalling dimensions, Lagashites only need to learn how to act in response to a single social signal 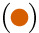. Next, consider the Girshites. Suppose the Lagashites have already found a social signal, red 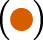, that allows them to optimally co-ordinate among themselves. With two signalling dimensions, the Girshites can easily optimize their interactions with the Lagashites by broadcasting the same red signal and giving the Lagash greeting in response to that signal. This does not depend on any change in behaviour from the Lagashites. Adding the blue (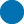) signal in a separate dimension allows them to co-ordinate among themselves. As we’ve already observed, majority preference groups are prone to ignoring the social signals of minority preference groups. With multiple dimensions of social signals, Girshites can broadcast their shared identity with the Lagashites while using a second dimension of signals to optimize co-ordinate among themselves. This is impossible with one-dimensional signals. Multidimensional signals allow the Girshites to signal their shared identity with the Lagashites while the Lagashites ignore the social signal unique to the Girshites, indicative of a niche subgroup embedded within a larger community. More generally, our model suggests that 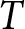 levels of nested embeddings in the group structure require 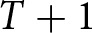 dimensions of social signals are required.

### 4.3. The intersectional structure and the importance of actions

Next, we consider an example inspired by the idea of overlapping or intersecting identity groups, and show how two distinct group structures can exhibit identical signalling systems. Our analysis will highlight how observing only social signals, without accounting for co-ordinative behaviours, can lead to overlooking intersectional identities.

Consider the co-ordination preferences shown in Table 8. Recall the narrative that Ummians and Lagashites hate each other, and so receive no pay-off from co-ordinating on each others’ greetings. Kishus, on the other hand, are happy to give either the Umma or Lagash greeting. But notice this is compatible with two distinct Kishu identities. Kishus could be a blend of Ummians and Lagashites, happily engaging in either greeting, and without any distinct greeting they prefer to use among themselves (
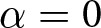
). Alternatively, Kishus’s preferences may be more than the mere conjunction of Ummian and Lagashite preference. There may instead be a Kish greeting that Kishus prefer above all others (
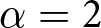
).

**Table 8.** Co-ordination preferences for the intersectional subsection

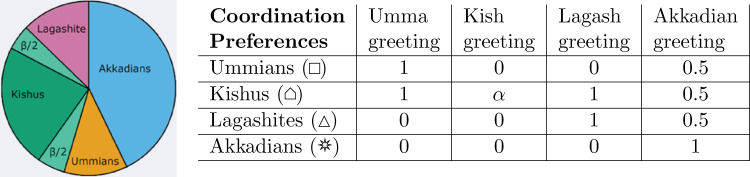

*Note:* Kishus are a proportion 
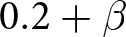
 of the population, Ummians and Lagashites are each 
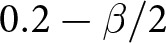
 proportion of the population, and Akkadians the remaining 40% of the population.

The connection to intersectionality should be clear. When the Combahee River Collective formed, it was because there were issues faced by black women that were not being addressed by mainstream feminism nor by Black civil rights movements (Combahee River Collective, 1977). People who were members of the two groups wanted to co-ordinate in a way that was not reducible to the conjunction of the opportunities for co-ordination afforded them by either group in isolation. This is clearly a different type of group structure than, for example, a group of people who are both rock climbers and sociologists, who may co-ordinate on activities related to either group but do not have any additional opportunities for co-ordination afforded them in virtue of being members of both groups.

**Figure 10.**
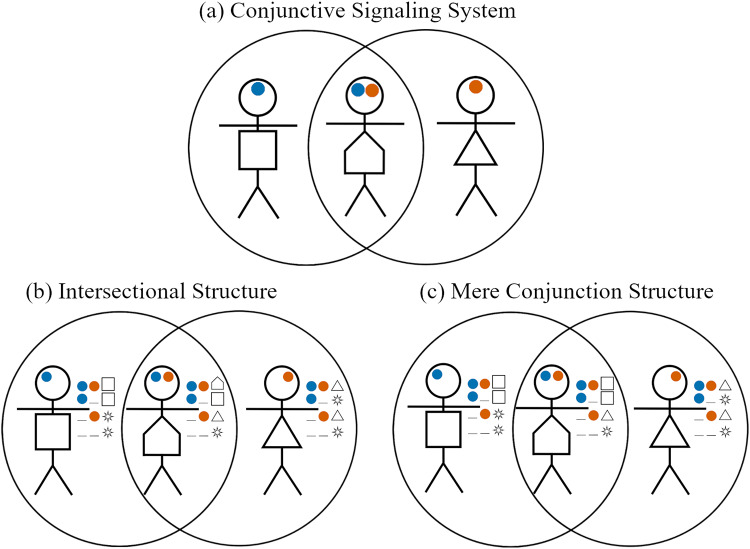
The conjunctive signalling system (a) can be indicative of two distinct group structures: one in which Kishus use their own preferred greeting among themselves (b), and one in which they use the Ummian greeting (c).

**Figure 11.**
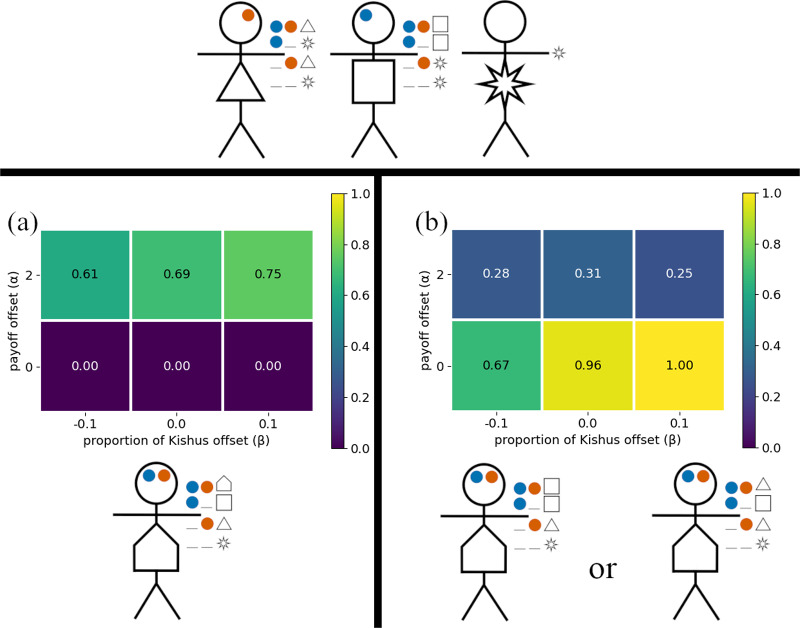
Proportion of simulations that resulted in (a) the intersectional structure, optimal when 
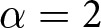
, or (b) either of the two mere conjunction structures, optimal when 
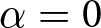
. The trio of stick figures at the top represent the optimal strategy profiles used by non-Kishus in all cases.

As in the previous section, we find that optimal outcomes evolve more frequently when agents are allowed multidimensional signals (see Appendix I). Here we only show results for simulations in which agents were allowed multidimensional signals, one signal in each of two dimensions. For all parameters shown here, at least 95% of the simulations resulted in the conjunctive signalling system (Fig. 10). When Kishus did not have a greeting that they preferred over the Umma and Lagash greetings (
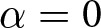
), they evolved to use one of these among themselves. The proportion of simulations that resulted in either of these two group structures is shown in Figure 11b. When Kishus did have a strong preference for a distinct greeting (
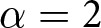
), then the optimal structure is the intersectional structure shown in Figure 10b. The proportion of simulations that resulted in this structure is shown in Figure 11a.

Our interpretation of intersectional identity aligns with the suggestion by Varley and Kaminski (2022) that intersectional identities can be identified as having a greater proportion of total information being synergistic information. Synergistic information is a formal measure of the information gained when considering the combined effect of multiple sources of information. In this case, information from what was signaled in the first dimension combined with information from what was signaled in the second dimension. Using partial information decomposition (Williams & Beer, 2010) and focusing on Varley and Kaminski’s definition of synergistic information, we found that, for 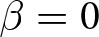, the synergistic information in the intersectional equilibrium, (Fig. 11a) was around 49% of the total information. Compared to this, the synergistic information in the equilibria for which Kishus were merely members of both groups (Fig. 11b) averaged only 36% of the total information.

Figure 11 shows that simulations frequently resulted in optimal outcomes. However, optimal outcomes became less frequent when Kishus constituted a smaller proportion of the population. We observed in Section 3.4 that large majority preference groups can evolve to ignore social signals, while minority preference groups evolve to attend to them. Here, we see a limit to this effect. When Kishus are a smaller proportion of the population, they have fewer opportunities to learn the relevant response to conjunctive social signals to obtain the optimal structure. This suggests that in the real world, there may be minority intersectional identities (with respect to individuals’ preferences) that we are simply unable to identify because they are too small for their members to learn the strategy profiles that would identify them as an intersectional group. We note that this result is obtained in the absence of ingroup homophily, which could provide more opportunities to learn group-relevant signals, but which would also require these potential intersectional groups to have some mechanism for assortment other than signalling.

If we assume that people exhibit optimal behaviour given their preferences, it is likely that their social signals will underdetermine the population’s structure. Therefore, if empirical researchers only consider the group labels or social signals that people use, then an intersectional structure and a mere conjunction structure will look identical. Furthermore, a biased sampling of interaction pairings can skew inference. In the conjunctive signalling system, for example, Kishus look like Lagashites from the perspective of Lagashites and like Ummians from the perspective of Ummians. This gives more reason to think we are prone to overlook some identities.

To overcome these issues, attention must be paid to how people co-ordinate their actions with different partners. This involves anticipating unique group-specific behavioural domains by conducting inductive assays on what individuals hope others say, react, or do during an interaction. Our model shows this is especially important in an intersectional group, such that a bottom-up approach to documenting co-ordination foci is imperative across the range of group membership.

### 4.4. Double embedding structures

We refer to scenarios in which more than one subgroup is embedded in a larger group as double embedding structures. Here, we compare two different types of double embedding group structures, and illustrate the fact that there are social structures in nature that cannot be adequately modelled without agents being afforded sufficiently complex social signals.

**Figure 12.**
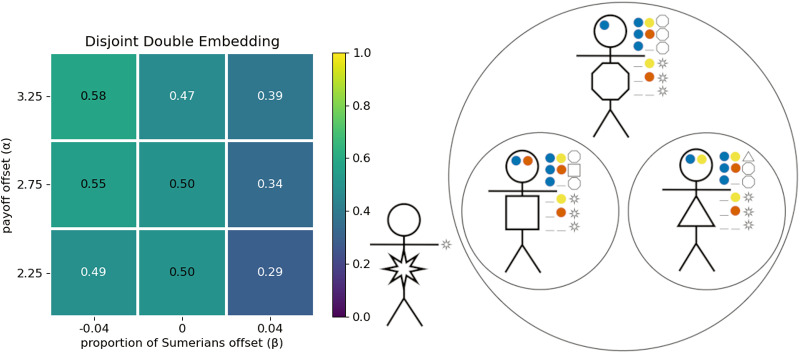
Proportion of simulations that resulted in the disjoint double embedding structure.

Consider the co-ordination preferences in Table 9. While Ummians and Lagashites get no benefit from each other’s most preferred greeting, they are both Sumerian and so prefer a generic Summerian greeting over the Akkadian greeting. In this scenario, we allow agents signals in two dimensions such that they have access to one non-null signal in the first dimension and two non-null signals in the second. In the optimal structure, this will allow agents to use that one signal in the first dimension to indicate the Sumerian identity that is shared between Ummians, Lagashites, and Sumerians at large; and, it will allow Ummians and Lagashites to signal their differences by using different non-null signals in the second dimension. We call this the disjoint double embedding structure (Fig. 12b) and it is the unique optimal outcome. Figure 12a shows that the disjoint double embedding obtained in about half the evolutionary simulations we ran.

**Table 9.** Co-ordination preferences for the subsection on the disjoint double embedding

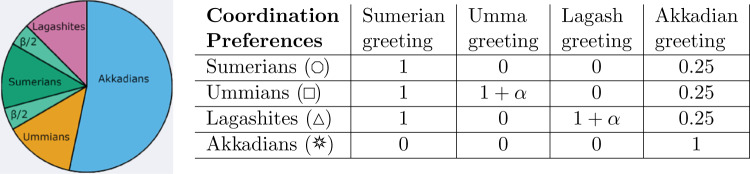

*Note:* In simulations, Sumerians were 
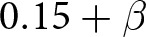
 proportion of the population, Ummians 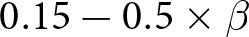 proportion of the population, Lagashites 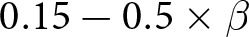 proportion of the population, and Akkadians the remaining 55% of the population. Agents were allowed one signal in the first dimension and two in the second.

**Figure 13.**
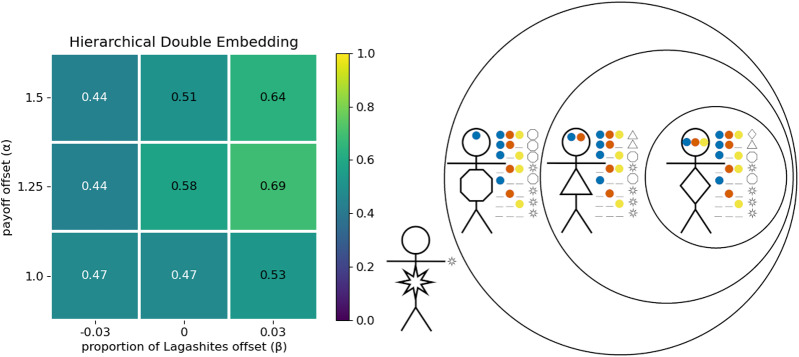
Proportion of simulations that resulted in the hierarchical double embedding structure.

Now consider the co-ordination preferences in Table 10. Recall that the Girshites can be viewed as a sect of particularly zealous Lagashites. The Girshites have their own distinct greeting which they most prefer to co-ordinate on. Girshites also prefer the Lagash greeting over the generic Sumerian greeting, which in turn is preferred over the greeting of the foreign Akkadians. In this scenario, we allow agents one non-null signal in each of three dimensions. Allowing agents three dimensions of signals allows three degrees of identity specificity to be broadcast at once. It allows Girshites to broadcast a signal specific to themselves in one dimension, a signal shared with the Lagashites in another, and a signal shared with all Sumerians in a third dimension. So Sumerians can optimally co-ordinate by recognizing the Sumerian social signal broadcast by Lagashites and Girshites without having to know anything about the signals that are specific to Lagashites and Girshites. Likewise, Lagashites can optimally co-ordinate with Girshites by recognizing the Lagashite signal without having to know about anything specific to the Girshite identity. And Girshites can optimally co-ordinate among themselves using their own signal. Given agents preferences, the hierarchical double embedding structure (Fig. 13b), is the unique optimal outcome. Figure 13a shows that the hierarchical double embedding obtained in about half the evolutionary simulations we ran.

**Table 10.** Co-ordination preferences for the hierarchical double embedding

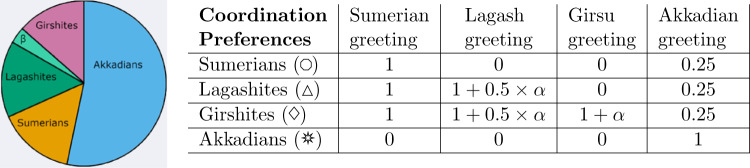

*Note:* In simulations, Sumerians were 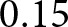 proportion of the population, Lagashites 
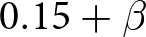
 proportion of the population, Girshites 
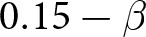
 proportion of the population, and Akkadians the remaining 55% of the population. Agents were allowed one signal in each of three dimensions.

Both double embedding group structures showcase complex signalling and behavioural phenomena that could not be captured by a simpler model with only one dimension of social signals. It is not just the case that multidimensional signals make it easier for agents to arrive at optimal outcomes. In addition, the *ways* that they arrive at optimal outcomes depends on their signalling affordances. In the disjoint double embedding structure, Ummians and Lagashites just look like Sumerians from the perspective of Sumerians, because they all broadcast the same social signal. This cannot be represented in a model with just one dimension of social signals. In the hierarchical double embedding, from the perspective of the Sumerians, Lagashites and Girshites look like Sumerians because of their shared social signal; Girshites look like Lagashites to the Lagashites, while from the perspective of Girshites, they are distinguishable from the two groups. Exhibiting these three perspectives in a single structure is only possible with at least three dimensions of social signals.

## 5. Discussion

We have considered the nature of identity signalling in societies with diverse preferences for Co-ordination. Our analysis provides a foundation for a general theory of identity signalling in the context of co-ordinating normative behaviour in a structured population, including the role of multidimensional signalling in affording complex group structures involving nested and overlapping identities with unequal power.

Without social signals, interactions in diverse societies will tend to be suboptimal, with this suboptimality manifesting in at least two ways. First, a society could be characterized by failures of Co-ordination whenever people holding different preferences interact. In this case, because people lack social signals to identify the type of individual they are interacting with, they may simply adopt the action they most prefer. This scenario is most probable when preferences are strongly held or when a society is very diverse and no preference is held by a significant majority. Second, interactions will be suboptimal when either preferences are weakly held or when a significant majority of the population holds the same preferences. In this latter case, a dominant norm will tend to emerge on which people always successfully co-ordinate. However, this successful Co-ordination comes at the cost of some people, typically members of minority groups, adopting an action for Co-ordination that they do not like, even when interacting among themselves. Our analysis replicates prior research suggesting that minorities have low odds of maintaining their culture when frequently interacting with members of a dominate culture (Bunce & McElreath, 2018; O’Connor, 2019). Many prior evolutionary models of ethnic markers focus on their use in partner selection, assuming that individuals always use the same norm once an interaction has begun (Boyd & Richerson, 1987; Bunce and McElreath, 2018; McElreath et al., 2003; O’Connor, 2019). If only individuals could choose their actions based on whether they were interacting with an in-group or out-group member, minorities could co-ordinate on their more preferred action among themselves despite being forced to adopt the majorities preference for between-group interactions.

By adopting social signals, individuals can avoid these suboptimal situations by conditioning their actions on the signals of the person they are interacting with (Section 3.2). Here, our model closely aligns with prior work by Bunce (2021), who found that cross-cultural competence allows minorities to sustain their culture. Strategic use of social signals allow members of a minority group to adopt the majority’s preference as the norm for Co-ordination between groups while retaining their preferred norms when interacting among themselves. Bunce (2020) finds empirical support for this idea in his studies of intra- and inter ethnic interactions in Peru, showing that cross-cultural competence is most prevalent among minorities living among a larger group.

Previous research has indicated that social signals – also called identity signals, tags, or ethnic markers – are particularly useful at borders where different cultures meet (Barth, 1969; Boyd & Richerson, 1987; Johnson et al., 2024; McElreath et al., 2003). While our model does not include a spatial or network component, the random mixing between agent types is reflective of what one might expect at a border between cultures rather than at the centre of a homogeneous group. It may also represent more quotidian interactions in a diverse population under moderate or low amounts of segregation. When there is uniformity across a population, there is no need for social signals. In fact, majority groups often act *as if* there is uniformity rather than diversity in the population by unflinchingly taking their preference to be the norm (Devos & Banaji, 2005; Simoni & Walters, 2001). Our model produces this result, and compliments previous models of ethnic markers by focusing on using signals for selecting behaviours during an interaction that has already begun.

A reality of modern society is that people have complex social identities (Roccas & Brewer, 2002; Smaldino, 2019). Who we are is often determined, at least in part, by *where* we are and whom we are with. There is therefore a significant benefit to the ability to independently signal different aspects of one’s identity, so that one aspect can be detected even if other aspects go ignored or unappreciated, as when an individual belongs to a niche group that is embedded within a larger group. Members of the larger group can optimize their behaviour without having to learn the social signals associated with the embedded group, while members of the smaller group can still co-ordinate optimally when the opportunity arises. We have demonstrated how the ability to signal in multiple dimensions simultaneously creates the conditions for hierarchical or intersectional identity signalling to emerge and achieve optimal Co-ordination. Relatedly, we also demonstrated how considering the ways in which different individuals co-ordinate (or fail to co-ordinate) is just as important as considering the signals they send to determine the structure of identity and power relationships in a population. For example, we show in Section 4.3 that the same patterns of signalling could be associated with individuals that are equally affiliated with each of two groups, or with individuals whose intersectional identities are not captured by the conjunction of their other affiliations. Only by examining their behaviours can the difference between these two signalling systems be parsed. This has implications for empirical researchers interested in identity, particularly those interested in capturing community relationships based solely on the signals used by the constituents of those communities. A focus on signals alone will fail to capture the group structures that include nested or intersectional identities as well as power dynamics.

We believe our model is a good fit for some aspects of Co-ordination among people with intersectional identities. However, it should be noted that intersectionality is typically discussed in terms of disadvantage experienced by marginalized groups. What we have called the intersectional structure requires only that there is some group whose members signal two identities but co-ordinate among themselves on an action that is not preferred by people who are members of only one of the two groups. This could occur among a group of people that does not experience any of the disadvantages traditionally associated with intersectionality. Readers for whom intersectionality does not exist without this power imbalance may wish to restrict use of the term ‘intersectional’ in the context of the model only for those group structures that include similar power imbalances, with ŉon-reductive group structures’ being a more general term that does not imply a particular power relationships.

Our model captures the three-step process of (i) differences in *preferences* (ii) contributing to the evolution of social *signals* such that (iii) people can optimally condition their *actions* based on identifying in-group and out-group members through their social signals. In so doing, we encapsulate more of the complexity of human identity signalling and co-ordinative behaviour than most related modelling work. Nevertheless, there are important limitations that can and should be addressed to produce a more comprehensive theory of identity signalling in diverse populations. First, we do not address how people come to have different preferences in the first place. Preferences are often shaped by our social groups and identity commitments (Cialdini & Goldstein, 2004; Fehr & Hoff, 2011), so an important direction for future research is to consider how a model of optimal signalling and assortment coheres with models of identity-influenced preference change (Steiglechner et al., 2023, 2025), which might additionally include the role of immigration and even long-distance interaction (Kunst & Mesoudi, 2025; Smaldino et al., 2025). In a future model, we hope to include dynamic preferences that evolve concurrently with group signals and individuals’ dispositions to act in particular ways in response to social signals. Second, we limited the context for interactions (and therefore preferences) to Co-ordination, as captured by the generalized BoS game. While Co-ordination is at the heart of much of human social behaviour (Bicchieri, 2005; Calcott, 2008; Eickers, 2023), other contexts are better captured by other games, including games involving more than two players. For example, Bednar and Page (2007) have advocated for the importance of exploring contexts in which defecting from a cooperative strategy is incentivized and we may want to explore contexts in which such defection are conditioned on complex group memberships. Third, we only considered discrete, all-or-none signals. While some signals, such as symbols or words may be discrete, others contain more continuity. For example, consider the strength or consistency of an accent in spoken language (Cohen, 2012). In the United States, a slight drawl might signal someone is from the South, while a stronger accent might additional signal that they are from a particularly rural part of the South. Finally, we only considered overt signals that are unambiguous and equally observable to any interlocutor. However, many signals of identity are encrypted or covert, especially those used by minority groups, oppressed groups, or groups incentivized to maintain secrecy (Smaldino & Turner, 2022; Smaldino et al., 2018). Such covert signals have not yet been considered in the context of complex identity structures with multidimensional signalling. While we have yet to explore these future research directions, we have laid a solid foundation for these and other possible continuations of modelling social identities.

## Supporting information

10.1017/ehs.2026.10037.sm001 Gabriel et al. supplementary material Gabriel et al. supplementary material

## Data Availability

https://github.com/nathanlgabriel/social_identity_signaling.

## Supplementary material

The supplementary material for this article can be found at https://doi.org/10.1017/ehs.2026.10037.

## Research transparency and reproducibility interest

https://github.com/nathanlgabriel/social_identity_signaling.

## References

[ref1] Barth, F. (1969). *Ethnic groups and boundaries: The social organization of culture difference*. Waveland Press.

[ref2] Bednar, J., & Page, S. (2007). Can game(s) theory explain culture?: The emergence of cultural behavior within multiple games. *Rationality and Society*, 19(1), 65–97.

[ref3] Berger, J., & Heath, C. (2008). Who drives divergence? Identity signalling, outgroup dissimilarity, and the abandonment of cultural tastes. *Journal of Personality and Social Psychology*, 95(3), 593. 18729697 10.1037/0022-3514.95.3.593

[ref4] Bergstrom, C. T., & Lachmann, M. (2003). The red king effect: when the slowest runner wins the coevolutionary race. *Proceedings of the National Academy of Sciences*, 100(2), 593–598. 10.1073/pnas.0134966100 PMC141041 12525707

[ref5] Bicchieri, C. (2005). *The Grammar of society: The nature and dynamics of social norms*. Cambridge University Press.

[ref6] Bisin, A., Patacchini, E., Verdier, T., & Zenou, Y. (2011). Formation and persistence of oppositional identities. *European Economic Review*, 55(8), 1046–1071.

[ref7] Bourdieu, P. (1985). The social space and the genesis of groups. *Theory and Society*, 14(6), 723–744.

[ref8] Boyd, R., & Richerson, P. J. (1987). The evolution of ethnic markers. *Cultural Anthropology*, 2(1), 65–79.

[ref9] Bunce, J. A. (2020). Field evidence for two paths to cross-cultural competence: implications for cultural dynamics. *Evolutionary Human Sciences*, 2, 3. 10.1017/ehs.2020.1 PMC10427313 37588369

[ref10] Bunce, J. A. (2021). Cultural diversity in unequal societies sustained through cross-cultural competence and identity valuation. *Humanities and Social Sciences Communications*, 8(238), 1–9.38617731

[ref11] Bunce, J. A., & McElreath, R. (2018). Sustainability of minority culture when inter-ethnic interaction is profitable. *Nature Human Behaviour*, 2, 205–212.

[ref12] Burt, R. S. (1977). Power in a social topology. *Social Science Research*, 6(1), 1–83.

[ref13] Calcott, B. (2008). The *other* cooperation problem: Generating benefit. *Biology & Philosophy*, 23(2), 179–203.

[ref14] Castro, L., & Toro, M. A. (2007). Mutual benefit cooperation and ethnic cultural diversity. *Theoretical Population Biology*, 71(3), 392–399. 17156805 10.1016/j.tpb.2006.10.003

[ref15] Cialdini, R. B., & Goldstein, N. J. (2004). Social influence: Compliance and conformity. *Annual Review of Psychology*, 55(1), 591–621. 10.1146/annurev.psych.55.090902.142015 14744228

[ref16] Cohen, E. (2012). The evolution of tag-based cooperation in humans: The case for accent. *Current Anthropology*, 53(5), 588–616.

[ref17] Combahee River Collective. (1977). The Combahee River Collective statement.

[ref18] Crenshaw, K. (1991). Mapping the margins: Intersectionality, identity politics, and violence against women of color. *Stanford Law Review*, 43(6), 1241–1299.

[ref19] Dekker, P., & Van Rooy, R. (2000). Bi-directional optimality theory: An application of game theory. *Journal of Semantics*, 17(3), 217–242.

[ref20] Delnero, P. (2016). Literature and identity in mesopotamia during the old babylonian period. In K. Ryholt & G. Barjamovic (Eds.), *Problems of canonicity and identity formation in Ancient Egypt and Mesopotamia* (pp. 19–50). Museum Tusculanum Press.

[ref21] Devos, T., & Banaji, M. R. (2005). American = White? *Journal of Personality and Social Psychology*, 88(3), 447–66. 15740439 10.1037/0022-3514.88.3.447

[ref22] Eickers, G. (2023). Coordinating behaviours: Is social interaction scripted? *Journal for the Theory of Social Behaviour*, 53(1), 85–99.

[ref23] Erev, I, & Roth, A. E. (1998). Predicting how people play games: Reinforcement learning in experimental games with unique, mixed strategy equilibria. *The American Economic Review*, 88(4), 848–881.

[ref24] Fehr, E., & Hoff, K. (2011). Tastes, castes and culture: The influence of society on preferences. *The Economic Journal*, 121(556), F396–F412.

[ref25] Goodman, J. R., Caines, A., & Foley, R. A. (2023). Shibboleth: An agent-based model of signalling mimicry. *PLoS ONE*, 18(7). 10.1371/journal.pone.0289333 PMC10389733 37523380

[ref26] Hodder, I. (1982). *Symbols in action: Ethnoarchaeological studies of material culture*. New Studies in Archaeology. Cambridge University Press.

[ref27] Ioannides, Y. M. (2006). Topologies of social interactions. *Economic Theory*, 28(3), 559–584.

[ref28] Johnson, D. G., Mattan, B. D., Flores, N., Lauharatanahirun, N., & Falk, E. B. (2022). Social-cognitive and affective antecedents of code switching and the consequences of linguistic racism for black people and people of color. *Affective Science*, 3(1), 5–13. 36046097 10.1007/s42761-021-00072-8 PMC9382929

[ref29] Johnson, L. M., Bell, A. V., & Di Paolo, M. (2024). Evidence for greater marking along ethnic boundaries. *Human Nature*, 35(3), 307–322. 39432140 10.1007/s12110-024-09479-1

[ref30] Kunst, J. R., & Mesoudi, A. (2025). Decoding the dynamics of cultural change: A cultural evolution approach to the psychology of acculturation. *Personality and Social Psychology Review*, 29(2), 111–144. 39056551 10.1177/10888683241258406 PMC11960022

[ref31] Lin, W., Kang, S., Zhu, J., & Ding, L. (2024). Till we have red faces: Drinking to signal trustworthiness in contemporary china. *Public Choice*, 203, 3–22.

[ref32] Luce, R. D. & Raiffa, H. (1957). *Games and decisions: Introduction and critical survey*. Wiley

[ref33] Macanovic, A., Tsvetkova, M., Przepiorka, W., & Buskens, V. (2024). Signals of belonging: Emergence of signalling norms as facilitators of trust and parochial cooperation. *Philosophical Transactions B*, 379(20230029), 1897. 10.1098/rstb.2023.0029 PMC10799729 38244608

[ref34] McCluney, C. L., Durkee, M. I, Smith, R. E., Robotham, K. J., & Lee, S. S.-L. (2021). To be, or not to be… black: The effects of racial codeswitching on perceived professionalism in the workplace. *Journal of Experimental Social psychology*, 97, 104199.

[ref35] McElreath, R., Boyd, R., & Richerson, P. J. (2003). Shared norms and the evolution of ethnic markers. *Current Anthropology*, 44(1), 122–130.

[ref36] Moffett, M. W. (2013). Human identity and the evolution of societies. *Human Nature*, 68(3), 219–267. 10.1007/s12110-013-9170-3 23813244

[ref37] Neary, P. R. (2012). Competing conventions. *Games and Economic Behavior*, 76(1), 301–328.

[ref38] Nettle, D., & Dunbar, R. I. (1997). Social markers and the evolution of reciprocal exchange. *Current Anthropology*, 38(1), 93–99.

[ref39] O’Connor, C. (2017). The cultural red king effect. *The Journal of Mathematical Sociology*, 41(3), 155–171.

[ref40] O’Connor, C. (2019). *The origins of unfairness: Social categories and cultural evolution*. Oxford University Press.

[ref41] Pelzer, K. (2023). What does an upside-down pineapple mean? The hidden message behind the symbol. *Parade*.

[ref42] Roccas, S., & Brewer, M. B. (2002). Social identity complexity. *Personality and Social Psychology Review*, 6(2), 88–106.

[ref43] Simoni, J. M., & Walters, K. L. (2001). Heterosexual identity and heterosexism. *Journal of Homosexuality*, 41(1), 157–172. 11453516 10.1300/J082v41n01_06

[ref44] Singh, M., & Glowacki, L. (2022). Human social organization during the late pleistocene: Beyond the Nomadic-Egalitarian model. *Evolution and Human Behavior*, 43(5), 418–431.

[ref45] Smaldino, P. E. (2019). Social identity and cooperation in cultural evolution. *Behavioural Processes*, 161, 108–116. 29223462 10.1016/j.beproc.2017.11.015

[ref46] Smaldino, P. E. (2022). Models of identity signalling. *Current Directions in Psychological Science*, 31(3), 231–237.

[ref47] Smaldino, P. E. (2023). *Modeling social behavior: Mathematical and agent-based models of social dynamics and cultural evolution*. Princeton University Press.

[ref48] Smaldino, P. E. (2025). A functionalist account of social identity. In W. Wagner, & C. Moya (Eds.) *Culture, identity, and essentialism in intergroup relations*. Springer-Nature.

[ref49] Smaldino, P. E., Flamson, T. J., & McElreath, R. (2018). The evolution of covert signalling. *Scientific Reports*, 8(4905). 10.1038/s41598-018-22926-1 PMC5861109 29559650

[ref50] Smaldino, P. E., Russell, A., Zefferman, M. R., Donath, J., Foster, J. G., Guilbeault, D., Hilbert, M., Hobson, E. A., Lerman, K., Miton, H., & Moser, C. (2025). Information architectures: A framework for understanding socio-technical systems. *Npj Complexity*, 2(13). 10.1038/s44260-025-00037-z PMC12006018 40255931

[ref51] Smaldino, P. E., & Turner, M. A. (2022). Covert signalling is an adaptive communication strategy in diverse populations. *Psychological Review*, 129(4), 812. 34968133 10.1037/rev0000344

[ref52] Smaldino, P. E., & Velilla, A. P. (2025). The evolution of similarity-biased social learning. *Evolutionary Human Sciences*, 7, e4. 40008386 10.1017/ehs.2024.46 PMC11859121

[ref53] Steiglechner, P., Smaldino, P. E., & Merico, A. (2025). How opinion variation among in-groups can skew perceptions of ideological polarization. *PNAS Nexus* 4(7), pgaf184. 40607106 10.1093/pnasnexus/pgaf184 PMC12218193

[ref54] Steiglechner, P., Smaldino, P. E., Moser, D., & Merico, A. (2023). Social identity bias and communication network clustering interact to shape patterns of opinion dynamics. *Journal of the Royal Society Interface*, 20(209), 20230372. 38086404 10.1098/rsif.2023.0372 PMC10715916

[ref55] van der Does, T., Galesic, M., Dunivin, Z. O., & Smaldino, P. E. (2022). Strategic identity signalling in heterogeneous networks. *Proceedings of the National Academy of Sciences*, 119(10), e2117898119. 10.1073/pnas.2117898119 PMC8915961 35239438

[ref56] Varley, T. F., & Kaminski, P. (2022). Untangling synergistic effects of intersecting social identities with partial information decomposition. *Entropy*, 24(10), 1387. 37420406 10.3390/e24101387 PMC9611752

[ref57] Williams, P. L., & Beer, R. D. (2010). Nonnegative decomposition of multivariate information. *arXiv preprint arXiv:1004.2515*.

