# Supplementary material for: The evolution of identity signals for co-ordination in diverse societies
Source: Evol Hum Sci. 2026 Mar 30;8:e17. doi: 10.1017/ehs.2026.10037 (PMC13184662; doi:10.1017/ehs.2026.10037)
Supplement: Gabriel et al. supplementary material [file S2513843X26100371sup001.zip › S2513843X26100371sup001/SupplementB.pdf]

Supplement B

The evolution of identity signals for coordination in diverse societies

Nathan Gabriel[1], Adrian V. Bell[2], & Paul E. Smaldino[1,3]
[1] University of California, Merced
[2] University of Utah
[3] Santa Fe Institute

This supplemental gives further details corresponding to Supplemental A, Appendix C. Specifically, it shows results for simulations with alpha = 0.4 and beta = 0.04. Immediately below are listed which outcome obtained for each of 1000 simulations. E.g. simulations 5, 23, 68, etc. were all outcome A. It seemed imprudent to make sweeping generalizations about all of the simulations that resulted in a particular outcome. Consequently, the supplement then shows, for the first ten simulations of each outcome, the signaling and behavior dispositions of the agents as they evolved over time. So, for outcome (i) the evolution over time of signaling and acting are shown for simulations 5, 23, 68, 69, 153, 343, 446, 459, 503, 511. While simulations were run for 8x10^4 timesteps, we have selected the range of timesteps most relevant to understanding each simulation. Thus if the graph ends prior to 8x10^4 timesteps, you should assume that whatever trends are shown in the figure are continued for the remainder of the simulation. Across all of the alpha and beta parameters investigated in section 3.7 of the paper, outcomes (xiv-xvii) were exceedingly rare prima facie it does not make sense how these simulations could occur. Thus, after completing the graphs for alpha = 0.4 and beta = 0.04 (none of which are for outcomes (xiv-xvii)), we then identified every occurrence of outcomes (xiv-xvii) for all alpha and beta values investigated and show the graphs over time for how the outcome obtained. For most of these, the graphs make sense once observed. However, starting with outcome (xv) on page 87, there were some simulations where two different strategy profiles amongst a type reinforced each other such that increasing the proportion of agents with one of the strategy profiles decreased its utility. How this can happen is explained on page 89. To understand this sort of outcome you need to calculate that the expected utility of each of the two strategy profiles is equal. While this is explained for the first occurrence of these symbiotic strategy profiles, in subsequent occurrences we merely copy-pasted the values from the spreadsheet in which we made the calculations, but did not copy down the formulas used to calculate the value of each cell in the spreadsheet. If you would like to see these formulas, the spreadsheets are available on request (please contact Nathan Gabriel at nathan.lawrence.gabriel@gmail.com).

outcomes (i) starts on page 4:
[5, 23, 68, 69, 153, 343, 446, 459, 503, 511, 512, 522, 598, 669, 710, 788, 799, 831]

outcomes (ii):
[]

outcomes (iii):
[]

outcomes (iv) starts on page 14:
[0, 4, 12, 19, 22, 39, 42, 58, 63, 85, 91, 100, 102, 109, 113, 126, 129, 136, 143, 144, 146, 150, 164, 175, 177, 186, 187, 191, 196, 198, 201, 208, 222, 237, 243, 247, 254, 265, 271, 275, 284, 287, 288, 292, 294, 296, 310, 314, 316, 325, 338, 350, 352, 356, 365, 373, 381, 389, 394, 397, 401, 407, 427, 430, 437, 438, 452, 453, 457, 458, 472, 480, 481, 484, 487, 490, 498, 505, 517, 524, 527, 535, 539, 543, 546, 547, 551, 552, 555, 556, 558, 559, 567, 586, 587, 596, 610, 614, 615, 617, 621, 622, 629, 630, 633, 639, 647, 649, 654, 658, 666, 670, 682, 692, 693, 698, 702, 704, 706, 714, 715, 730, 736, 742, 744, 751, 759, 772, 782, 784, 791, 792, 793, 796, 812, 816, 817, 825, 837, 846, 847, 857, 869, 876, 883, 911, 918, 941, 945, 947, 964, 974, 975, 979, 980, 986, 987, 993, 998, 999]

outcomes (v) starts on page 24:
[32, 43, 67, 125, 133, 179, 188, 199, 200, 236, 396, 431, 445, 465, 466, 469, 493, 651, 703, 824, 832, 856, 873, 877, 951]

outcomes (vi):
[]

outcomes (vii):
[]

outcomes (viii) starts on page 34:
[1, 2, 3, 7, 8, 11, 13, 14, 16, 18, 20, 24, 27, 28, 29, 31, 33, 34, 36, 37, 41, 44, 45, 46, 48, 51, 52, 54, 56, 57, 59, 61, 62, 64, 65, 70, 71, 72, 73, 77, 80, 81, 83, 86, 88, 89, 93, 94, 95, 98, 99, 101, 103, 104, 105, 106, 108, 110, 111, 112, 115, 118, 119, 120, 121, 122, 123, 124, 127, 130, 131, 135, 139, 140, 141, 142, 147, 148, 149, 155, 158, 161, 162, 165, 166, 168, 169, 171, 172, 173, 174, 178, 181, 182, 183, 184, 185, 190, 192, 193, 195, 197, 202, 203, 205, 209, 211, 212, 215, 216, 217, 219, 220, 224, 225, 228, 229, 230, 232, 233, 234, 238, 240, 241, 244, 245, 246, 248, 249, 250, 252, 255, 256, 258, 260, 261, 262, 263, 264, 267, 268, 270, 272, 274, 278, 280, 281, 283, 285, 286, 289, 290, 291, 293, 295, 299, 300, 301, 302, 303, 305, 306, 307, 308, 309, 311, 315, 318, 319, 320, 321, 322, 324, 326, 327, 328, 329, 330, 332, 334, 335, 339, 341, 342, 344, 345, 346, 347, 348, 349, 351, 353, 354, 357, 360, 361, 362, 363, 367, 368, 370, 372, 374, 376, 377, 378, 379, 380, 382, 383, 384, 388, 390, 391, 398, 400, 403, 404, 405, 406, 408, 409, 411, 414, 415, 416, 417, 419, 422, 424, 425, 436, 440, 441, 444, 447, 448, 449, 450, 451, 454, 456, 462, 463, 464, 467, 468, 470, 471, 473, 474, 482, 483, 486, 488, 489, 491, 495, 497, 499, 500, 502, 506, 507, 510, 513, 514, 515, 516, 518, 519, 520, 525, 528, 529, 530, 531, 532, 533, 534, 536, 538, 540, 544, 545, 549, 553, 554, 561, 563, 564, 566, 568, 570, 571, 573, 574, 575, 578, 579, 580, 582, 583, 584, 585, 590, 592, 595, 597, 601, 604, 605, 606, 607, 608, 611, 612, 613, 618, 619, 620, 625, 626, 631, 632, 634, 635, 636, 638, 642, 645, 646, 648, 652, 653, 655, 659, 660, 661, 662, 663, 664, 665, 667, 673, 674, 676, 677, 678, 680, 683, 686, 687, 688, 689, 690, 694, 700, 705, 707, 709, 711, 712, 716, 718, 720, 721, 725, 726, 728, 729, 732, 733, 734, 735, 738, 739, 743, 745, 747, 748, 750, 752, 753, 754, 755, 756, 757, 760, 761, 762, 763, 764, 766, 768, 769, 770, 773, 775, 776, 778, 779, 780, 783, 785, 786, 787, 795, 797, 798, 800, 801, 802, 803, 804, 805, 807, 808, 809, 810, 813, 814, 818, 819, 820, 821, 822, 823, 826, 828, 829, 830, 833, 834,

835, 838, 840, 842, 844, 845, 848, 850, 853, 854, 858, 859, 860, 861, 863, 865, 866, 867, 868, 870, 871, 874, 875, 878, 879, 880, 881, 882, 884, 885, 887, 889, 891, 892, 893, 894, 895, 897, 901, 902, 904, 906, 908, 909, 910, 915, 916, 917, 919, 921, 922, 926, 927, 928, 932, 935, 937, 938, 943, 944, 946, 948, 949, 953, 955, 956, 957, 959, 960, 961, 962, 965, 966, 967, 968, 971, 972, 973, 977, 978, 981, 988, 990, 994, 995, 996, 997]

outcomes (ix) starts on page 47:
[92, 206, 637]

outcomes (x) starts on page 50:
[9]

outcomes (xi) starts on page 51:
[273, 443, 508, 696, 855, 929, 933]

outcomes (xii) starts on page 58:
[6, 17, 25, 26, 30, 35, 40, 47, 49, 50, 53, 60, 66, 78, 84, 90, 96, 107, 114, 117, 128, 134, 137, 138, 145, 151, 152, 156, 160, 163, 167, 170, 189, 194, 204, 207, 210, 214, 218, 221, 223, 226, 227, 231, 235, 242, 251, 253, 266, 279, 282, 297, 298, 304, 317, 323, 331, 333, 336, 337, 358, 359, 369, 385, 386, 387, 392, 393, 399, 402, 412, 418, 423, 426, 428, 429, 432, 433, 435, 439, 461, 475, 476, 477, 479, 485, 492, 494, 496, 501, 504, 526, 541, 542, 550, 557, 562, 565, 569, 576, 581, 588, 589, 591, 594, 599, 603, 609, 616, 624, 627, 641, 643, 657, 671, 672, 675, 679, 681, 695, 697, 699, 708, 717, 719, 722, 723, 727, 731, 737, 740, 746, 781, 790, 794, 806, 811, 815, 827, 841, 843, 851, 852, 862, 864, 872, 886, 890, 898, 899, 900, 912, 914, 920, 925, 931, 934, 936, 940, 950, 954, 958, 963, 982, 983, 989, 991]

outcomes (xiii) starts on page 68:
[10, 15, 21, 38, 55, 74, 75, 76, 79, 82, 87, 97, 116, 132, 154, 157, 159, 176, 180, 213, 239, 257, 259, 269, 276, 277, 312, 313, 340, 355, 364, 366, 371, 375, 395, 410, 413, 420, 421, 434, 442, 455, 460, 478, 509, 521, 523, 537, 548, 560, 572, 577, 593, 600, 602, 623, 628, 640, 644, 650, 656, 668, 684, 685, 691, 701, 713, 724, 741, 749, 758, 765, 767, 771, 774, 777, 789, 836, 839, 849, 888, 896, 903, 905, 907, 913, 923, 924, 930, 939, 942, 952, 969, 970, 976, 984, 985, 992]

outcomes (xiv-xvii):
[]

outcomes (i):

Run # 5

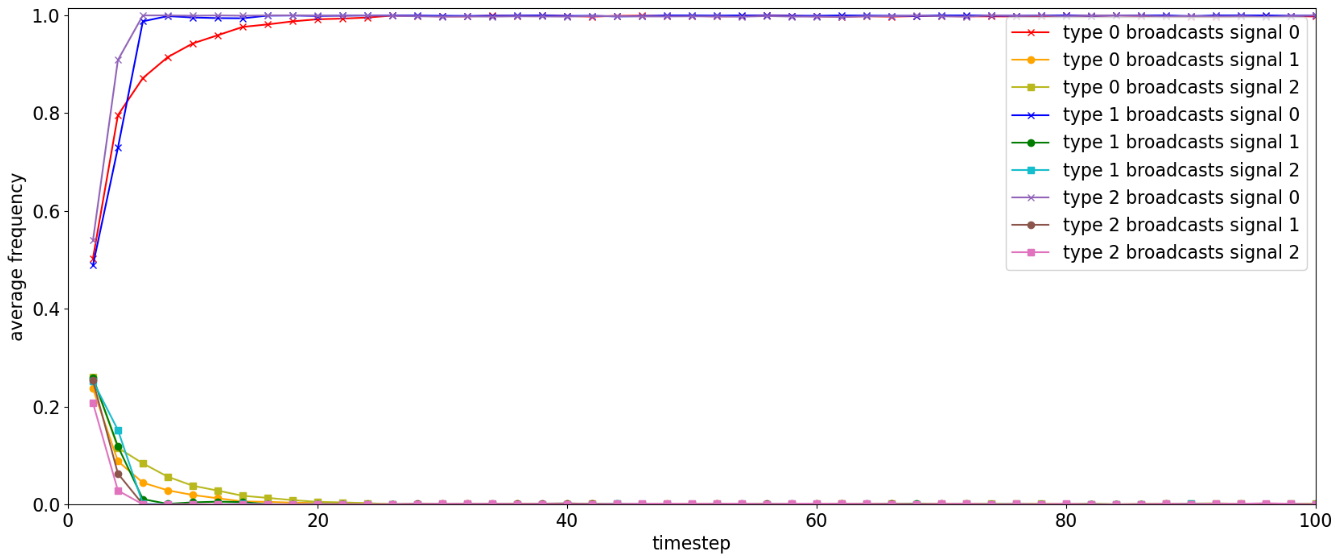

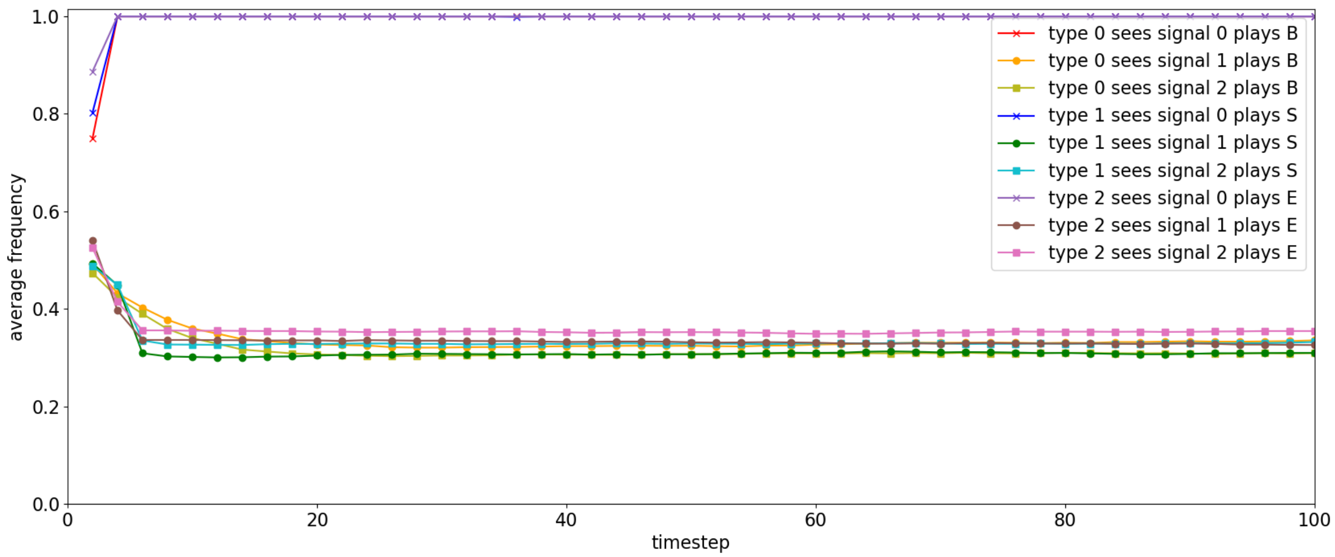

Run # 23

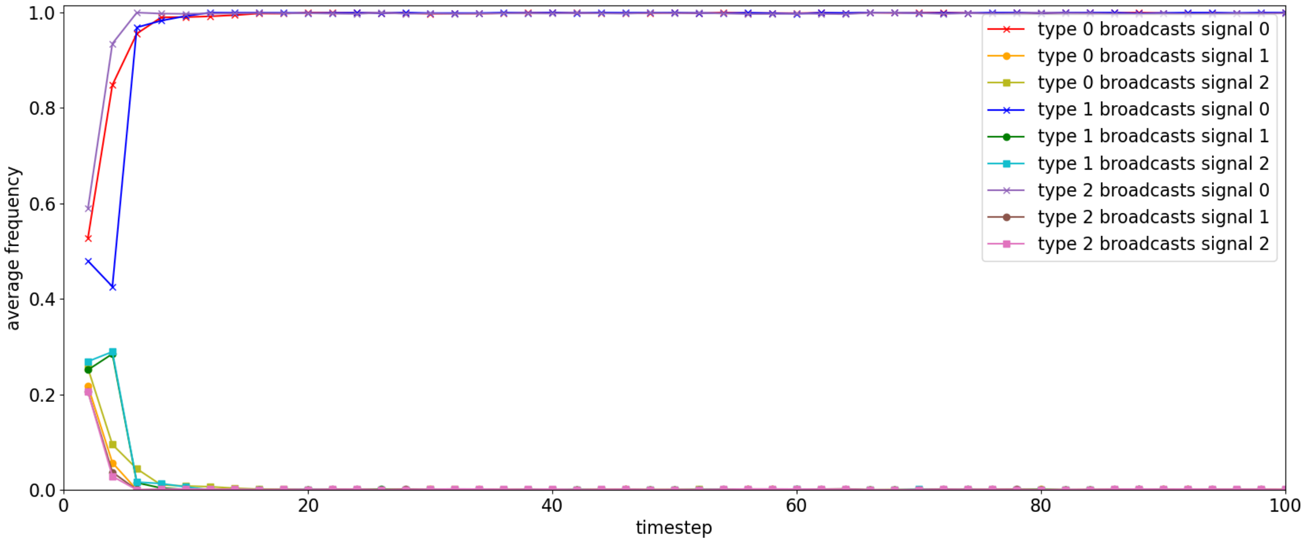

average frequency
timestep
type 0 broadcasts signal 0
type 0 broadcasts signal 1
type 0 broadcasts signal 2
type 1 broadcasts signal 0
type 1 broadcasts signal 1
type 1 broadcasts signal 2
type 2 broadcasts signal 0
type 2 broadcasts signal 1
type 2 broadcasts signal 2

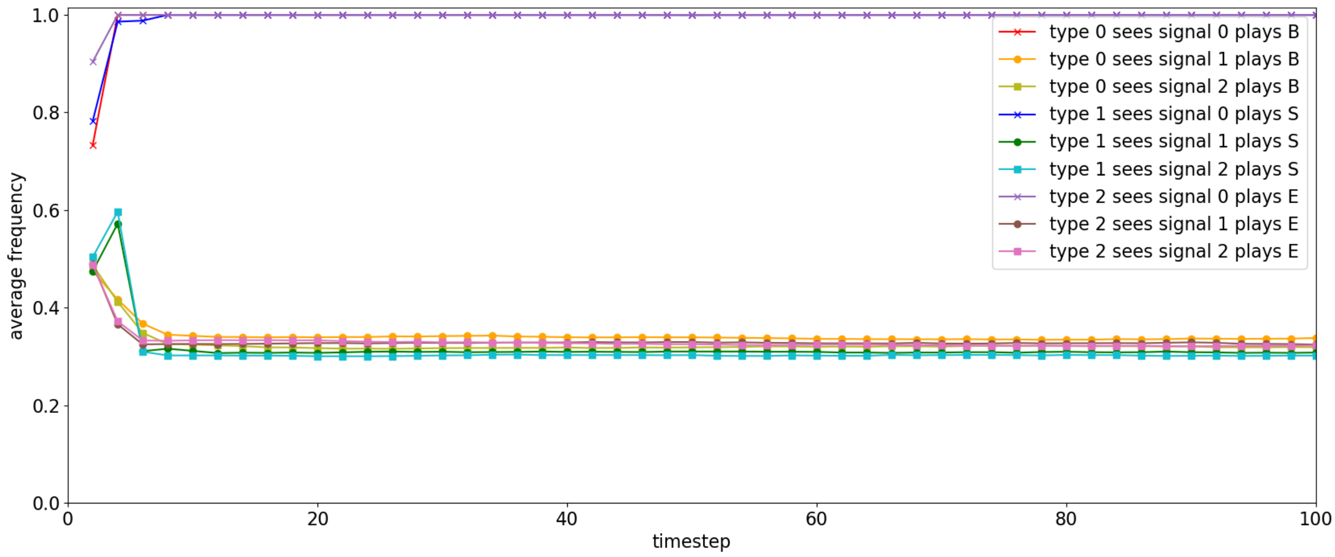

average frequency
timestep
type 0 sees signal 0 plays B
type 0 sees signal 1 plays B
type 0 sees signal 2 plays B
type 1 sees signal 0 plays S
type 1 sees signal 1 plays S
type 1 sees signal 2 plays S
type 2 sees signal 0 plays E
type 2 sees signal 1 plays E
type 2 sees signal 2 plays E

Run # 68

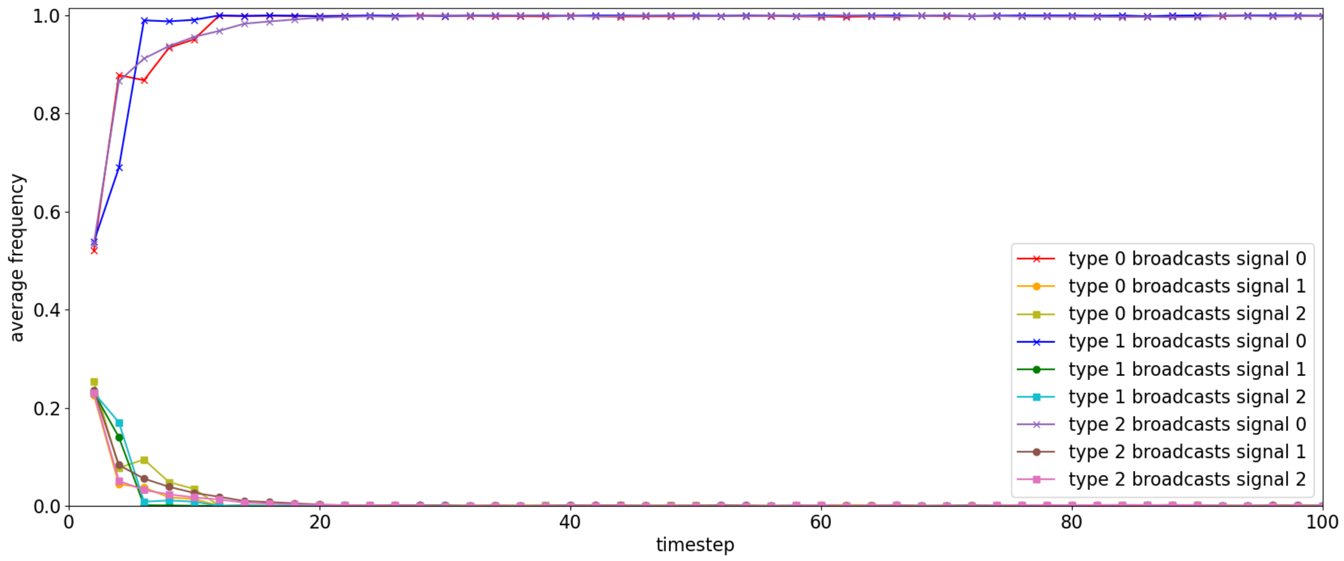
average frequency
timestep
type 0 broadcasts signal 0
type 0 broadcasts signal 1
type 0 broadcasts signal 2
type 1 broadcasts signal 0
type 1 broadcasts signal 1
type 1 broadcasts signal 2
type 2 broadcasts signal 0
type 2 broadcasts signal 1
type 2 broadcasts signal 2

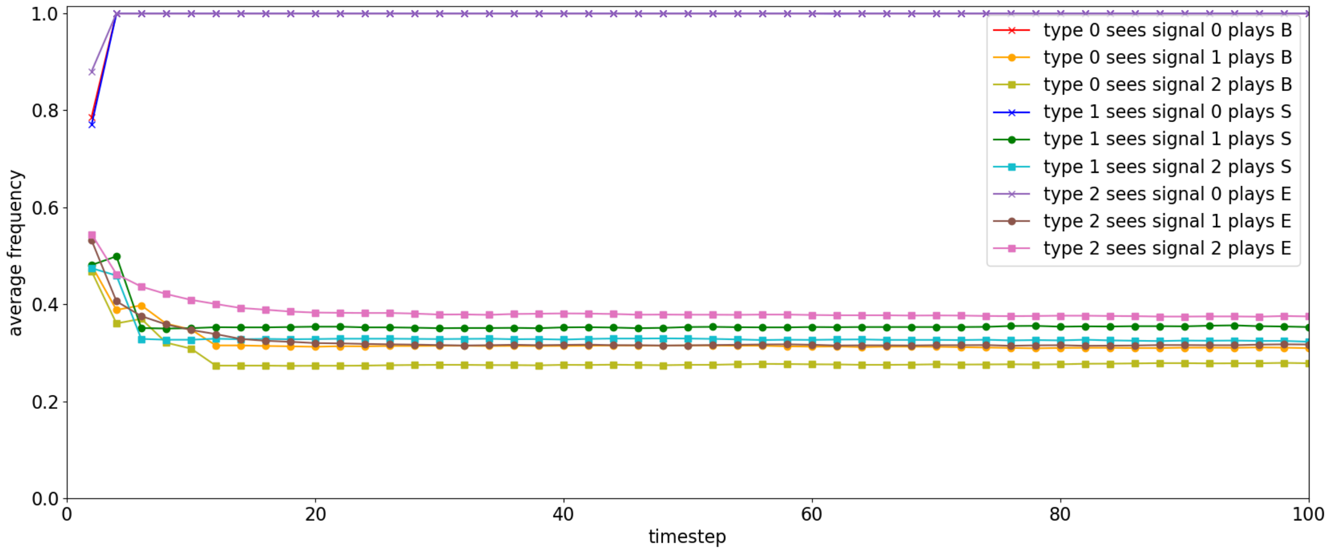
average frequency
timestep
type 0 sees signal 0 plays B
type 0 sees signal 1 plays B
type 0 sees signal 2 plays B
type 1 sees signal 0 plays S
type 1 sees signal 1 plays S
type 1 sees signal 2 plays S
type 2 sees signal 0 plays E
type 2 sees signal 1 plays E
type 2 sees signal 2 plays E

Run # 69

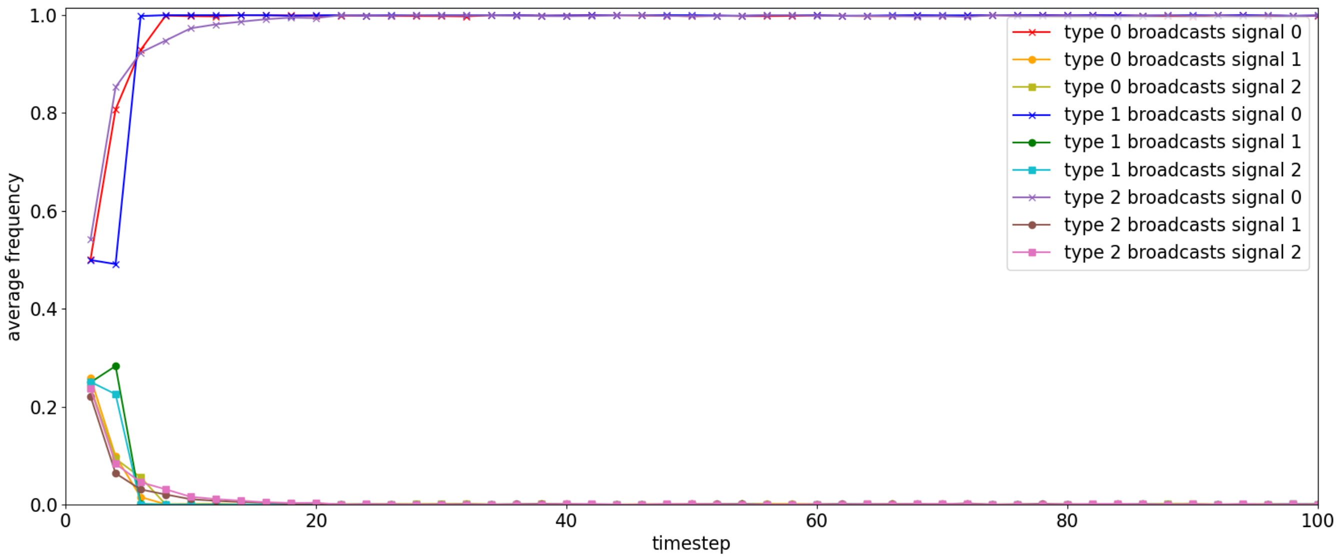
average frequency
timestep
type 0 broadcasts signal 0
type 0 broadcasts signal 1
type 0 broadcasts signal 2
type 1 broadcasts signal 0
type 1 broadcasts signal 1
type 1 broadcasts signal 2
type 2 broadcasts signal 0
type 2 broadcasts signal 1
type 2 broadcasts signal 2

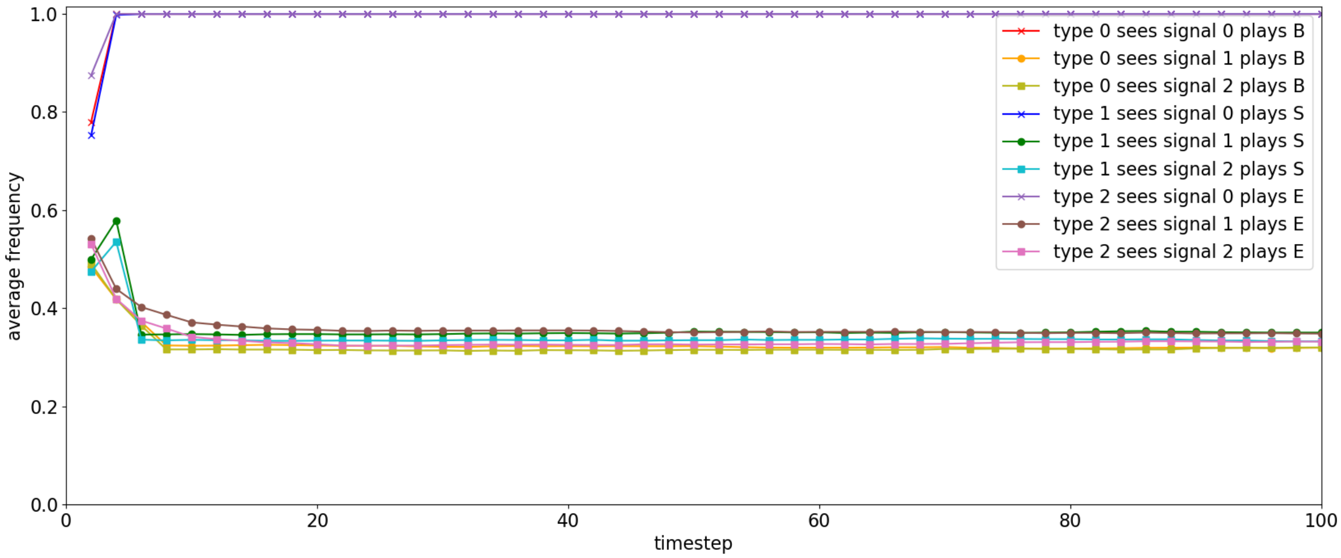
average frequency
timestep
type 0 sees signal 0 plays B
type 0 sees signal 1 plays B
type 0 sees signal 2 plays B
type 1 sees signal 0 plays S
type 1 sees signal 1 plays S
type 1 sees signal 2 plays S
type 2 sees signal 0 plays E
type 2 sees signal 1 plays E
type 2 sees signal 2 plays E

Run # 153

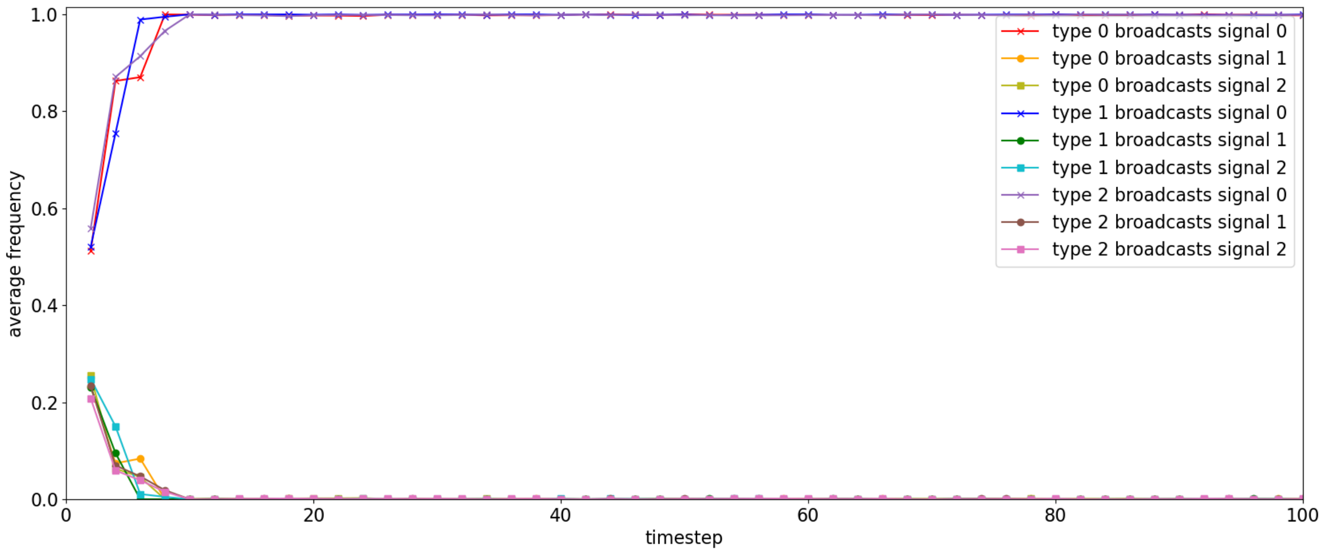
1.0
0.8
0.6
0.4
0.2
0.0
average frequency
0
20
40
60
80
100
timestep
type 0 broadcasts signal 0
type 0 broadcasts signal 1
type 0 broadcasts signal 2
type 1 broadcasts signal 0
type 1 broadcasts signal 1
type 1 broadcasts signal 2
type 2 broadcasts signal 0
type 2 broadcasts signal 1
type 2 broadcasts signal 2

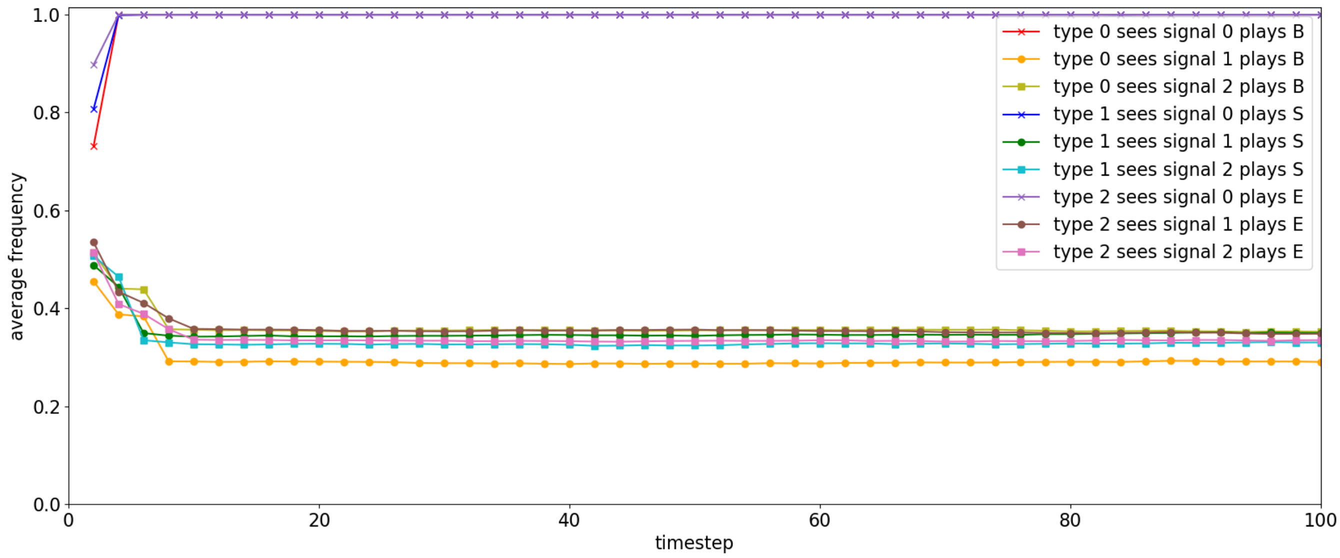
1.0
0.8
0.6
0.4
0.2
0.0
average frequency
0
20
40
60
80
100
timestep
type 0 sees signal 0 plays B
type 0 sees signal 1 plays B
type 0 sees signal 2 plays B
type 1 sees signal 0 plays S
type 1 sees signal 1 plays S
type 1 sees signal 2 plays S
type 2 sees signal 0 plays E
type 2 sees signal 1 plays E
type 2 sees signal 2 plays E

Run # 343

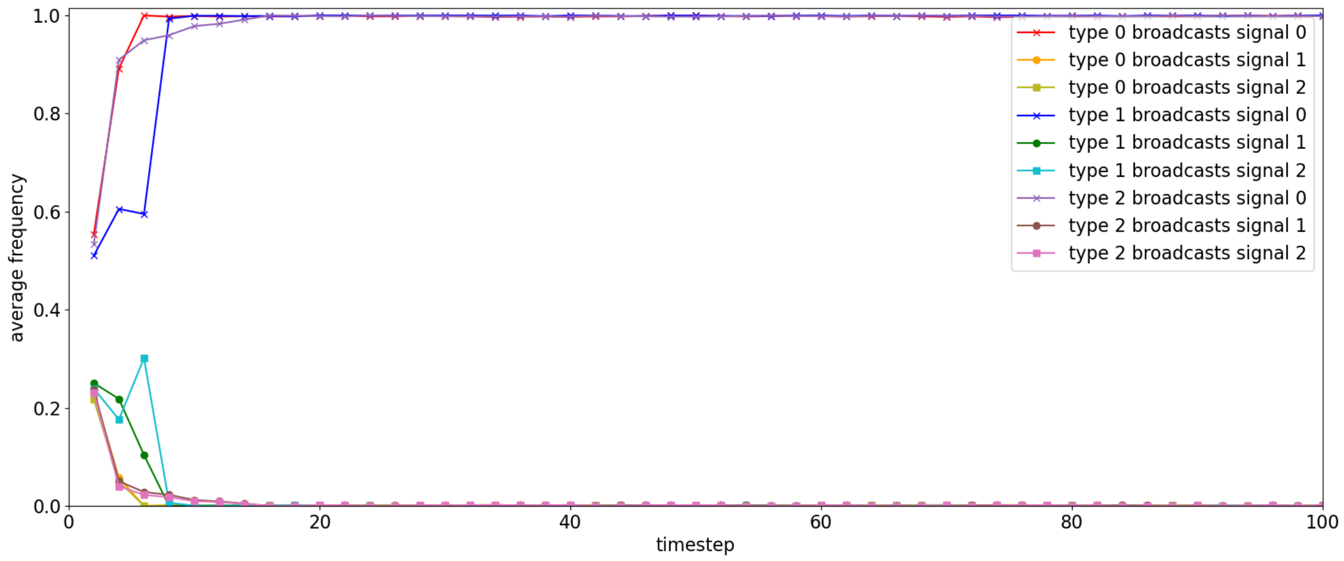
type 0 broadcasts signal 0
type 0 broadcasts signal 1
type 0 broadcasts signal 2
type 1 broadcasts signal 0
type 1 broadcasts signal 1
type 1 broadcasts signal 2
type 2 broadcasts signal 0
type 2 broadcasts signal 1
type 2 broadcasts signal 2
average frequency
timestep

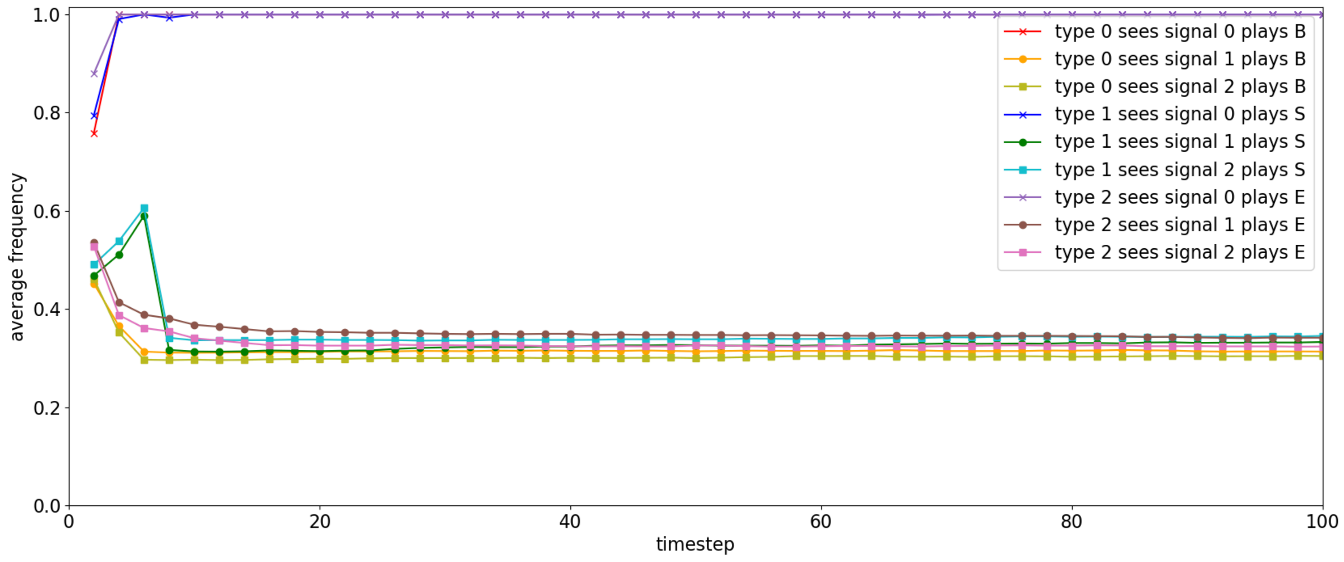
type 0 sees signal 0 plays B
type 0 sees signal 1 plays B
type 0 sees signal 2 plays B
type 1 sees signal 0 plays S
type 1 sees signal 1 plays S
type 1 sees signal 2 plays S
type 2 sees signal 0 plays E
type 2 sees signal 1 plays E
type 2 sees signal 2 plays E
average frequency
timestep

Run # 446

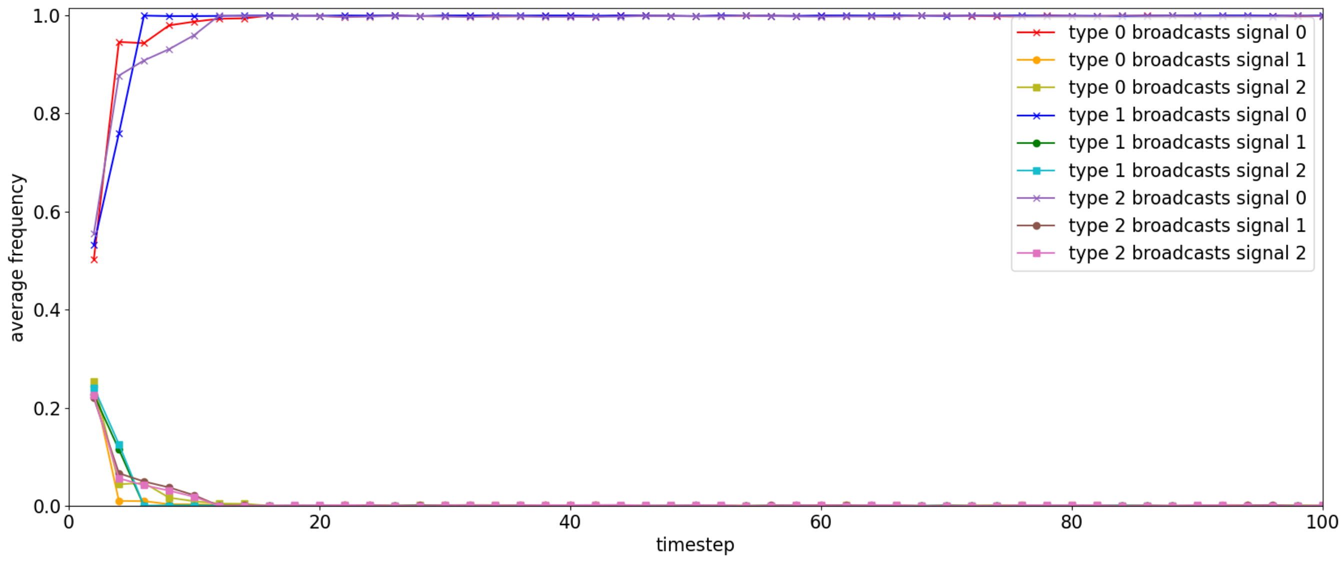
1.0
0.8
0.6
0.4
0.2
0.0
average frequency
0
20
40
60
80
100
timestep
type 0 broadcasts signal 0
type 0 broadcasts signal 1
type 0 broadcasts signal 2
type 1 broadcasts signal 0
type 1 broadcasts signal 1
type 1 broadcasts signal 2
type 2 broadcasts signal 0
type 2 broadcasts signal 1
type 2 broadcasts signal 2

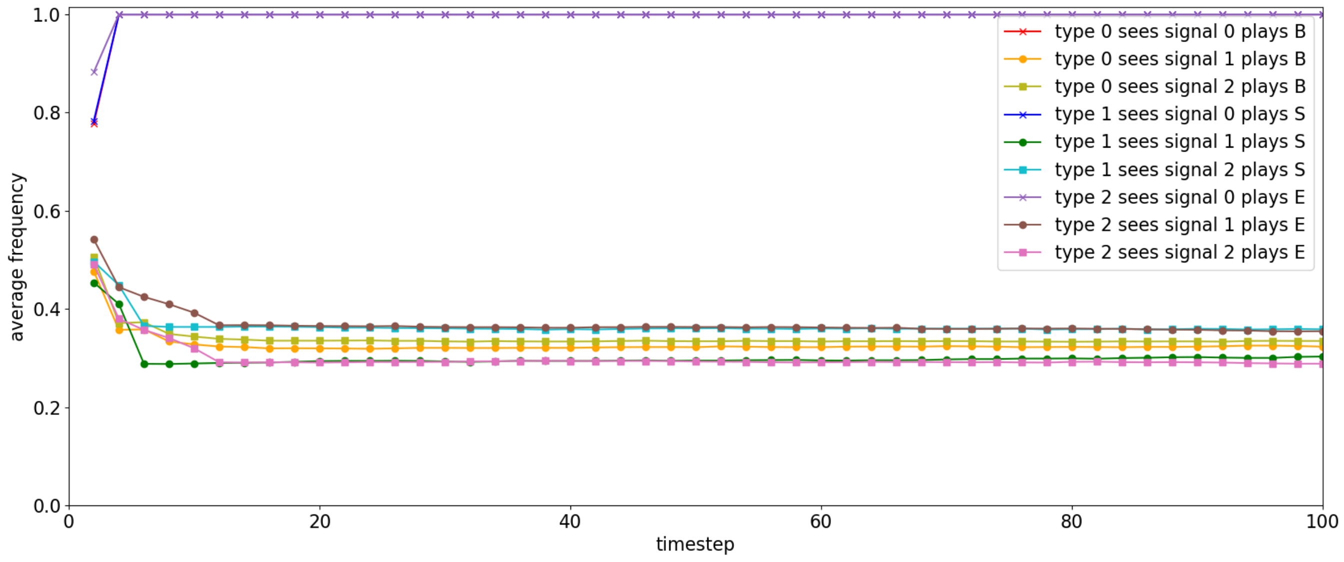
1.0
0.8
0.6
0.4
0.2
0.0
average frequency
0
20
40
60
80
100
timestep
type 0 sees signal 0 plays B
type 0 sees signal 1 plays B
type 0 sees signal 2 plays B
type 1 sees signal 0 plays S
type 1 sees signal 1 plays S
type 1 sees signal 2 plays S
type 2 sees signal 0 plays E
type 2 sees signal 1 plays E
type 2 sees signal 2 plays E

Run # 459

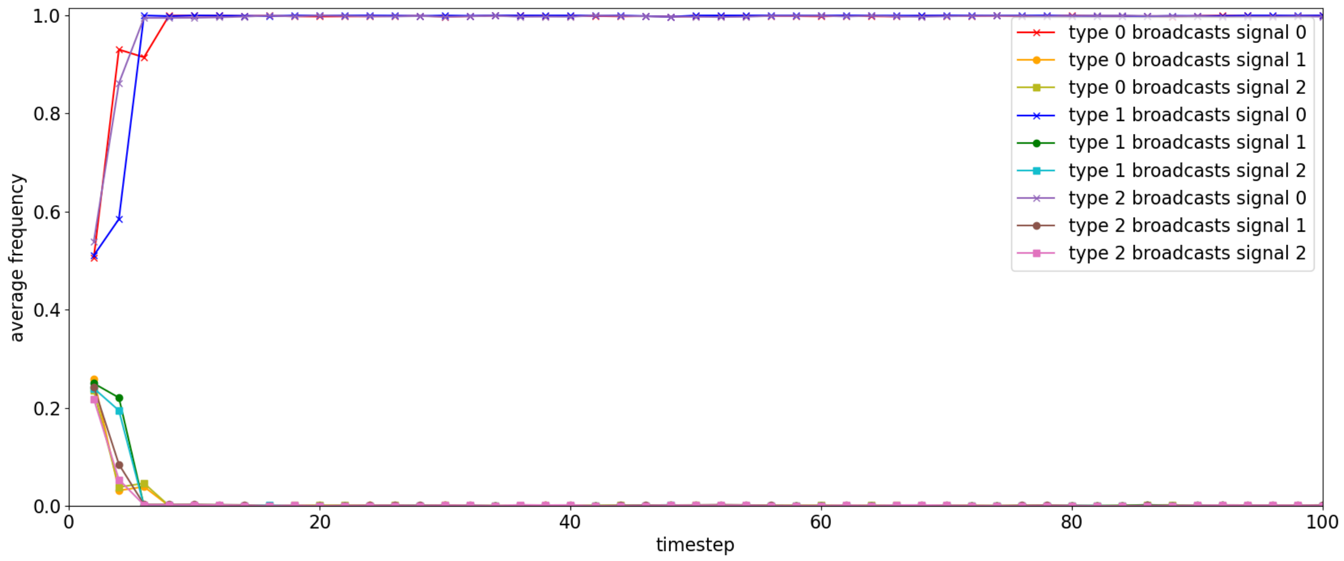

average frequency
timestep
type 0 broadcasts signal 0
type 0 broadcasts signal 1
type 0 broadcasts signal 2
type 1 broadcasts signal 0
type 1 broadcasts signal 1
type 1 broadcasts signal 2
type 2 broadcasts signal 0
type 2 broadcasts signal 1
type 2 broadcasts signal 2

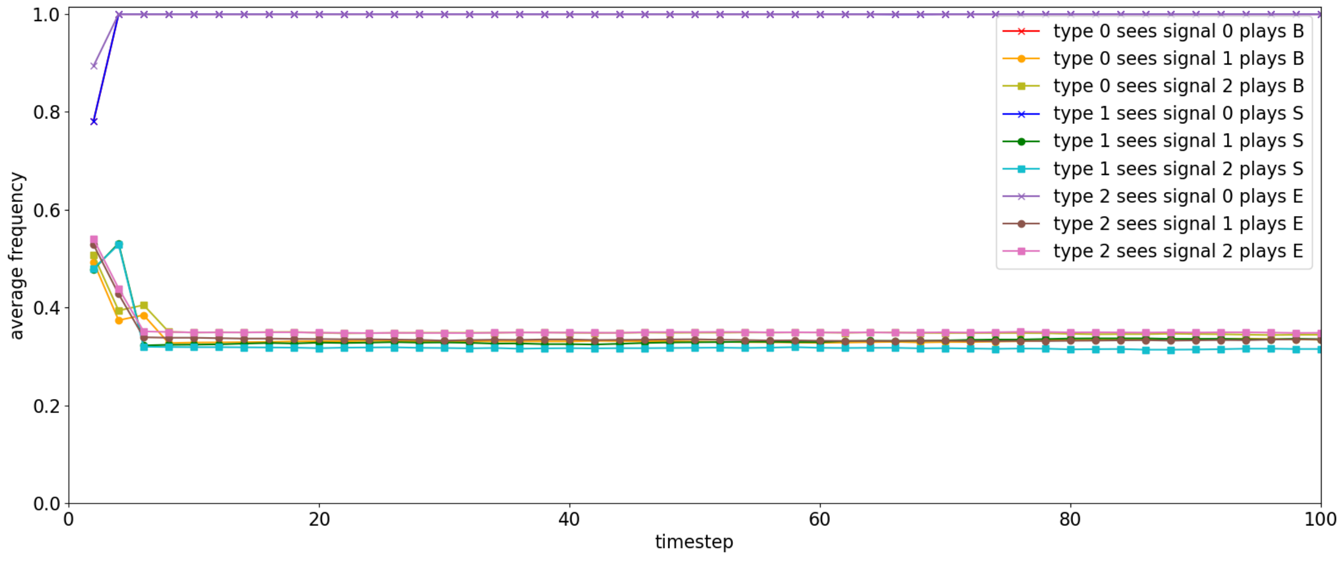

average frequency
timestep
type 0 sees signal 0 plays B
type 0 sees signal 1 plays B
type 0 sees signal 2 plays B
type 1 sees signal 0 plays S
type 1 sees signal 1 plays S
type 1 sees signal 2 plays S
type 2 sees signal 0 plays E
type 2 sees signal 1 plays E
type 2 sees signal 2 plays E

Run # 503

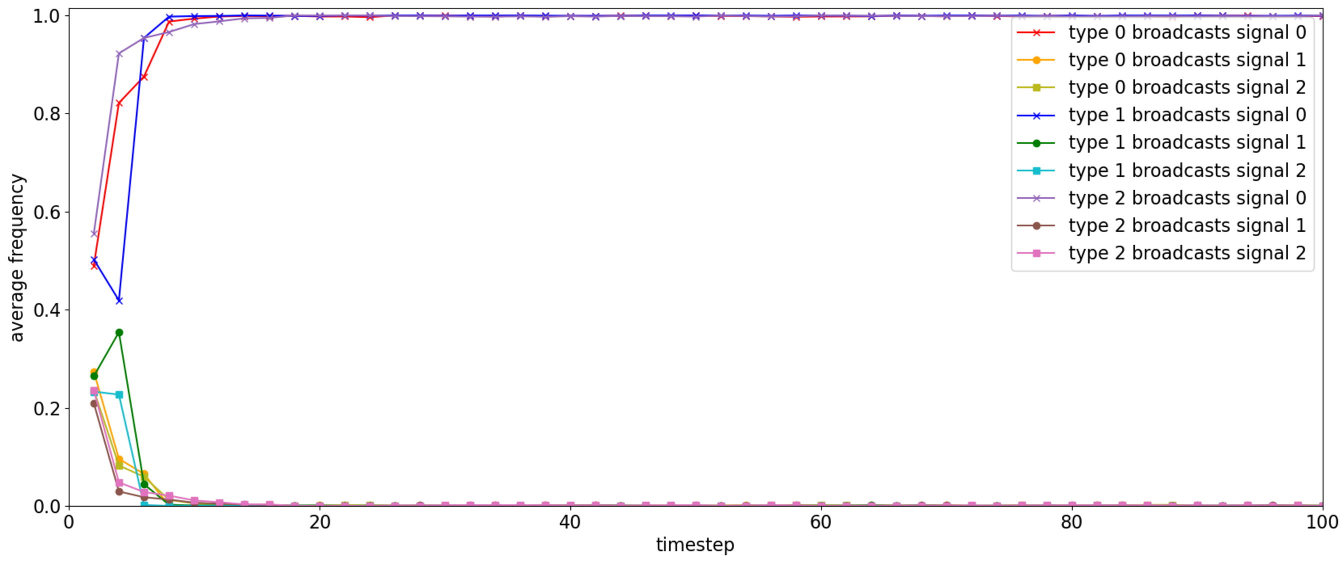
average frequency
timestep
type 0 broadcasts signal 0
type 0 broadcasts signal 1
type 0 broadcasts signal 2
type 1 broadcasts signal 0
type 1 broadcasts signal 1
type 1 broadcasts signal 2
type 2 broadcasts signal 0
type 2 broadcasts signal 1
type 2 broadcasts signal 2

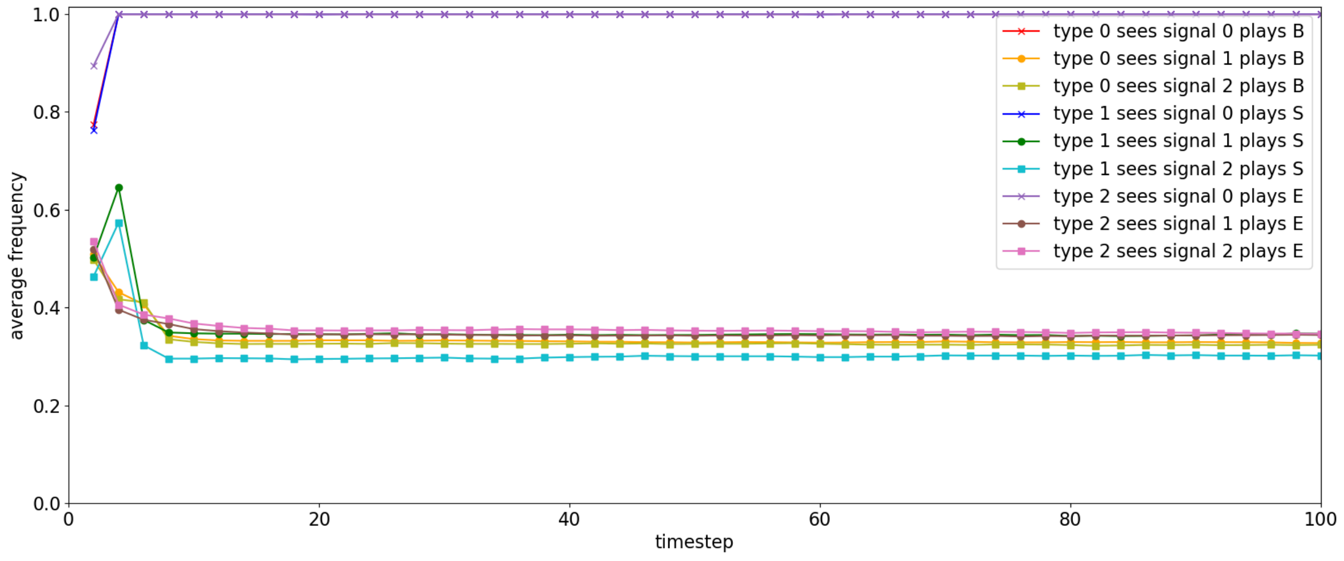
average frequency
timestep
type 0 sees signal 0 plays B
type 0 sees signal 1 plays B
type 0 sees signal 2 plays B
type 1 sees signal 0 plays S
type 1 sees signal 1 plays S
type 1 sees signal 2 plays S
type 2 sees signal 0 plays E
type 2 sees signal 1 plays E
type 2 sees signal 2 plays E

Run # 511

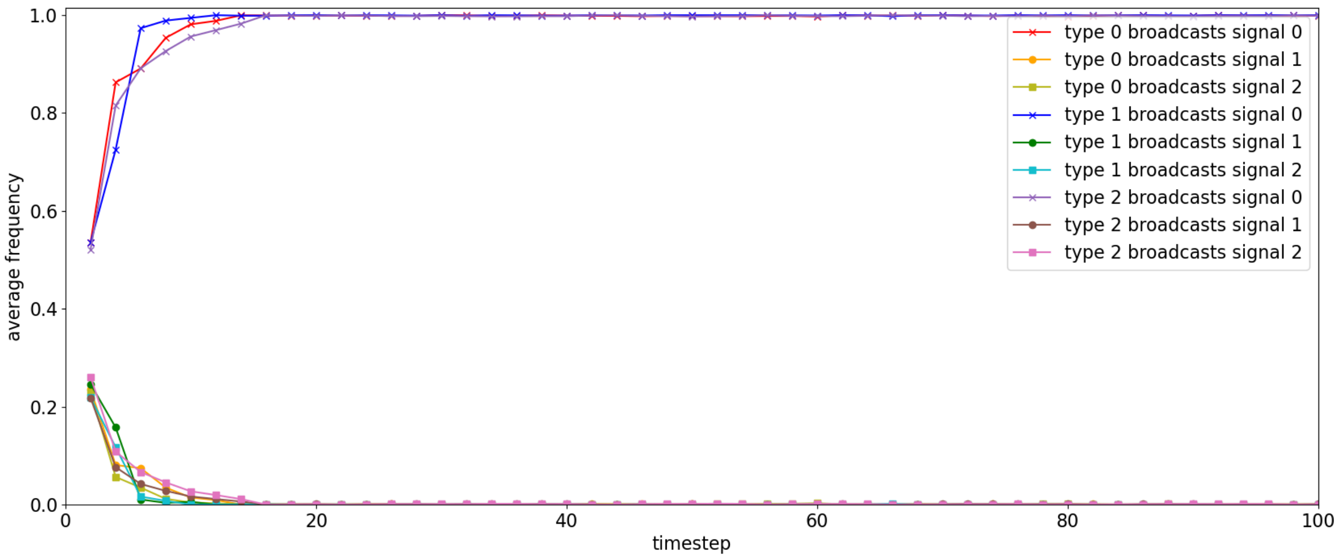

average frequency
timestep
type 0 broadcasts signal 0
type 0 broadcasts signal 1
type 0 broadcasts signal 2
type 1 broadcasts signal 0
type 1 broadcasts signal 1
type 1 broadcasts signal 2
type 2 broadcasts signal 0
type 2 broadcasts signal 1
type 2 broadcasts signal 2

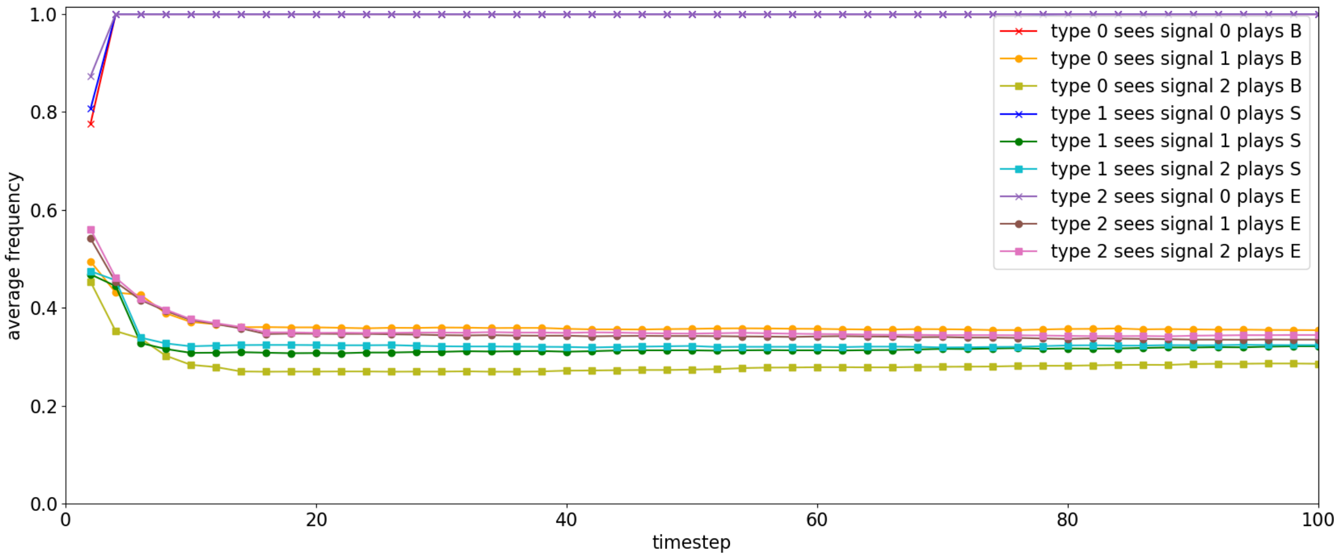

average frequency
timestep
type 0 sees signal 0 plays B
type 0 sees signal 1 plays B
type 0 sees signal 2 plays B
type 1 sees signal 0 plays S
type 1 sees signal 1 plays S
type 1 sees signal 2 plays S
type 2 sees signal 0 plays E
type 2 sees signal 1 plays E
type 2 sees signal 2 plays E

outcomes (iv): generally type 2 signaling 0 and types 0 and 1 sending same signal

Run # 0

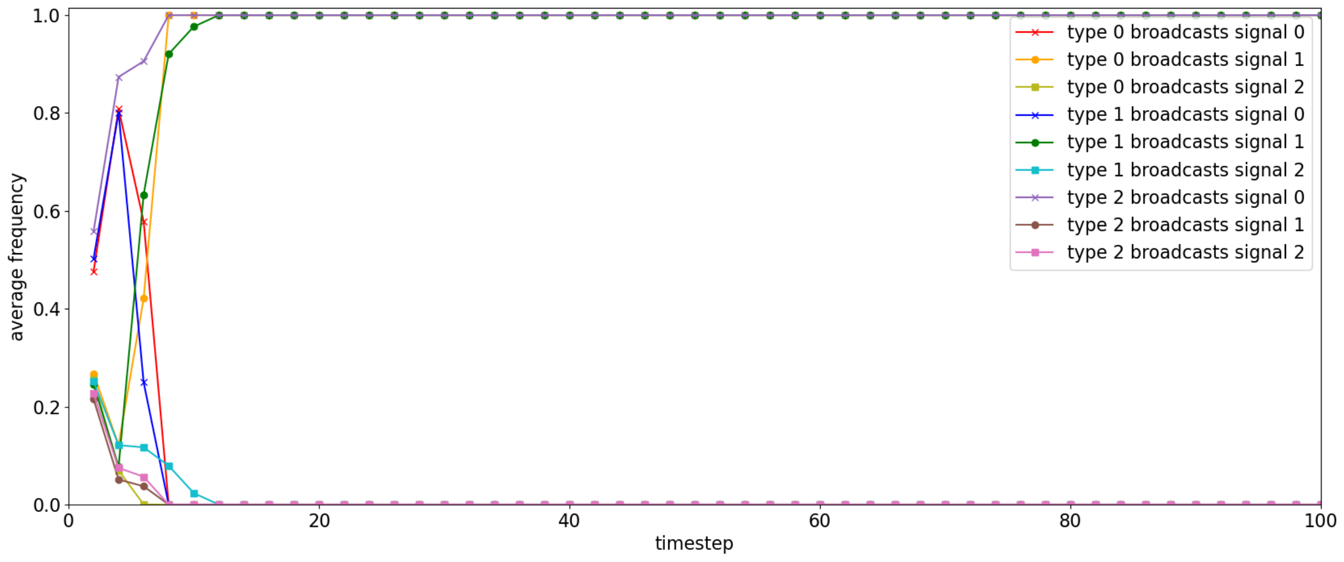

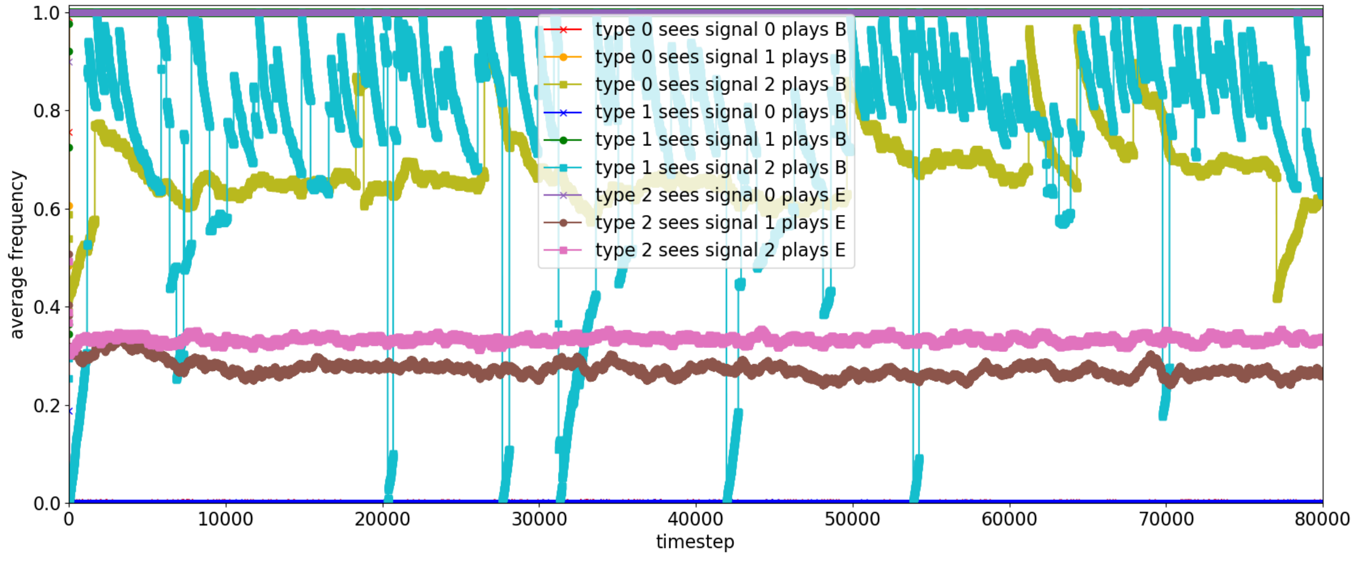

Run # 4

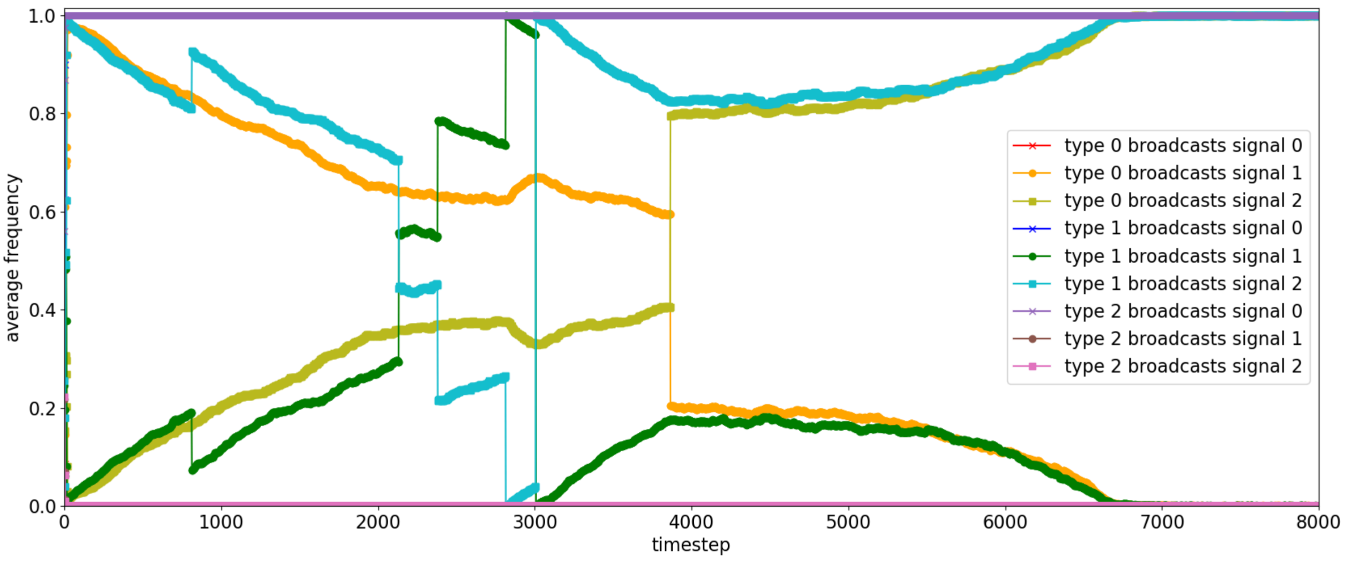

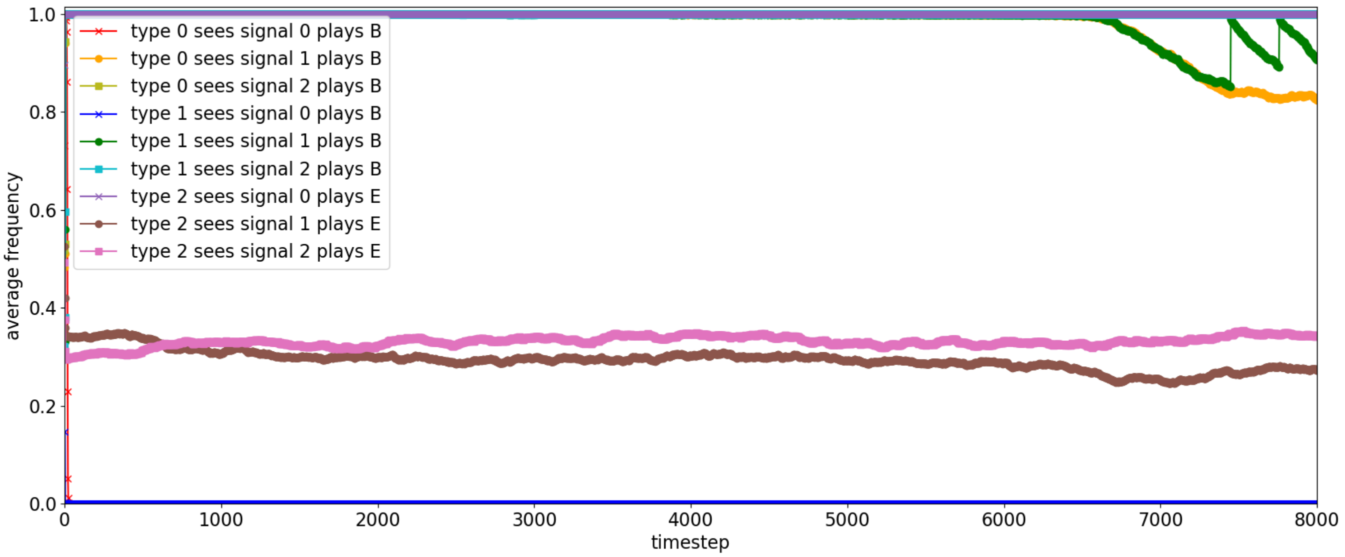

Continued drift in dispositions towards signals, but no further changes to signaling dispositions

Run # 12

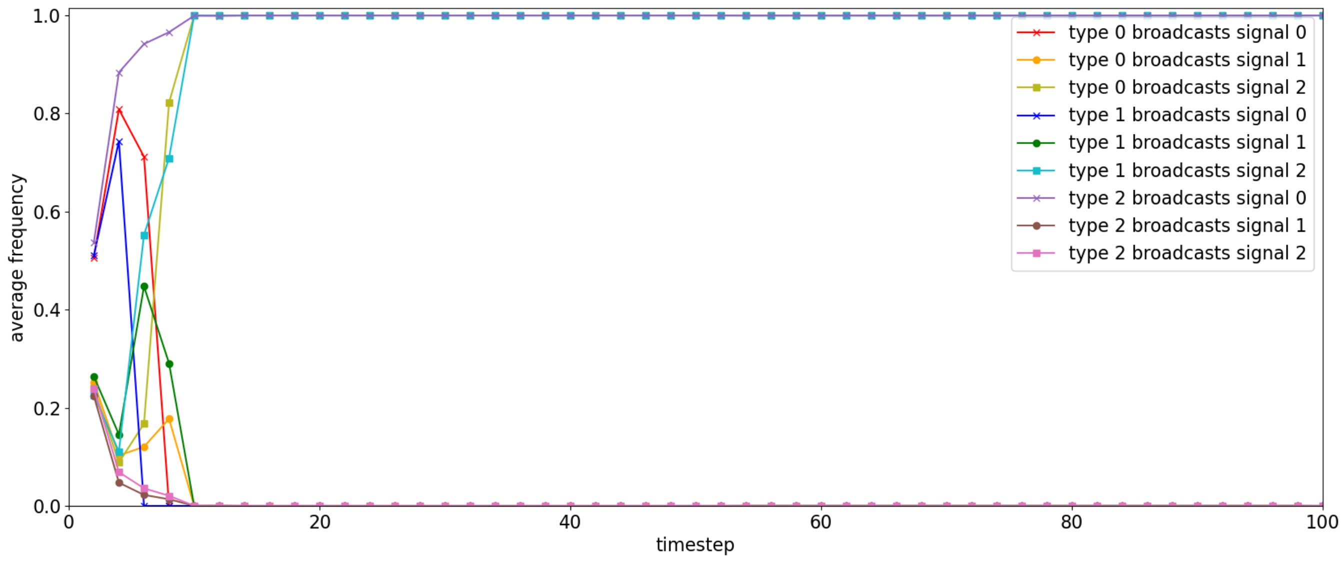
average frequency
timestep
1.0
0.8
0.6
0.4
0.2
0.0
0
20
40
60
80
100
type 0 broadcasts signal 0
type 0 broadcasts signal 1
type 0 broadcasts signal 2
type 1 broadcasts signal 0
type 1 broadcasts signal 1
type 1 broadcasts signal 2
type 2 broadcasts signal 0
type 2 broadcasts signal 1
type 2 broadcasts signal 2

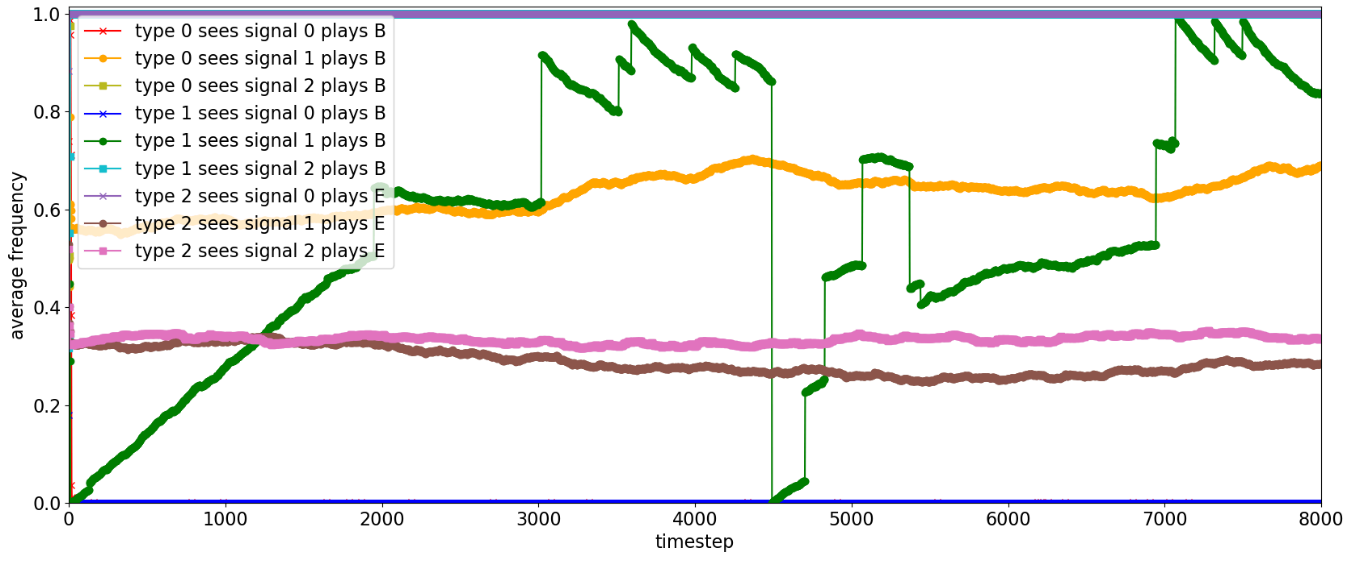
average frequency
timestep
1.0
0.8
0.6
0.4
0.2
0.0
0
1000
2000
3000
4000
5000
6000
7000
8000
type 0 sees signal 0 plays B
type 0 sees signal 1 plays B
type 0 sees signal 2 plays B
type 1 sees signal 0 plays B
type 1 sees signal 1 plays B
type 1 sees signal 2 plays B
type 2 sees signal 0 plays E
type 2 sees signal 1 plays E
type 2 sees signal 2 plays E

Run # 19

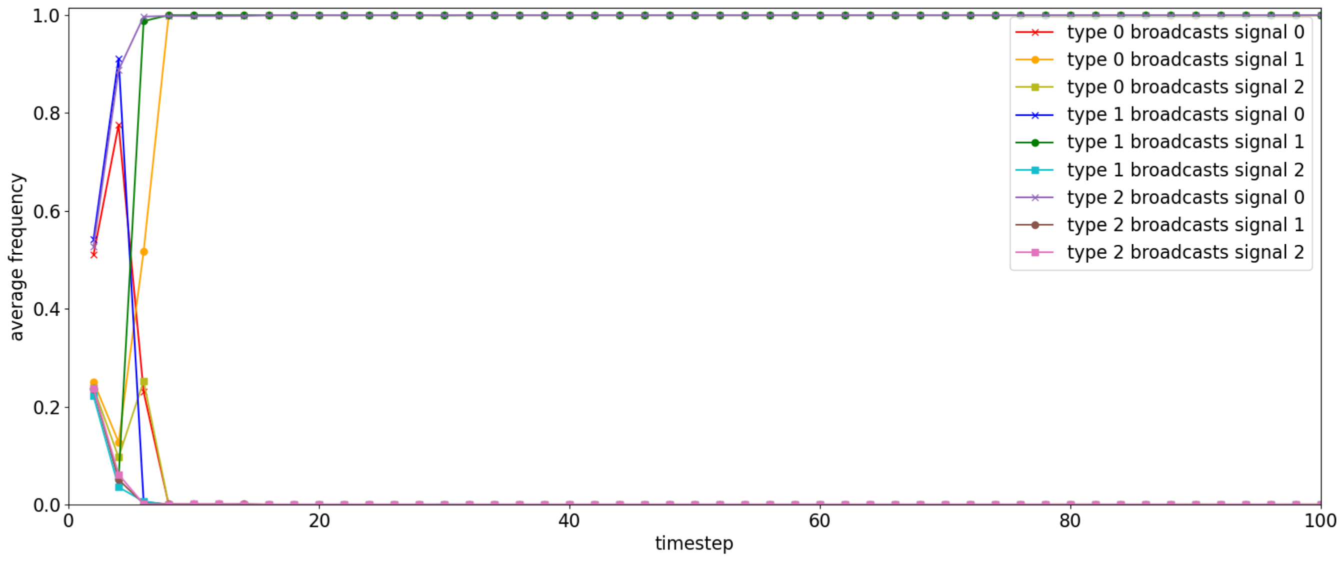

average frequency
timestep
type 0 broadcasts signal 0
type 0 broadcasts signal 1
type 0 broadcasts signal 2
type 1 broadcasts signal 0
type 1 broadcasts signal 1
type 1 broadcasts signal 2
type 2 broadcasts signal 0
type 2 broadcasts signal 1
type 2 broadcasts signal 2

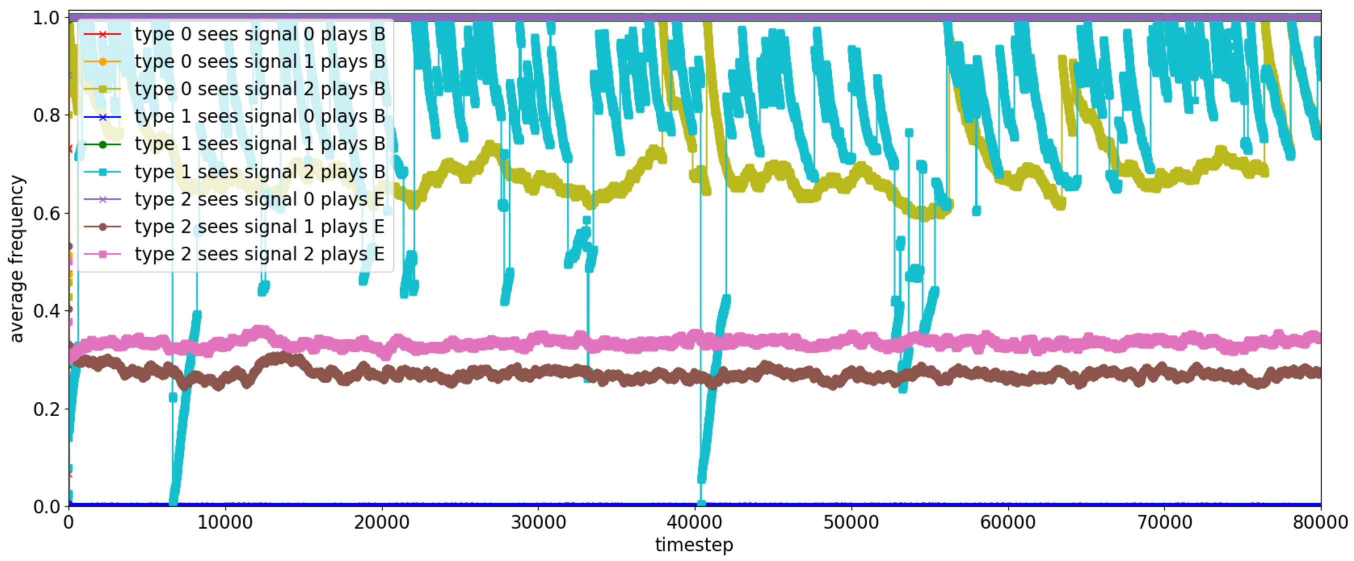

average frequency
timestep
type 0 sees signal 0 plays B
type 0 sees signal 1 plays B
type 0 sees signal 2 plays B
type 1 sees signal 0 plays B
type 1 sees signal 1 plays B
type 1 sees signal 2 plays B
type 2 sees signal 0 plays E
type 2 sees signal 1 plays E
type 2 sees signal 2 plays E

Run # 22

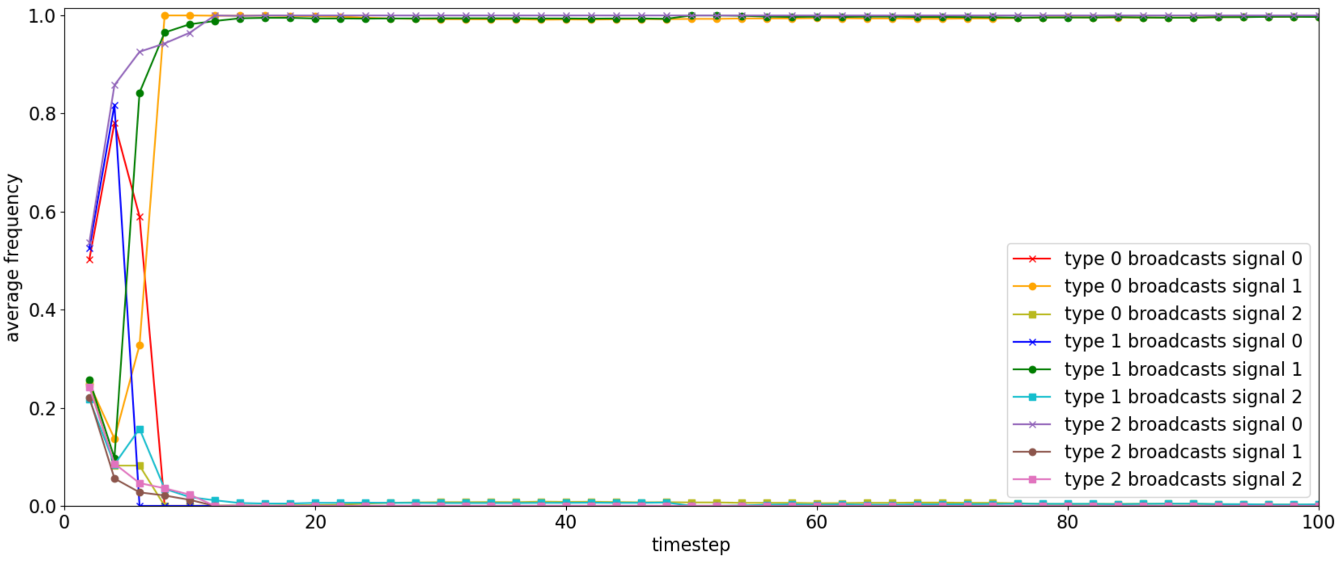

average frequency
timestep
type 0 broadcasts signal 0
type 0 broadcasts signal 1
type 0 broadcasts signal 2
type 1 broadcasts signal 0
type 1 broadcasts signal 1
type 1 broadcasts signal 2
type 2 broadcasts signal 0
type 2 broadcasts signal 1
type 2 broadcasts signal 2

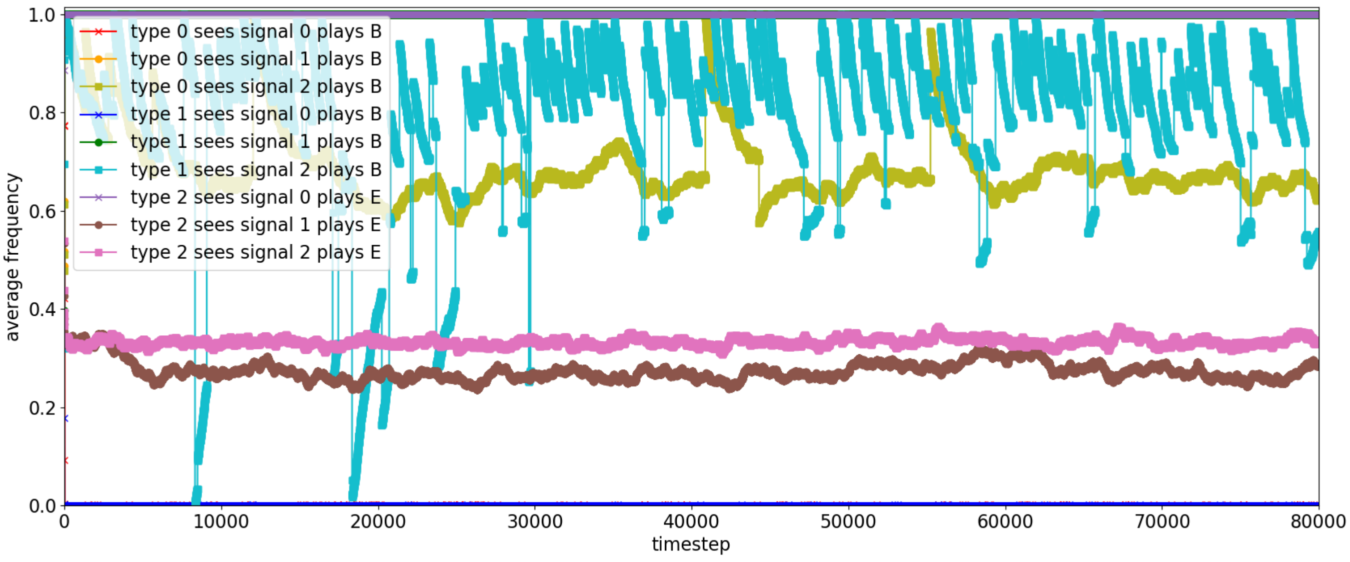

average frequency
timestep
type 0 sees signal 0 plays B
type 0 sees signal 1 plays B
type 0 sees signal 2 plays B
type 1 sees signal 0 plays B
type 1 sees signal 1 plays B
type 1 sees signal 2 plays B
type 2 sees signal 0 plays E
type 2 sees signal 1 plays E
type 2 sees signal 2 plays E

Run # 39

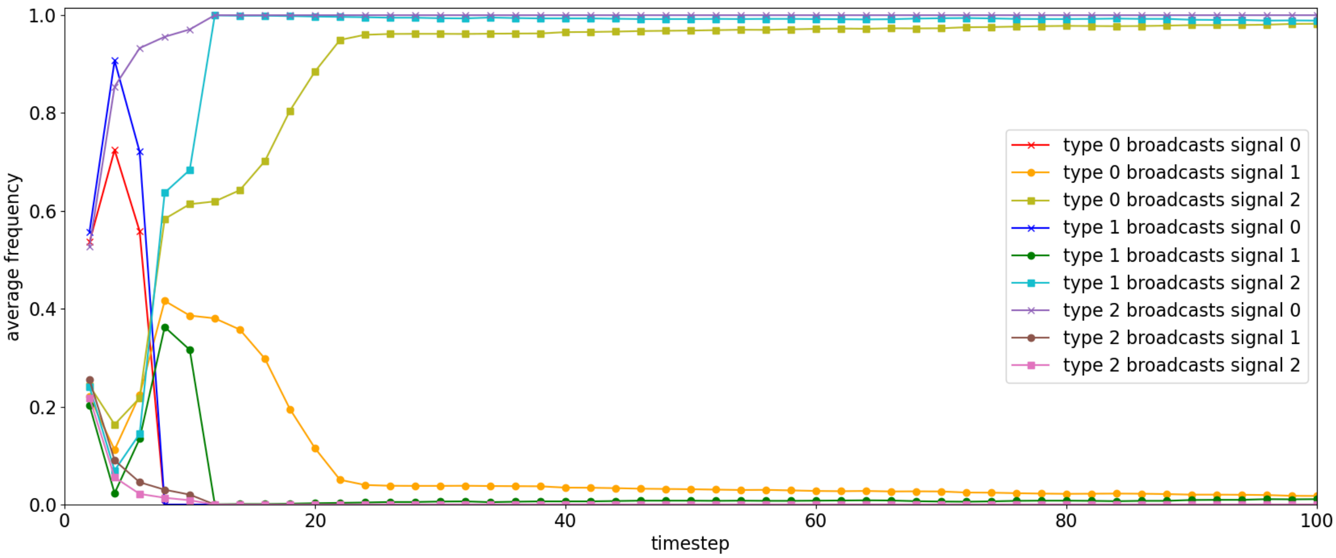

average frequency
timestep
type 0 broadcasts signal 0
type 0 broadcasts signal 1
type 0 broadcasts signal 2
type 1 broadcasts signal 0
type 1 broadcasts signal 1
type 1 broadcasts signal 2
type 2 broadcasts signal 0
type 2 broadcasts signal 1
type 2 broadcasts signal 2

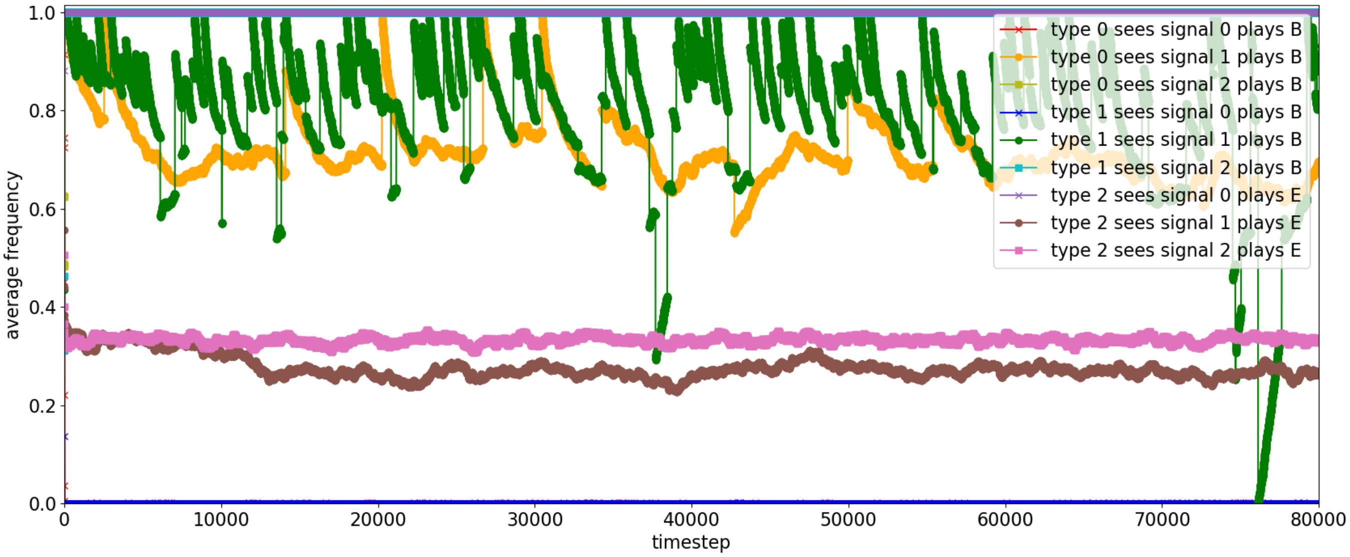

average frequency
timestep
type 0 sees signal 0 plays B
type 0 sees signal 1 plays B
type 0 sees signal 2 plays B
type 1 sees signal 0 plays B
type 1 sees signal 1 plays B
type 1 sees signal 2 plays B
type 2 sees signal 0 plays E
type 2 sees signal 1 plays E
type 2 sees signal 2 plays E

Run # 42
[[[0 3700 0]
[0 2900 0]
[0 0 3400]]]

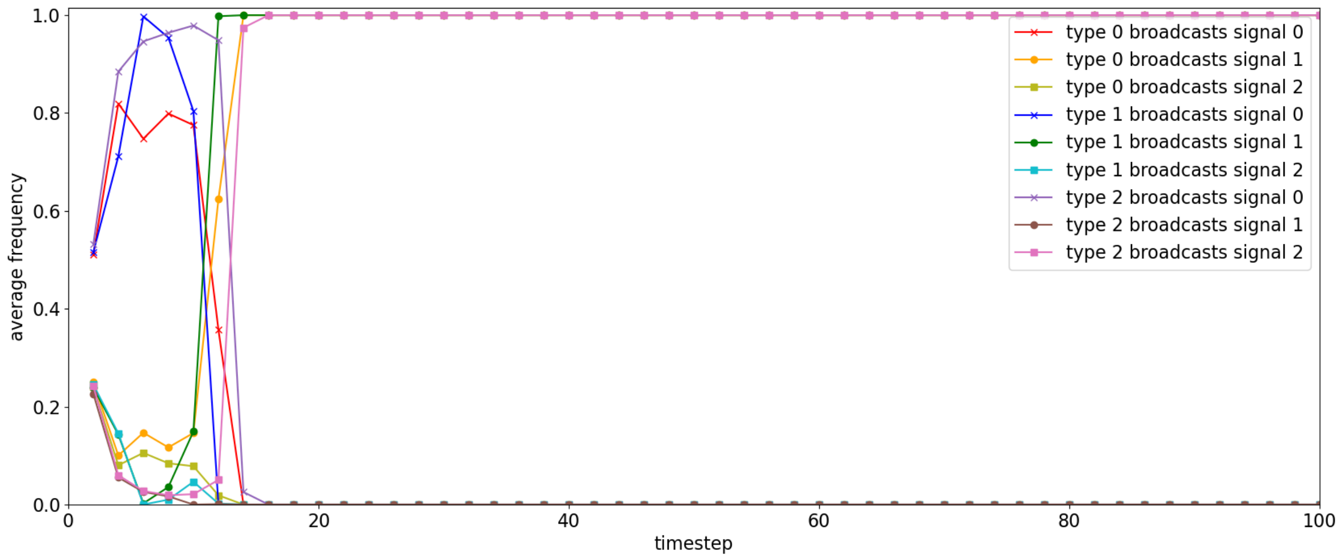

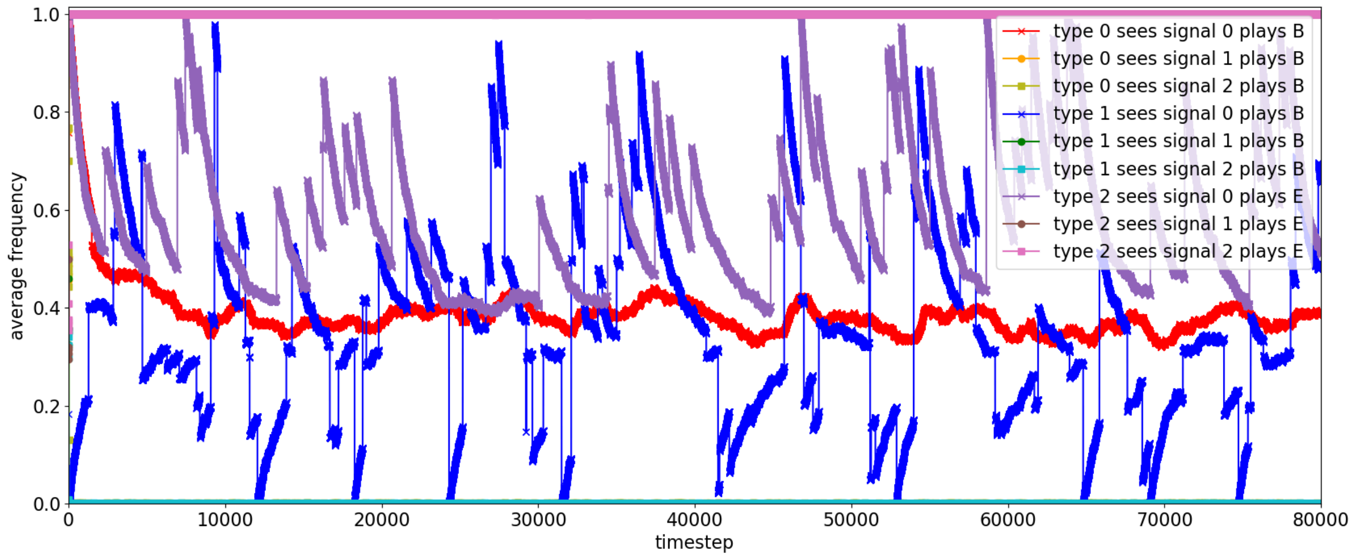

Run # 58

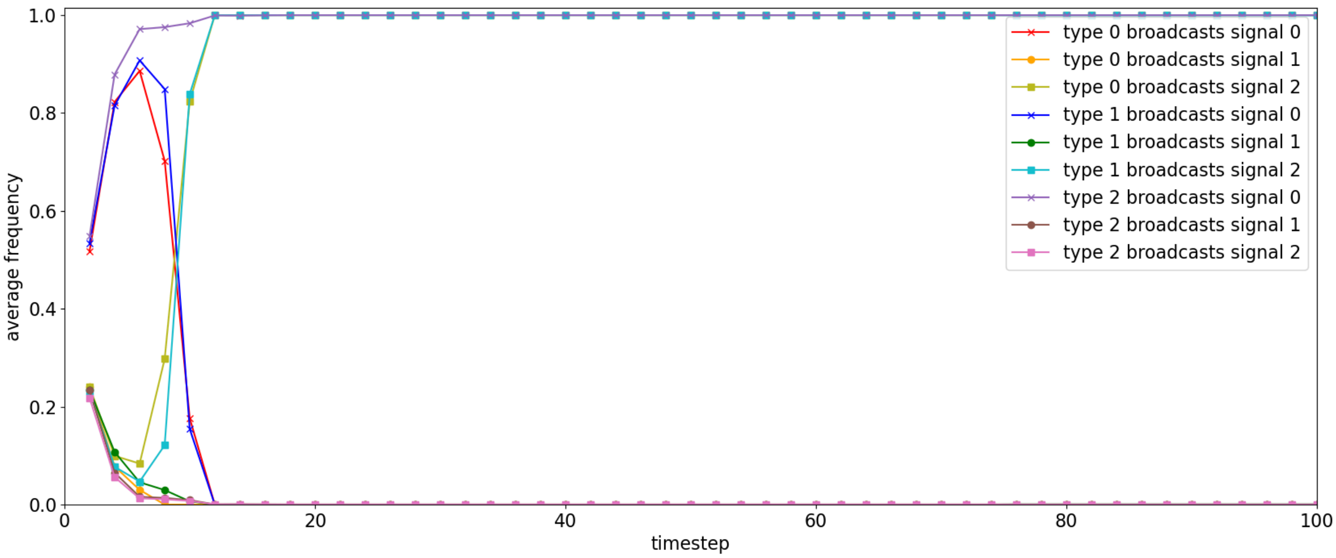
average frequency
timestep
type 0 broadcasts signal 0
type 0 broadcasts signal 1
type 0 broadcasts signal 2
type 1 broadcasts signal 0
type 1 broadcasts signal 1
type 1 broadcasts signal 2
type 2 broadcasts signal 0
type 2 broadcasts signal 1
type 2 broadcasts signal 2

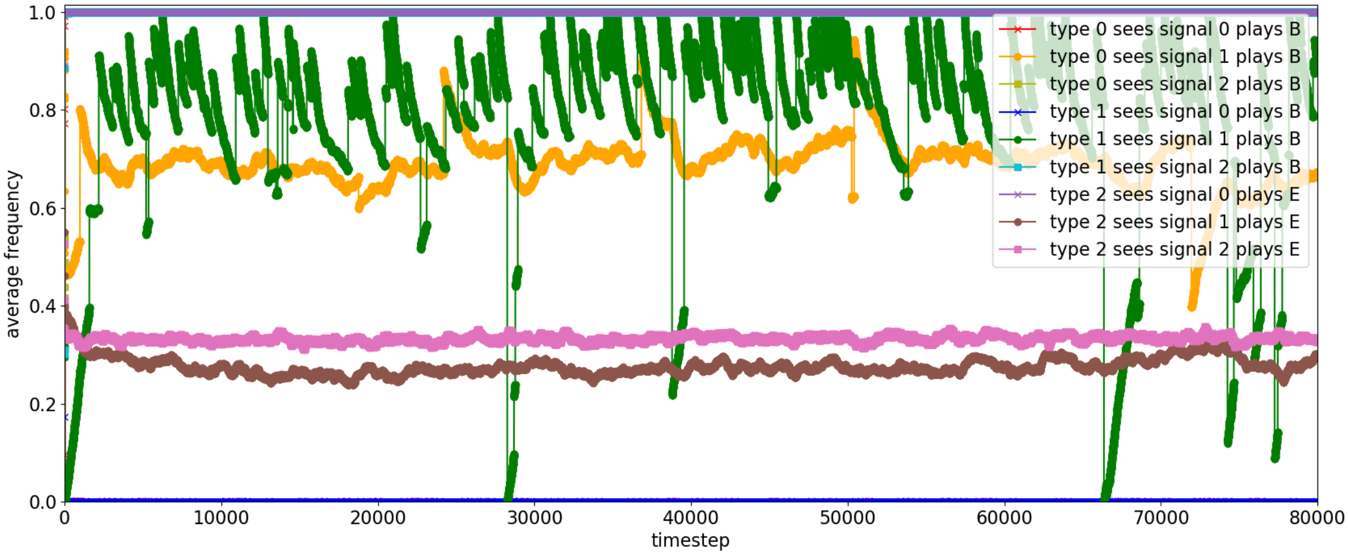
average frequency
timestep
type 0 sees signal 0 plays B
type 0 sees signal 1 plays B
type 0 sees signal 2 plays B
type 1 sees signal 0 plays B
type 1 sees signal 1 plays B
type 1 sees signal 2 plays B
type 2 sees signal 0 plays E
type 2 sees signal 1 plays E
type 2 sees signal 2 plays E

Run # 63

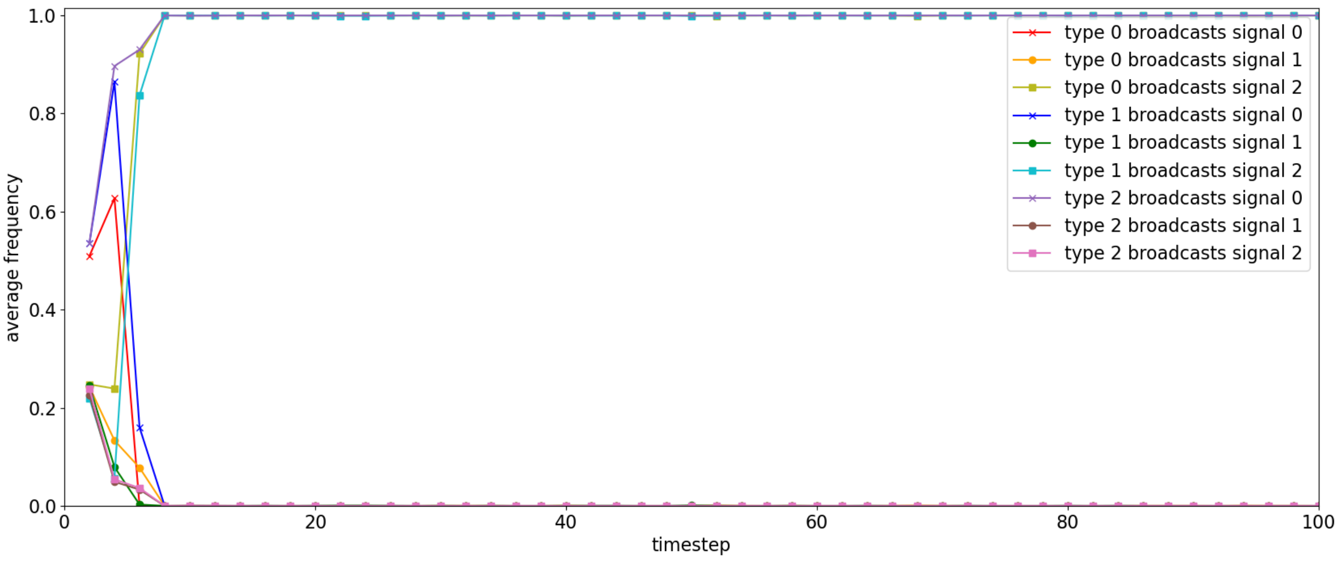

1.0
0.8
0.6
0.4
0.2
0.0
0
20
40
60
80
100
timestep
average frequency
type 0 broadcasts signal 0
type 0 broadcasts signal 1
type 0 broadcasts signal 2
type 1 broadcasts signal 0
type 1 broadcasts signal 1
type 1 broadcasts signal 2
type 2 broadcasts signal 0
type 2 broadcasts signal 1
type 2 broadcasts signal 2

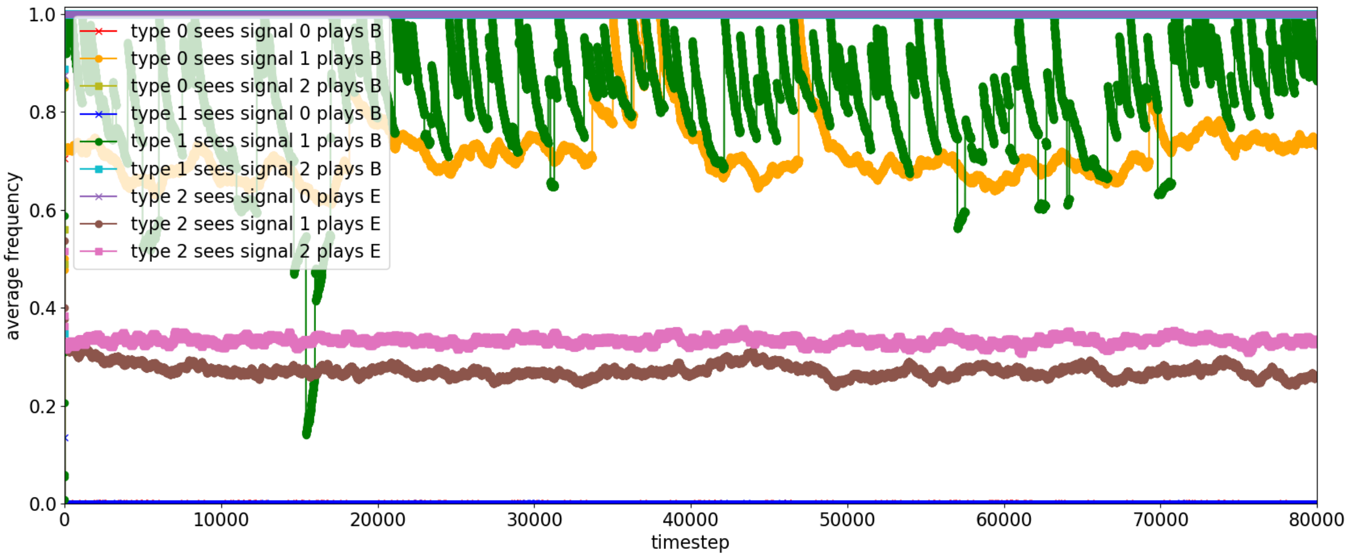

1.0
0.8
0.6
0.4
0.2
0.0
0
10000
20000
30000
40000
50000
60000
70000
80000
timestep
average frequency
type 0 sees signal 0 plays B
type 0 sees signal 1 plays B
type 0 sees signal 2 plays B
type 1 sees signal 0 plays B
type 1 sees signal 1 plays B
type 1 sees signal 2 plays B
type 2 sees signal 0 plays E
type 2 sees signal 1 plays E
type 2 sees signal 2 plays E

Run # 85

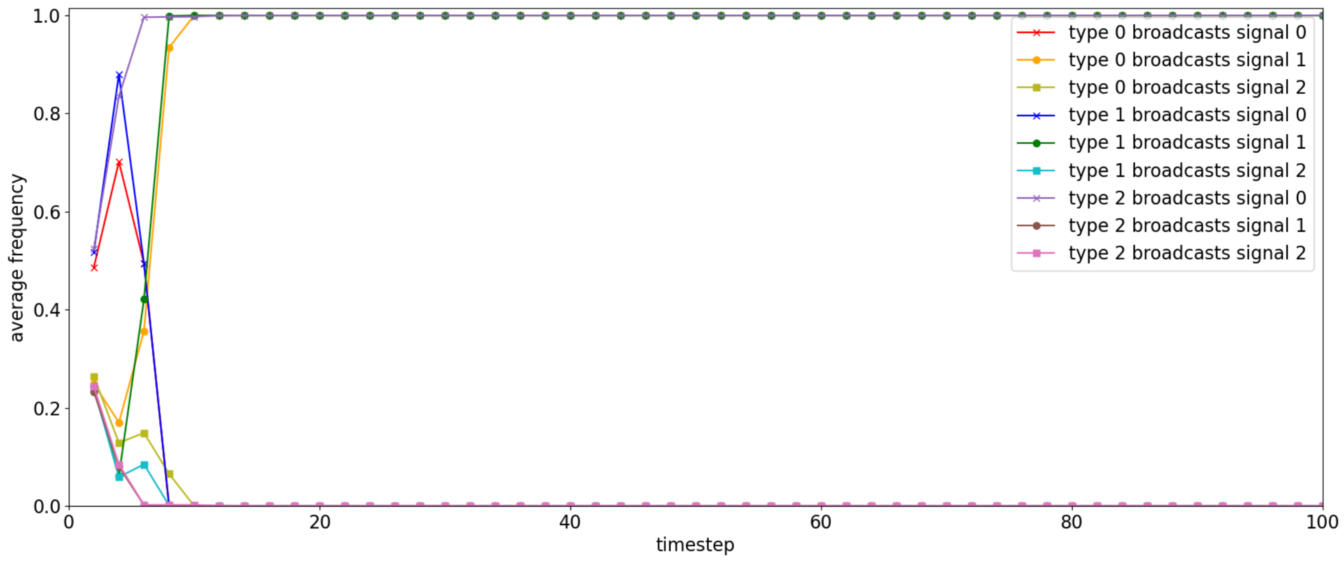
average frequency
timestep
type 0 broadcasts signal 0
type 0 broadcasts signal 1
type 0 broadcasts signal 2
type 1 broadcasts signal 0
type 1 broadcasts signal 1
type 1 broadcasts signal 2
type 2 broadcasts signal 0
type 2 broadcasts signal 1
type 2 broadcasts signal 2

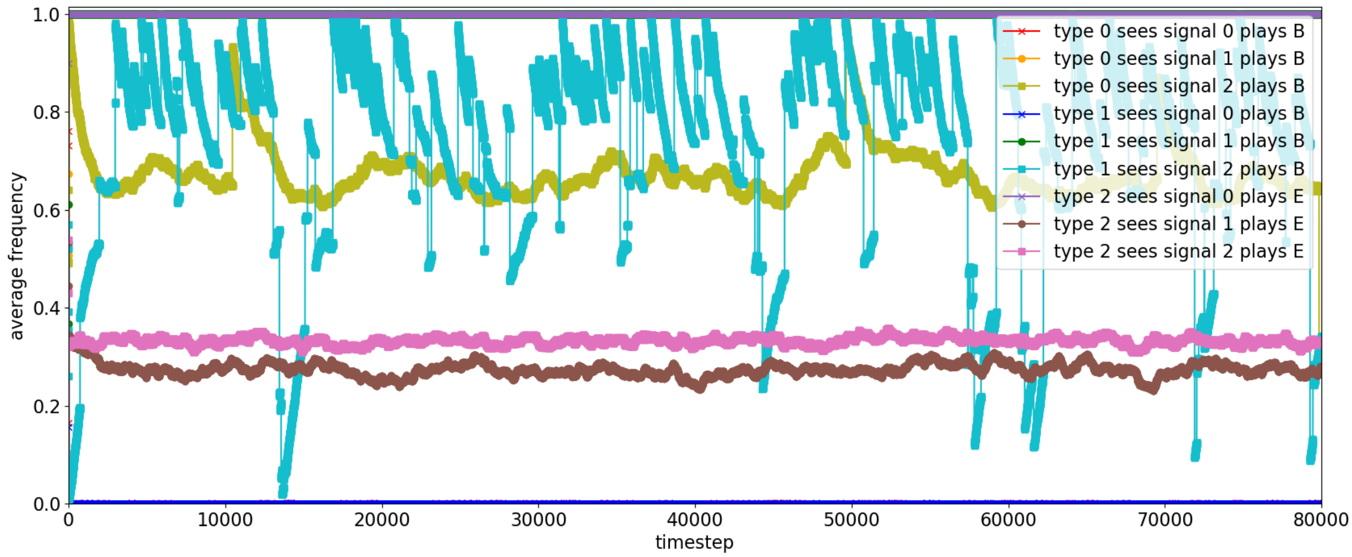
average frequency
timestep
type 0 sees signal 0 plays B
type 0 sees signal 1 plays B
type 0 sees signal 2 plays B
type 1 sees signal 0 plays B
type 1 sees signal 1 plays B
type 1 sees signal 2 plays B
type 2 sees signal 0 plays E
type 2 sees signal 1 plays E
type 2 sees signal 2 plays E

outcomes (v): sometimes type 2 signaling 0 and types 0 and 1 sending same signal, but also frequent cyclical behavior

Run # 32

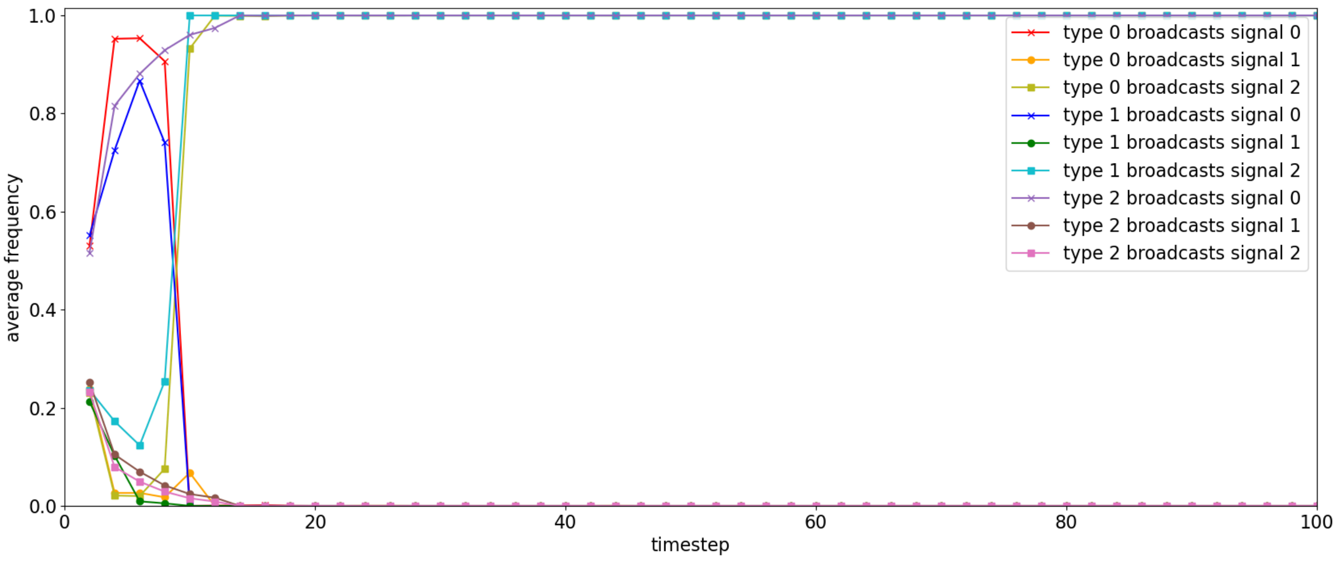

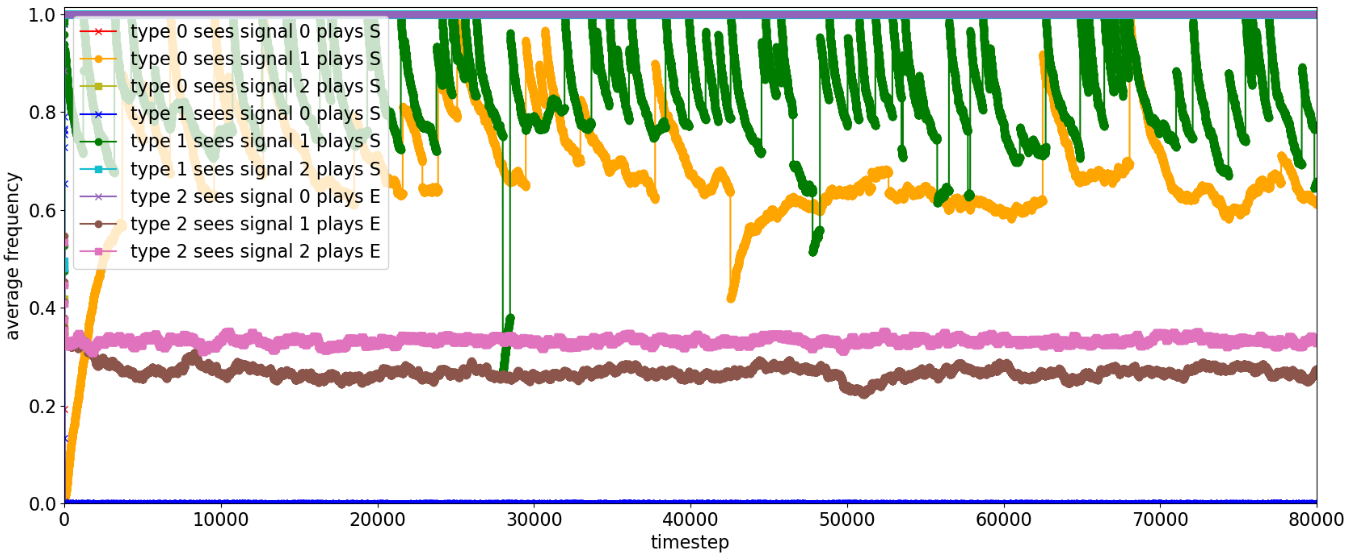

Run # 43

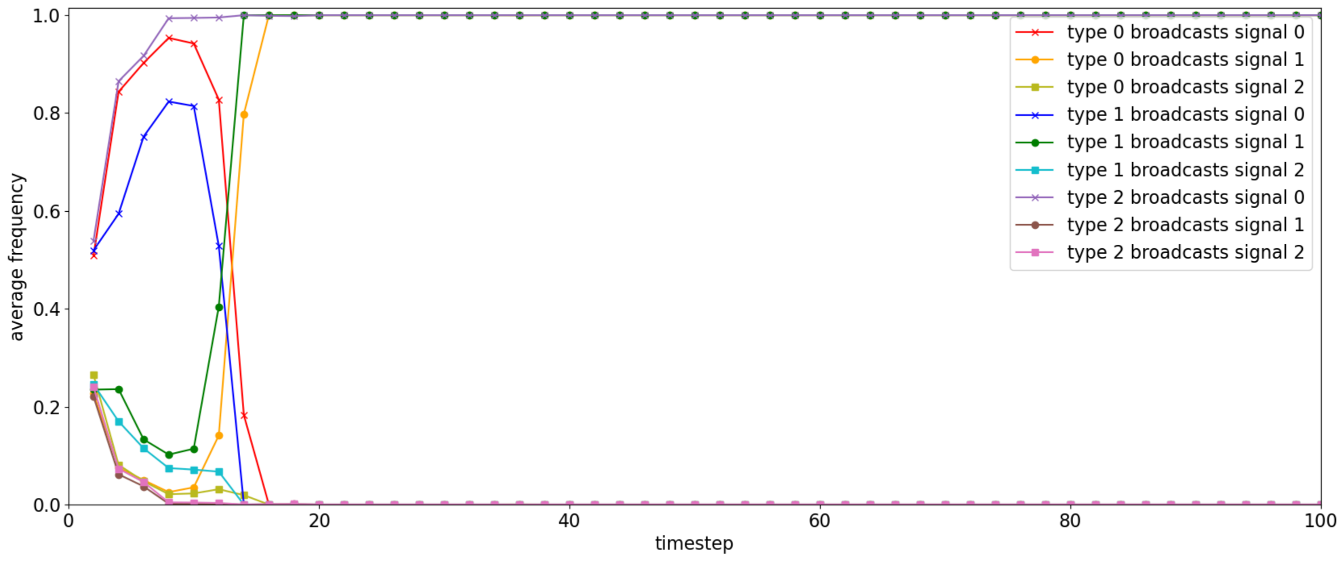
average frequency
timestep
type 0 broadcasts signal 0
type 0 broadcasts signal 1
type 0 broadcasts signal 2
type 1 broadcasts signal 0
type 1 broadcasts signal 1
type 1 broadcasts signal 2
type 2 broadcasts signal 0
type 2 broadcasts signal 1
type 2 broadcasts signal 2

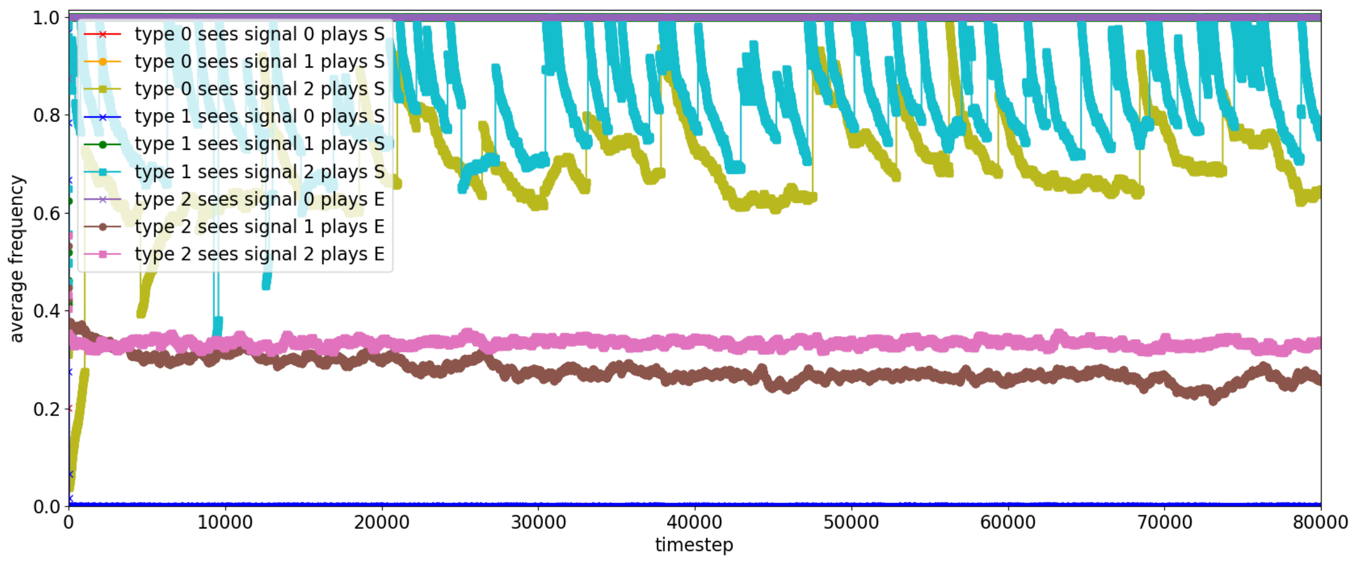
average frequency
timestep
type 0 sees signal 0 plays S
type 0 sees signal 1 plays S
type 0 sees signal 2 plays S
type 1 sees signal 0 plays S
type 1 sees signal 1 plays S
type 1 sees signal 2 plays S
type 2 sees signal 0 plays E
type 2 sees signal 1 plays E
type 2 sees signal 2 plays E

Run # 67
[[[149   0 3551]
  [0   0 2900]
  [0 3400   0]]]

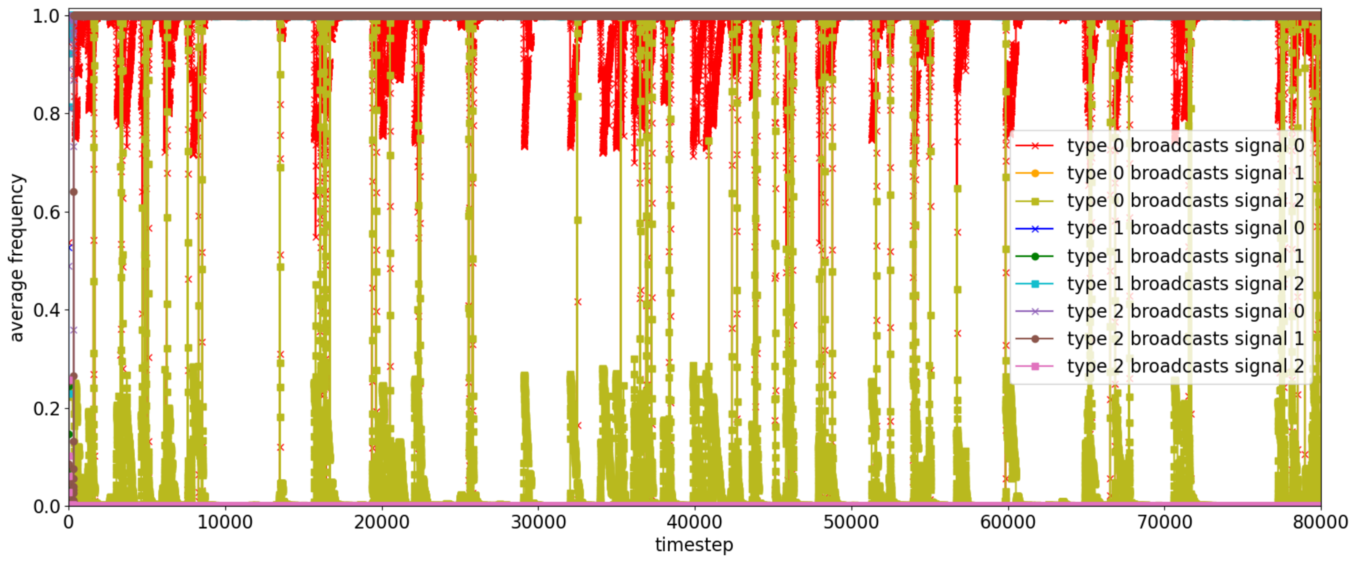

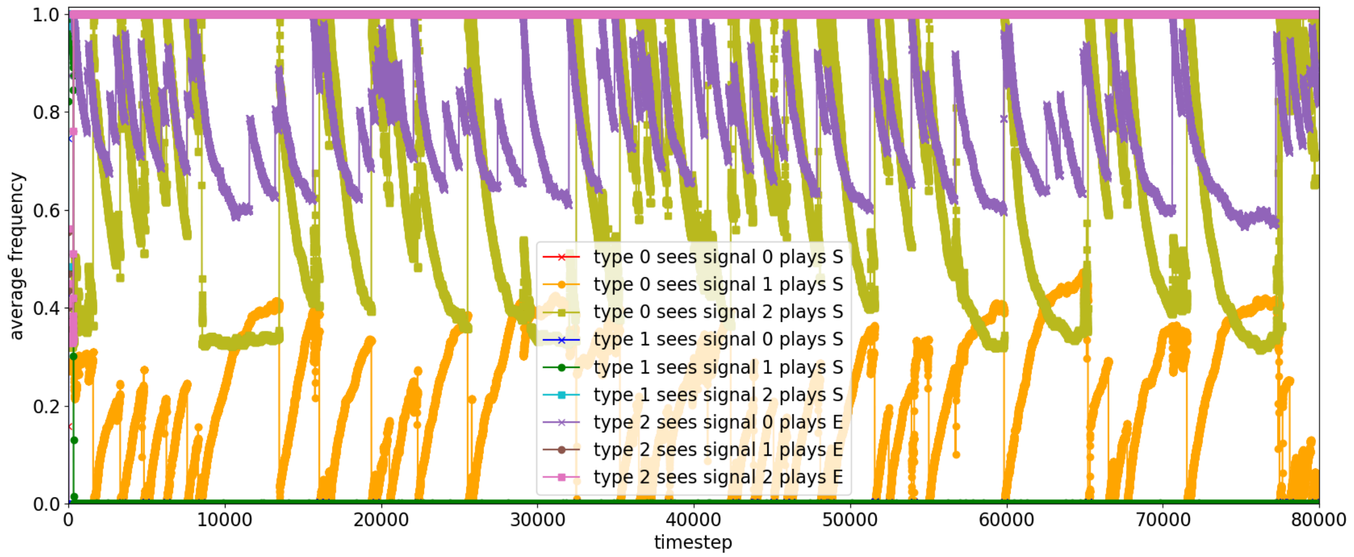

Run # 125

```
[[[  11 3689    0]
  [   0 2900    0]
  [   0    0 3400]]]
```

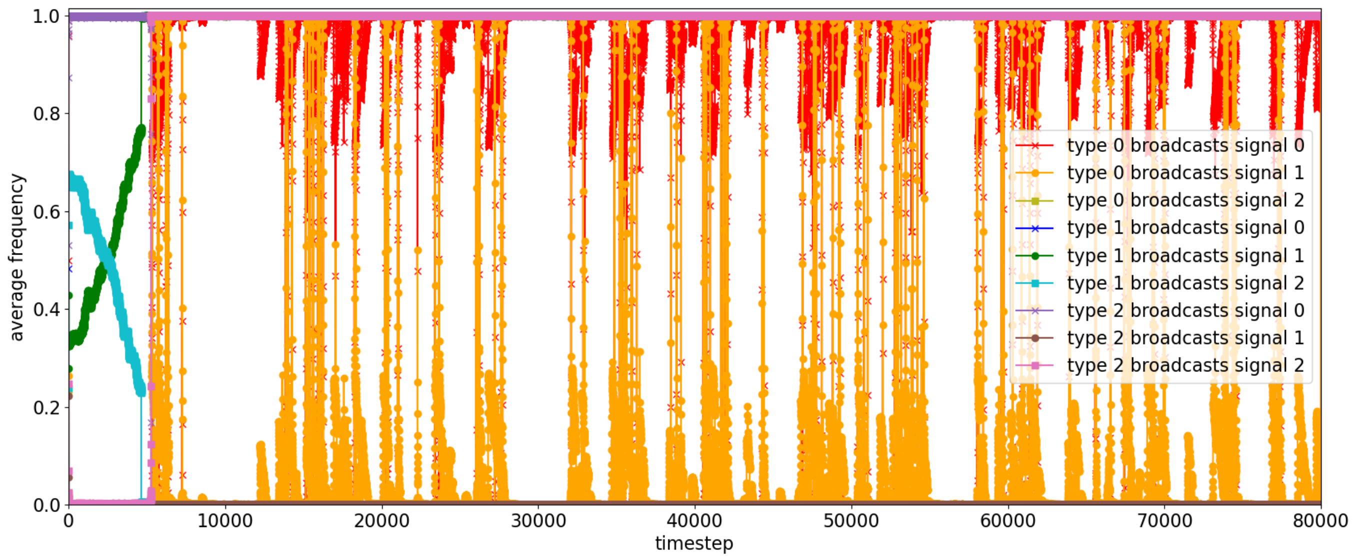

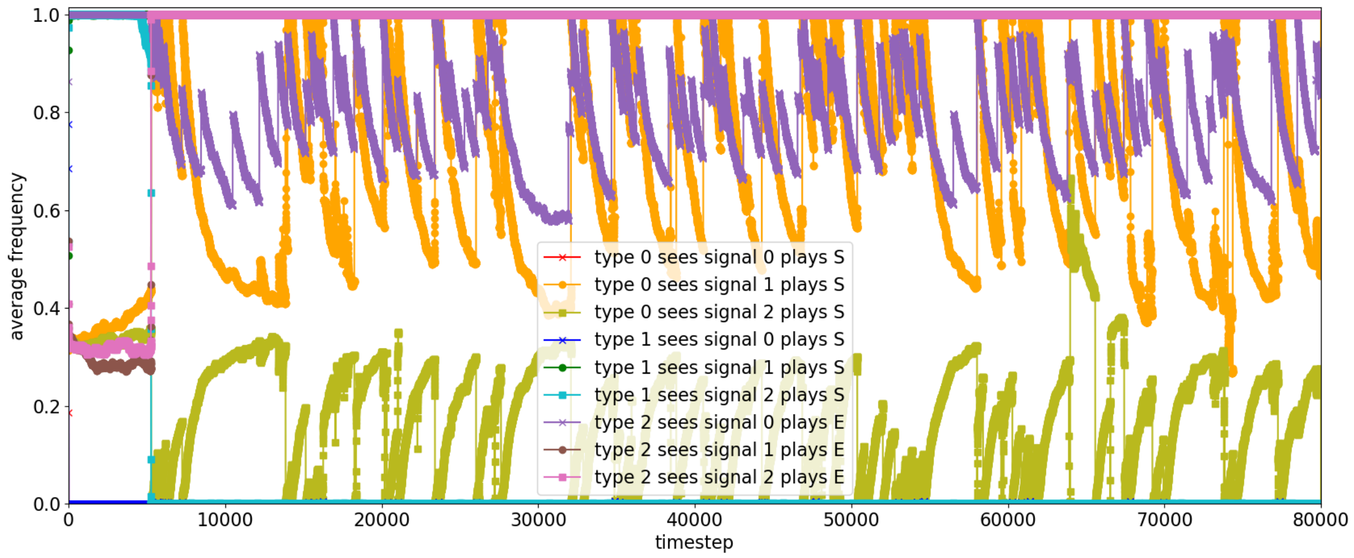

Run # 133

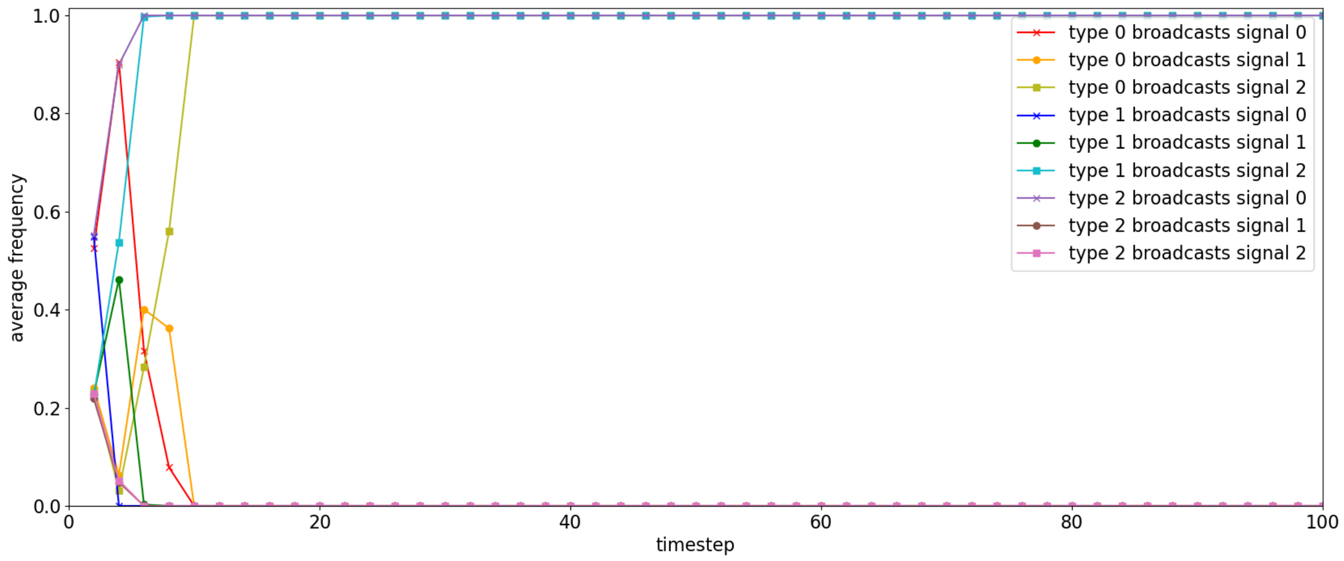
type 0 broadcasts signal 0
type 0 broadcasts signal 1
type 0 broadcasts signal 2
type 1 broadcasts signal 0
type 1 broadcasts signal 1
type 1 broadcasts signal 2
type 2 broadcasts signal 0
type 2 broadcasts signal 1
type 2 broadcasts signal 2
average frequency
timestep
0.0
0.2
0.4
0.6
0.8
1.0
0
20
40
60
80
100

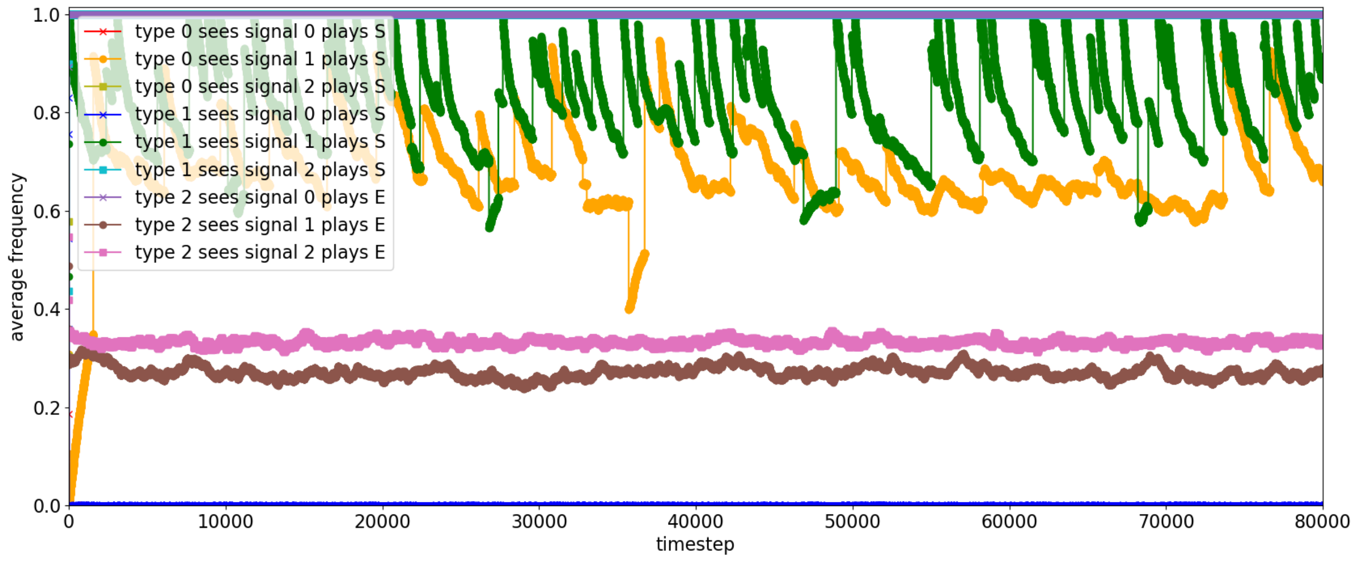
type 0 sees signal 0 plays S
type 0 sees signal 1 plays S
type 0 sees signal 2 plays S
type 1 sees signal 0 plays S
type 1 sees signal 1 plays S
type 1 sees signal 2 plays S
type 2 sees signal 0 plays E
type 2 sees signal 1 plays E
type 2 sees signal 2 plays E
average frequency
timestep
0.0
0.2
0.4
0.6
0.8
1.0
0
10000
20000
30000
40000
50000
60000
70000
80000

Run # 179

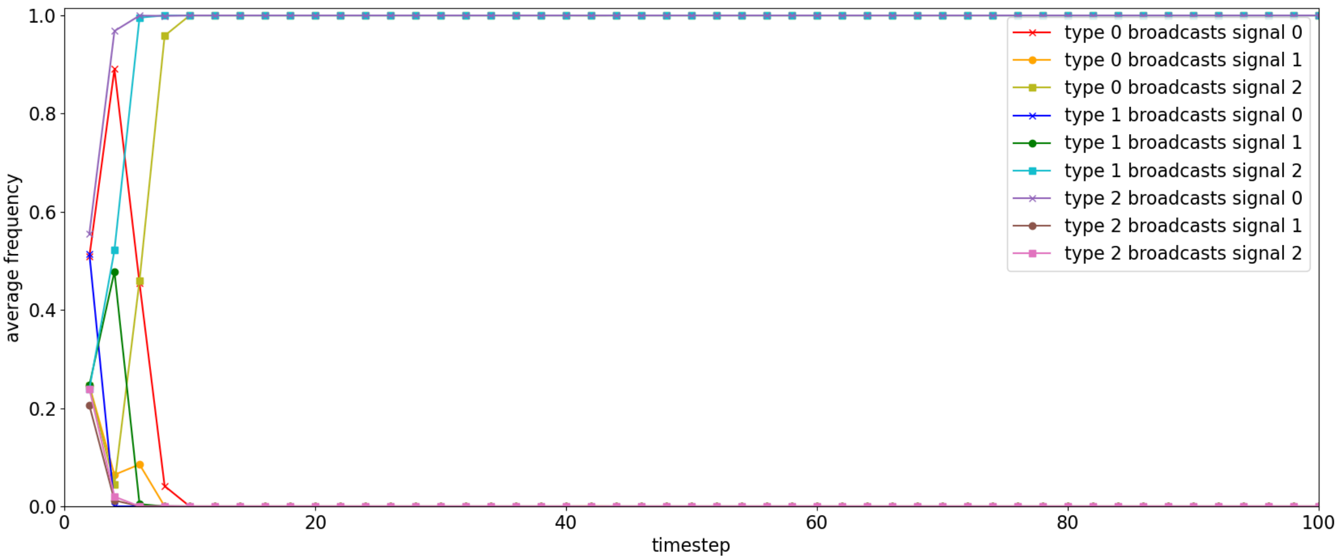
average frequency
timestep
type 0 broadcasts signal 0
type 0 broadcasts signal 1
type 0 broadcasts signal 2
type 1 broadcasts signal 0
type 1 broadcasts signal 1
type 1 broadcasts signal 2
type 2 broadcasts signal 0
type 2 broadcasts signal 1
type 2 broadcasts signal 2

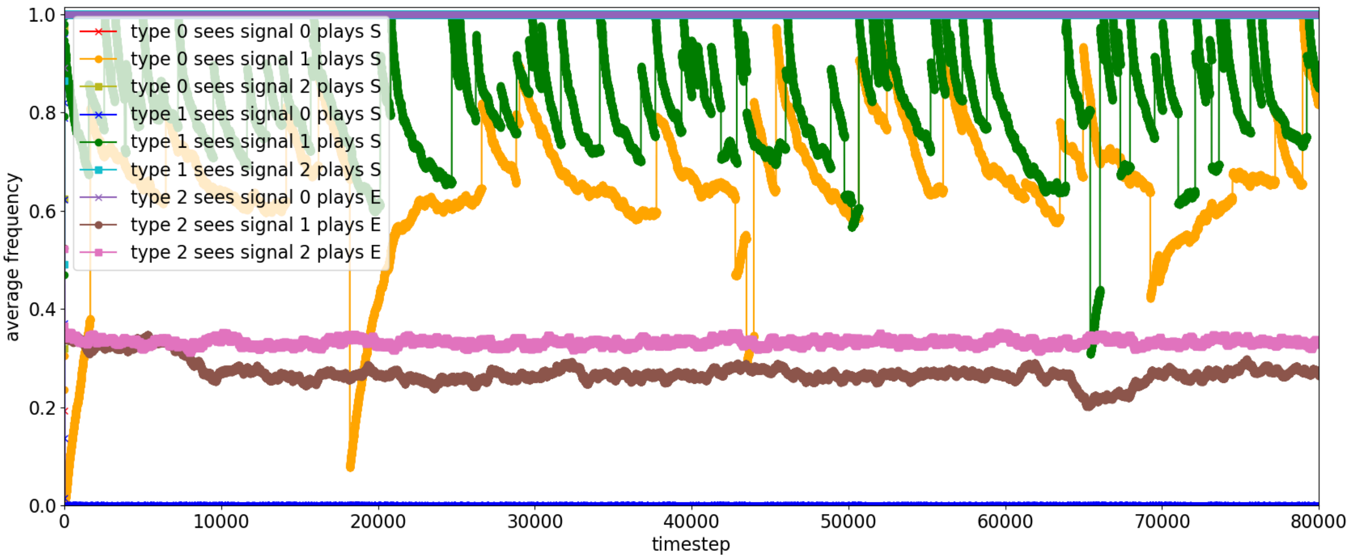
average frequency
timestep
type 0 sees signal 0 plays S
type 0 sees signal 1 plays S
type 0 sees signal 2 plays S
type 1 sees signal 0 plays S
type 1 sees signal 1 plays S
type 1 sees signal 2 plays S
type 2 sees signal 0 plays E
type 2 sees signal 1 plays E
type 2 sees signal 2 plays E

Run # 188
[[[1646 2054   0]
  [0 2900   0]
  [0   0 3400]]]

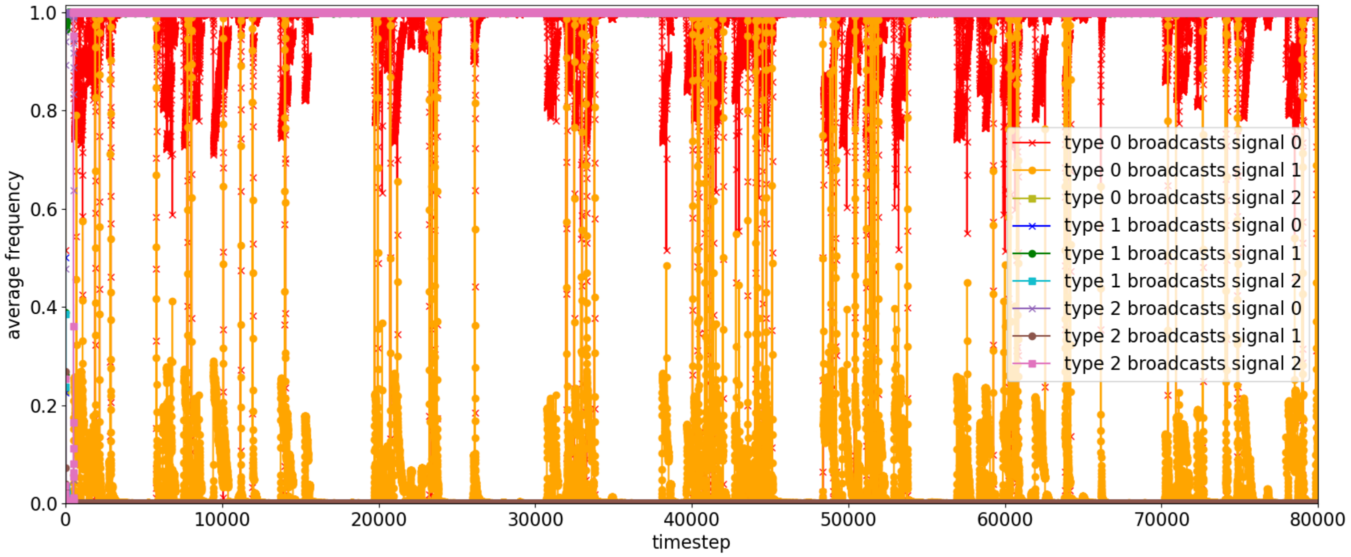

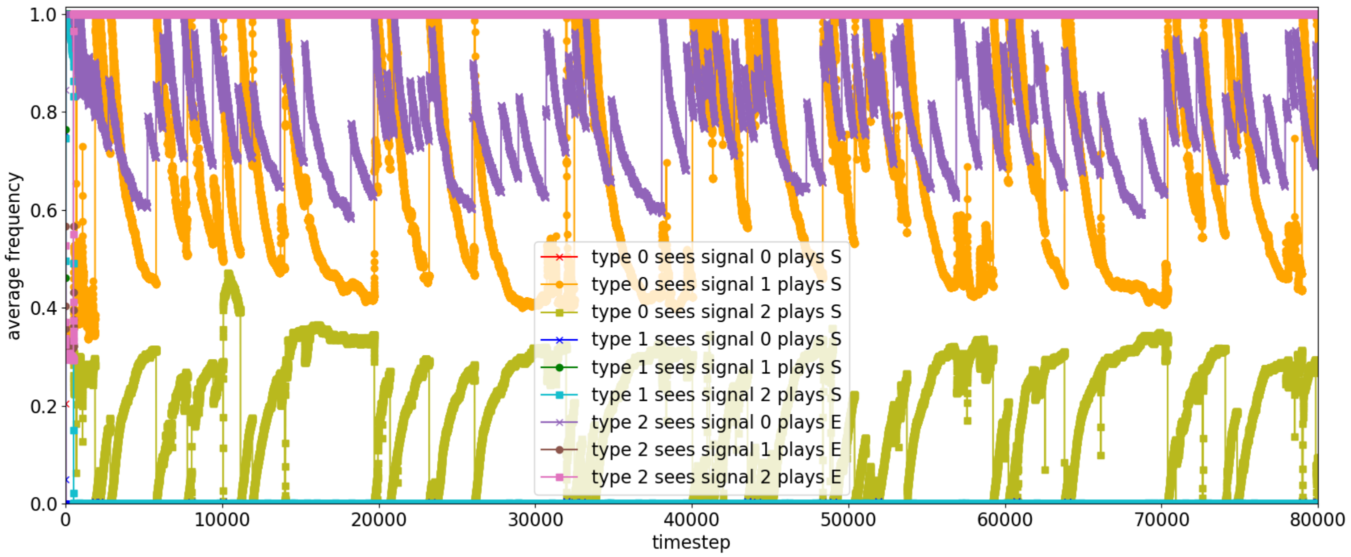

Run # 199
[[[30   0 3670]
  [0   0 2900]
  [0 3400   0]]]
Type 0 continues oscillating between signal 0 and 2

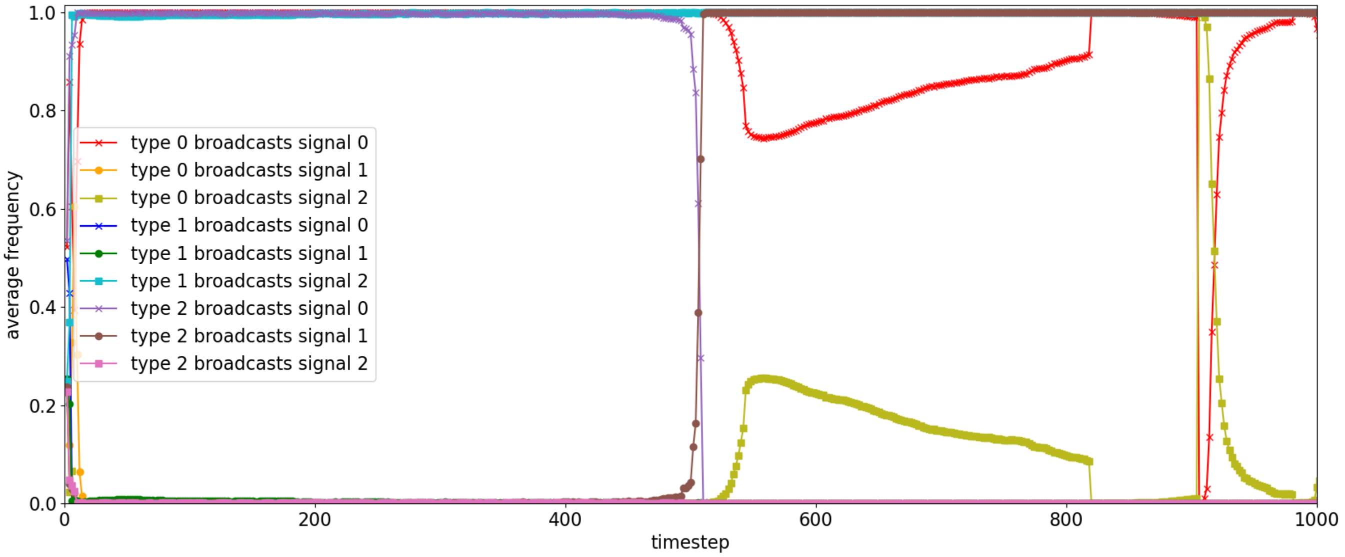

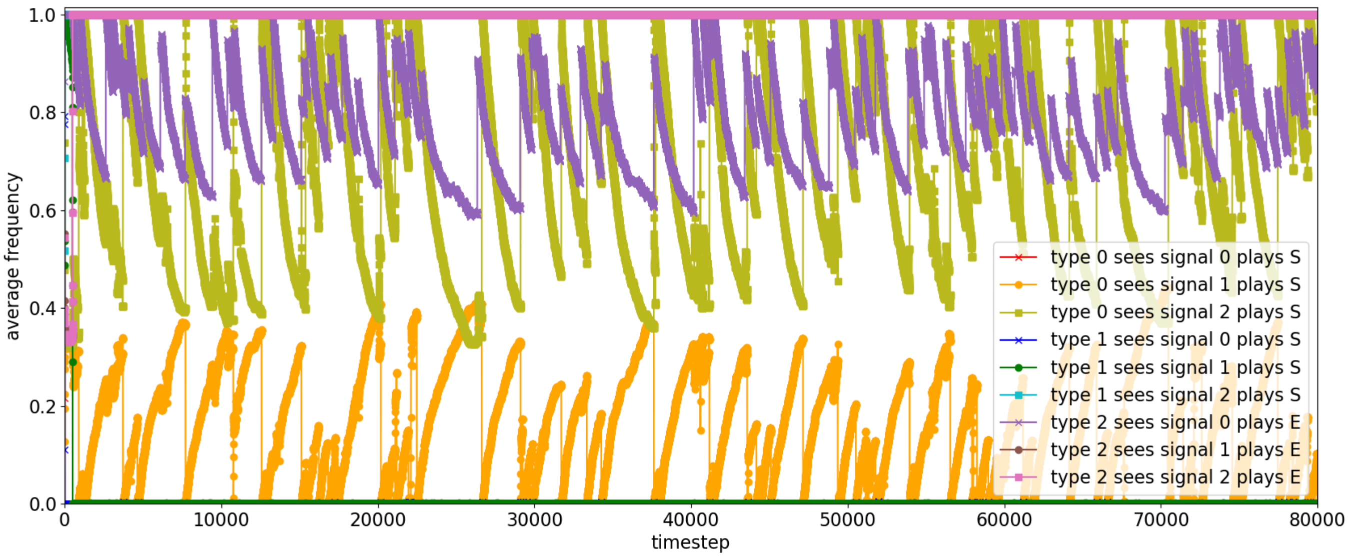

Run # 200
[[[177   0 3523]
  [0   0 2900]
  [0 3400   0]]]
Type 0 continues oscillating between signal 0 and 2

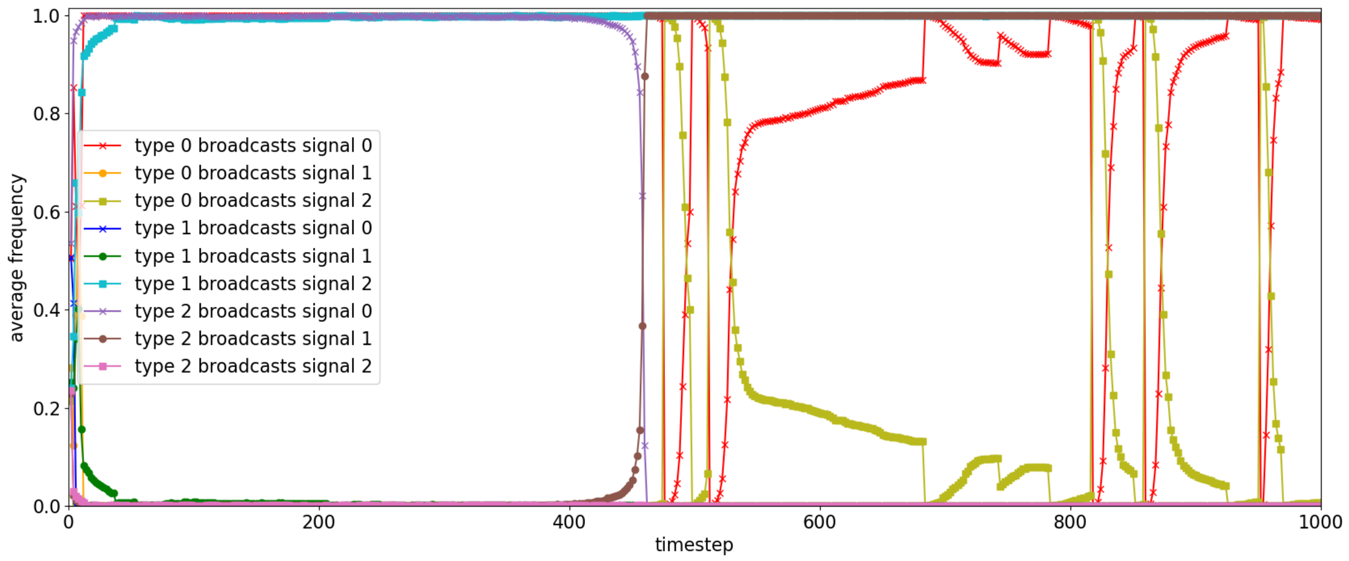

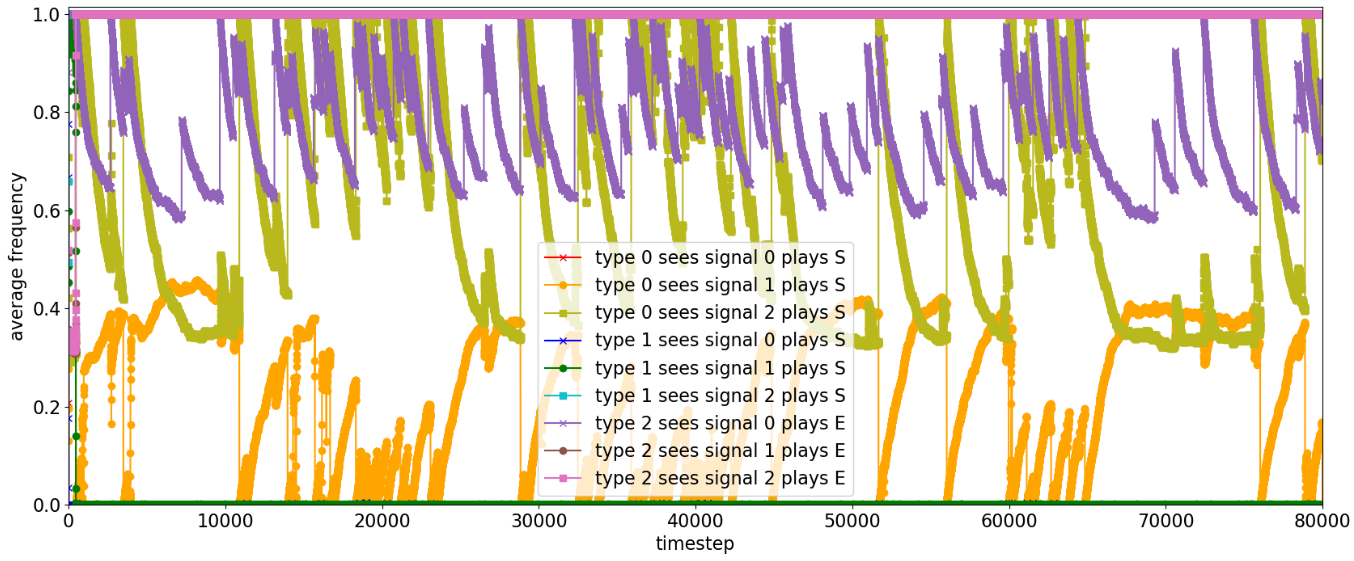

Run # 236

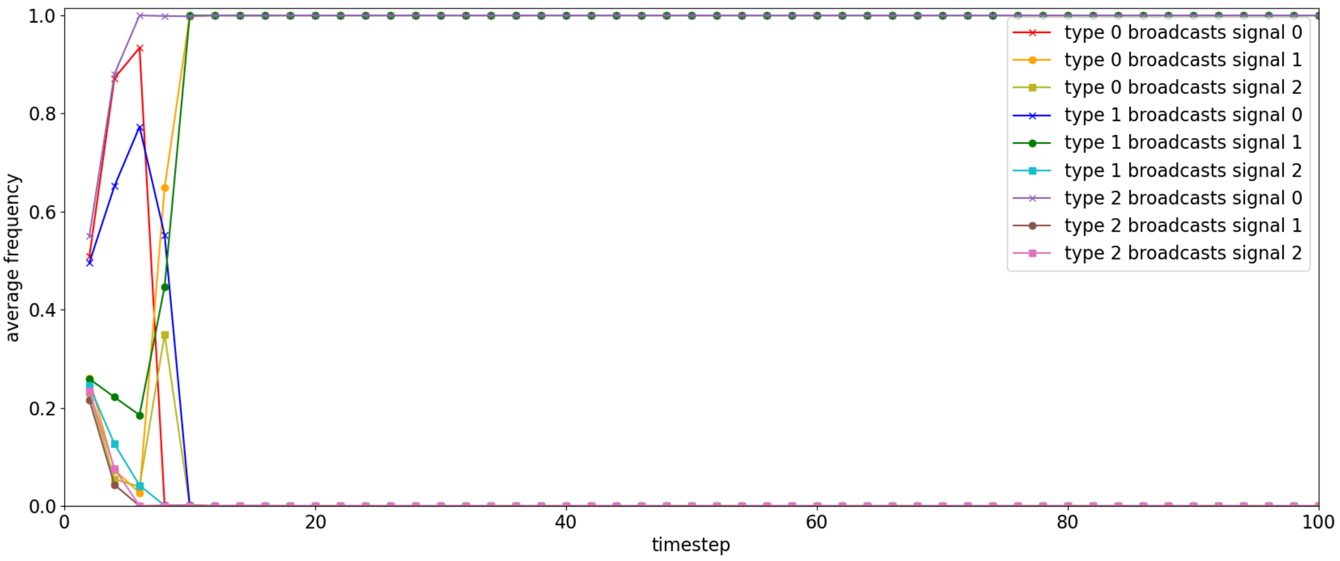

average frequency
timestep
type 0 broadcasts signal 0
type 0 broadcasts signal 1
type 0 broadcasts signal 2
type 1 broadcasts signal 0
type 1 broadcasts signal 1
type 1 broadcasts signal 2
type 2 broadcasts signal 0
type 2 broadcasts signal 1
type 2 broadcasts signal 2

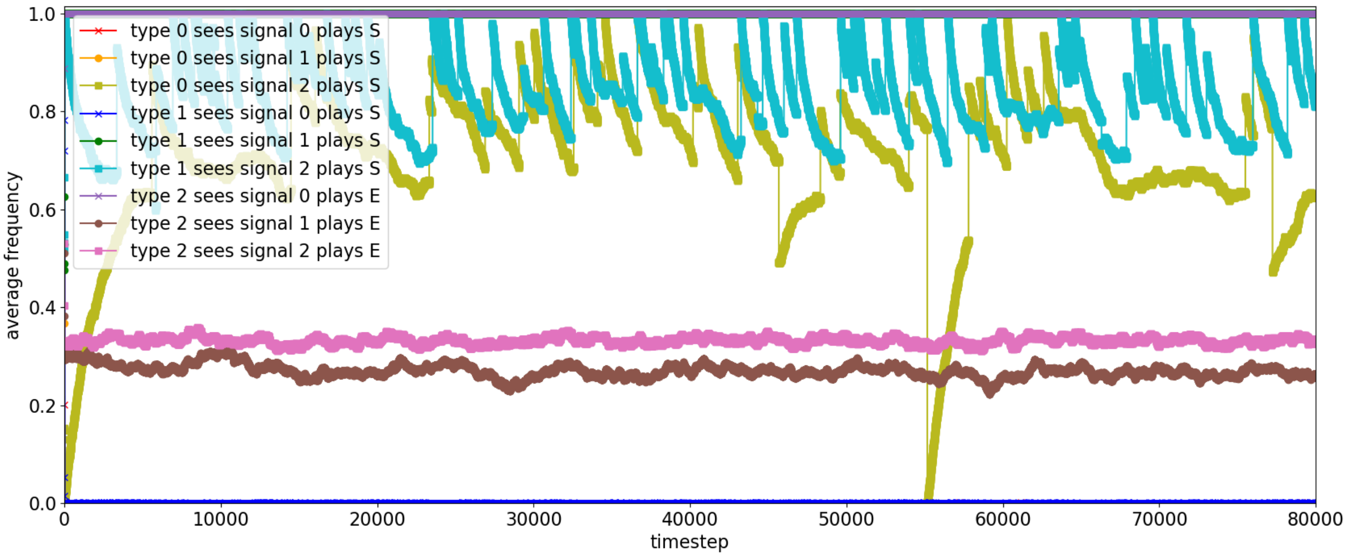

average frequency
timestep
type 0 sees signal 0 plays S
type 0 sees signal 1 plays S
type 0 sees signal 2 plays S
type 1 sees signal 0 plays S
type 1 sees signal 1 plays S
type 1 sees signal 2 plays S
type 2 sees signal 0 plays E
type 2 sees signal 1 plays E
type 2 sees signal 2 plays E

outcomes (viii): characterized by type 0 signaling 0

Run # 1

[[[3486   0 214]
  [1   0 2899]
  [0 3400   0]]]

Continued type 0 fluctuations between signal 0 and 2

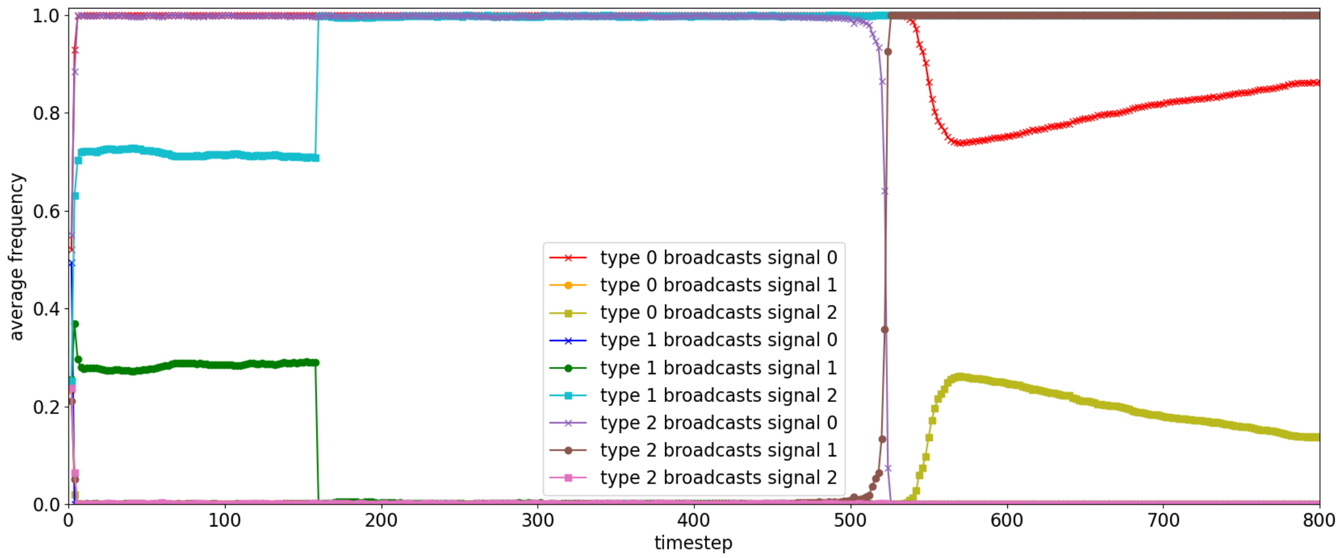

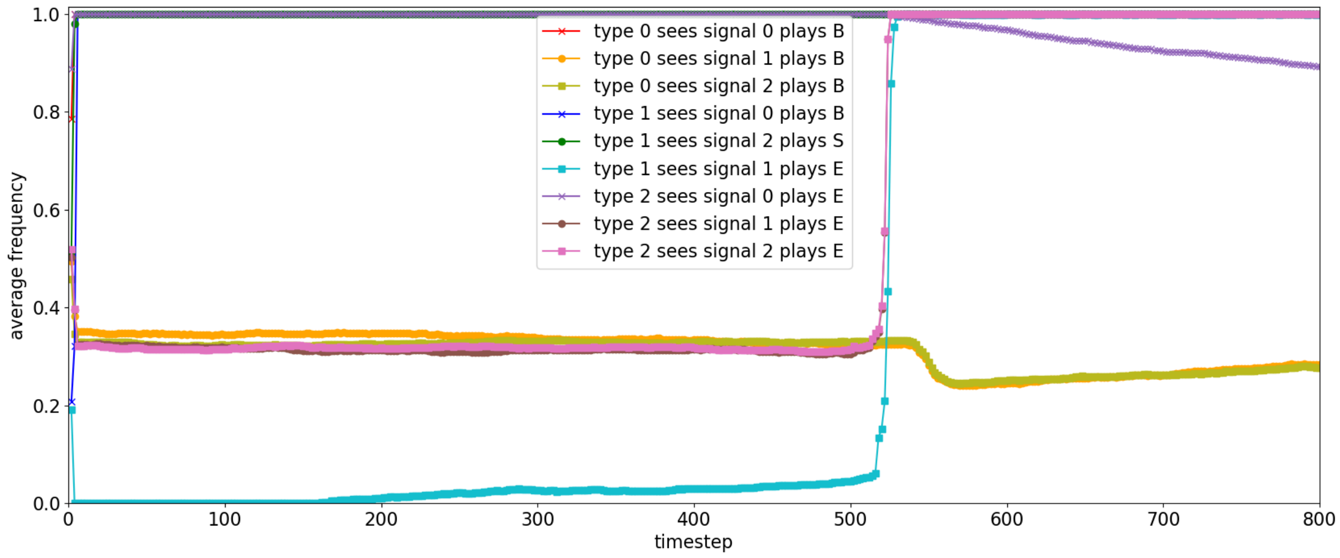

More of Run # 1

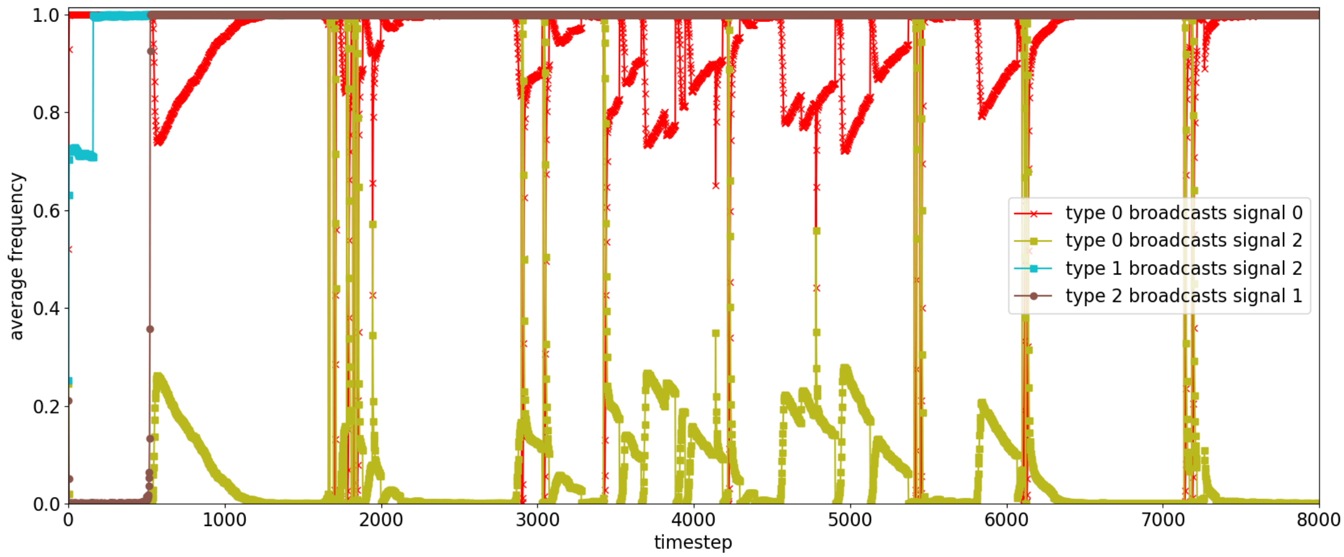
average frequency
timestep
type 0 broadcasts signal 0
type 0 broadcasts signal 2
type 1 broadcasts signal 2
type 2 broadcasts signal 1

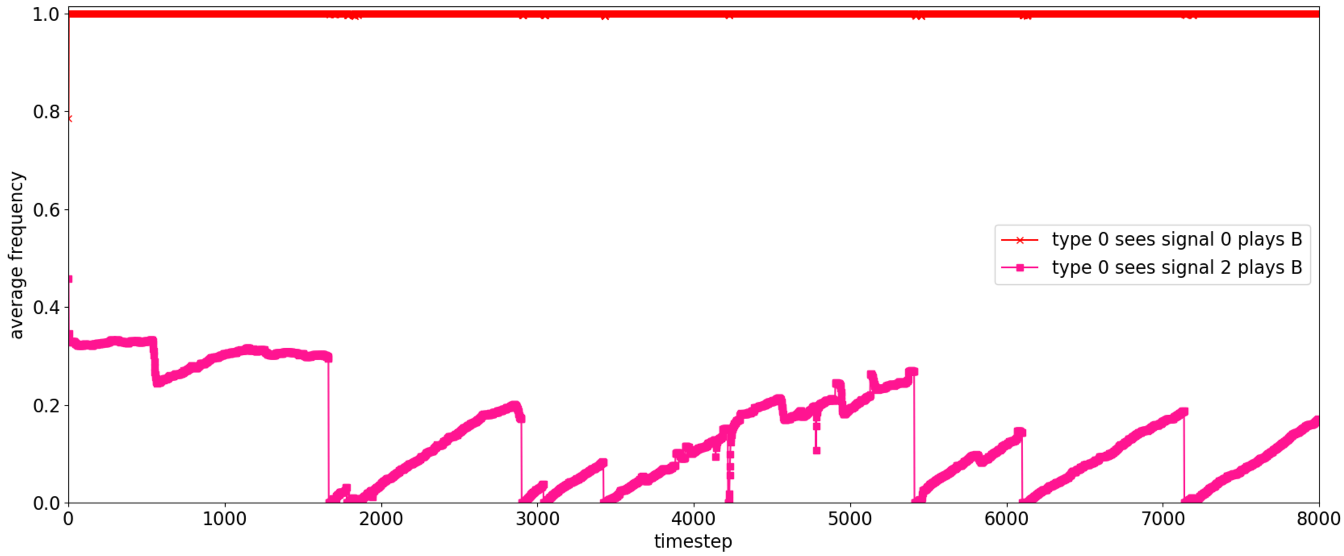
average frequency
timestep
type 0 sees signal 0 plays B
type 0 sees signal 2 plays B

Run # 2

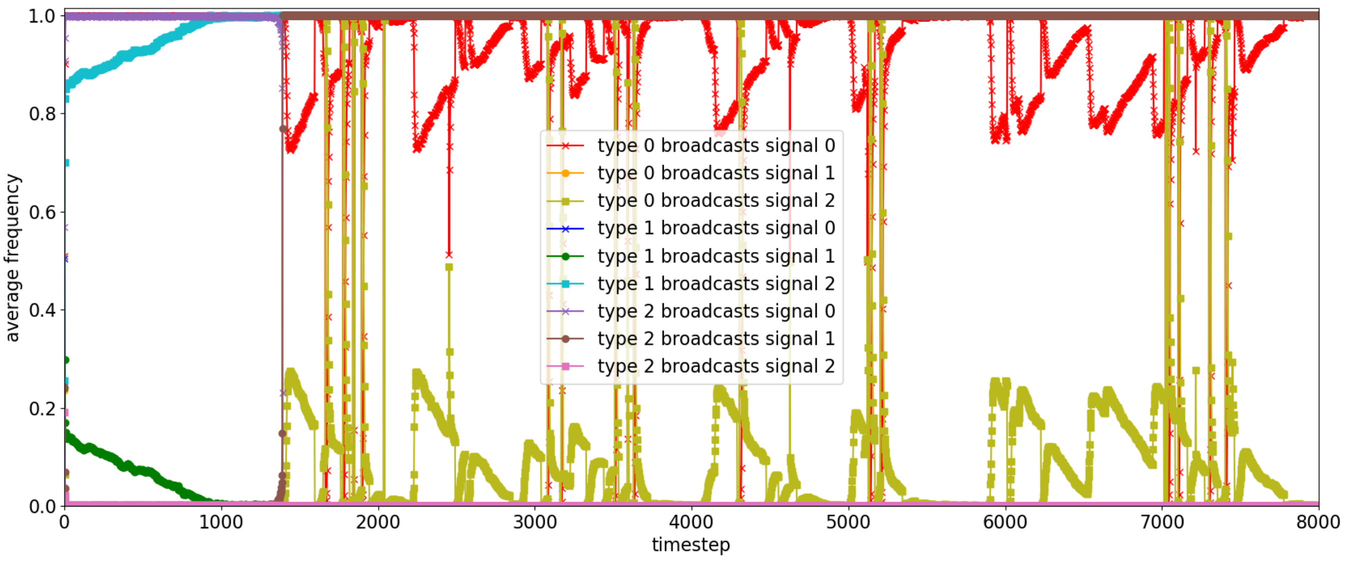

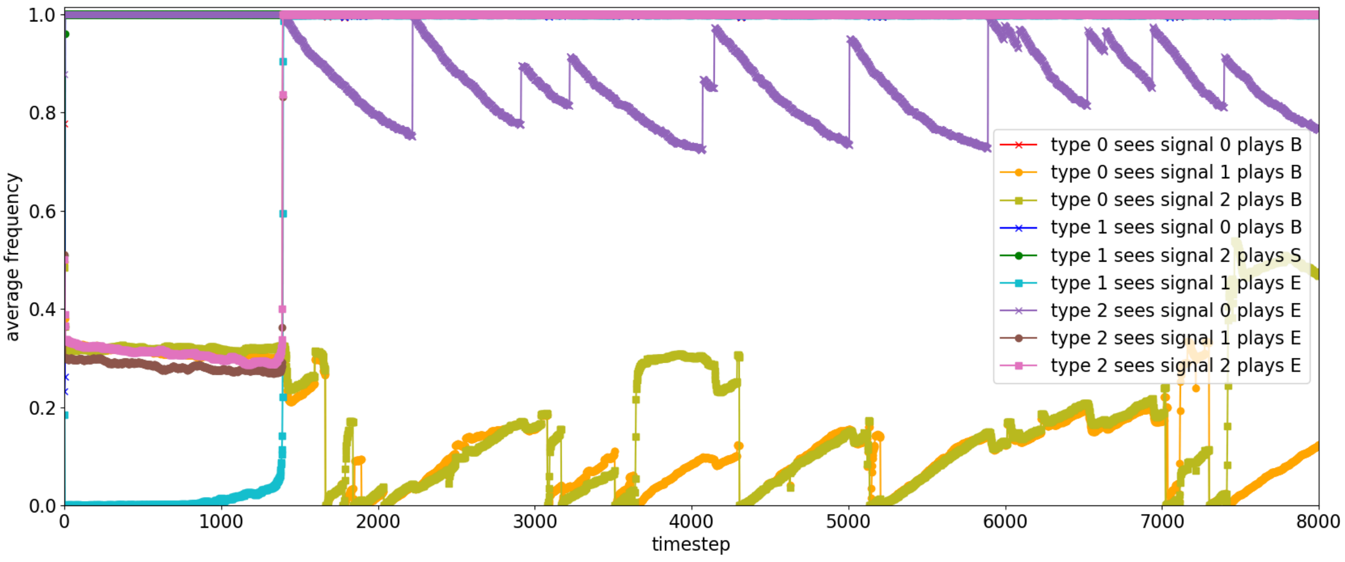

Run # 2 continued on the next page. It can look like type 0 is playing E when paired with type 2, but recall that since type 0 is signaling 0, that means type 0 is interacting with all agents as if they were signaling 0.

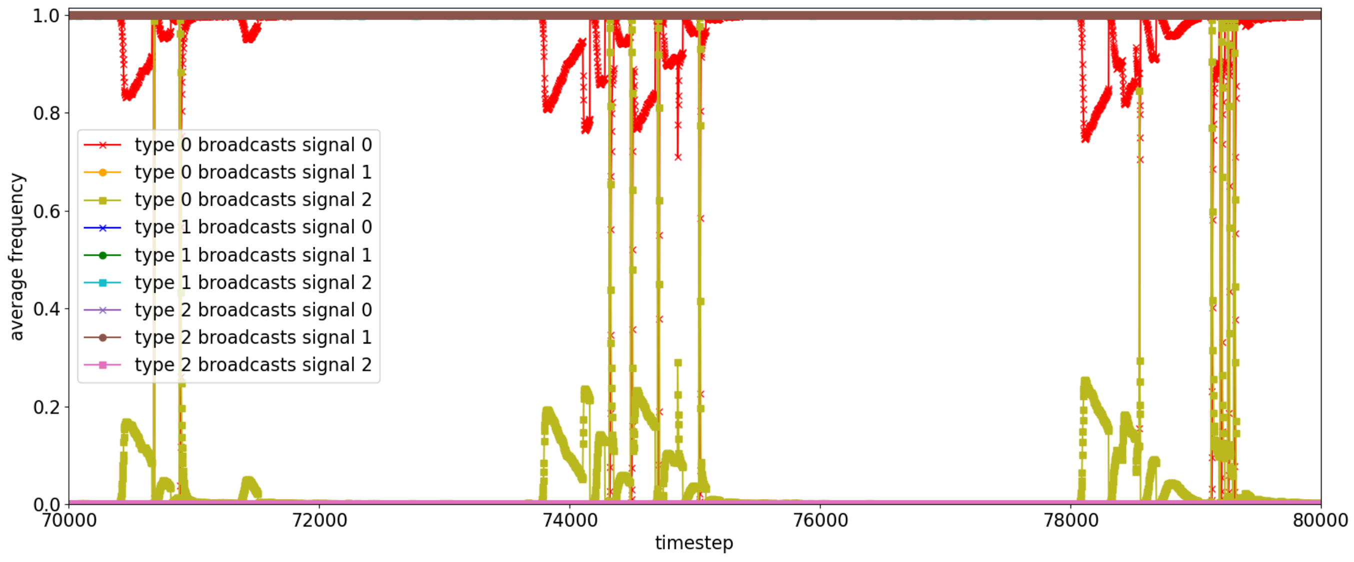
1.0
0.8
0.6
0.4
0.2
0.0
average frequency
type 0 broadcasts signal 0
type 0 broadcasts signal 1
type 0 broadcasts signal 2
type 1 broadcasts signal 0
type 1 broadcasts signal 1
type 1 broadcasts signal 2
type 2 broadcasts signal 0
type 2 broadcasts signal 1
type 2 broadcasts signal 2
70000
72000
74000
76000
78000
80000
timestep

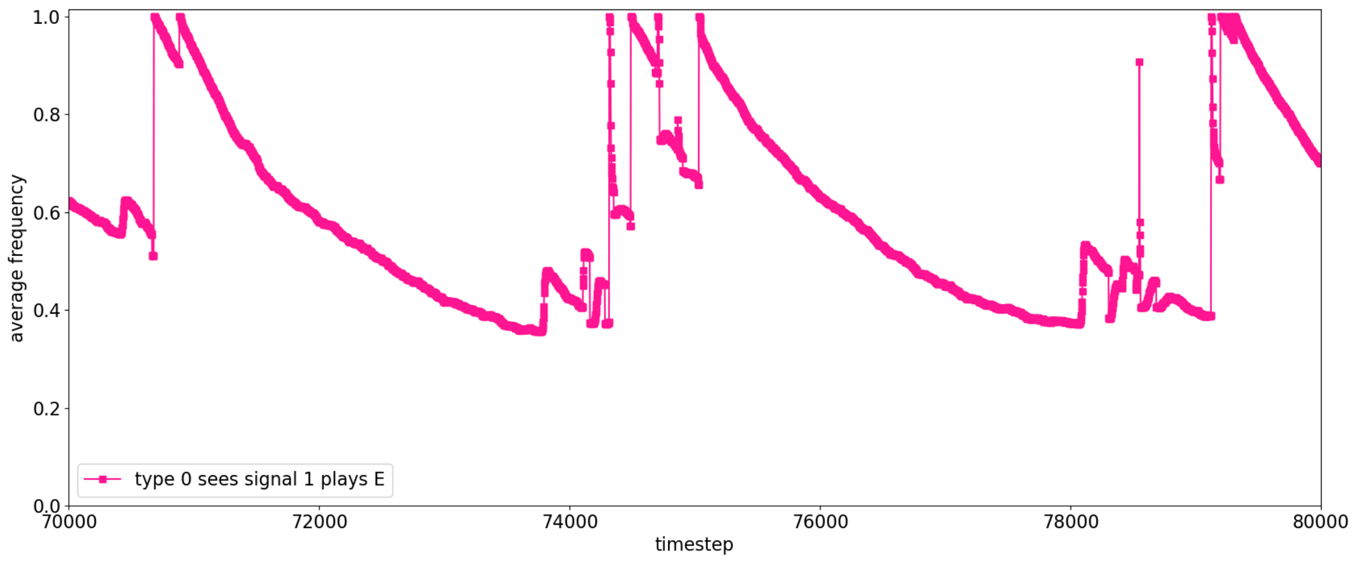
1.0
0.8
0.6
0.4
0.2
0.0
average frequency
type 0 sees signal 1 plays E
70000
72000
74000
76000
78000
80000
timestep

Run # 3
Type 0 signaling 0 vs signaling 1 fluctuations continue

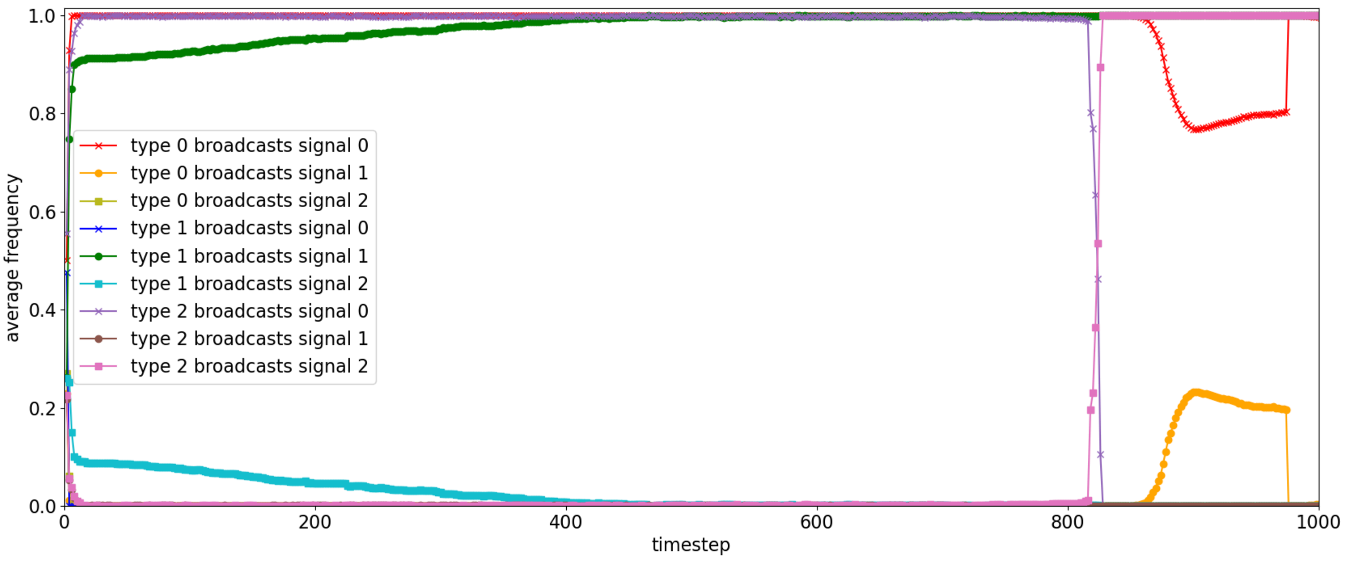

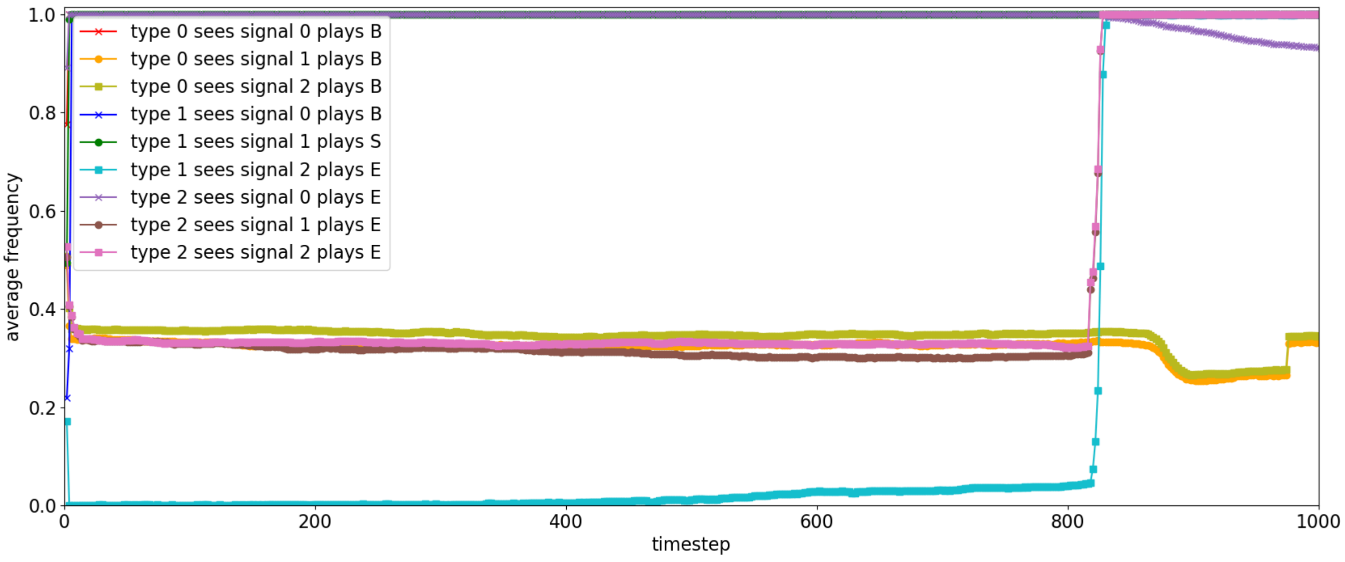

Run # 7

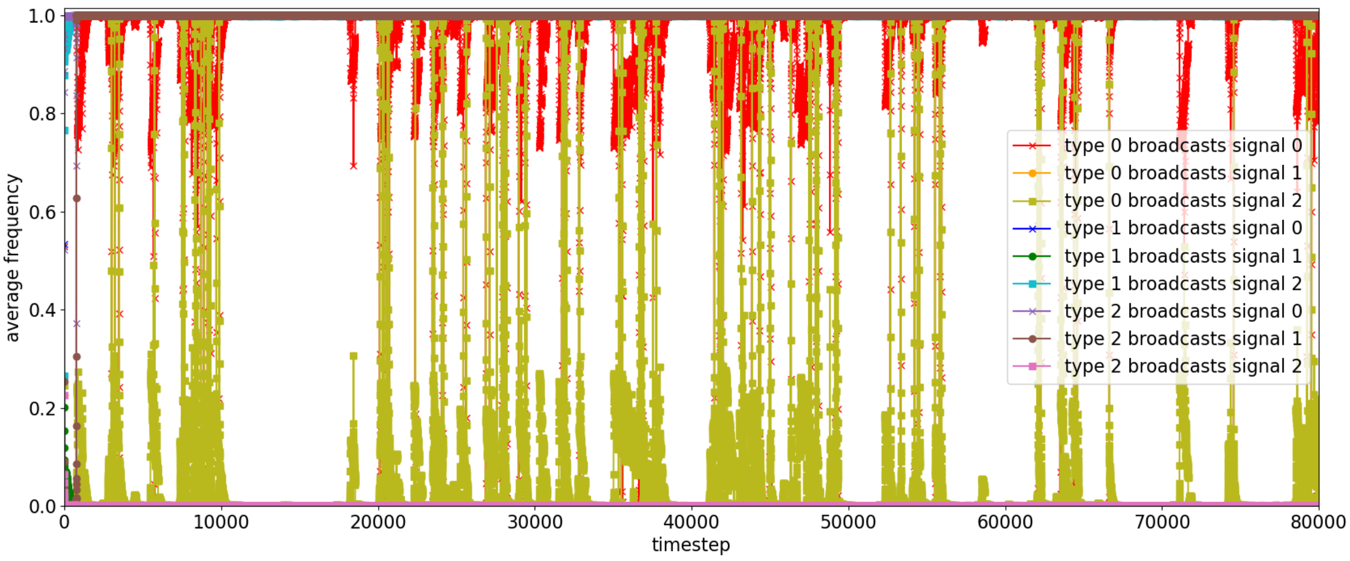
average frequency
timestep
type 0 broadcasts signal 0
type 0 broadcasts signal 1
type 0 broadcasts signal 2
type 1 broadcasts signal 0
type 1 broadcasts signal 1
type 1 broadcasts signal 2
type 2 broadcasts signal 0
type 2 broadcasts signal 1
type 2 broadcasts signal 2

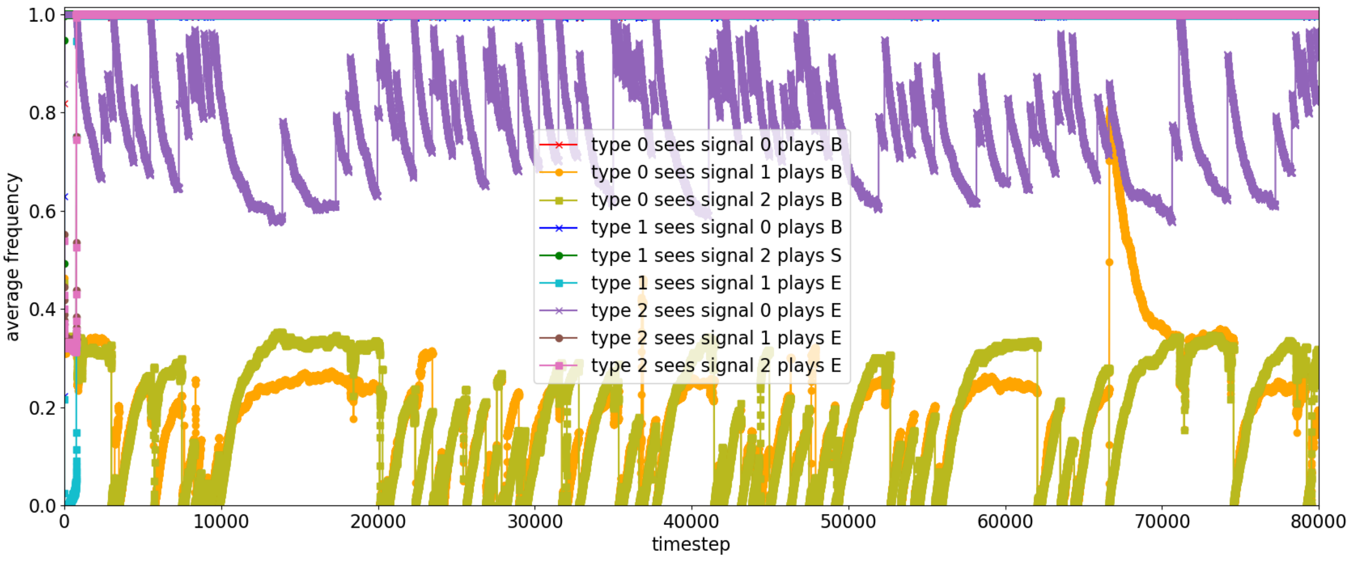
average frequency
timestep
type 0 sees signal 0 plays B
type 0 sees signal 1 plays B
type 0 sees signal 2 plays B
type 1 sees signal 0 plays B
type 1 sees signal 2 plays S
type 1 sees signal 1 plays E
type 2 sees signal 0 plays E
type 2 sees signal 1 plays E
type 2 sees signal 2 plays E

Run # 8

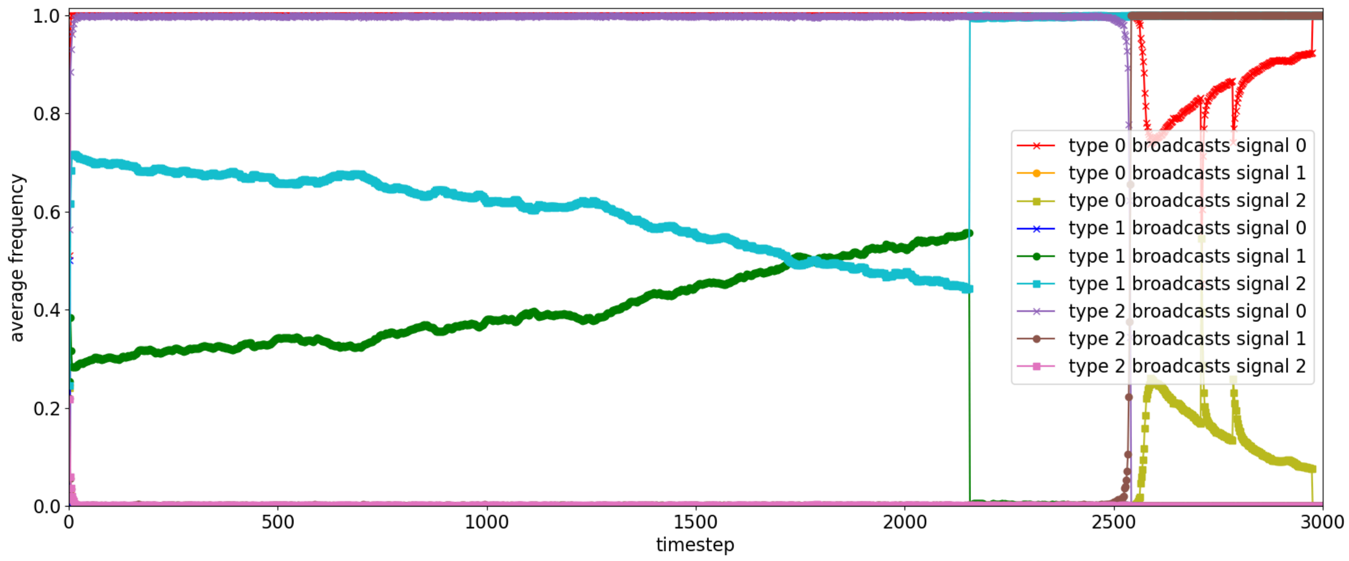

average frequency
timestep
type 0 broadcasts signal 0
type 0 broadcasts signal 1
type 0 broadcasts signal 2
type 1 broadcasts signal 0
type 1 broadcasts signal 1
type 1 broadcasts signal 2
type 2 broadcasts signal 0
type 2 broadcasts signal 1
type 2 broadcasts signal 2

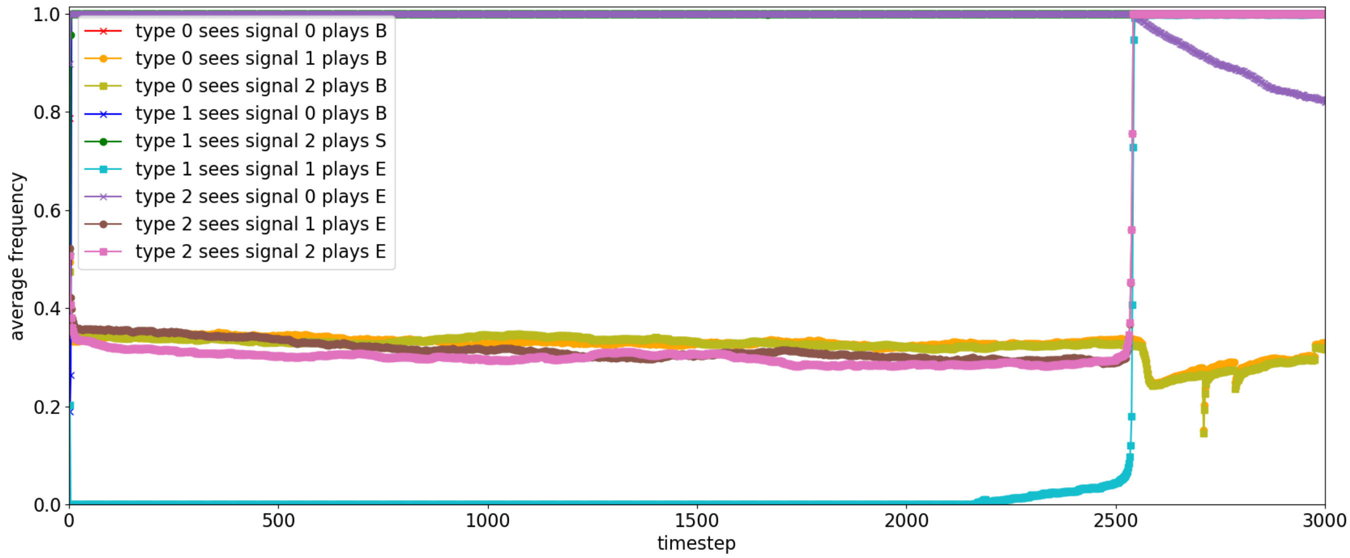

average frequency
timestep
type 0 sees signal 0 plays B
type 0 sees signal 1 plays B
type 0 sees signal 2 plays B
type 1 sees signal 0 plays B
type 1 sees signal 2 plays S
type 1 sees signal 1 plays E
type 2 sees signal 0 plays E
type 2 sees signal 1 plays E
type 2 sees signal 2 plays E

Run # 11

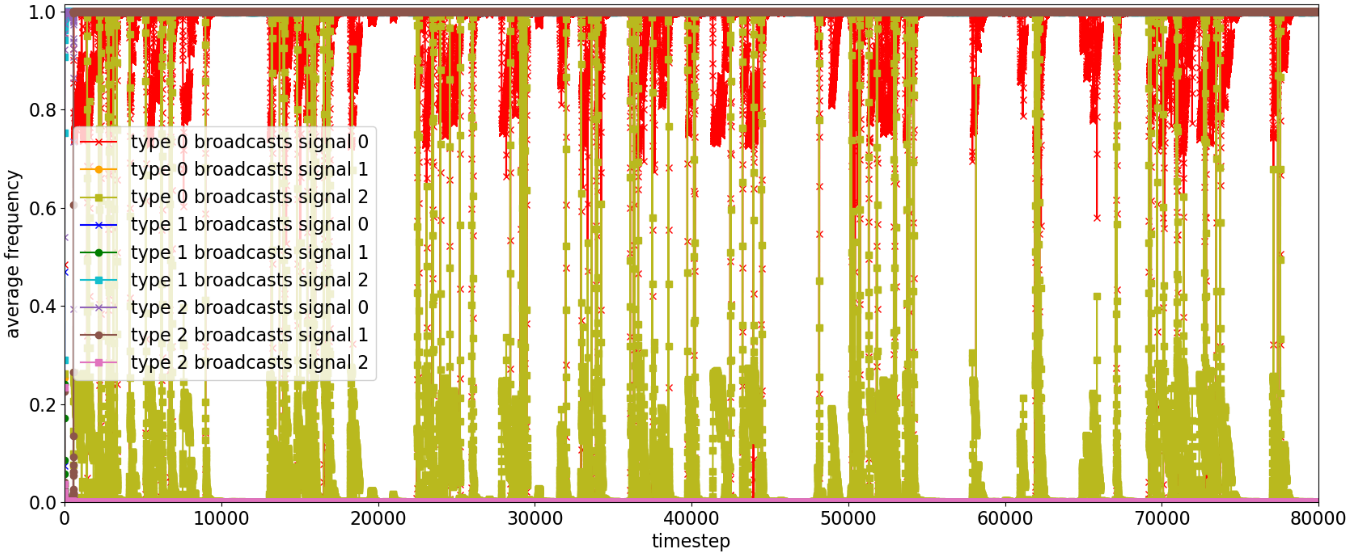

average frequency
timestep
type 0 broadcasts signal 0
type 0 broadcasts signal 1
type 0 broadcasts signal 2
type 1 broadcasts signal 0
type 1 broadcasts signal 1
type 1 broadcasts signal 2
type 2 broadcasts signal 0
type 2 broadcasts signal 1
type 2 broadcasts signal 2

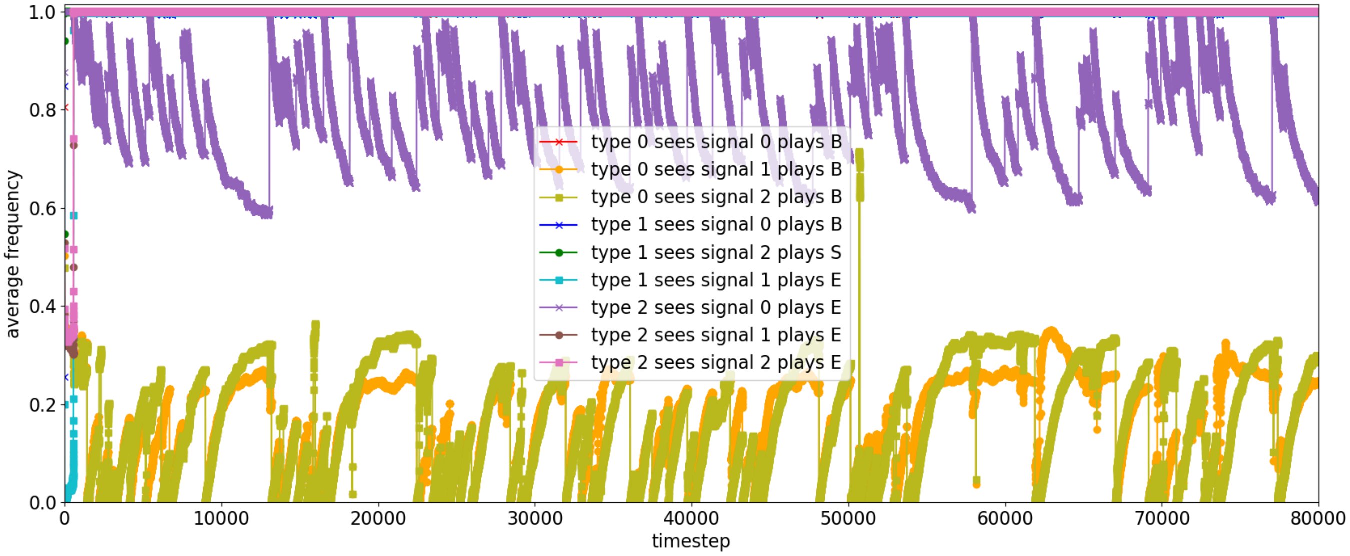

average frequency
timestep
type 0 sees signal 0 plays B
type 0 sees signal 1 plays B
type 0 sees signal 2 plays B
type 1 sees signal 0 plays B
type 1 sees signal 2 plays S
type 1 sees signal 1 plays E
type 2 sees signal 0 plays E
type 2 sees signal 1 plays E
type 2 sees signal 2 plays E

Run # 13
Serious fight between types 0 and 1 for what to do on signal 0

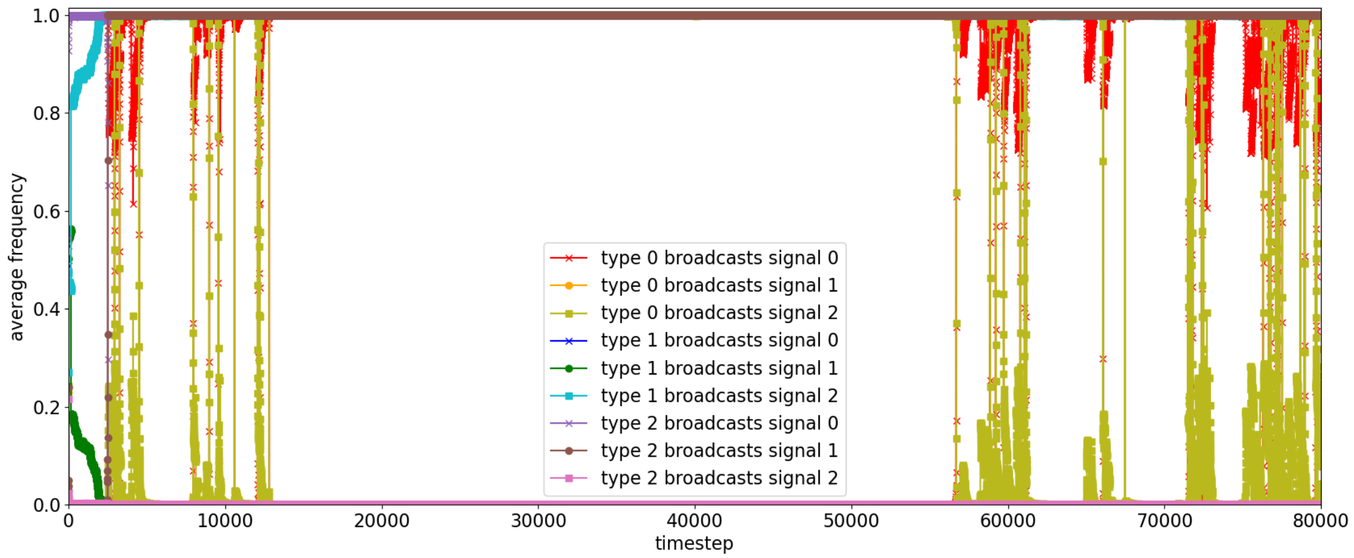

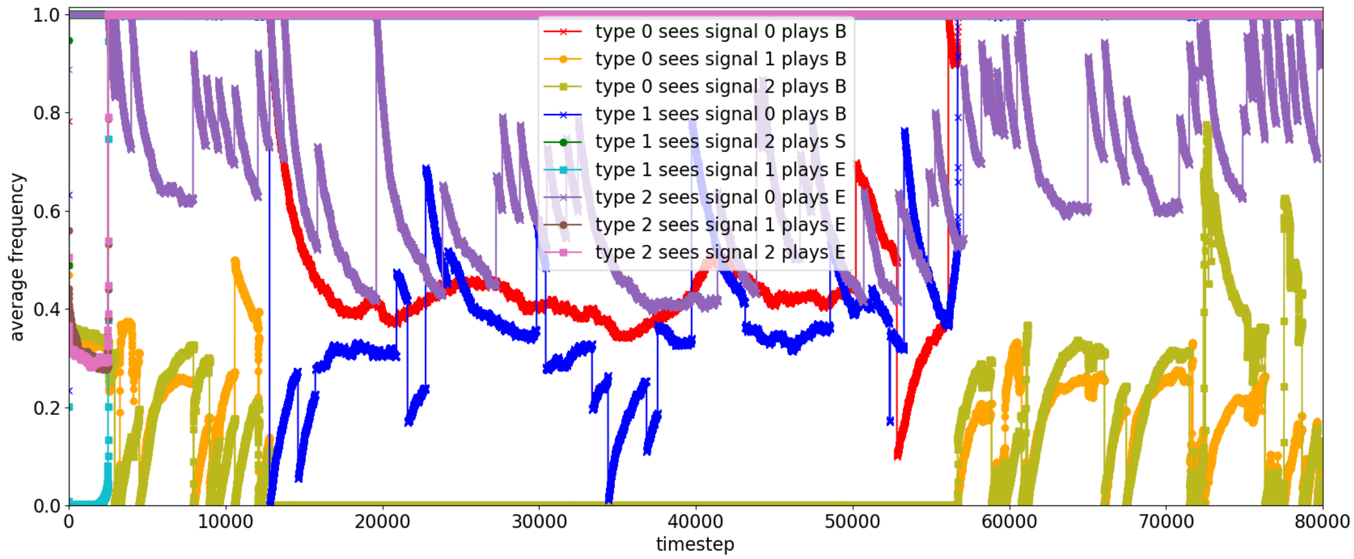

Run # 13 continued on next page

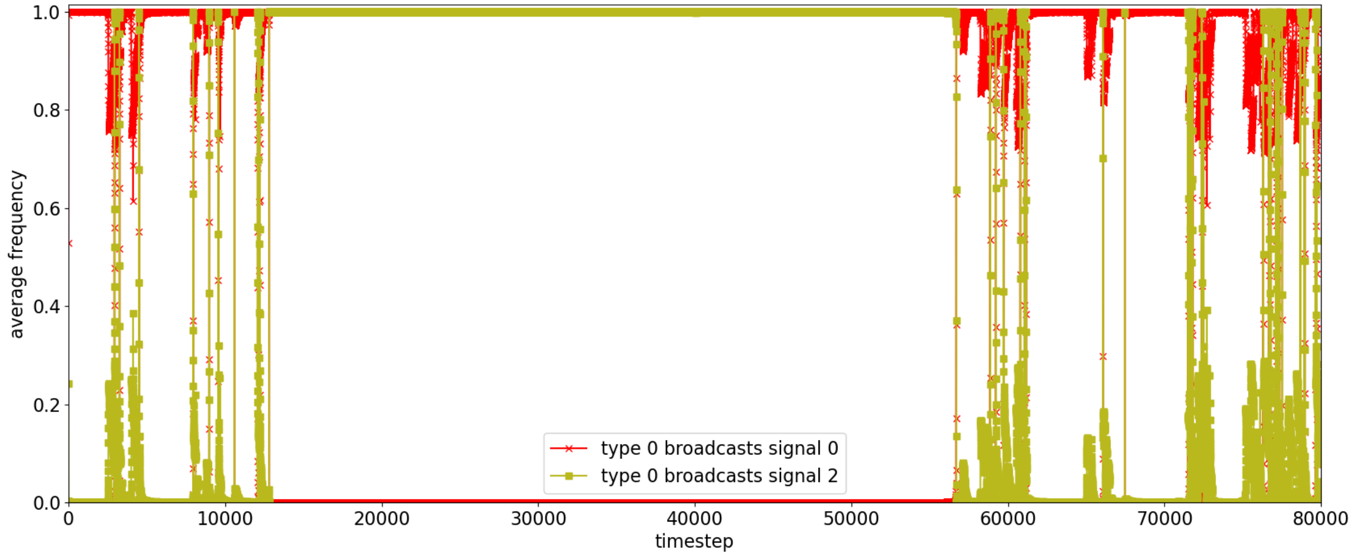

1.0
0.8
0.6
0.4
0.2
0.0
average frequency
0
10000
20000
30000
40000
50000
60000
70000
80000
timestep
type 0 broadcasts signal 0
type 0 broadcasts signal 2

Run # 14

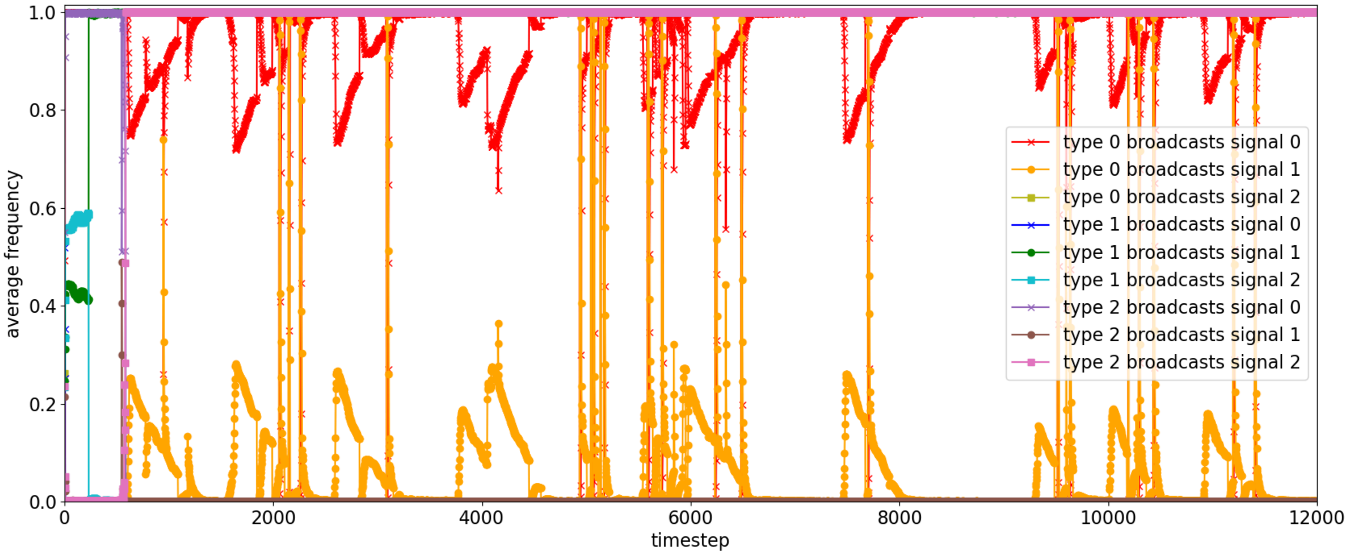
average frequency
timestep
type 0 broadcasts signal 0
type 0 broadcasts signal 1
type 0 broadcasts signal 2
type 1 broadcasts signal 0
type 1 broadcasts signal 1
type 1 broadcasts signal 2
type 2 broadcasts signal 0
type 2 broadcasts signal 1
type 2 broadcasts signal 2

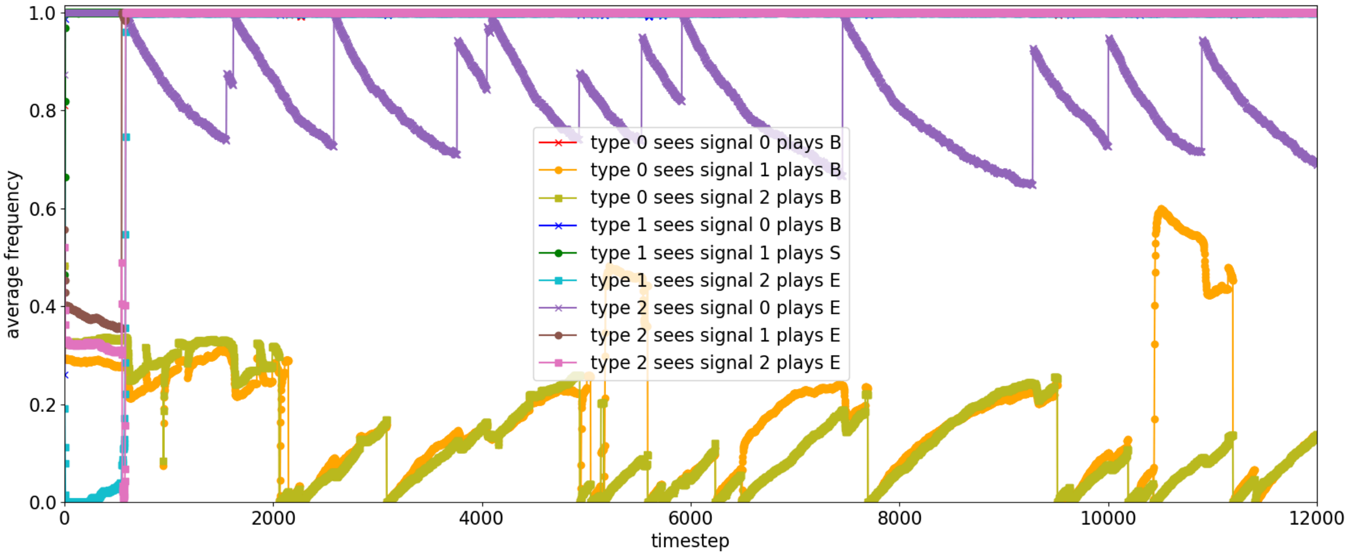
average frequency
timestep
type 0 sees signal 0 plays B
type 0 sees signal 1 plays B
type 0 sees signal 2 plays B
type 1 sees signal 0 plays B
type 1 sees signal 1 plays S
type 1 sees signal 2 plays E
type 2 sees signal 0 plays E
type 2 sees signal 1 plays E
type 2 sees signal 2 plays E

Run # 16

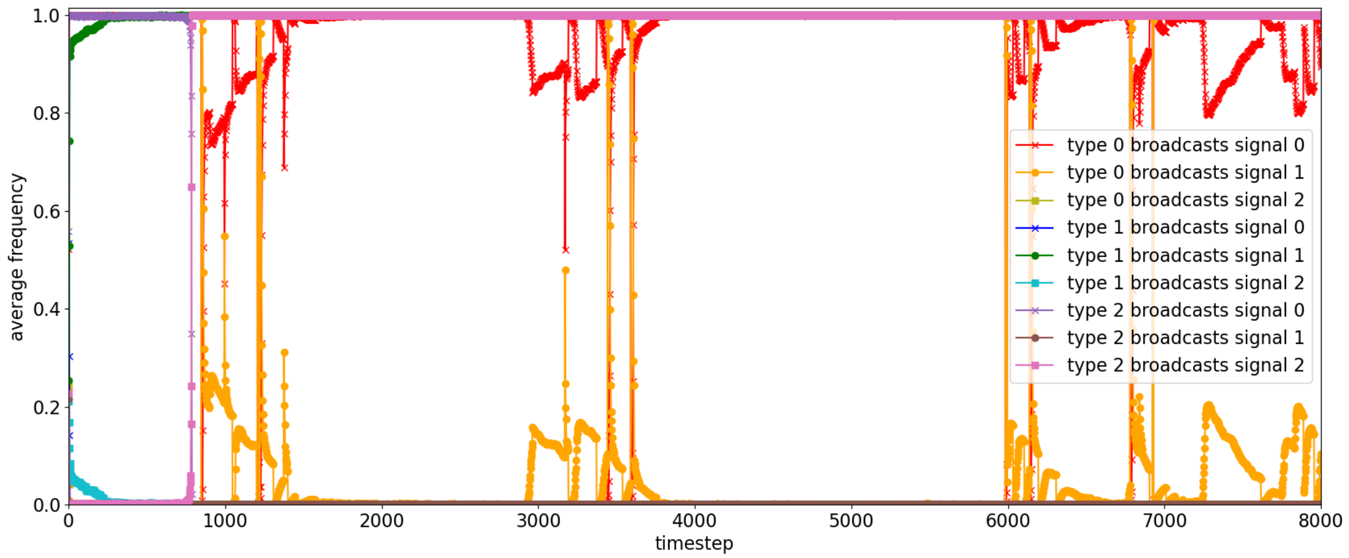
average frequency
timestep
type 0 broadcasts signal 0
type 0 broadcasts signal 1
type 0 broadcasts signal 2
type 1 broadcasts signal 0
type 1 broadcasts signal 1
type 1 broadcasts signal 2
type 2 broadcasts signal 0
type 2 broadcasts signal 1
type 2 broadcasts signal 2

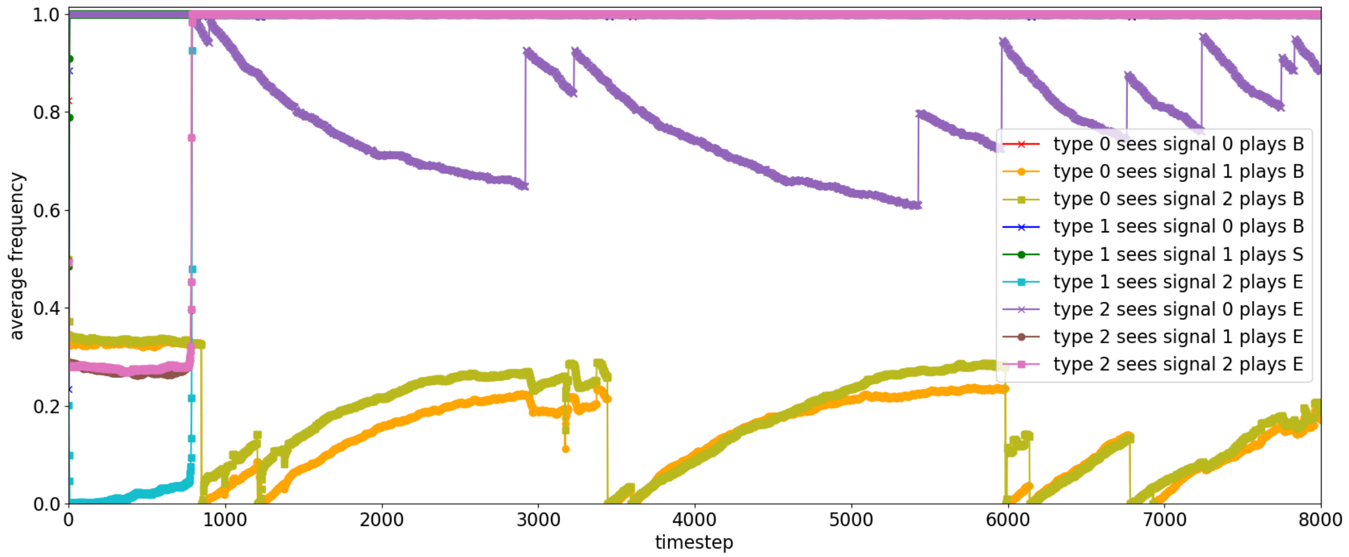
average frequency
timestep
type 0 sees signal 0 plays B
type 0 sees signal 1 plays B
type 0 sees signal 2 plays B
type 1 sees signal 0 plays B
type 1 sees signal 1 plays S
type 1 sees signal 2 plays E
type 2 sees signal 0 plays E
type 2 sees signal 1 plays E
type 2 sees signal 2 plays E

Run # 18

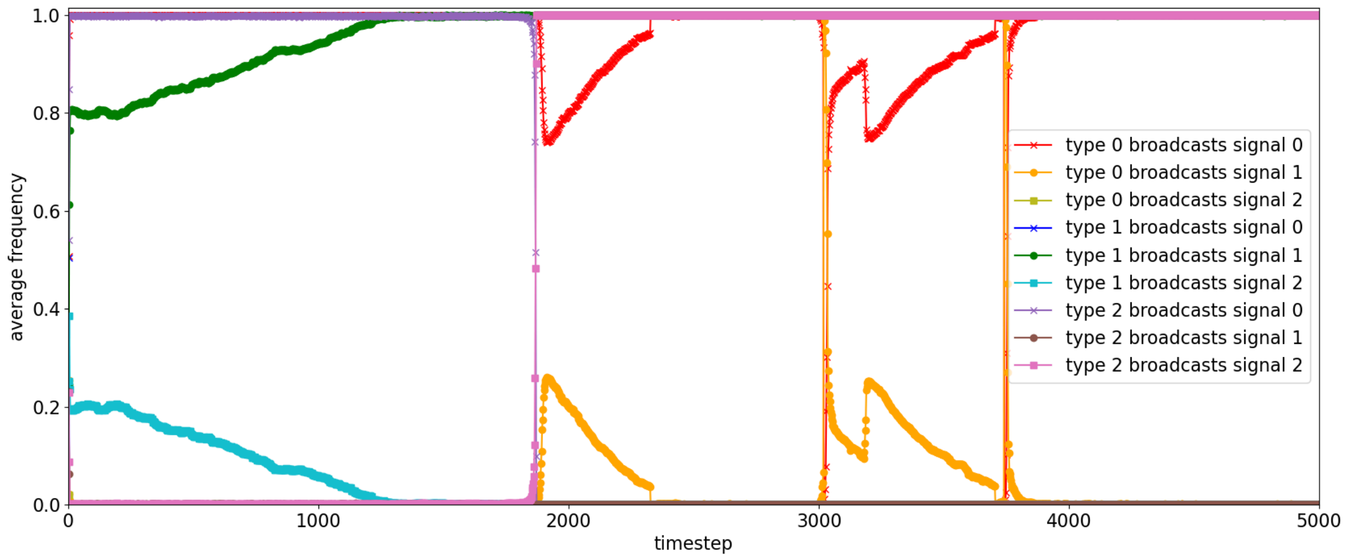
average frequency
timestep
type 0 broadcasts signal 0
type 0 broadcasts signal 1
type 0 broadcasts signal 2
type 1 broadcasts signal 0
type 1 broadcasts signal 1
type 1 broadcasts signal 2
type 2 broadcasts signal 0
type 2 broadcasts signal 1
type 2 broadcasts signal 2

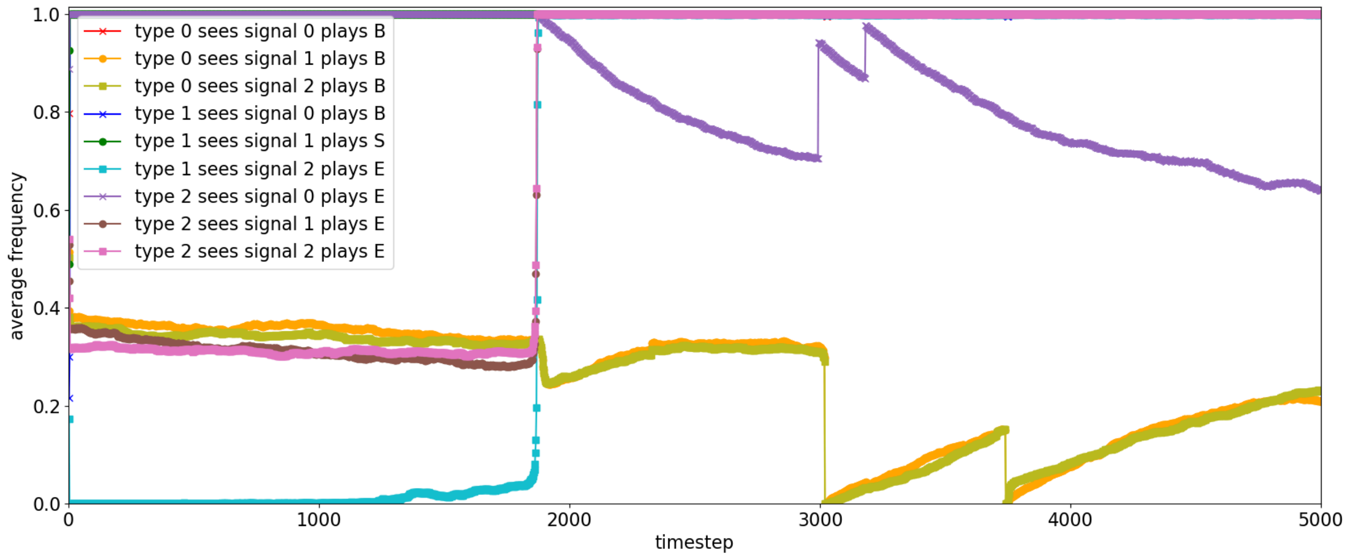
average frequency
timestep
type 0 sees signal 0 plays B
type 0 sees signal 1 plays B
type 0 sees signal 2 plays B
type 1 sees signal 0 plays B
type 1 sees signal 1 plays S
type 1 sees signal 2 plays E
type 2 sees signal 0 plays E
type 2 sees signal 1 plays E
type 2 sees signal 2 plays E

outcomes (ix): characterized by type 1 signaling 0

Run # 92

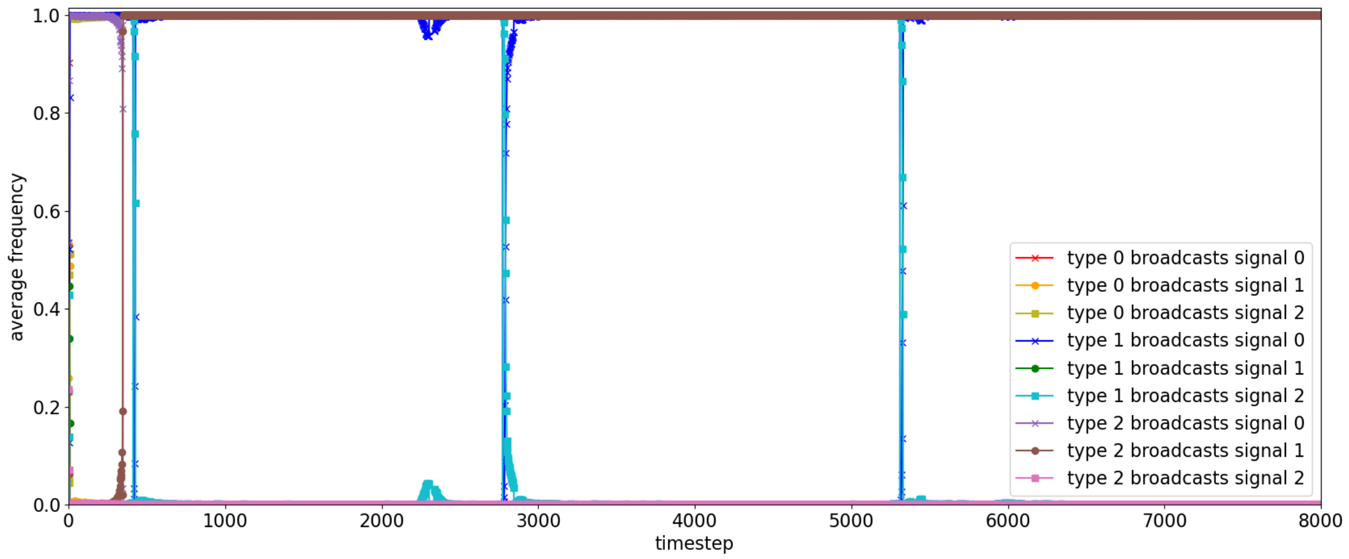

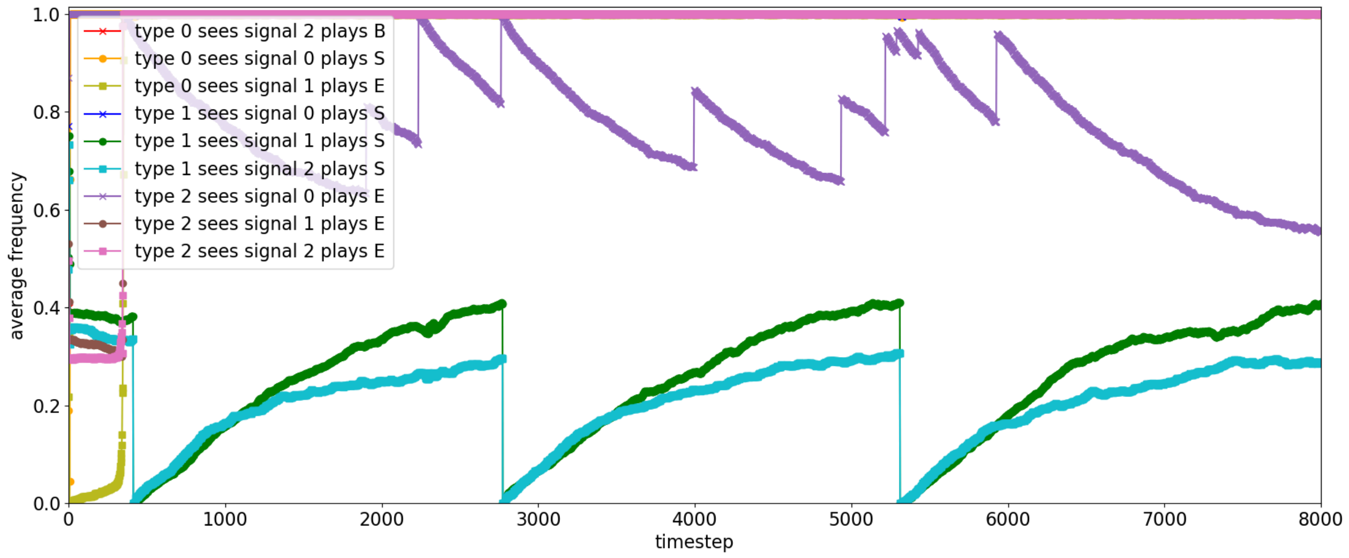

Run # 206

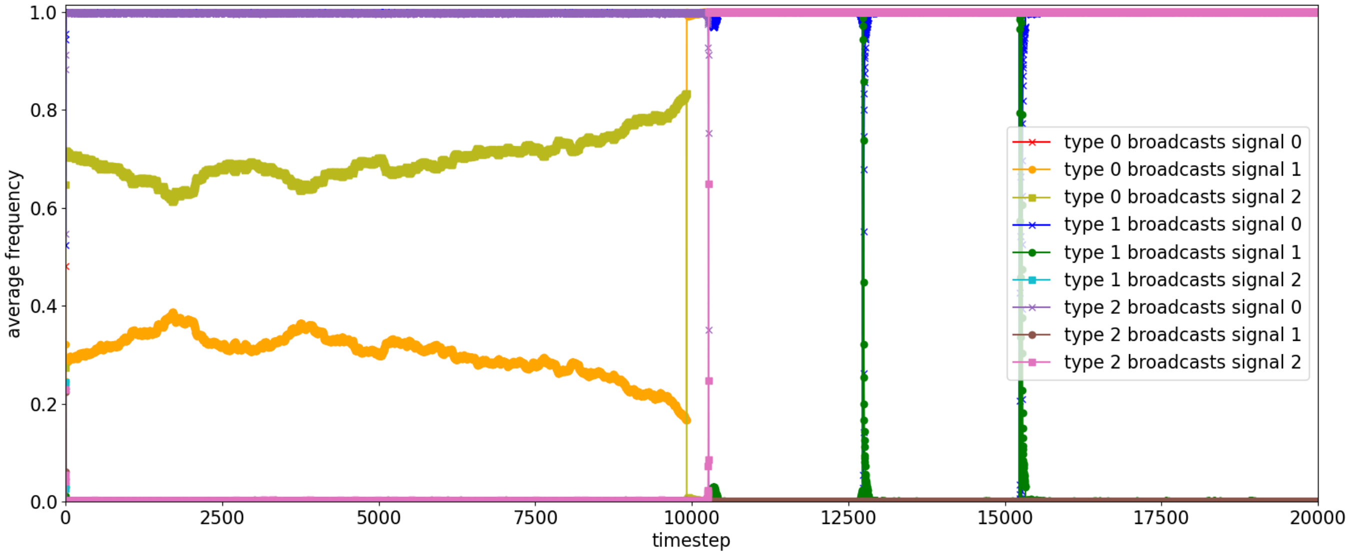
average frequency
timestep
type 0 broadcasts signal 0
type 0 broadcasts signal 1
type 0 broadcasts signal 2
type 1 broadcasts signal 0
type 1 broadcasts signal 1
type 1 broadcasts signal 2
type 2 broadcasts signal 0
type 2 broadcasts signal 1
type 2 broadcasts signal 2

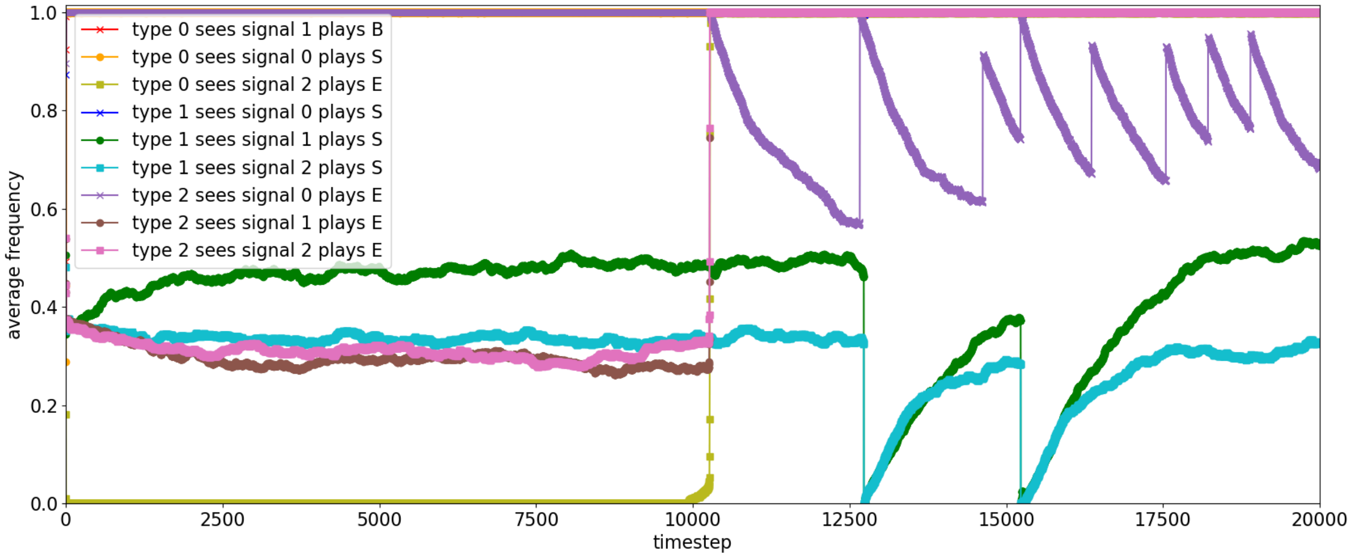
average frequency
timestep
type 0 sees signal 1 plays B
type 0 sees signal 0 plays S
type 0 sees signal 2 plays E
type 1 sees signal 0 plays S
type 1 sees signal 1 plays S
type 1 sees signal 2 plays S
type 2 sees signal 0 plays E
type 2 sees signal 1 plays E
type 2 sees signal 2 plays E

Run # 637

Like other runs with this outcome there are continued transient spikes of type 1 signaling 1 rather than 0

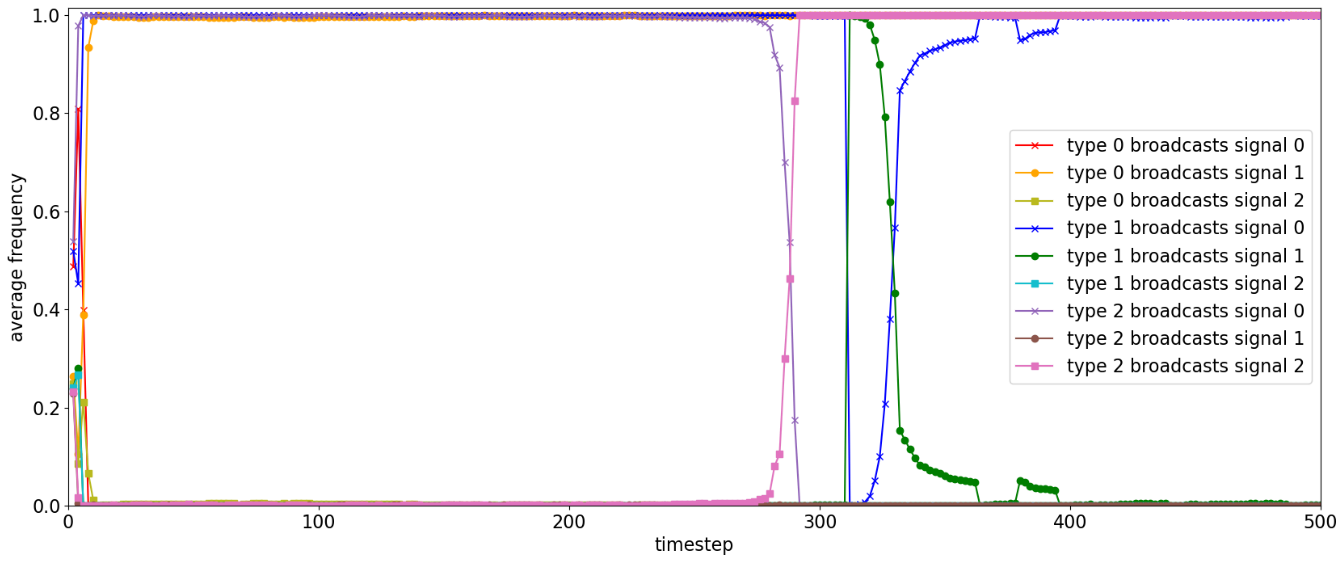

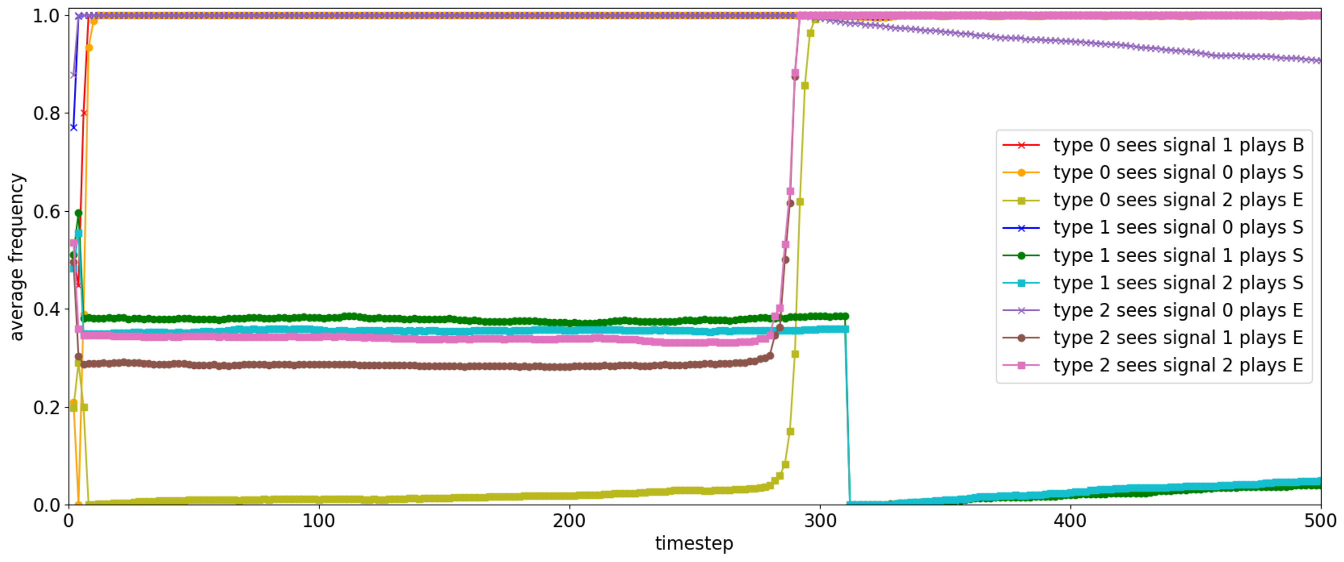

outcomes (x):

Run # 9

[[[0 403 3297]
[0 2900 0]
[3400 0 0]]]

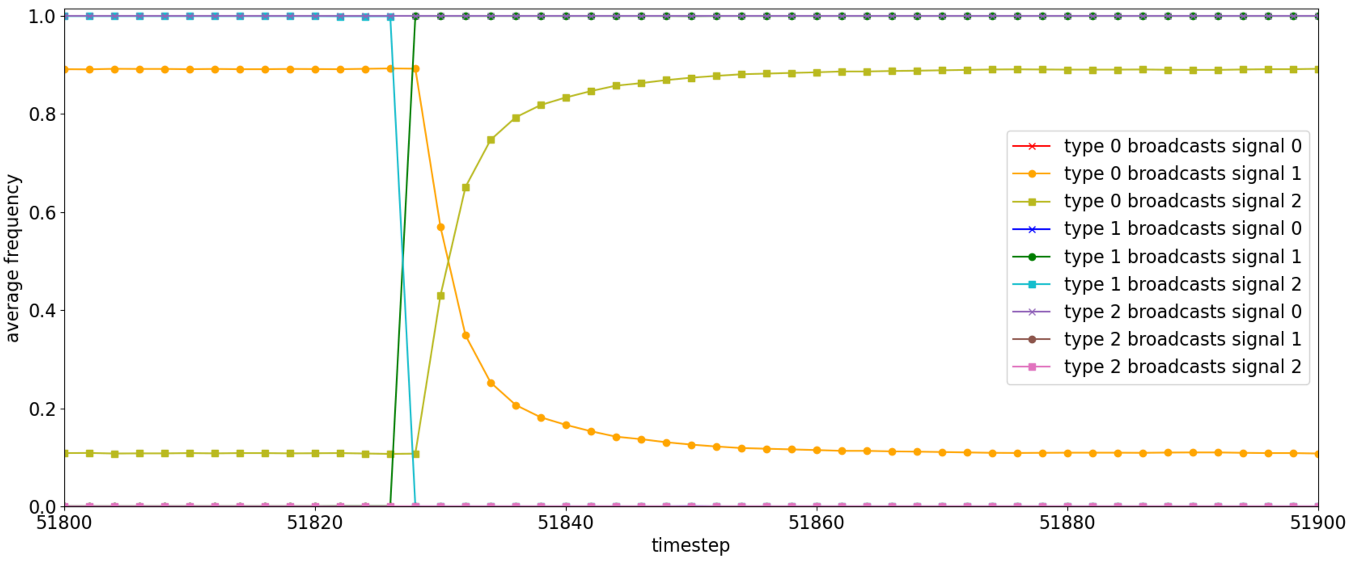

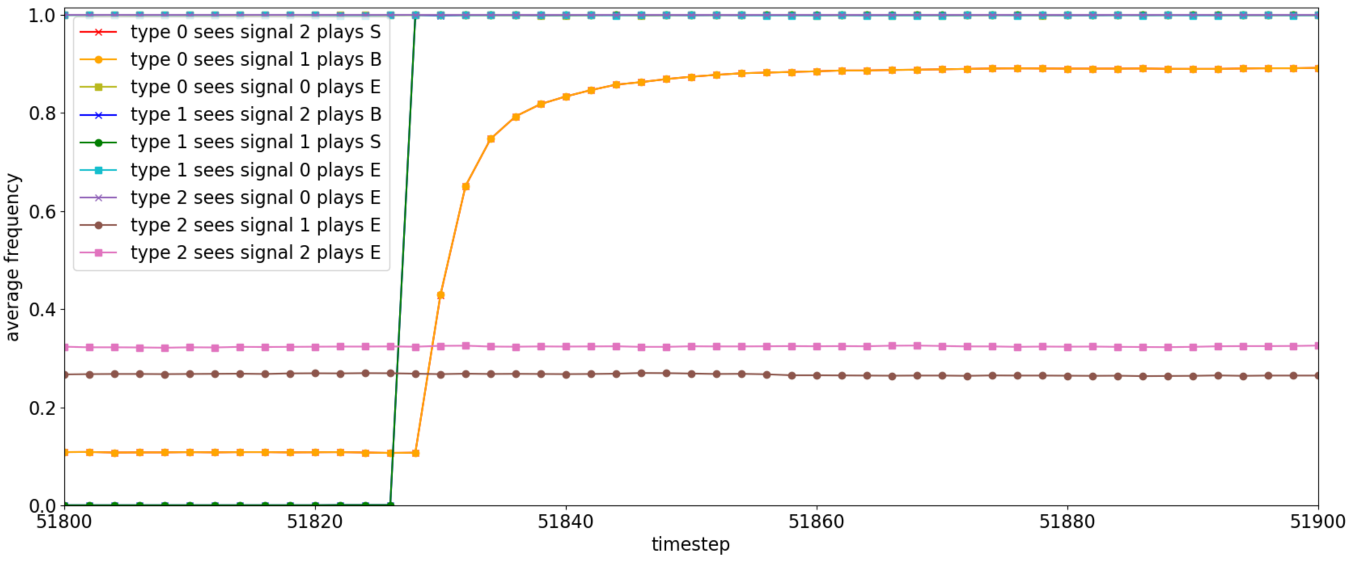

outcomes (xi): looks like the profile that has type 1 playing S with 0 is same one that has 1 playing B with 1, so the dispositions invade together

Run # 273

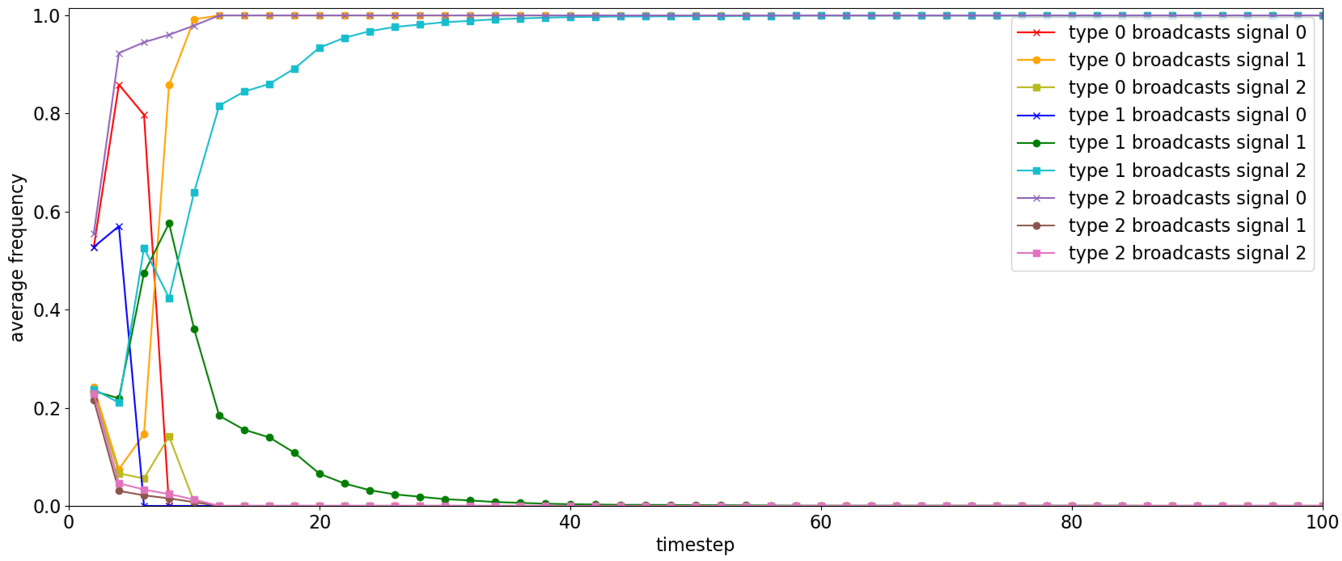

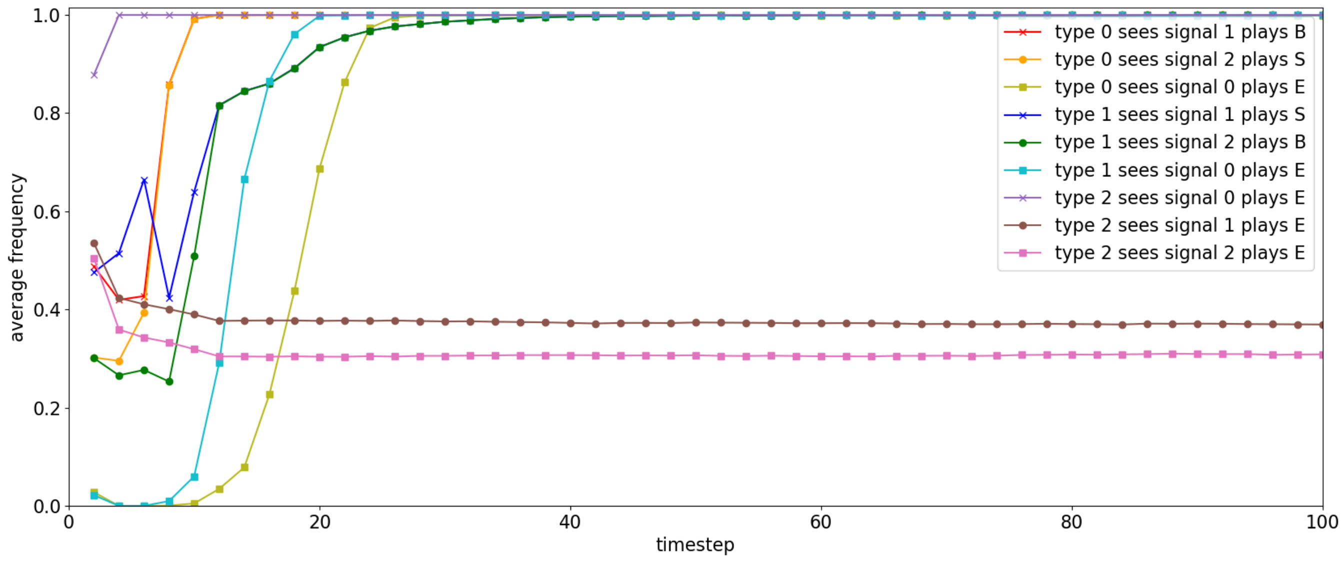

Run # 443

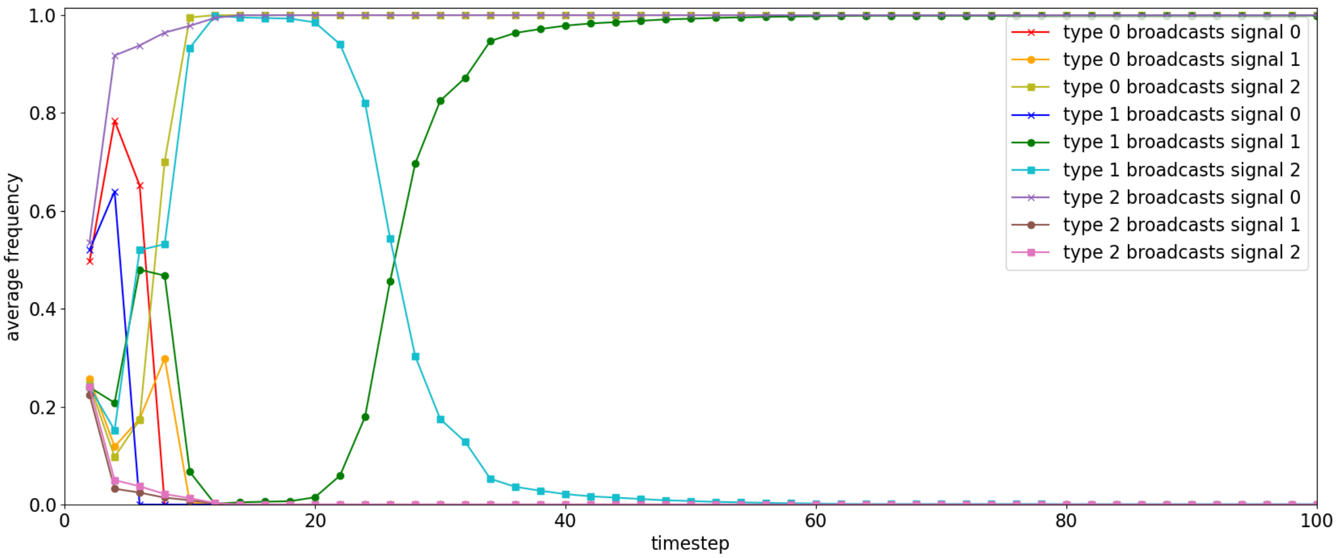
average frequency
timestep
type 0 broadcasts signal 0
type 0 broadcasts signal 1
type 0 broadcasts signal 2
type 1 broadcasts signal 0
type 1 broadcasts signal 1
type 1 broadcasts signal 2
type 2 broadcasts signal 0
type 2 broadcasts signal 1
type 2 broadcasts signal 2

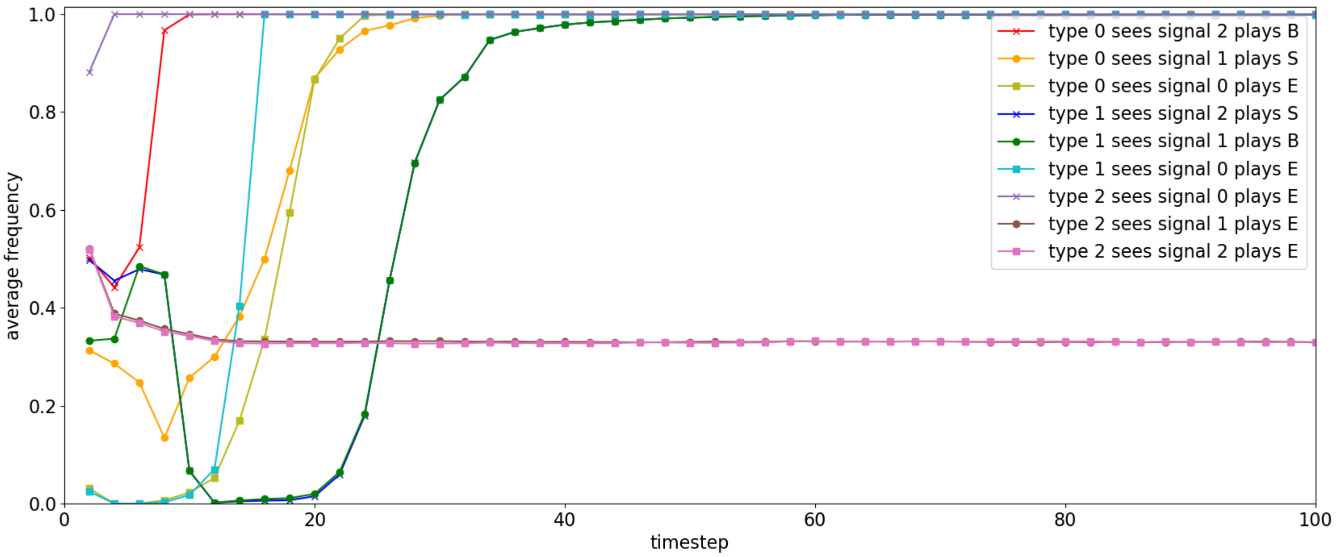
average frequency
timestep
type 0 sees signal 2 plays B
type 0 sees signal 1 plays S
type 0 sees signal 0 plays E
type 1 sees signal 2 plays S
type 1 sees signal 1 plays B
type 1 sees signal 0 plays E
type 2 sees signal 0 plays E
type 2 sees signal 1 plays E
type 2 sees signal 2 plays E

Run # 508

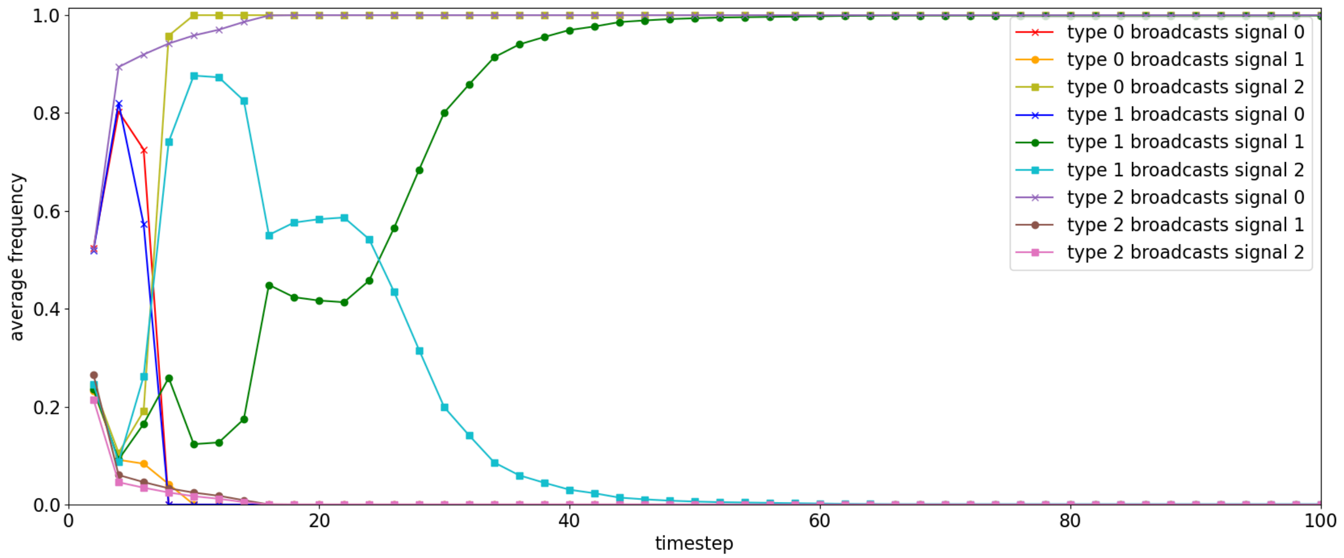
average frequency
timestep
type 0 broadcasts signal 0
type 0 broadcasts signal 1
type 0 broadcasts signal 2
type 1 broadcasts signal 0
type 1 broadcasts signal 1
type 1 broadcasts signal 2
type 2 broadcasts signal 0
type 2 broadcasts signal 1
type 2 broadcasts signal 2

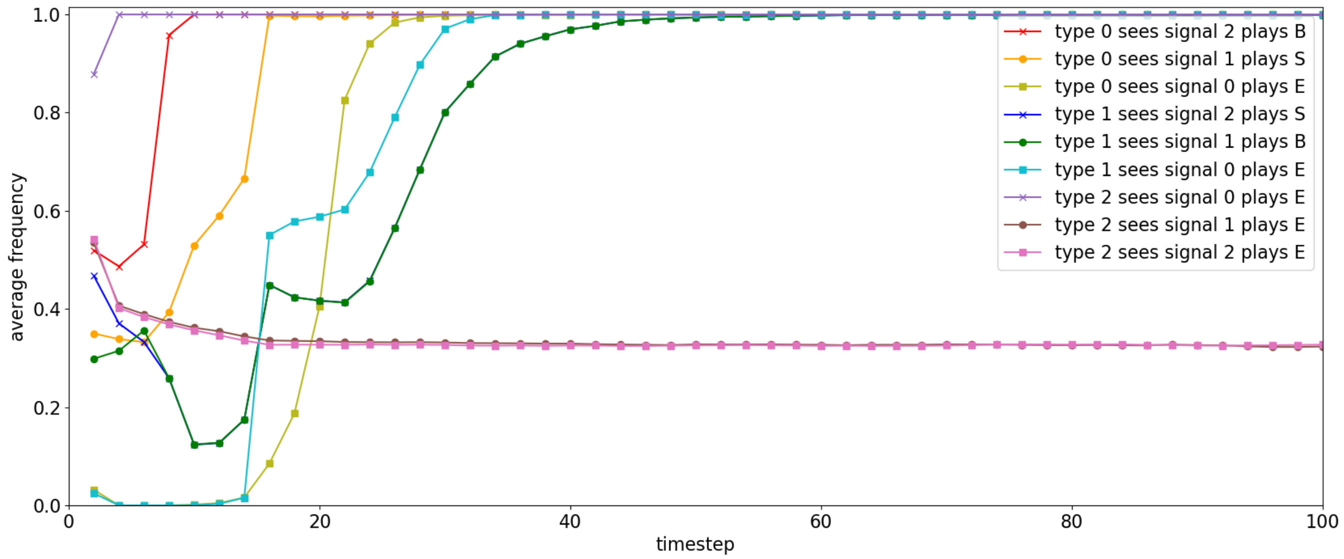
average frequency
timestep
type 0 sees signal 2 plays B
type 0 sees signal 1 plays S
type 0 sees signal 0 plays E
type 1 sees signal 2 plays S
type 1 sees signal 1 plays B
type 1 sees signal 0 plays E
type 2 sees signal 0 plays E
type 2 sees signal 1 plays E
type 2 sees signal 2 plays E

Run # 696

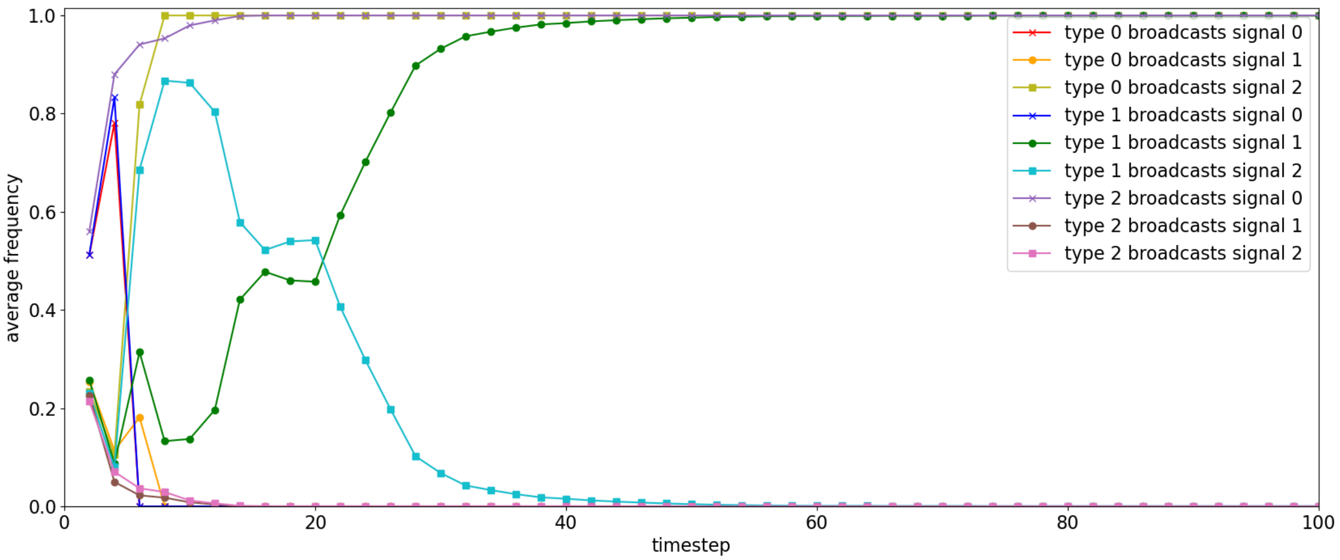
average frequency
timestep
0.0
0.2
0.4
0.6
0.8
1.0
0
20
40
60
80
100
type 0 broadcasts signal 0
type 0 broadcasts signal 1
type 0 broadcasts signal 2
type 1 broadcasts signal 0
type 1 broadcasts signal 1
type 1 broadcasts signal 2
type 2 broadcasts signal 0
type 2 broadcasts signal 1
type 2 broadcasts signal 2

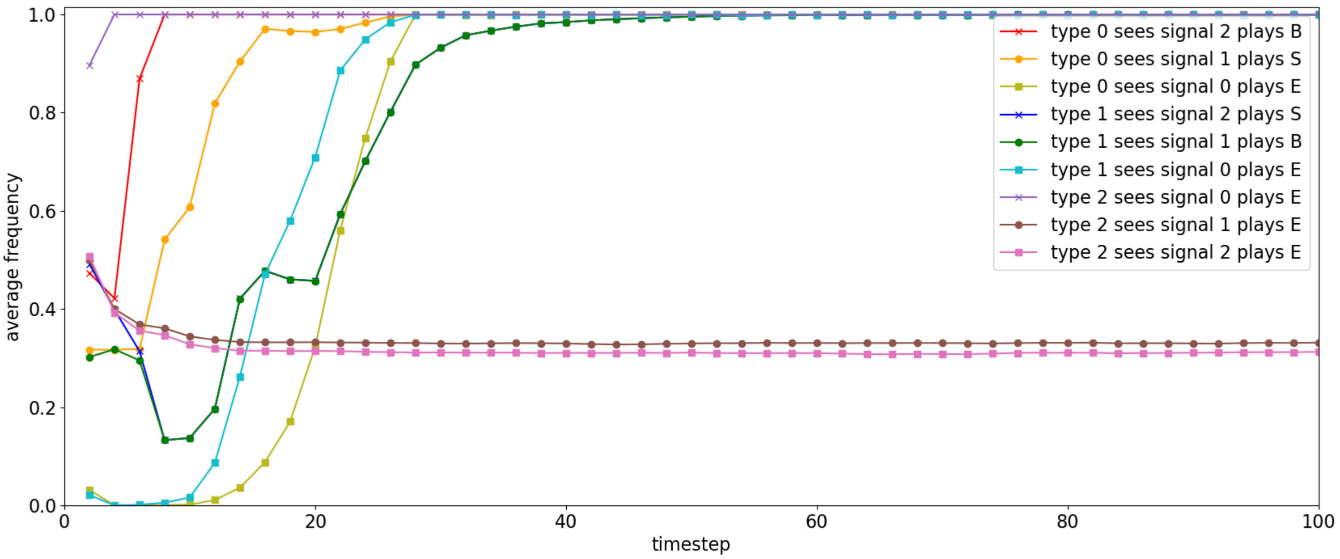
average frequency
timestep
0.0
0.2
0.4
0.6
0.8
1.0
0
20
40
60
80
100
type 0 sees signal 2 plays B
type 0 sees signal 1 plays S
type 0 sees signal 0 plays E
type 1 sees signal 2 plays S
type 1 sees signal 1 plays B
type 1 sees signal 0 plays E
type 2 sees signal 0 plays E
type 2 sees signal 1 plays E
type 2 sees signal 2 plays E

Run # 855

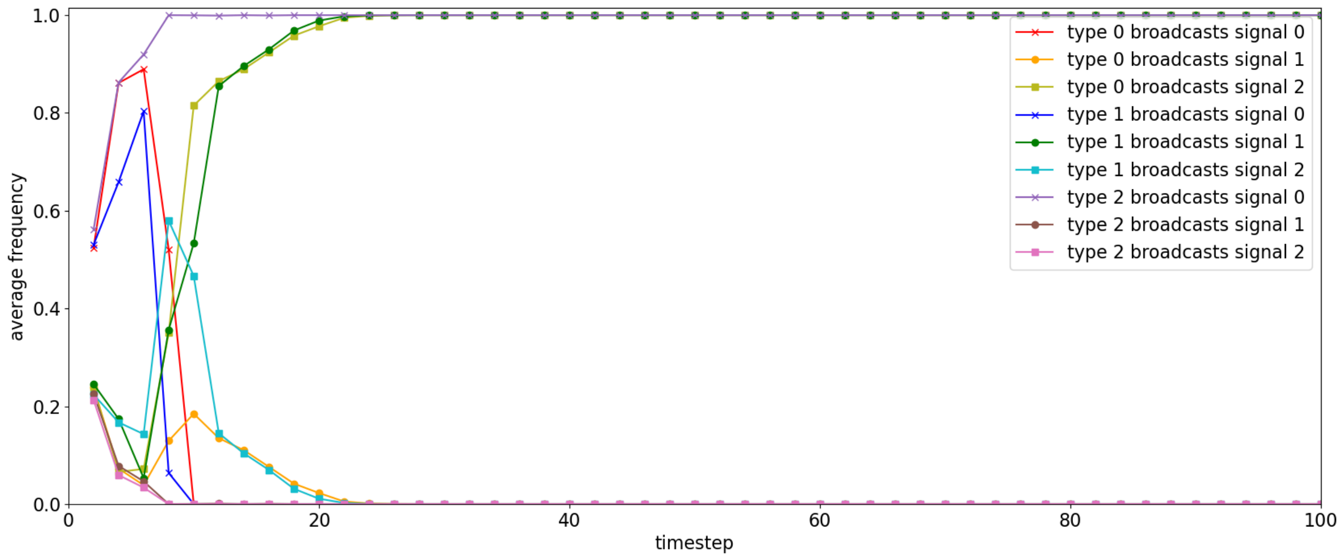
average frequency
timestep
0.0
0.2
0.4
0.6
0.8
1.0
0
20
40
60
80
100
type 0 broadcasts signal 0
type 0 broadcasts signal 1
type 0 broadcasts signal 2
type 1 broadcasts signal 0
type 1 broadcasts signal 1
type 1 broadcasts signal 2
type 2 broadcasts signal 0
type 2 broadcasts signal 1
type 2 broadcasts signal 2

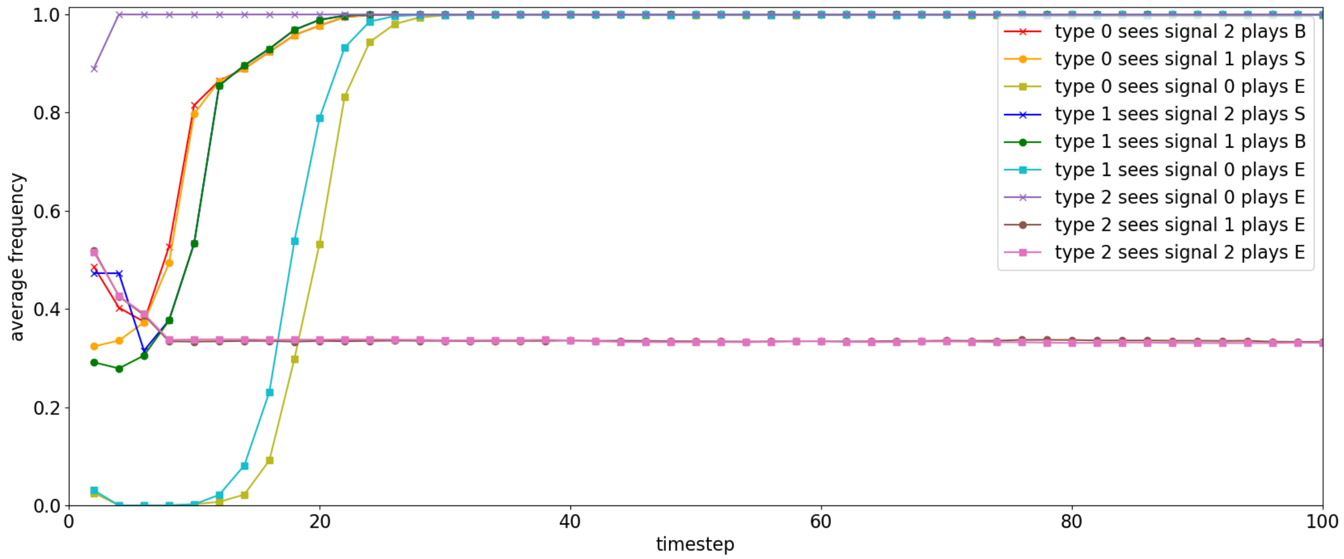
average frequency
timestep
0.0
0.2
0.4
0.6
0.8
1.0
0
20
40
60
80
100
type 0 sees signal 2 plays B
type 0 sees signal 1 plays S
type 0 sees signal 0 plays E
type 1 sees signal 2 plays S
type 1 sees signal 1 plays B
type 1 sees signal 0 plays E
type 2 sees signal 0 plays E
type 2 sees signal 1 plays E
type 2 sees signal 2 plays E

Run # 929

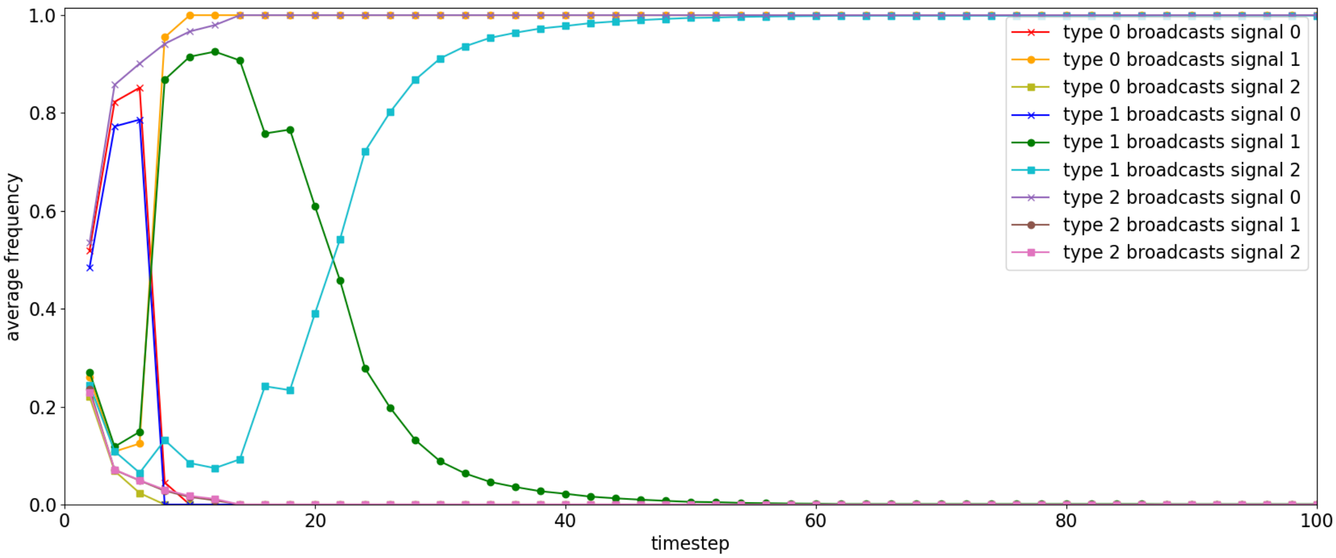
average frequency
timestep
type 0 broadcasts signal 0
type 0 broadcasts signal 1
type 0 broadcasts signal 2
type 1 broadcasts signal 0
type 1 broadcasts signal 1
type 1 broadcasts signal 2
type 2 broadcasts signal 0
type 2 broadcasts signal 1
type 2 broadcasts signal 2

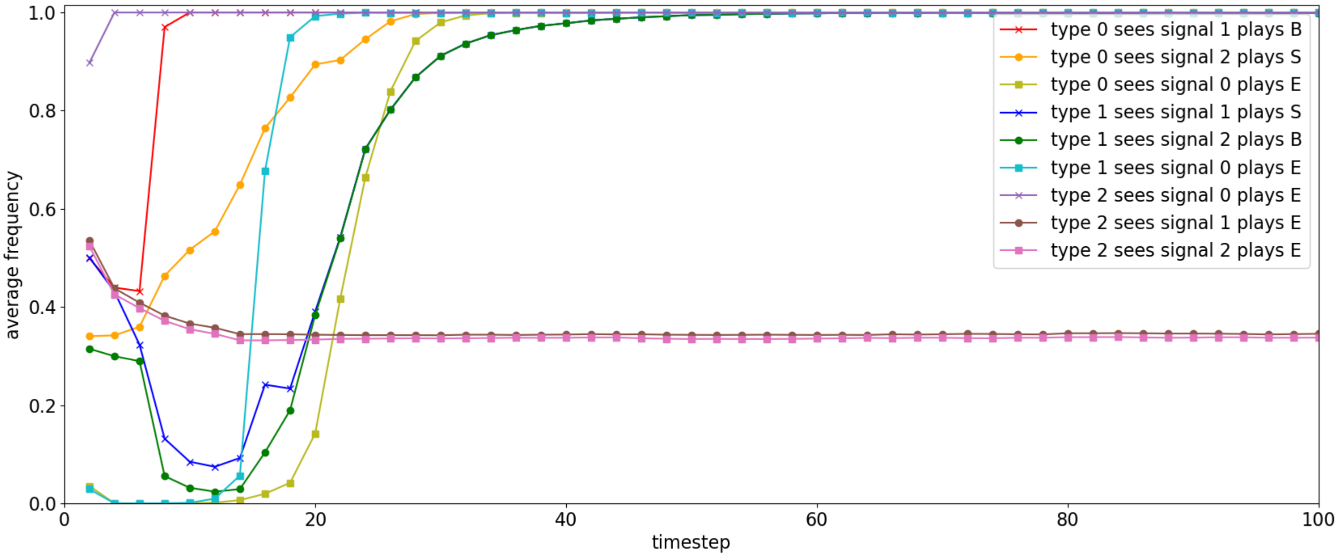
average frequency
timestep
type 0 sees signal 1 plays B
type 0 sees signal 2 plays S
type 0 sees signal 0 plays E
type 1 sees signal 1 plays S
type 1 sees signal 2 plays B
type 1 sees signal 0 plays E
type 2 sees signal 0 plays E
type 2 sees signal 1 plays E
type 2 sees signal 2 plays E

Run # 933

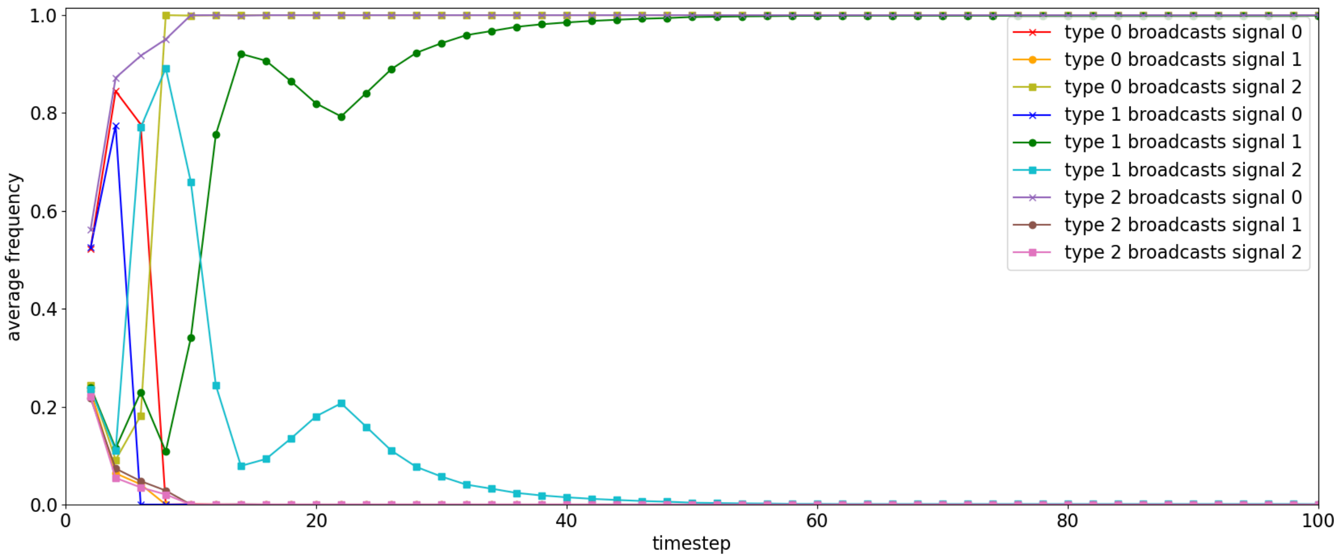
average frequency
timestep
type 0 broadcasts signal 0
type 0 broadcasts signal 1
type 0 broadcasts signal 2
type 1 broadcasts signal 0
type 1 broadcasts signal 1
type 1 broadcasts signal 2
type 2 broadcasts signal 0
type 2 broadcasts signal 1
type 2 broadcasts signal 2

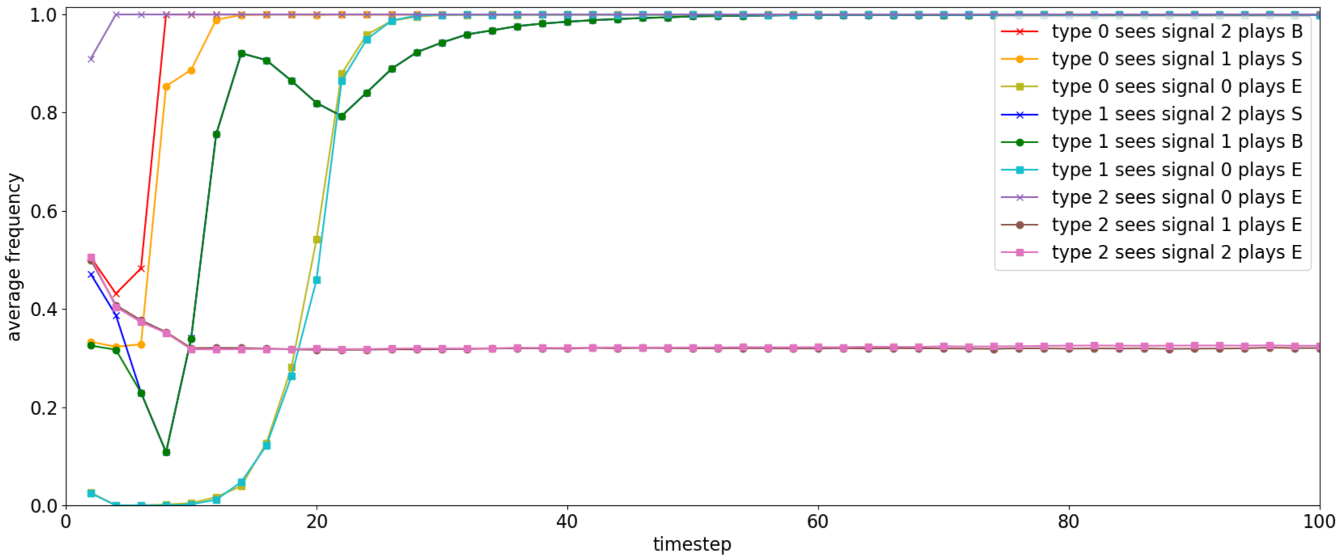
average frequency
timestep
type 0 sees signal 2 plays B
type 0 sees signal 1 plays S
type 0 sees signal 0 plays E
type 1 sees signal 2 plays S
type 1 sees signal 1 plays B
type 1 sees signal 0 plays E
type 2 sees signal 0 plays E
type 2 sees signal 1 plays E
type 2 sees signal 2 plays E

outcomes (xii): necessarily characterized by type 2 signaling 0
Run # 6

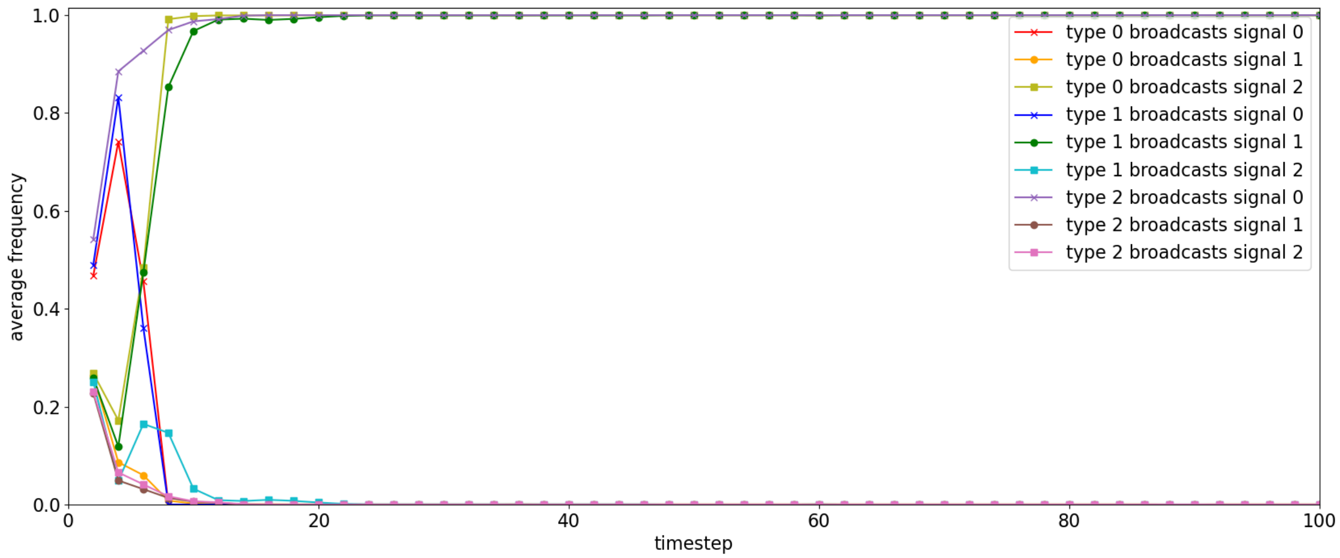

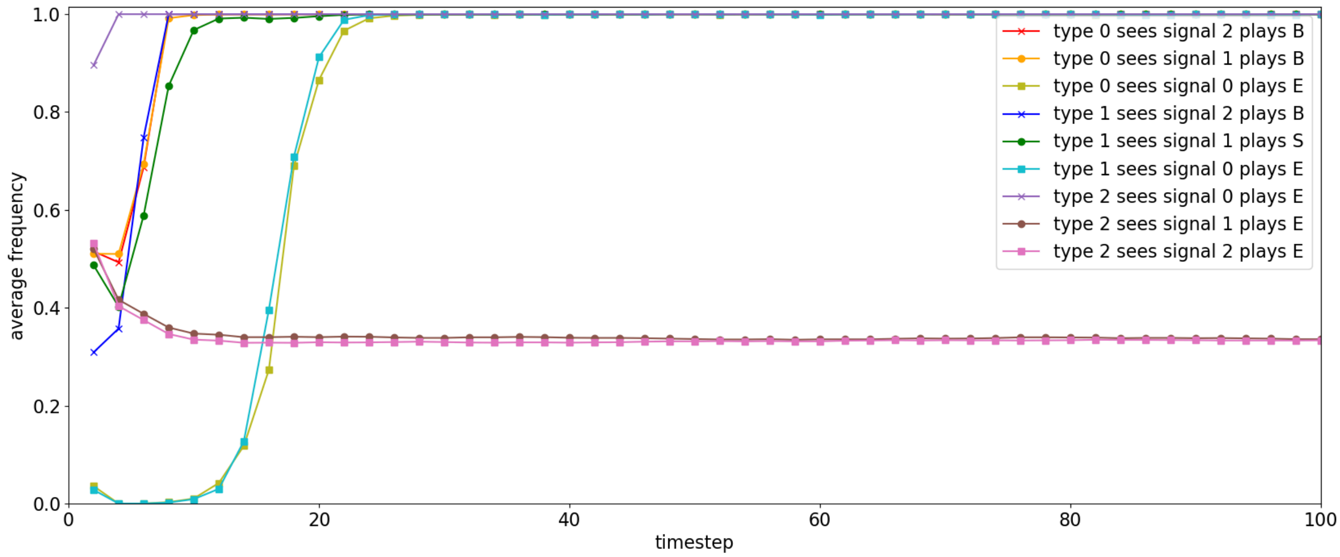

Run # 17

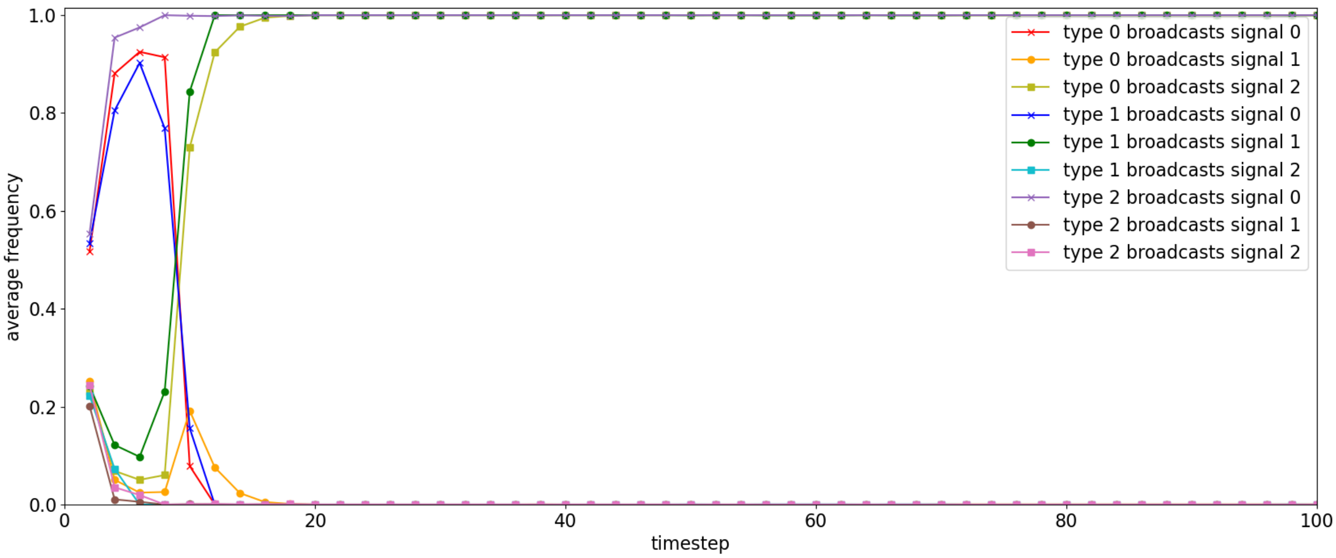
average frequency
timestep
type 0 broadcasts signal 0
type 0 broadcasts signal 1
type 0 broadcasts signal 2
type 1 broadcasts signal 0
type 1 broadcasts signal 1
type 1 broadcasts signal 2
type 2 broadcasts signal 0
type 2 broadcasts signal 1
type 2 broadcasts signal 2

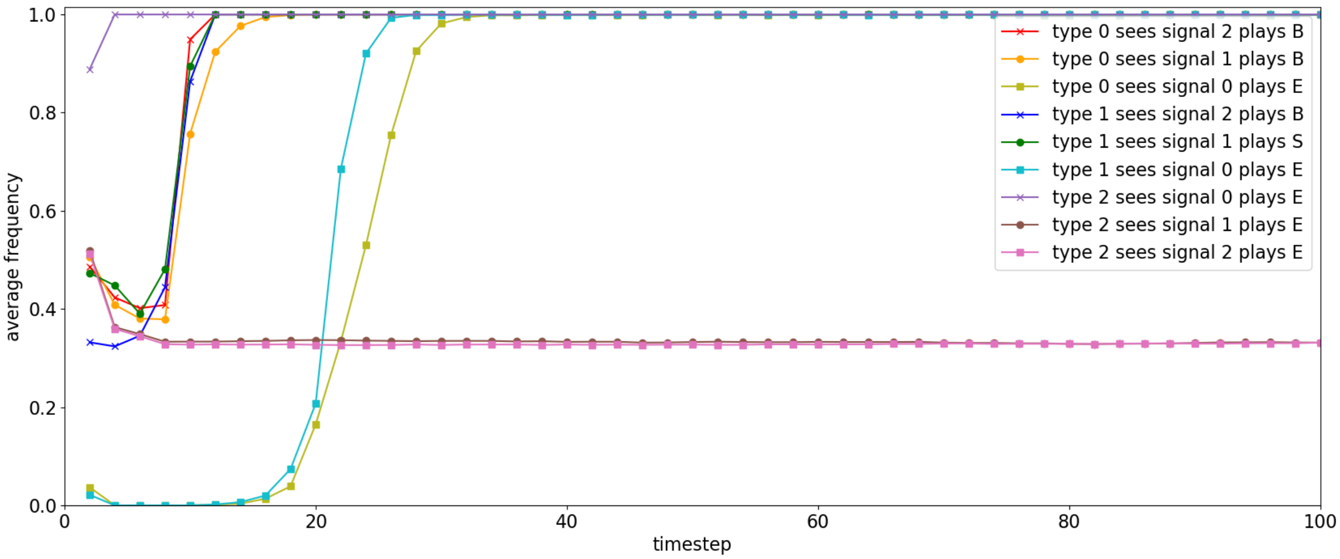
average frequency
timestep
type 0 sees signal 2 plays B
type 0 sees signal 1 plays B
type 0 sees signal 0 plays E
type 1 sees signal 2 plays B
type 1 sees signal 1 plays S
type 1 sees signal 0 plays E
type 2 sees signal 0 plays E
type 2 sees signal 1 plays E
type 2 sees signal 2 plays E

Run # 25

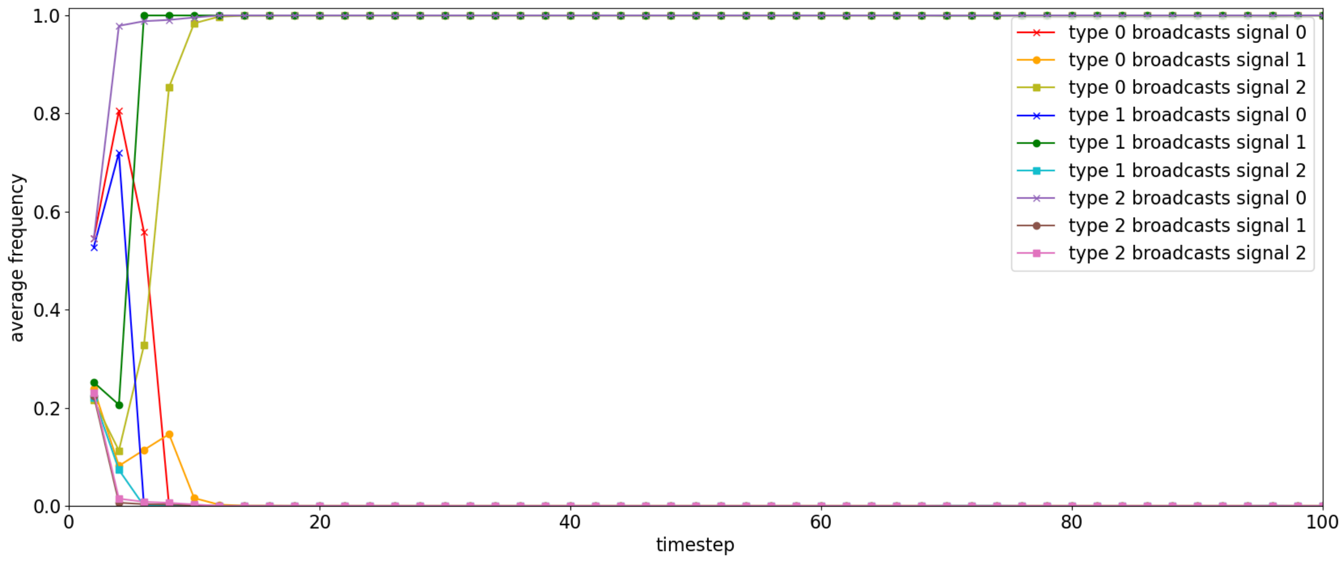
average frequency
timestep
type 0 broadcasts signal 0
type 0 broadcasts signal 1
type 0 broadcasts signal 2
type 1 broadcasts signal 0
type 1 broadcasts signal 1
type 1 broadcasts signal 2
type 2 broadcasts signal 0
type 2 broadcasts signal 1
type 2 broadcasts signal 2

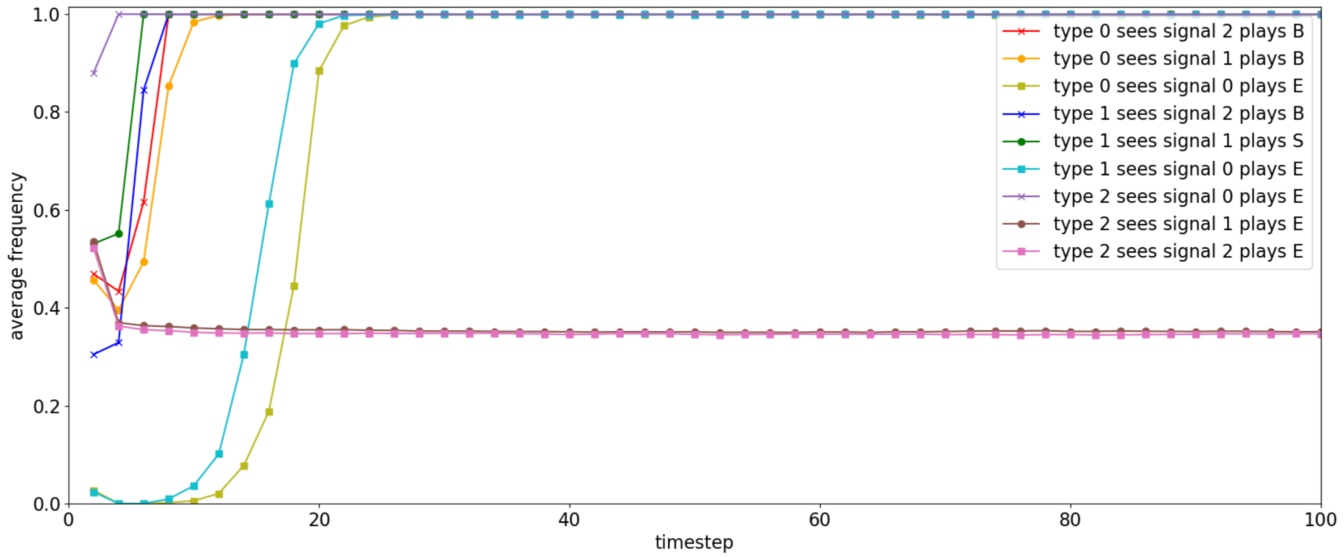
average frequency
timestep
type 0 sees signal 2 plays B
type 0 sees signal 1 plays B
type 0 sees signal 0 plays E
type 1 sees signal 2 plays B
type 1 sees signal 1 plays S
type 1 sees signal 0 plays E
type 2 sees signal 0 plays E
type 2 sees signal 1 plays E
type 2 sees signal 2 plays E

Run # 26

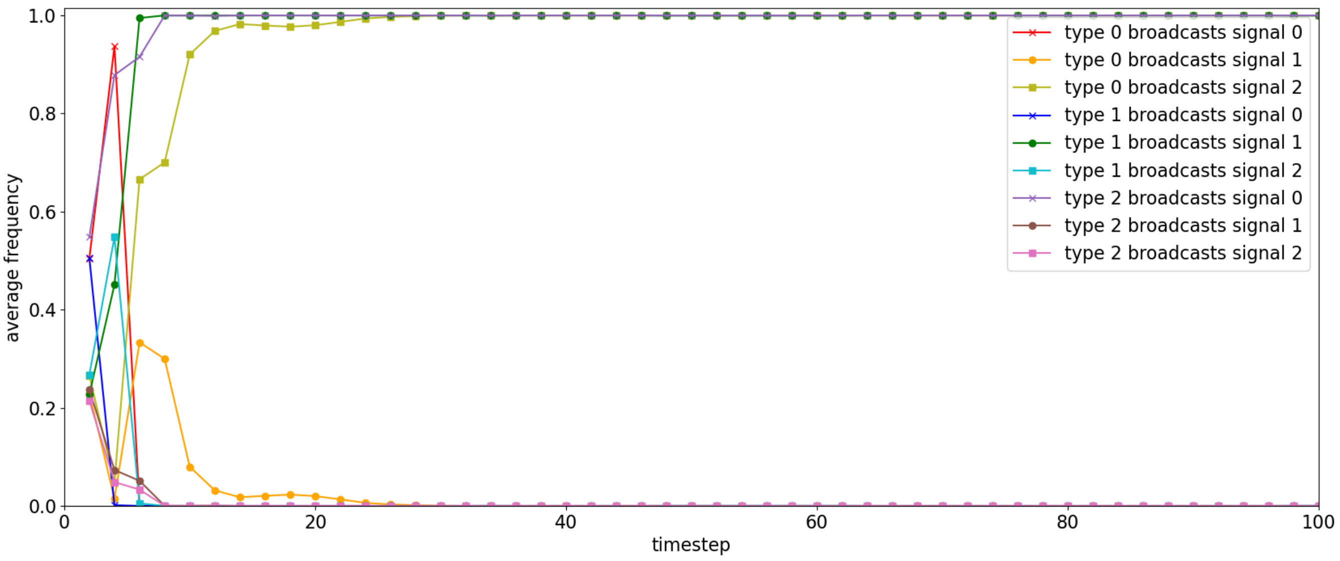
average frequency
timestep
type 0 broadcasts signal 0
type 0 broadcasts signal 1
type 0 broadcasts signal 2
type 1 broadcasts signal 0
type 1 broadcasts signal 1
type 1 broadcasts signal 2
type 2 broadcasts signal 0
type 2 broadcasts signal 1
type 2 broadcasts signal 2

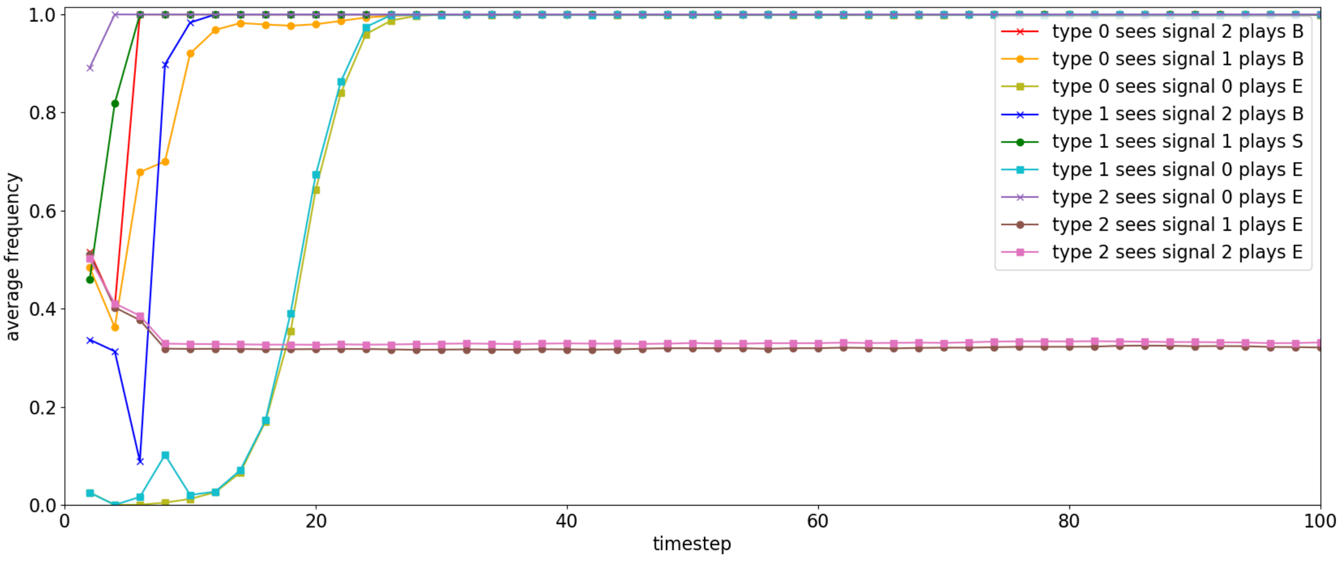
average frequency
timestep
type 0 sees signal 2 plays B
type 0 sees signal 1 plays B
type 0 sees signal 0 plays E
type 1 sees signal 2 plays B
type 1 sees signal 1 plays S
type 1 sees signal 0 plays E
type 2 sees signal 0 plays E
type 2 sees signal 1 plays E
type 2 sees signal 2 plays E

Run # 30

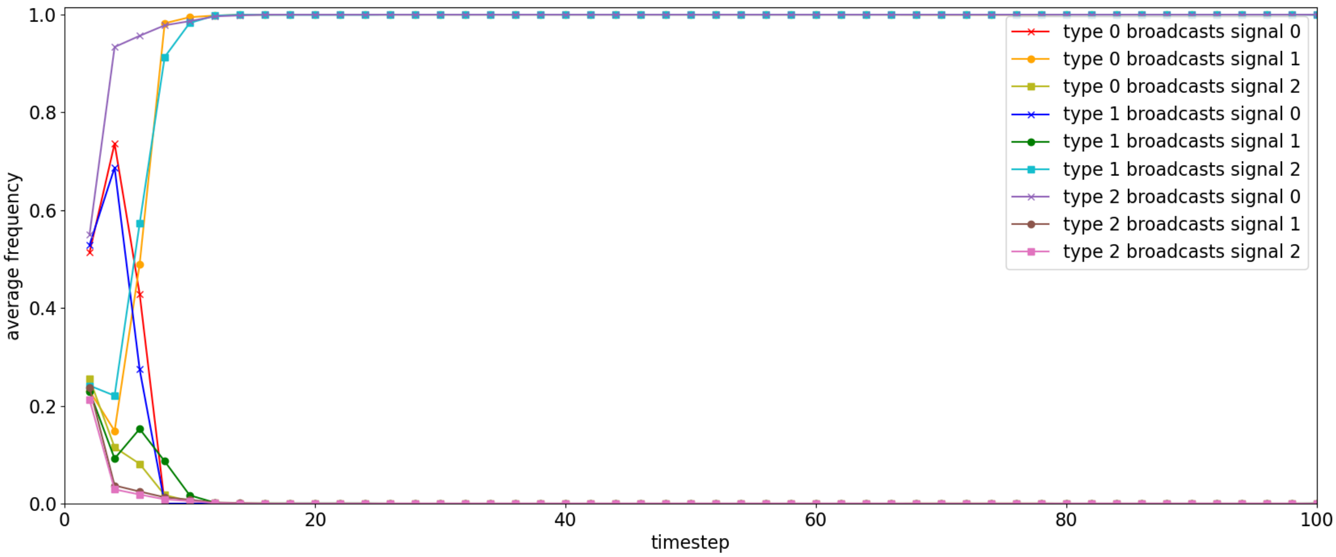
average frequency
timestep
type 0 broadcasts signal 0
type 0 broadcasts signal 1
type 0 broadcasts signal 2
type 1 broadcasts signal 0
type 1 broadcasts signal 1
type 1 broadcasts signal 2
type 2 broadcasts signal 0
type 2 broadcasts signal 1
type 2 broadcasts signal 2

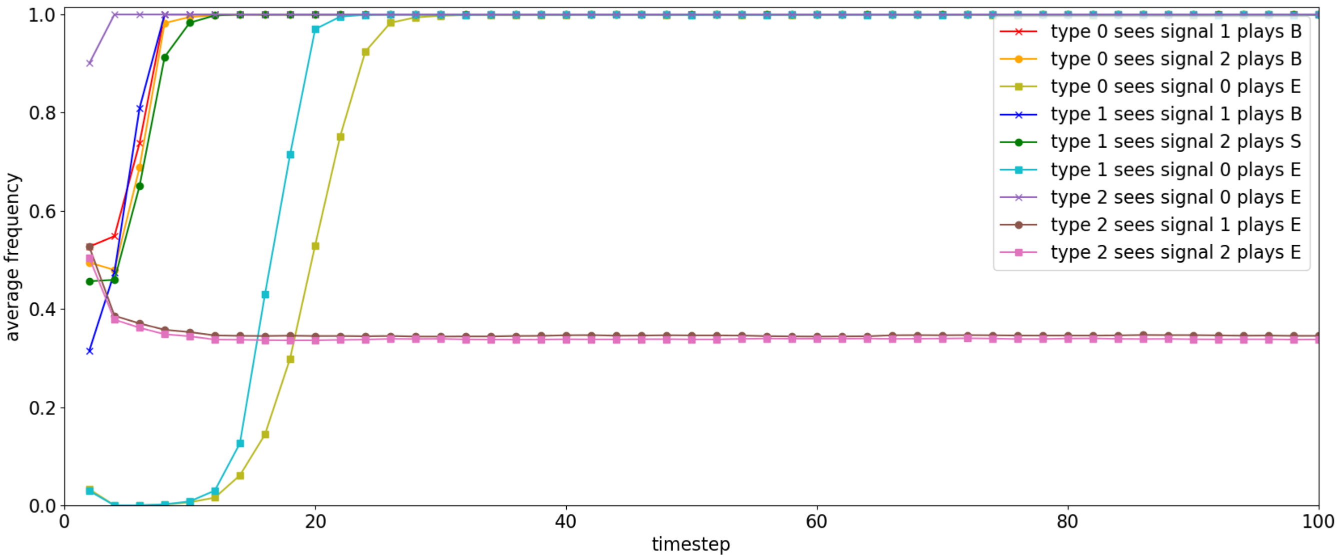
average frequency
timestep
type 0 sees signal 1 plays B
type 0 sees signal 2 plays B
type 0 sees signal 0 plays E
type 1 sees signal 1 plays B
type 1 sees signal 2 plays S
type 1 sees signal 0 plays E
type 2 sees signal 0 plays E
type 2 sees signal 1 plays E
type 2 sees signal 2 plays E

Run # 35

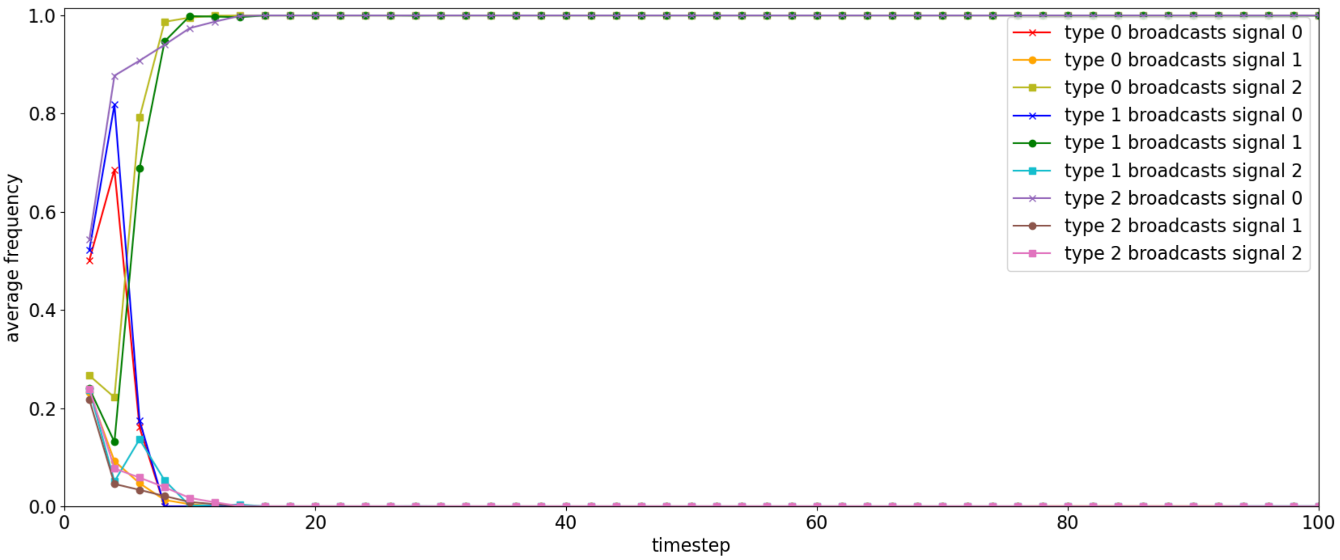
average frequency
timestep
type 0 broadcasts signal 0
type 0 broadcasts signal 1
type 0 broadcasts signal 2
type 1 broadcasts signal 0
type 1 broadcasts signal 1
type 1 broadcasts signal 2
type 2 broadcasts signal 0
type 2 broadcasts signal 1
type 2 broadcasts signal 2

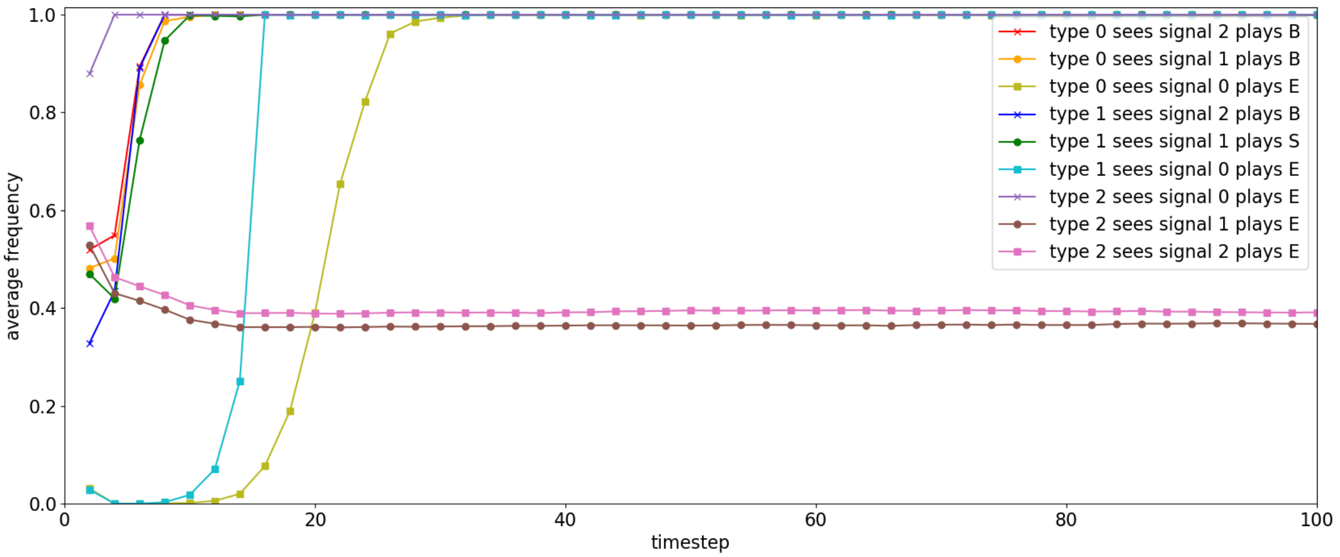
average frequency
timestep
type 0 sees signal 2 plays B
type 0 sees signal 1 plays B
type 0 sees signal 0 plays E
type 1 sees signal 2 plays B
type 1 sees signal 1 plays S
type 1 sees signal 0 plays E
type 2 sees signal 0 plays E
type 2 sees signal 1 plays E
type 2 sees signal 2 plays E

Run # 40

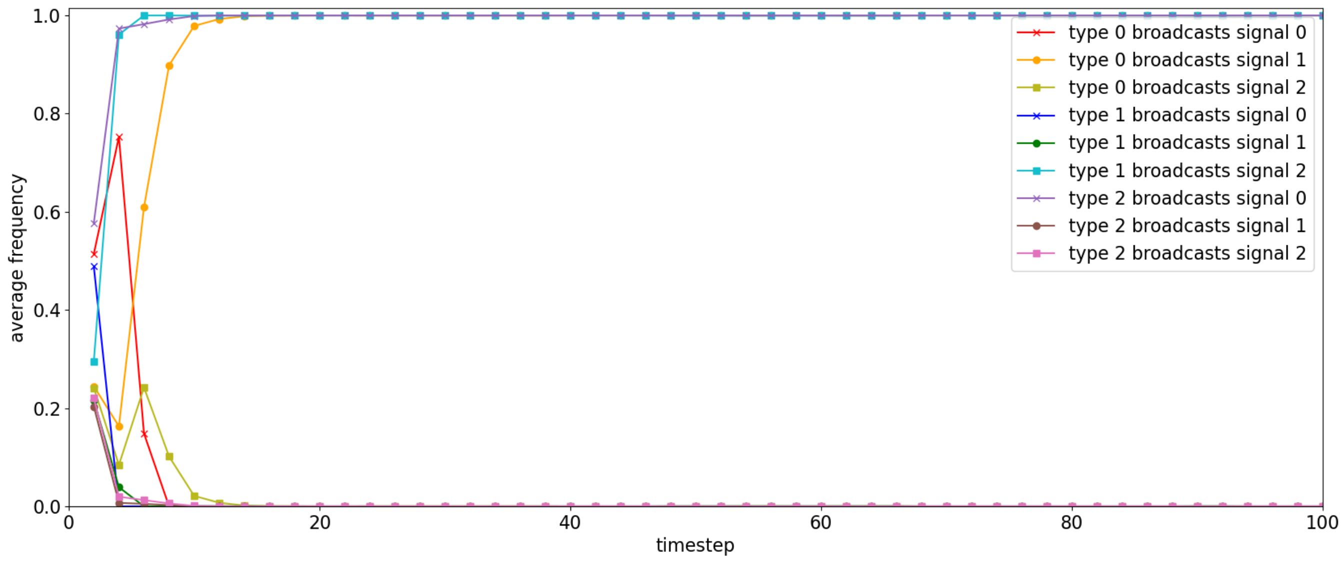
average frequency
timestep
type 0 broadcasts signal 0
type 0 broadcasts signal 1
type 0 broadcasts signal 2
type 1 broadcasts signal 0
type 1 broadcasts signal 1
type 1 broadcasts signal 2
type 2 broadcasts signal 0
type 2 broadcasts signal 1
type 2 broadcasts signal 2

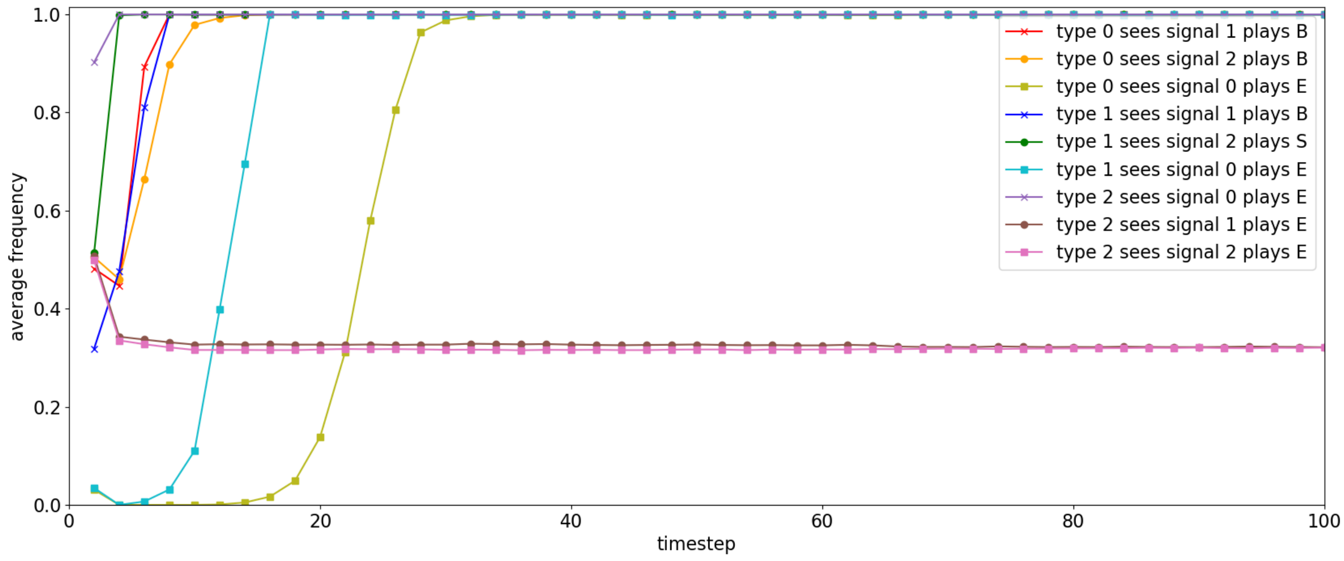
average frequency
timestep
type 0 sees signal 1 plays B
type 0 sees signal 2 plays B
type 0 sees signal 0 plays E
type 1 sees signal 1 plays B
type 1 sees signal 2 plays S
type 1 sees signal 0 plays E
type 2 sees signal 0 plays E
type 2 sees signal 1 plays E
type 2 sees signal 2 plays E

Run # 47

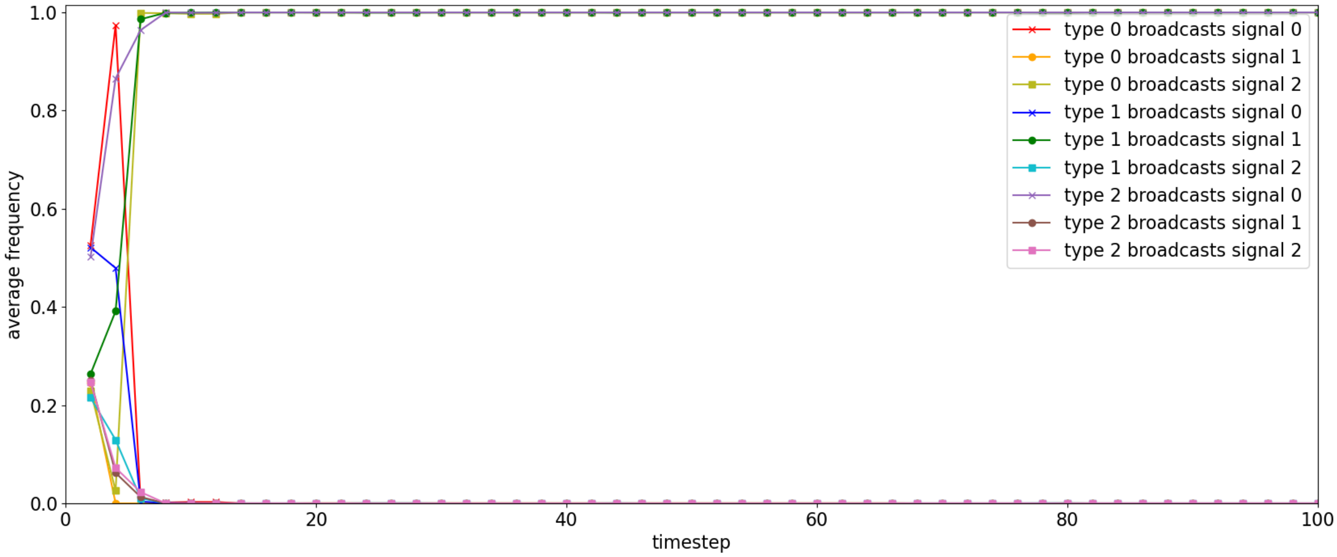
average frequency
timestep
type 0 broadcasts signal 0
type 0 broadcasts signal 1
type 0 broadcasts signal 2
type 1 broadcasts signal 0
type 1 broadcasts signal 1
type 1 broadcasts signal 2
type 2 broadcasts signal 0
type 2 broadcasts signal 1
type 2 broadcasts signal 2

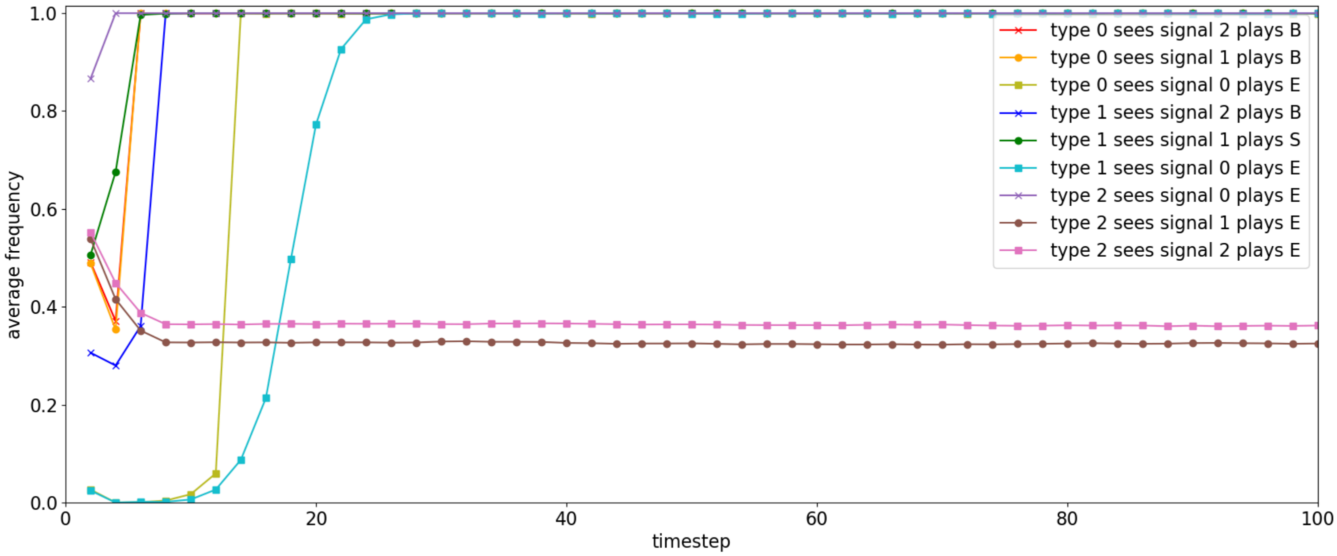
average frequency
timestep
type 0 sees signal 2 plays B
type 0 sees signal 1 plays B
type 0 sees signal 0 plays E
type 1 sees signal 2 plays B
type 1 sees signal 1 plays S
type 1 sees signal 0 plays E
type 2 sees signal 0 plays E
type 2 sees signal 1 plays E
type 2 sees signal 2 plays E

Run # 49

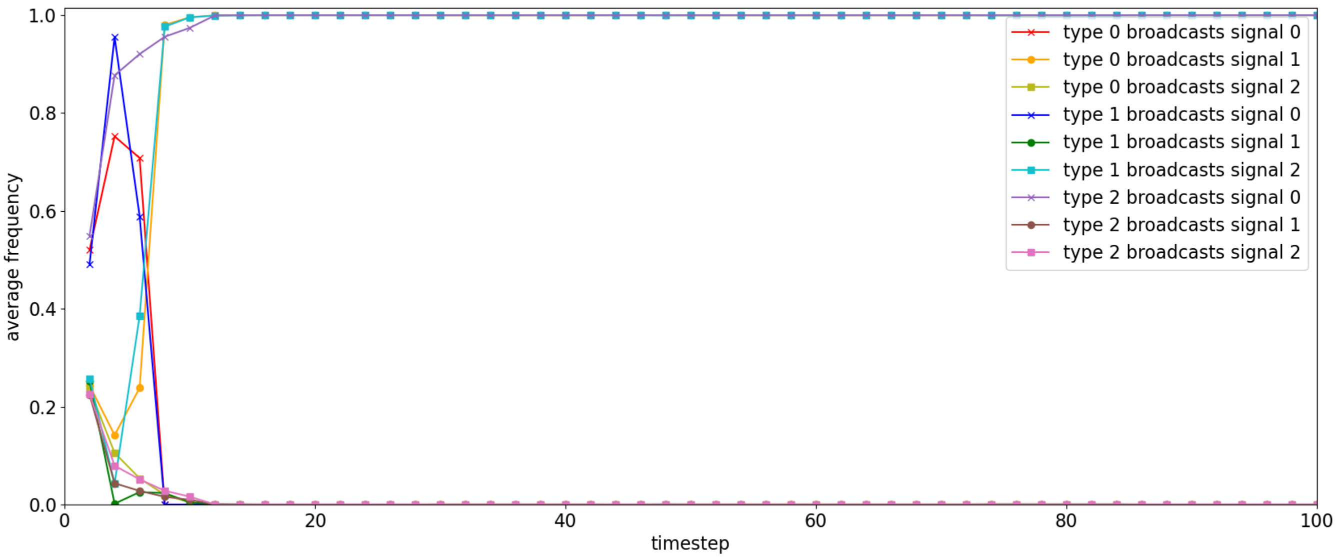

average frequency
timestep
type 0 broadcasts signal 0
type 0 broadcasts signal 1
type 0 broadcasts signal 2
type 1 broadcasts signal 0
type 1 broadcasts signal 1
type 1 broadcasts signal 2
type 2 broadcasts signal 0
type 2 broadcasts signal 1
type 2 broadcasts signal 2

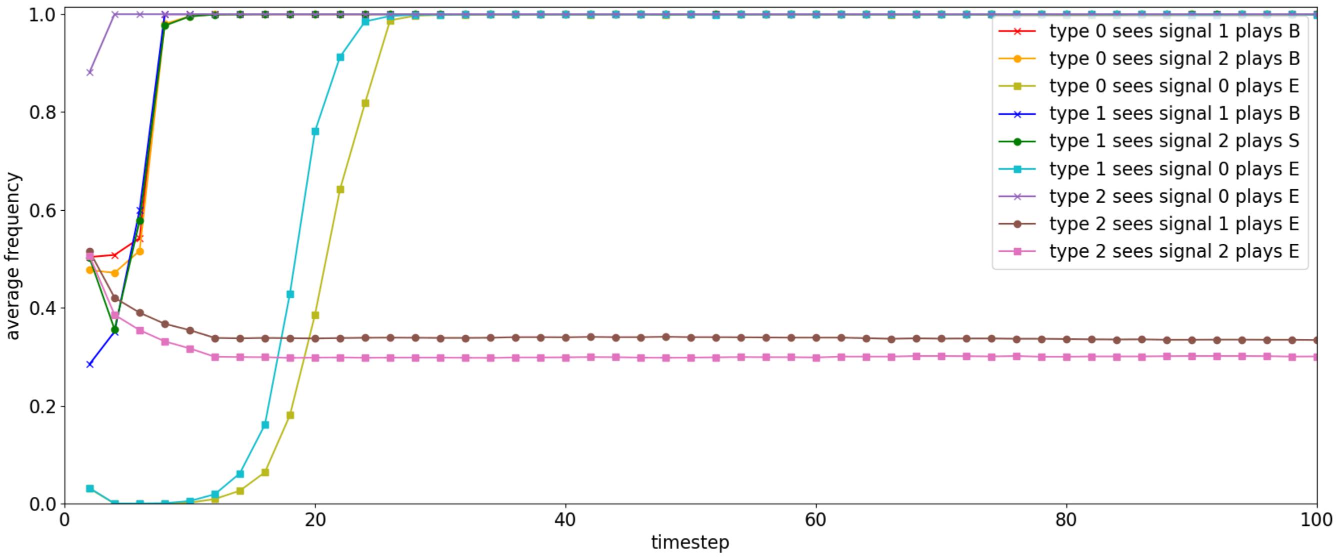

average frequency
timestep
type 0 sees signal 1 plays B
type 0 sees signal 2 plays B
type 0 sees signal 0 plays E
type 1 sees signal 1 plays B
type 1 sees signal 2 plays S
type 1 sees signal 0 plays E
type 2 sees signal 0 plays E
type 2 sees signal 1 plays E
type 2 sees signal 2 plays E

Run # 50

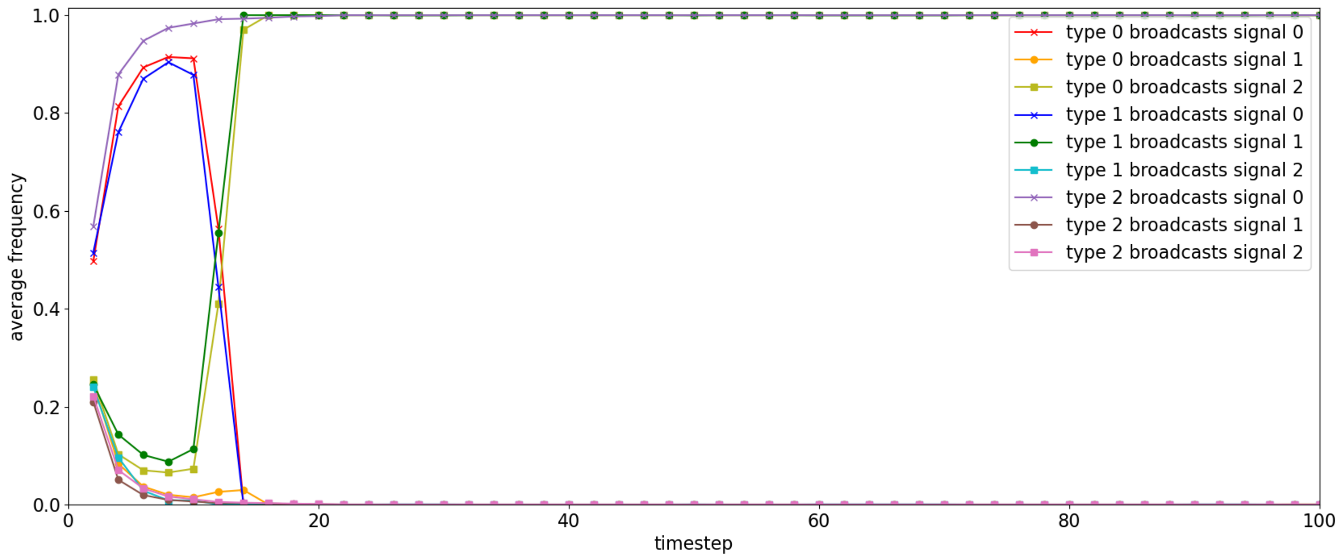
average frequency
timestep
type 0 broadcasts signal 0
type 0 broadcasts signal 1
type 0 broadcasts signal 2
type 1 broadcasts signal 0
type 1 broadcasts signal 1
type 1 broadcasts signal 2
type 2 broadcasts signal 0
type 2 broadcasts signal 1
type 2 broadcasts signal 2

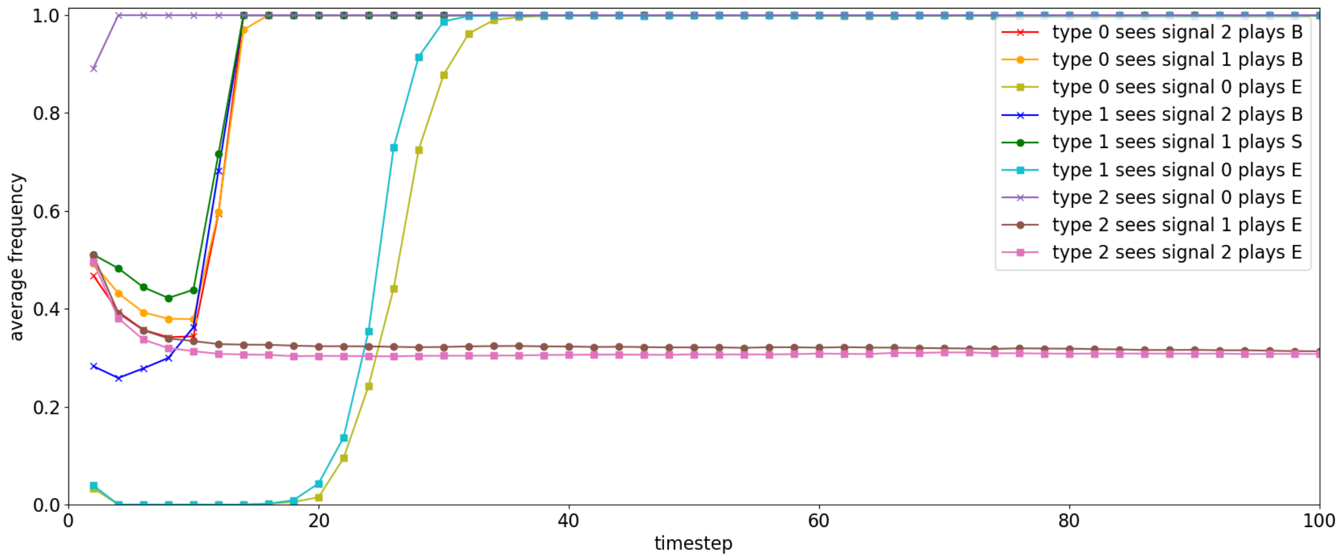
average frequency
timestep
type 0 sees signal 2 plays B
type 0 sees signal 1 plays B
type 0 sees signal 0 plays E
type 1 sees signal 2 plays B
type 1 sees signal 1 plays S
type 1 sees signal 0 plays E
type 2 sees signal 0 plays E
type 2 sees signal 1 plays E
type 2 sees signal 2 plays E

outcomes (xiii): necessarily characterized by type 2 signaling 0

Run # 10

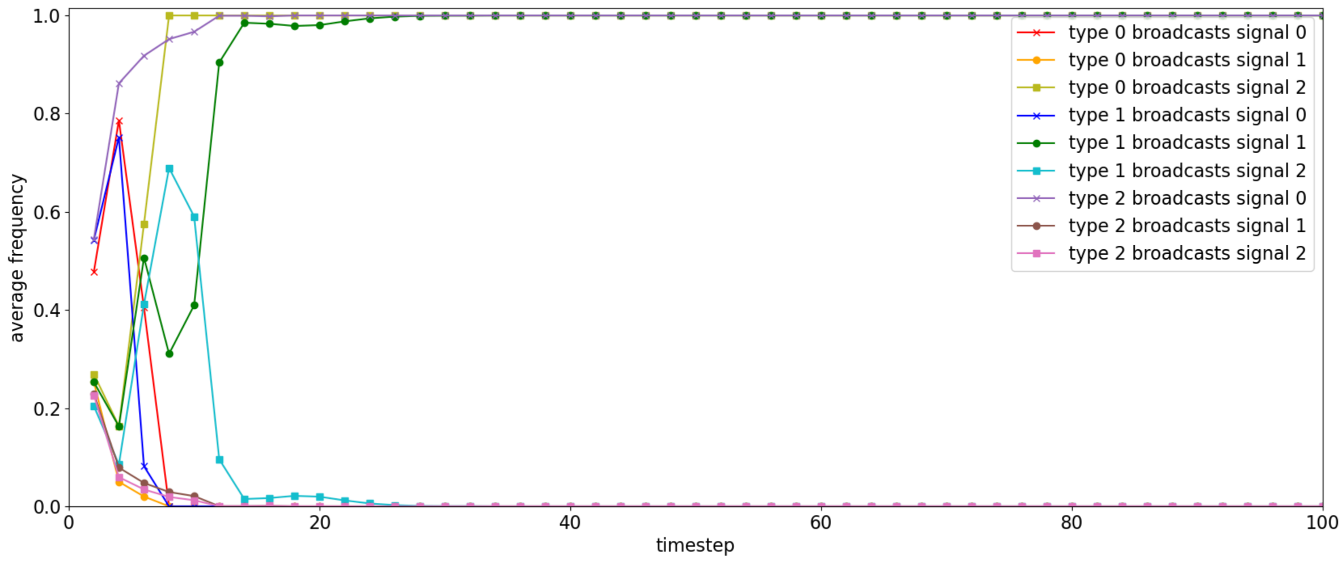

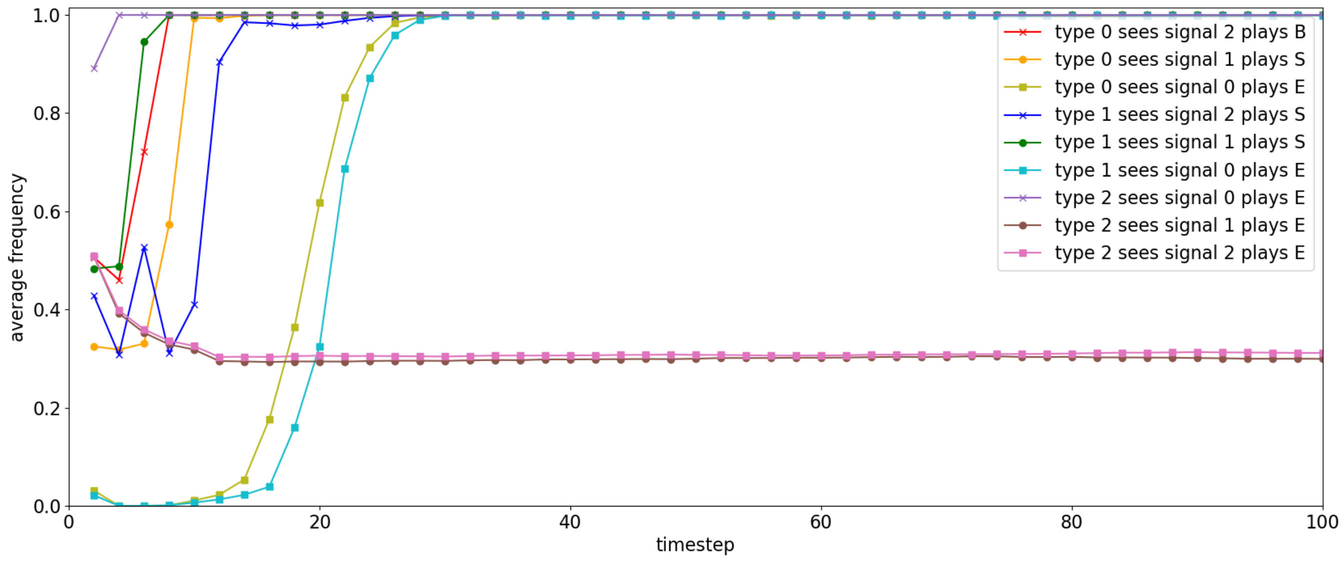

Run # 15

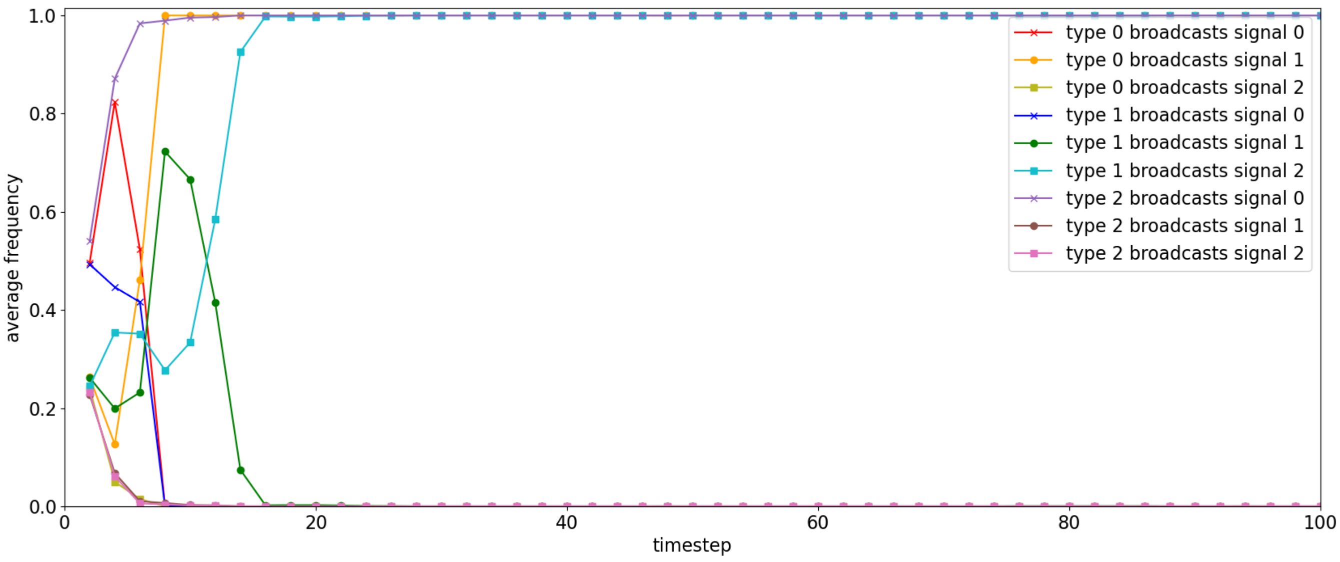
average frequency
timestep
type 0 broadcasts signal 0
type 0 broadcasts signal 1
type 0 broadcasts signal 2
type 1 broadcasts signal 0
type 1 broadcasts signal 1
type 1 broadcasts signal 2
type 2 broadcasts signal 0
type 2 broadcasts signal 1
type 2 broadcasts signal 2

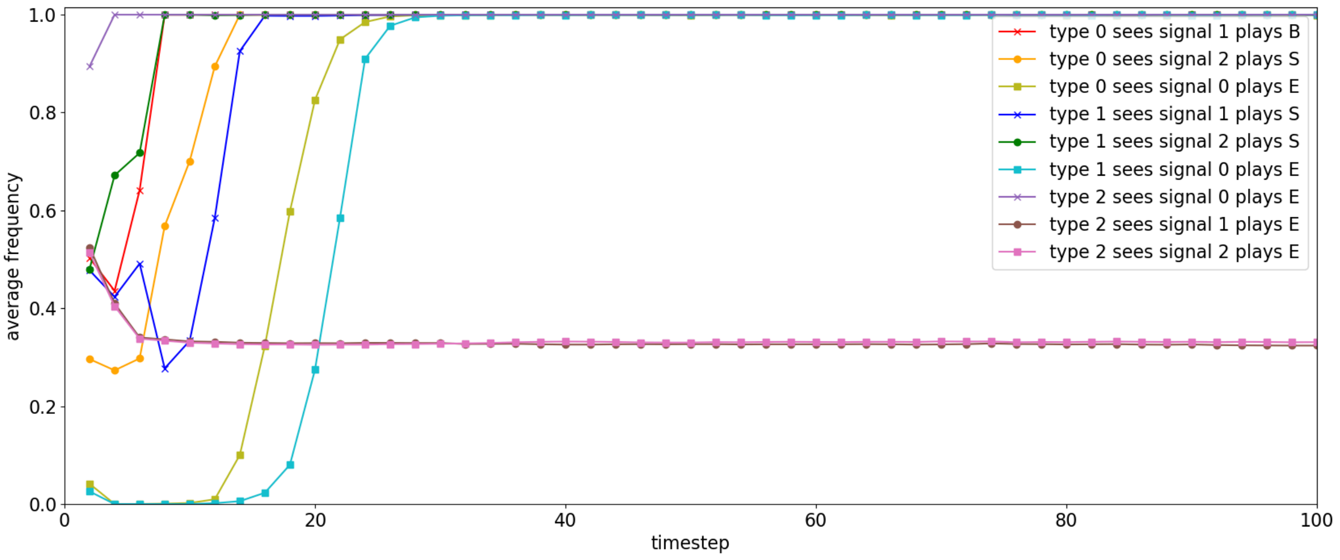
average frequency
timestep
type 0 sees signal 1 plays B
type 0 sees signal 2 plays S
type 0 sees signal 0 plays E
type 1 sees signal 1 plays S
type 1 sees signal 2 plays S
type 1 sees signal 0 plays E
type 2 sees signal 0 plays E
type 2 sees signal 1 plays E
type 2 sees signal 2 plays E

Run # 21

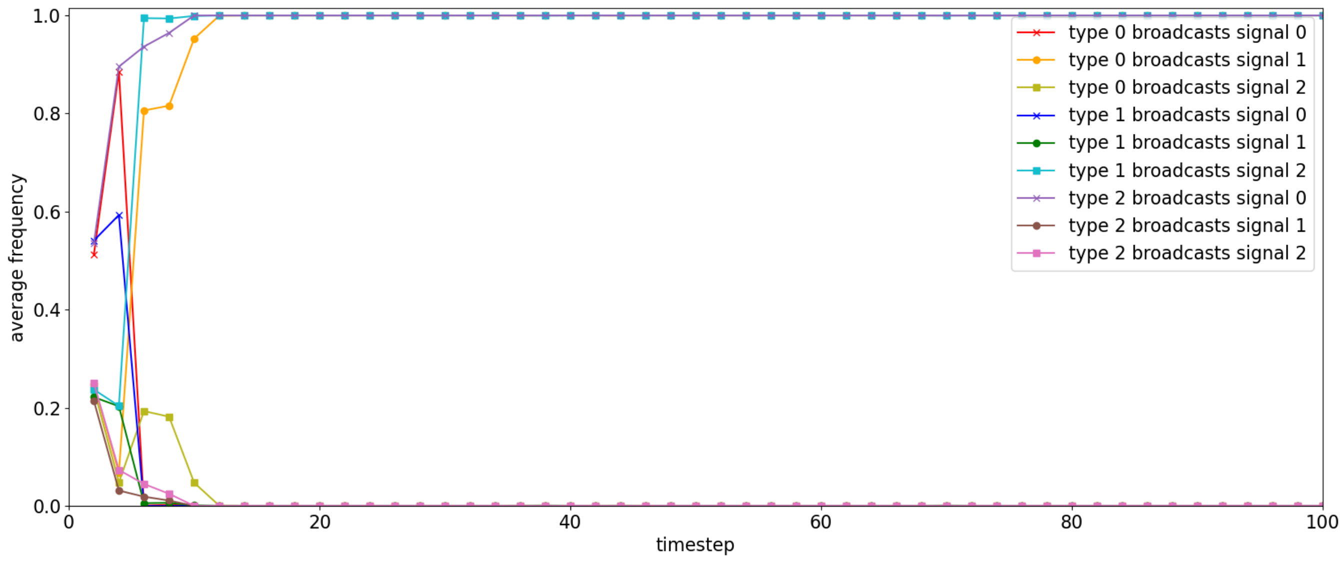
average frequency
timestep
type 0 broadcasts signal 0
type 0 broadcasts signal 1
type 0 broadcasts signal 2
type 1 broadcasts signal 0
type 1 broadcasts signal 1
type 1 broadcasts signal 2
type 2 broadcasts signal 0
type 2 broadcasts signal 1
type 2 broadcasts signal 2

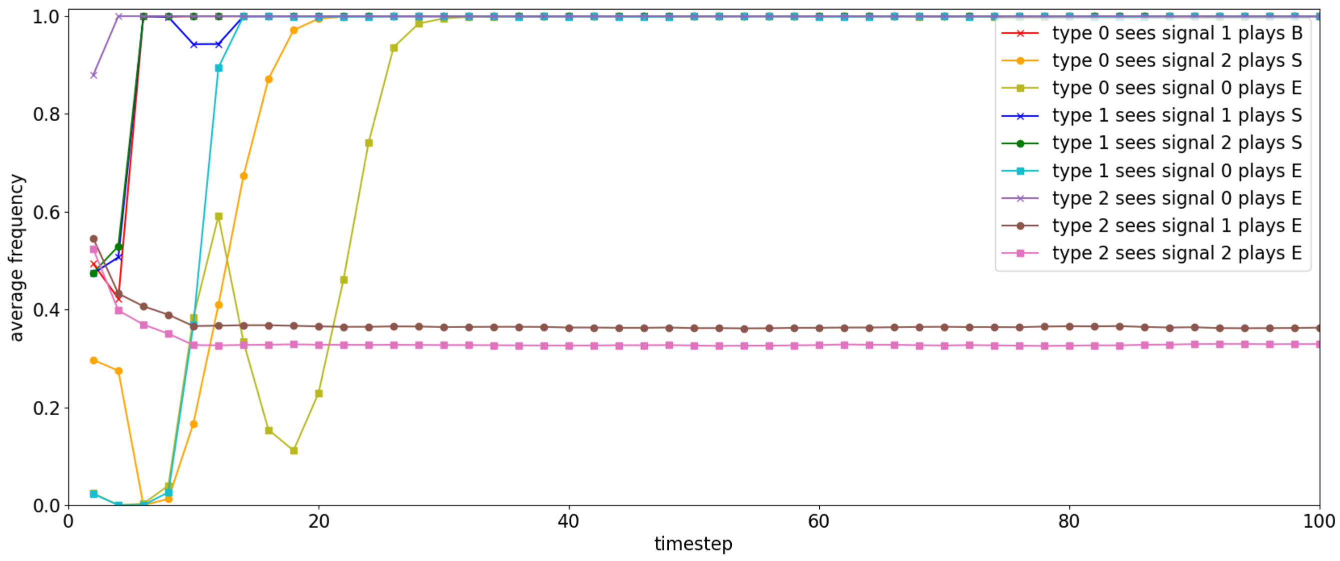
average frequency
timestep
type 0 sees signal 1 plays B
type 0 sees signal 2 plays S
type 0 sees signal 0 plays E
type 1 sees signal 1 plays S
type 1 sees signal 2 plays S
type 1 sees signal 0 plays E
type 2 sees signal 0 plays E
type 2 sees signal 1 plays E
type 2 sees signal 2 plays E

Run # 38

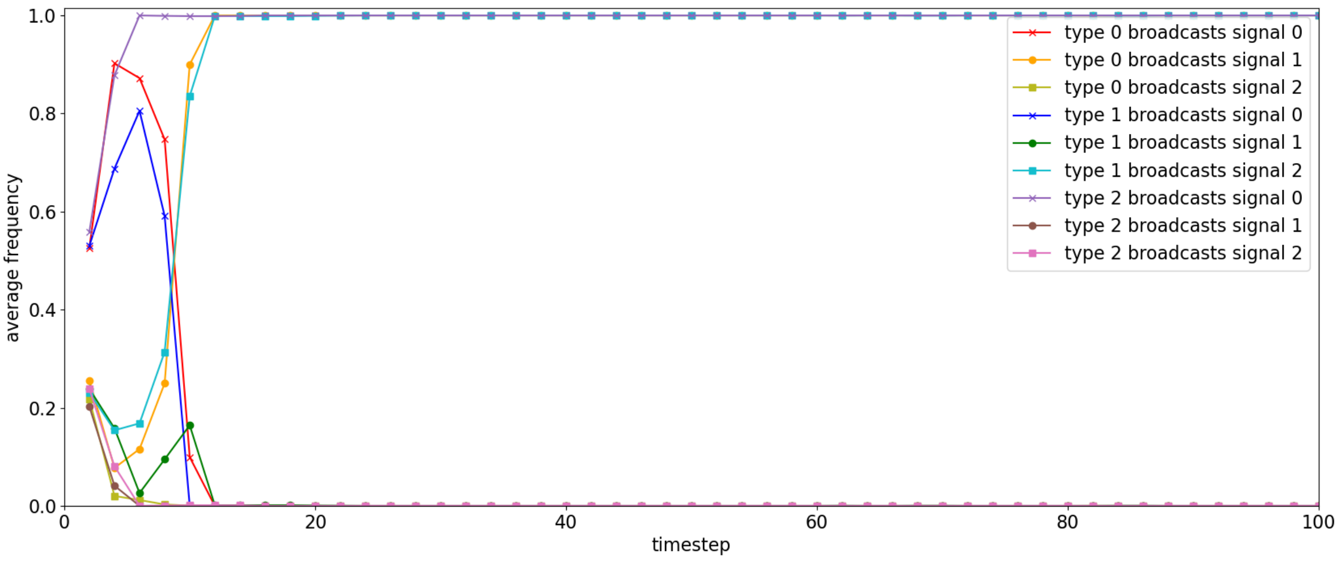
average frequency
timestep
type 0 broadcasts signal 0
type 0 broadcasts signal 1
type 0 broadcasts signal 2
type 1 broadcasts signal 0
type 1 broadcasts signal 1
type 1 broadcasts signal 2
type 2 broadcasts signal 0
type 2 broadcasts signal 1
type 2 broadcasts signal 2

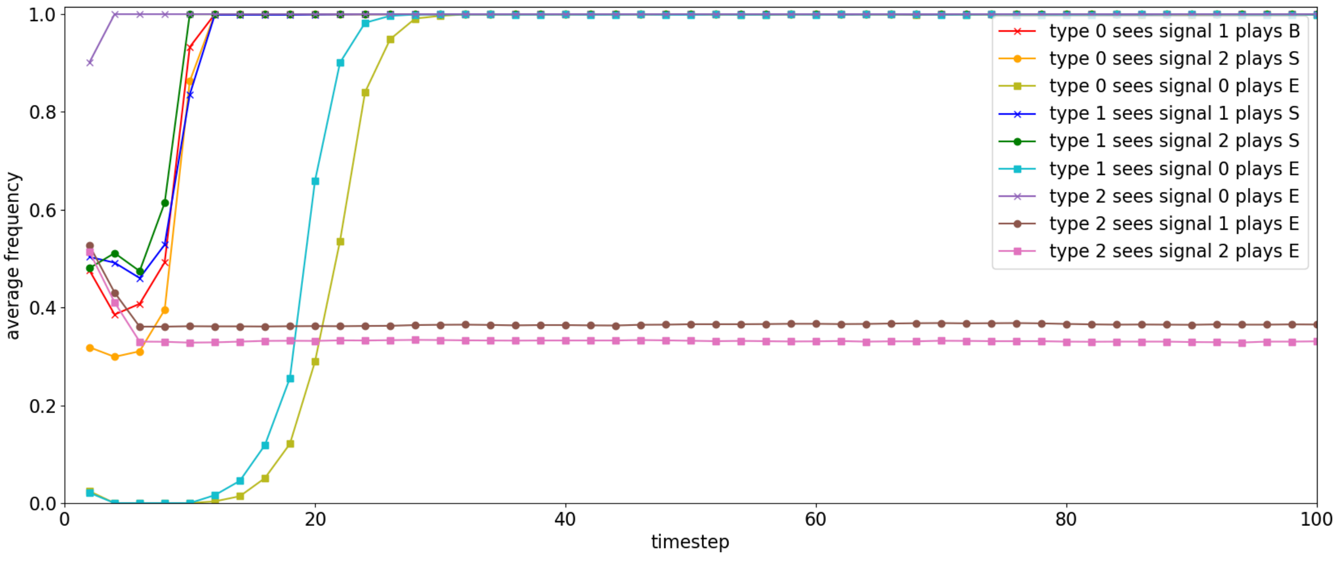
average frequency
timestep
type 0 sees signal 1 plays B
type 0 sees signal 2 plays S
type 0 sees signal 0 plays E
type 1 sees signal 1 plays S
type 1 sees signal 2 plays S
type 1 sees signal 0 plays E
type 2 sees signal 0 plays E
type 2 sees signal 1 plays E
type 2 sees signal 2 plays E

Run # 55

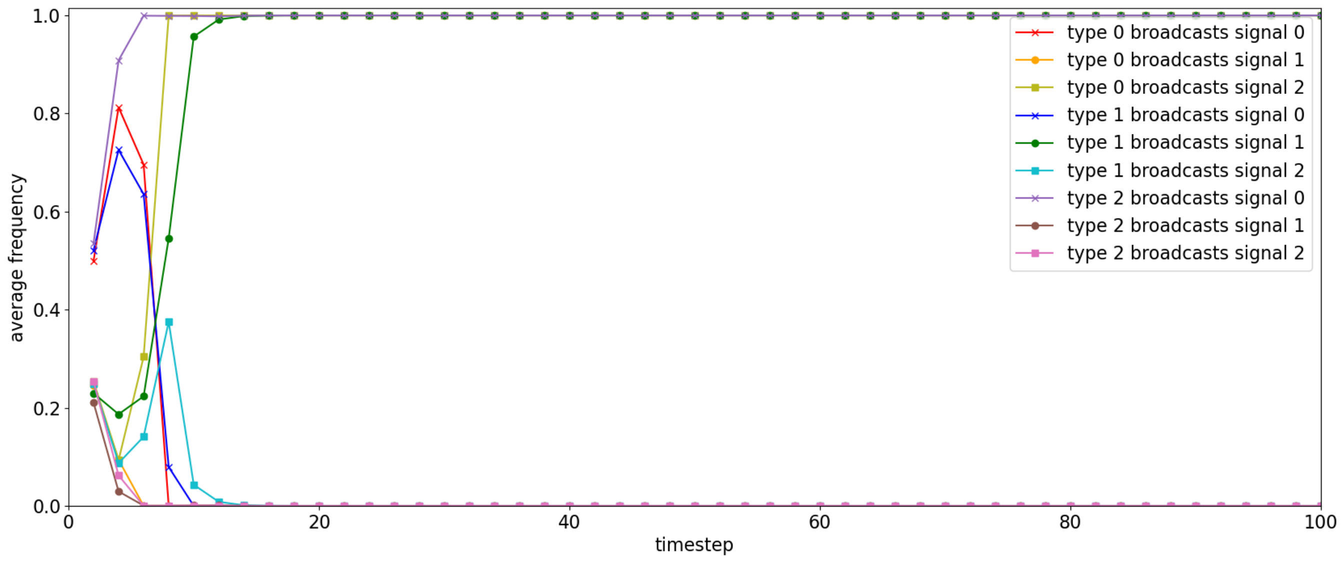
average frequency
timestep
type 0 broadcasts signal 0
type 0 broadcasts signal 1
type 0 broadcasts signal 2
type 1 broadcasts signal 0
type 1 broadcasts signal 1
type 1 broadcasts signal 2
type 2 broadcasts signal 0
type 2 broadcasts signal 1
type 2 broadcasts signal 2

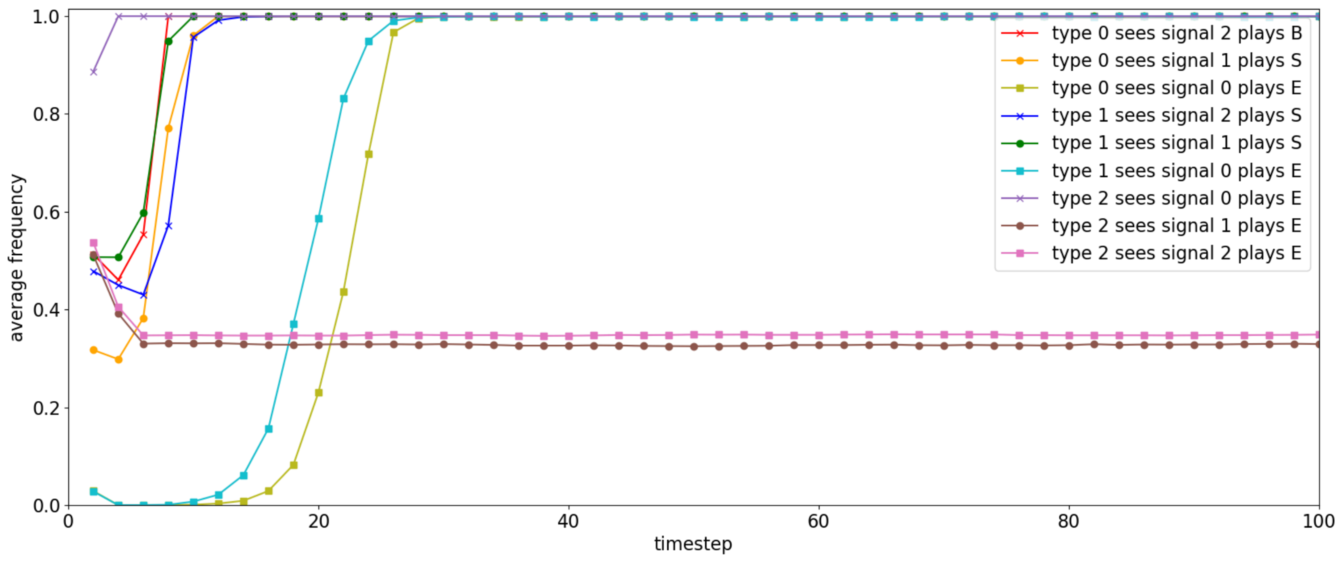
average frequency
timestep
type 0 sees signal 2 plays B
type 0 sees signal 1 plays S
type 0 sees signal 0 plays E
type 1 sees signal 2 plays S
type 1 sees signal 1 plays S
type 1 sees signal 0 plays E
type 2 sees signal 0 plays E
type 2 sees signal 1 plays E
type 2 sees signal 2 plays E

Run # 74

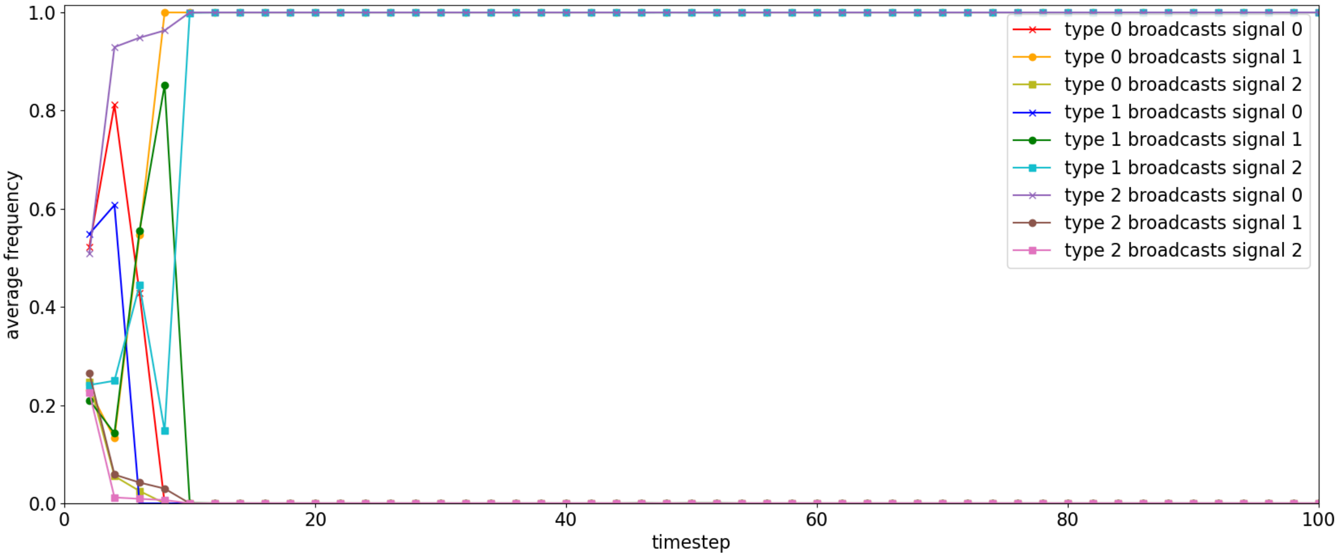
average frequency
timestep
type 0 broadcasts signal 0
type 0 broadcasts signal 1
type 0 broadcasts signal 2
type 1 broadcasts signal 0
type 1 broadcasts signal 1
type 1 broadcasts signal 2
type 2 broadcasts signal 0
type 2 broadcasts signal 1
type 2 broadcasts signal 2

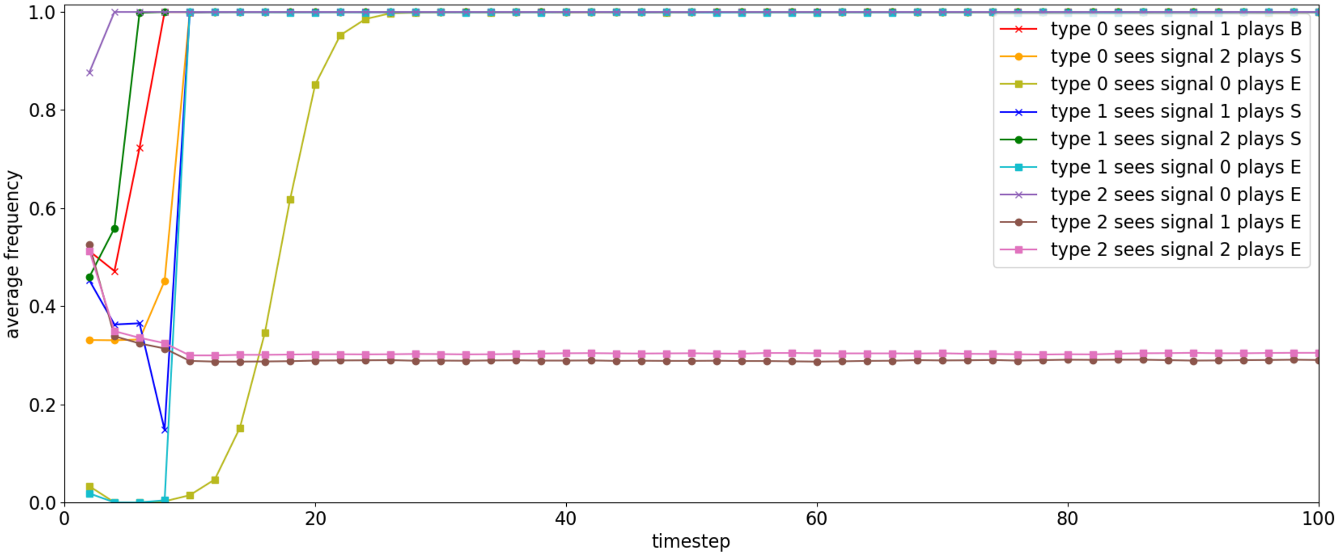
average frequency
timestep
type 0 sees signal 1 plays B
type 0 sees signal 2 plays S
type 0 sees signal 0 plays E
type 1 sees signal 1 plays S
type 1 sees signal 2 plays S
type 1 sees signal 0 plays E
type 2 sees signal 0 plays E
type 2 sees signal 1 plays E
type 2 sees signal 2 plays E

Run # 75

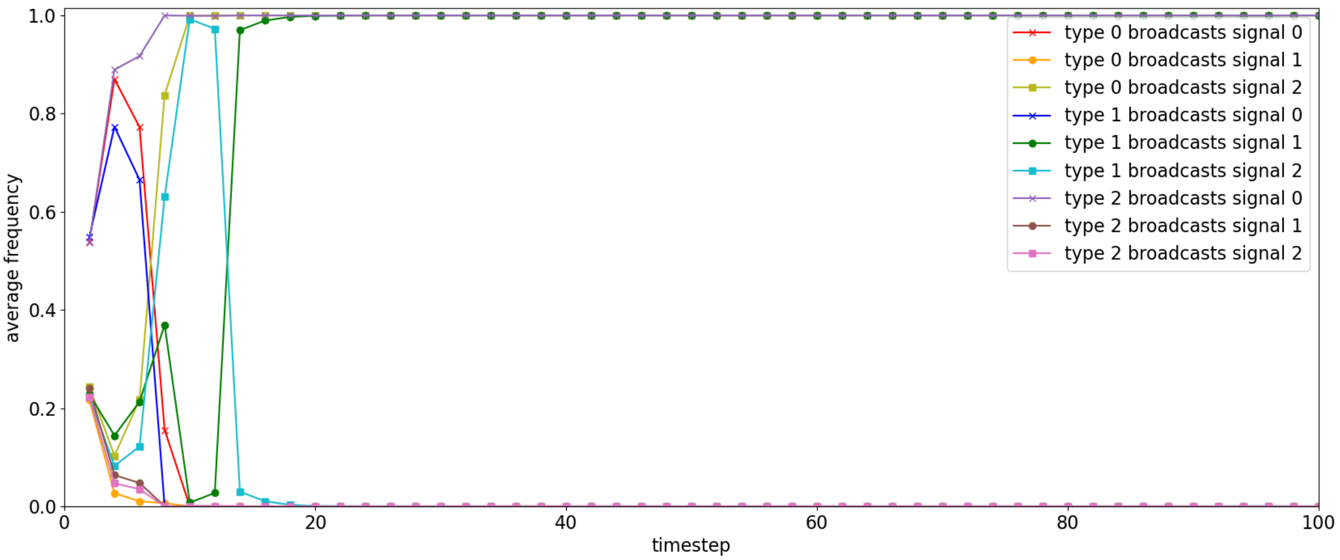

average frequency
timestep
0.0
0.2
0.4
0.6
0.8
1.0
0
20
40
60
80
100
type 0 broadcasts signal 0
type 0 broadcasts signal 1
type 0 broadcasts signal 2
type 1 broadcasts signal 0
type 1 broadcasts signal 1
type 1 broadcasts signal 2
type 2 broadcasts signal 0
type 2 broadcasts signal 1
type 2 broadcasts signal 2

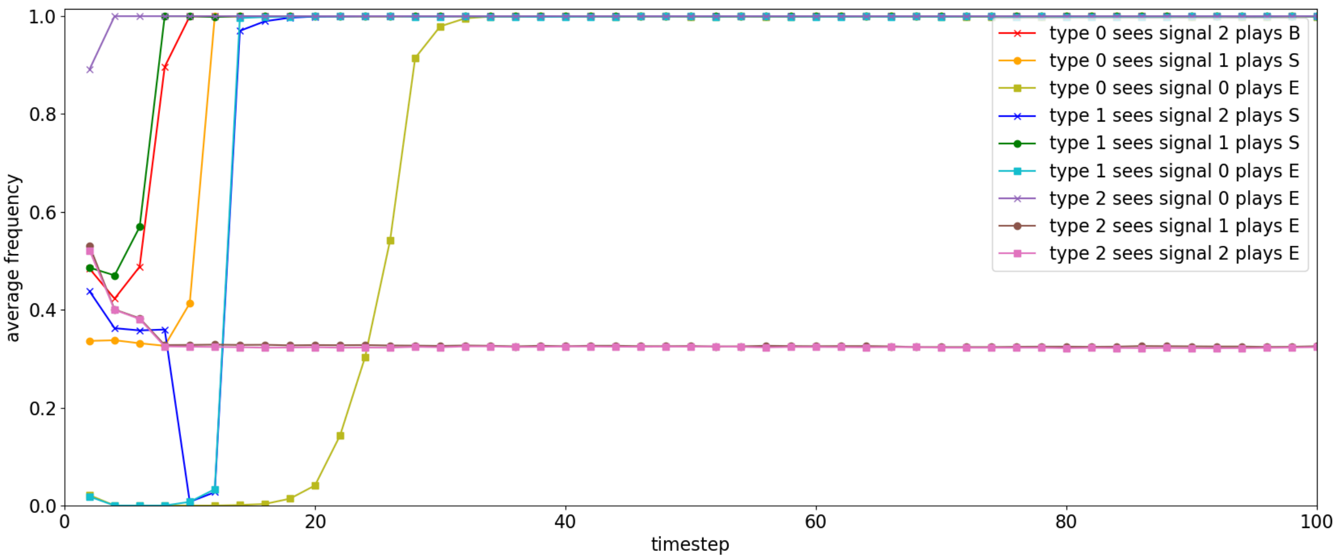

average frequency
timestep
0.0
0.2
0.4
0.6
0.8
1.0
0
20
40
60
80
100
type 0 sees signal 2 plays B
type 0 sees signal 1 plays S
type 0 sees signal 0 plays E
type 1 sees signal 2 plays S
type 1 sees signal 1 plays S
type 1 sees signal 0 plays E
type 2 sees signal 0 plays E
type 2 sees signal 1 plays E
type 2 sees signal 2 plays E

Run # 76

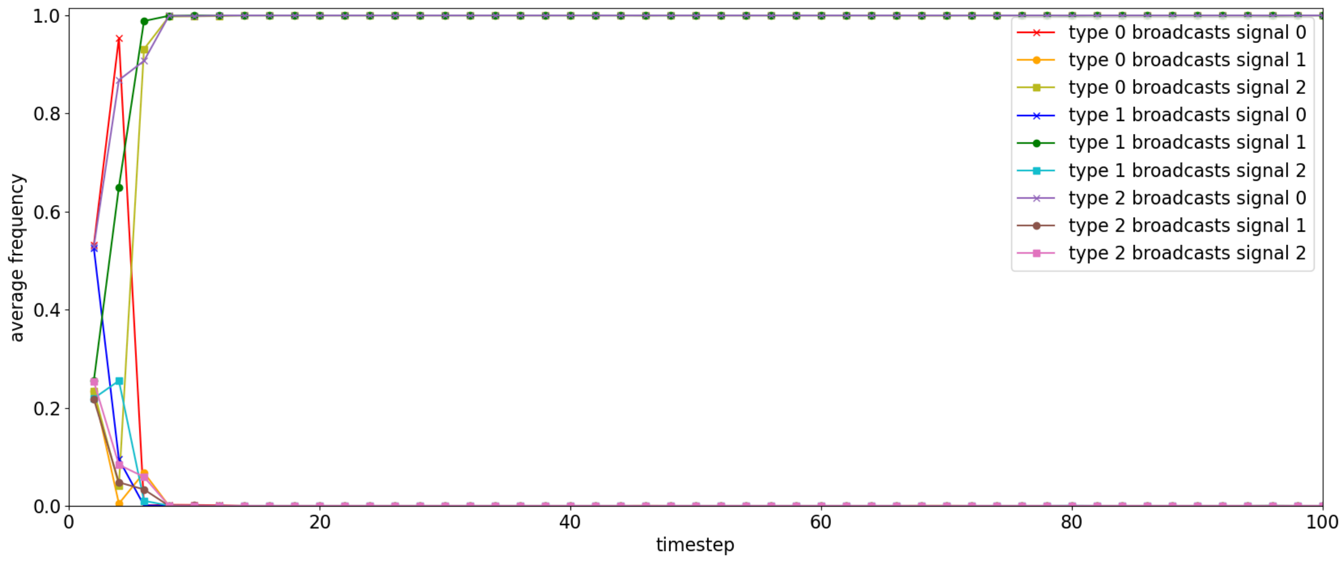
average frequency
timestep
type 0 broadcasts signal 0
type 0 broadcasts signal 1
type 0 broadcasts signal 2
type 1 broadcasts signal 0
type 1 broadcasts signal 1
type 1 broadcasts signal 2
type 2 broadcasts signal 0
type 2 broadcasts signal 1
type 2 broadcasts signal 2

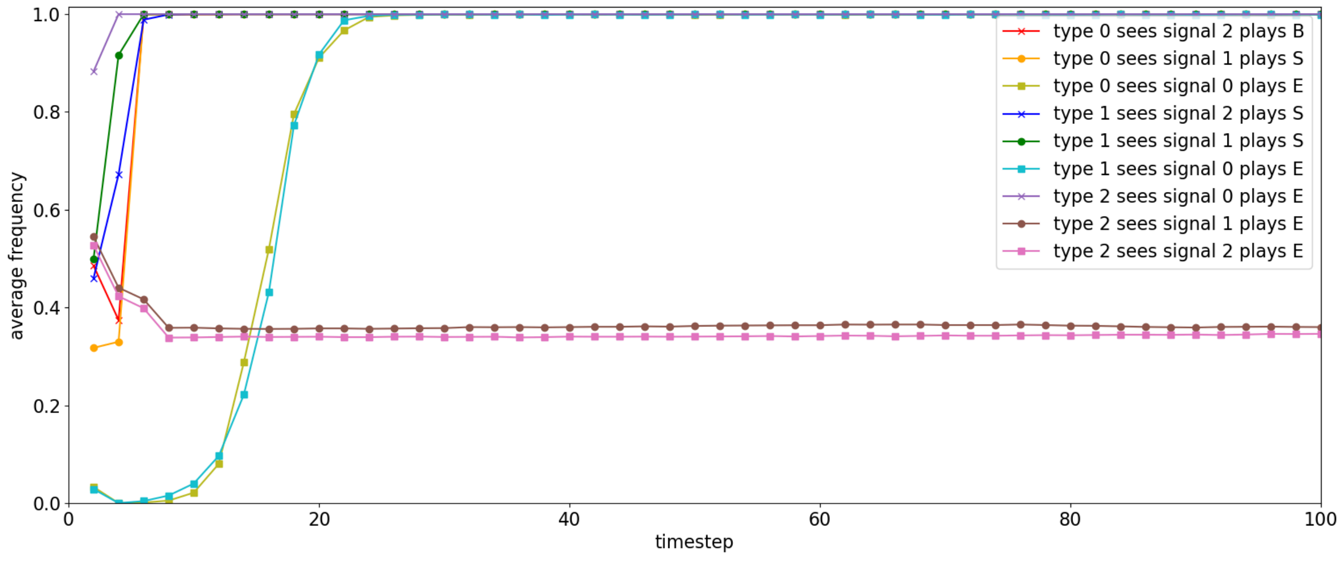
average frequency
timestep
type 0 sees signal 2 plays B
type 0 sees signal 1 plays S
type 0 sees signal 0 plays E
type 1 sees signal 2 plays S
type 1 sees signal 1 plays S
type 1 sees signal 0 plays E
type 2 sees signal 0 plays E
type 2 sees signal 1 plays E
type 2 sees signal 2 plays E

Run # 79

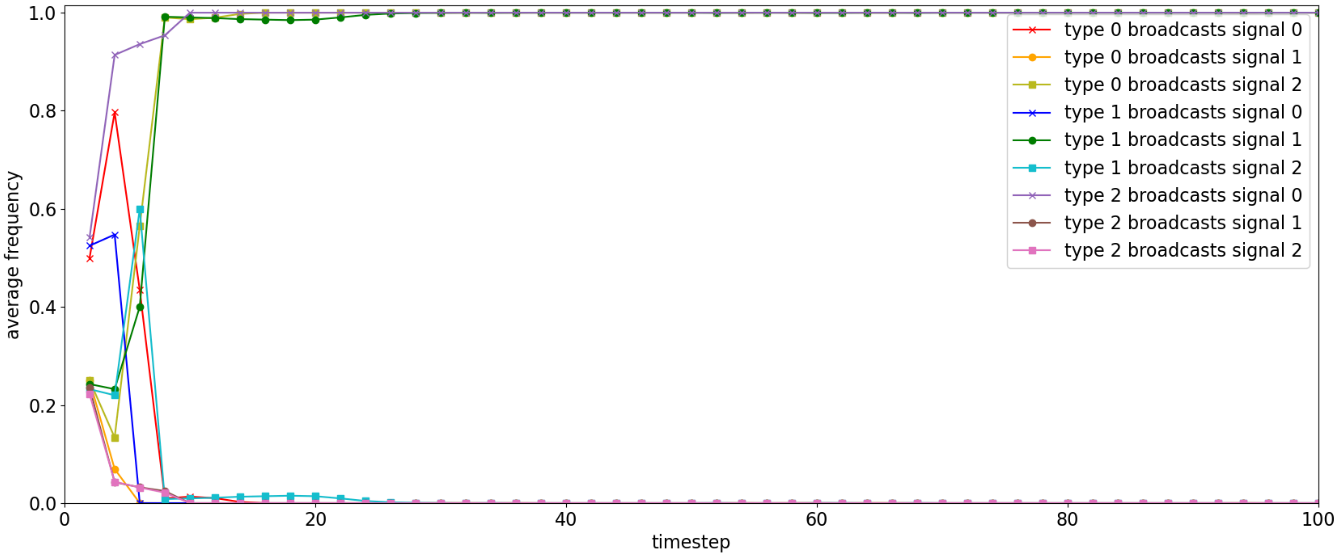

average frequency
timestep
type 0 broadcasts signal 0
type 0 broadcasts signal 1
type 0 broadcasts signal 2
type 1 broadcasts signal 0
type 1 broadcasts signal 1
type 1 broadcasts signal 2
type 2 broadcasts signal 0
type 2 broadcasts signal 1
type 2 broadcasts signal 2

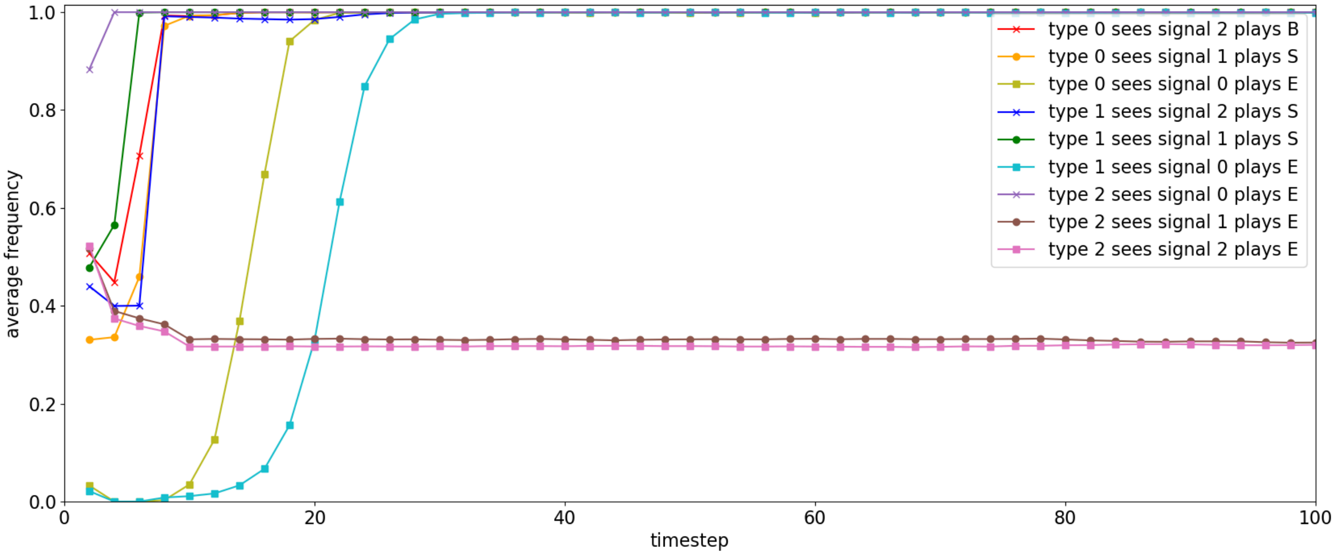

average frequency
timestep
type 0 sees signal 2 plays B
type 0 sees signal 1 plays S
type 0 sees signal 0 plays E
type 1 sees signal 2 plays S
type 1 sees signal 1 plays S
type 1 sees signal 0 plays E
type 2 sees signal 0 plays E
type 2 sees signal 1 plays E
type 2 sees signal 2 plays E

Run # 82

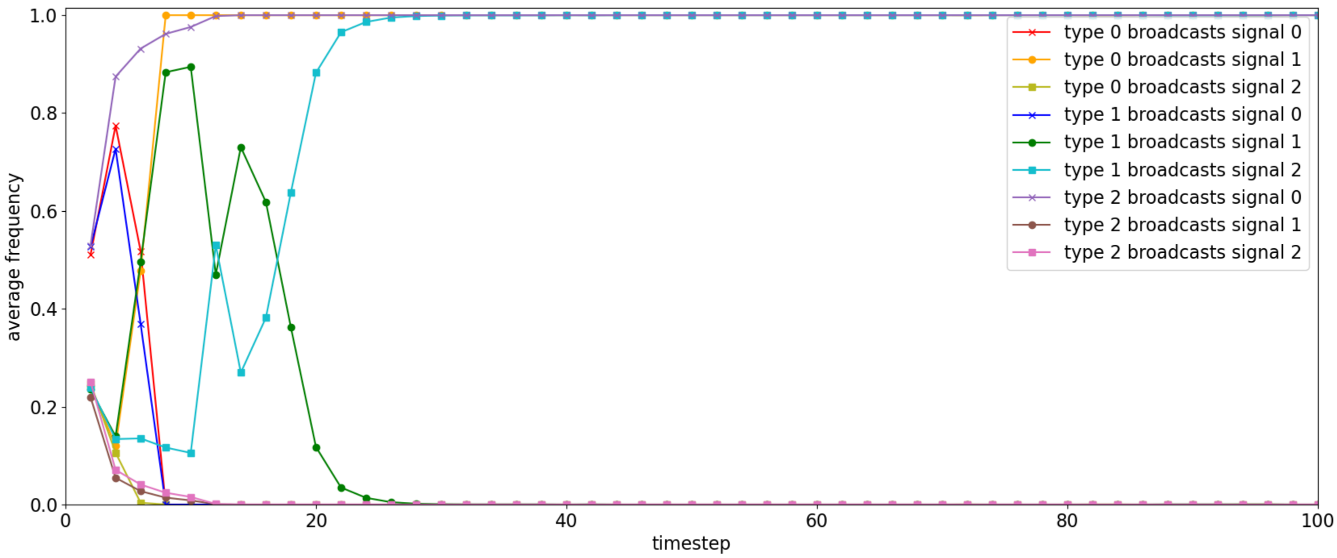

average frequency
timestep
type 0 broadcasts signal 0
type 0 broadcasts signal 1
type 0 broadcasts signal 2
type 1 broadcasts signal 0
type 1 broadcasts signal 1
type 1 broadcasts signal 2
type 2 broadcasts signal 0
type 2 broadcasts signal 1
type 2 broadcasts signal 2

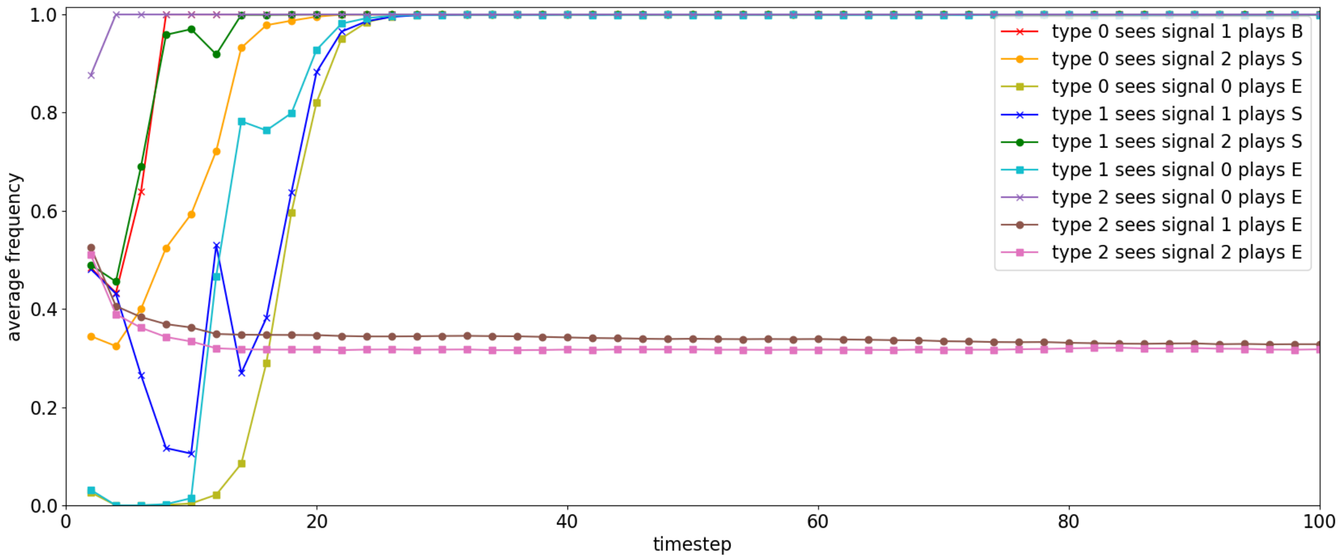

average frequency
timestep
type 0 sees signal 1 plays B
type 0 sees signal 2 plays S
type 0 sees signal 0 plays E
type 1 sees signal 1 plays S
type 1 sees signal 2 plays S
type 1 sees signal 0 plays E
type 2 sees signal 0 plays E
type 2 sees signal 1 plays E
type 2 sees signal 2 plays E

outcomes (xiv):

(note: don't forget that agents who signal 0 treat all received signals as 0)

alpha = 0.1 and beta = 0.08, Run # 689

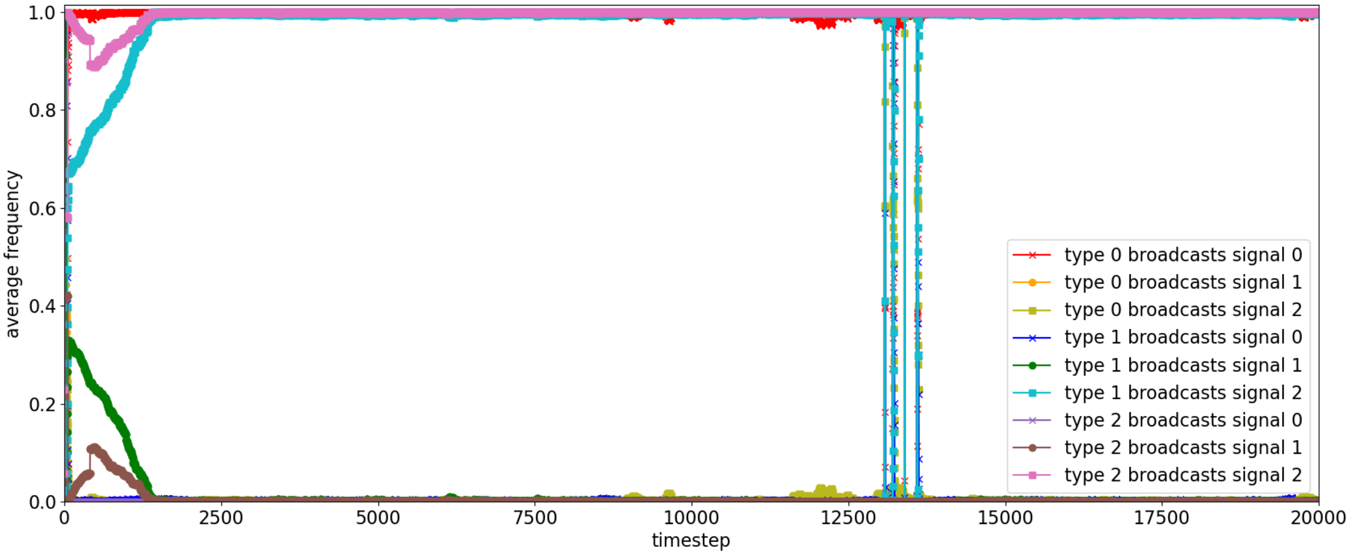

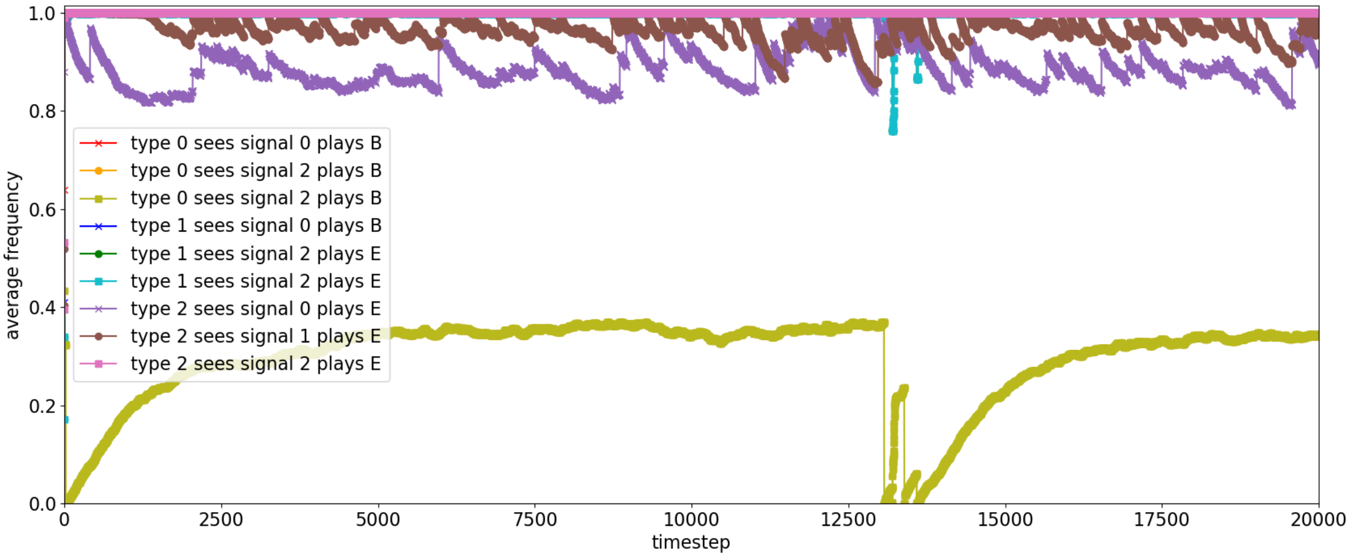

Continued on next page

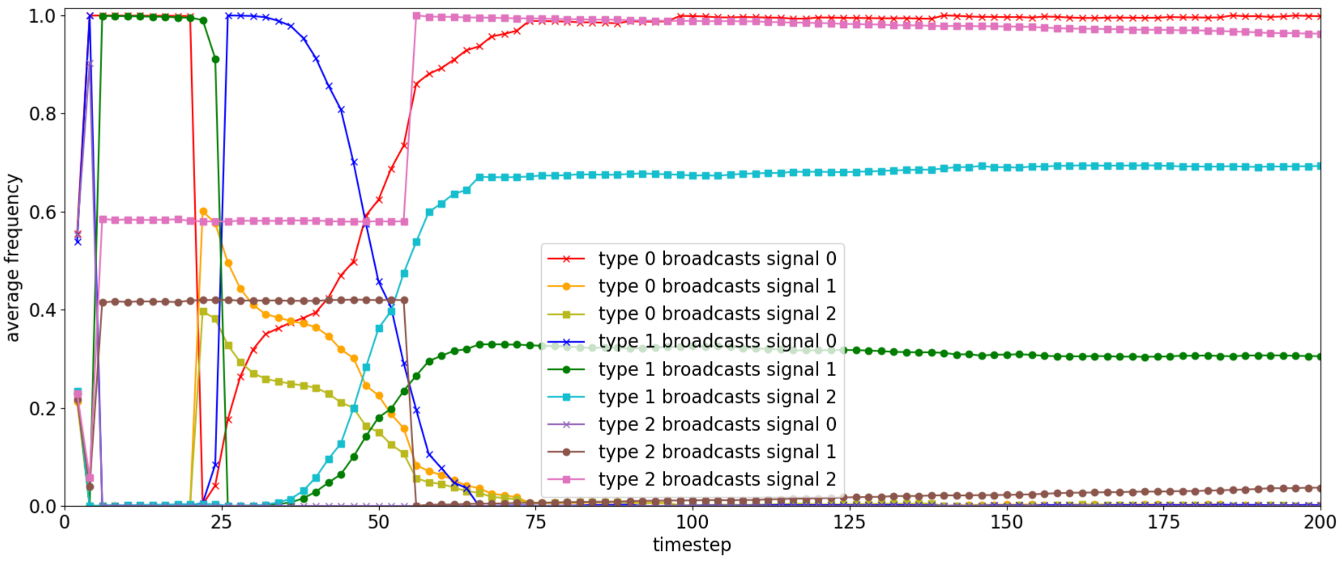

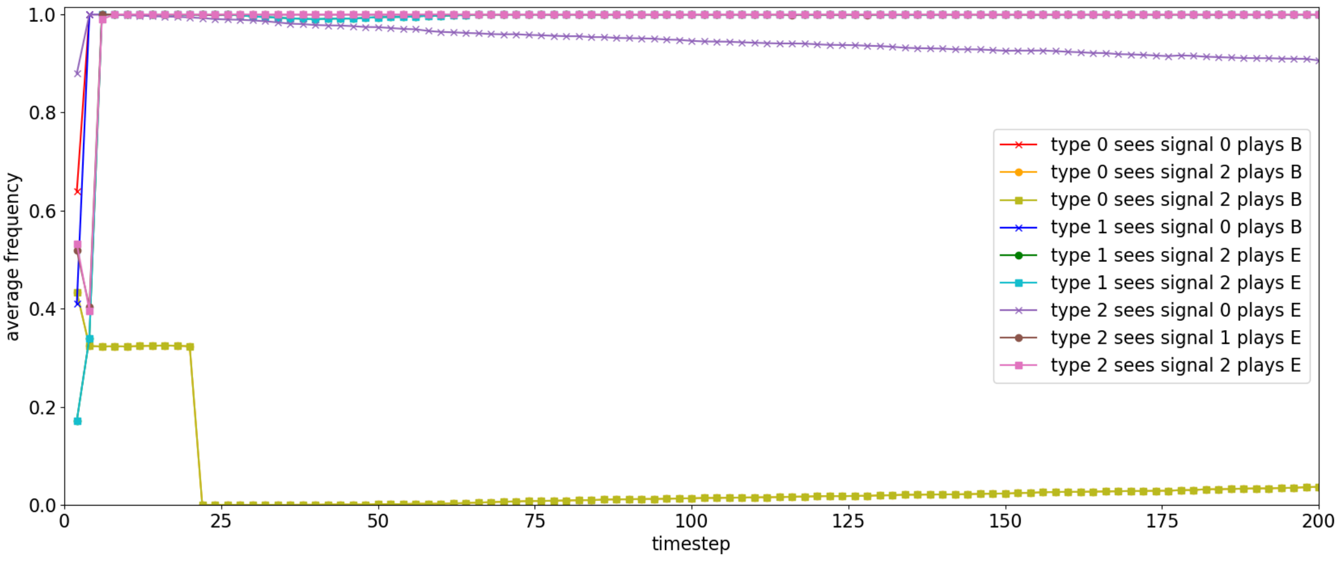

Continued on next page

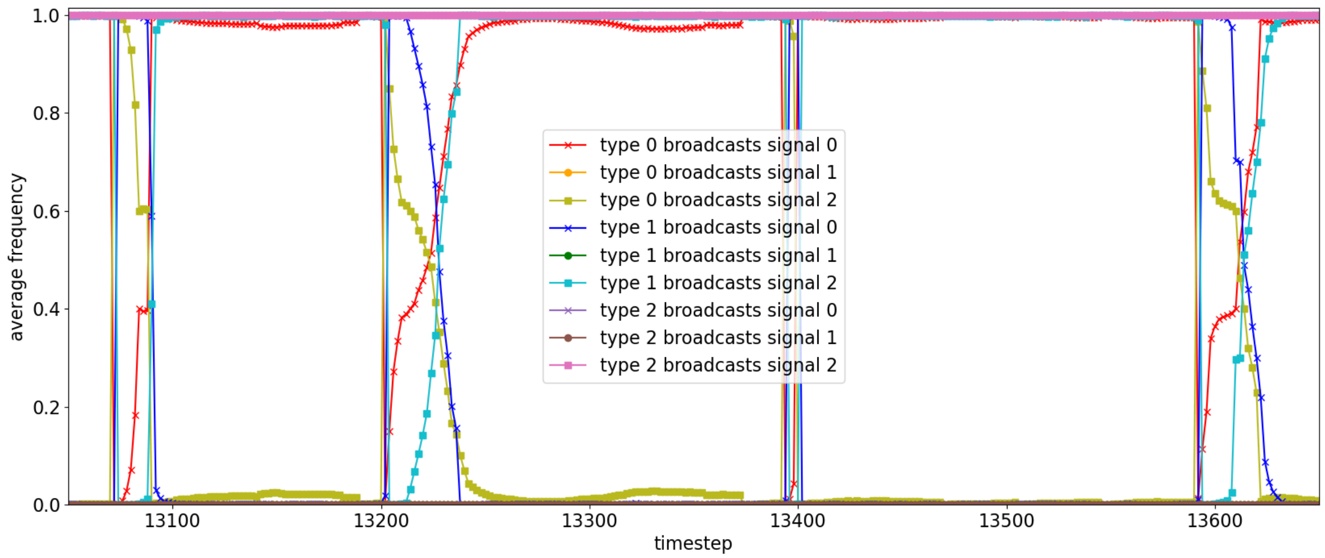

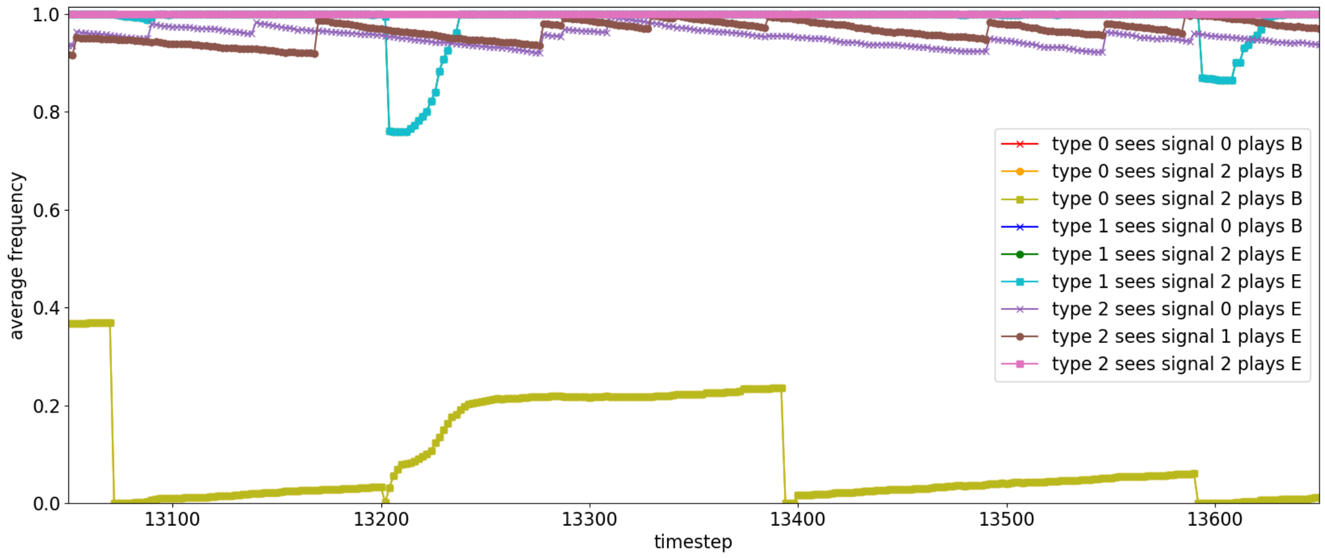

alpha = 0.2 and beta = 0.08 run # 293

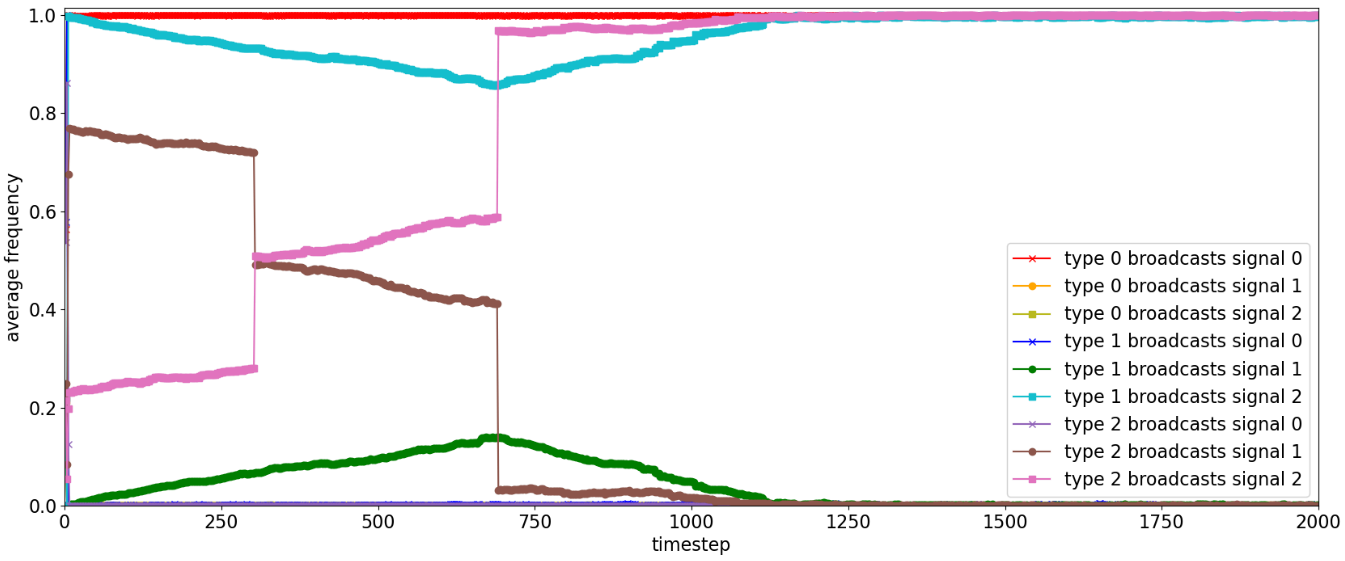

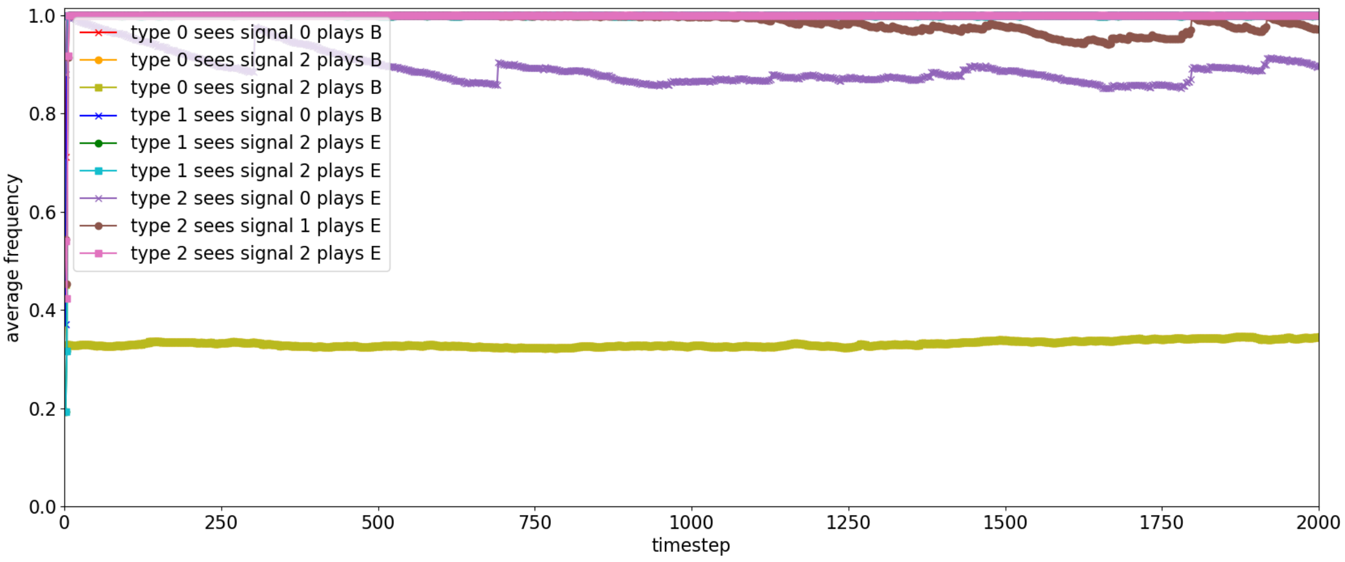

Continued on next page

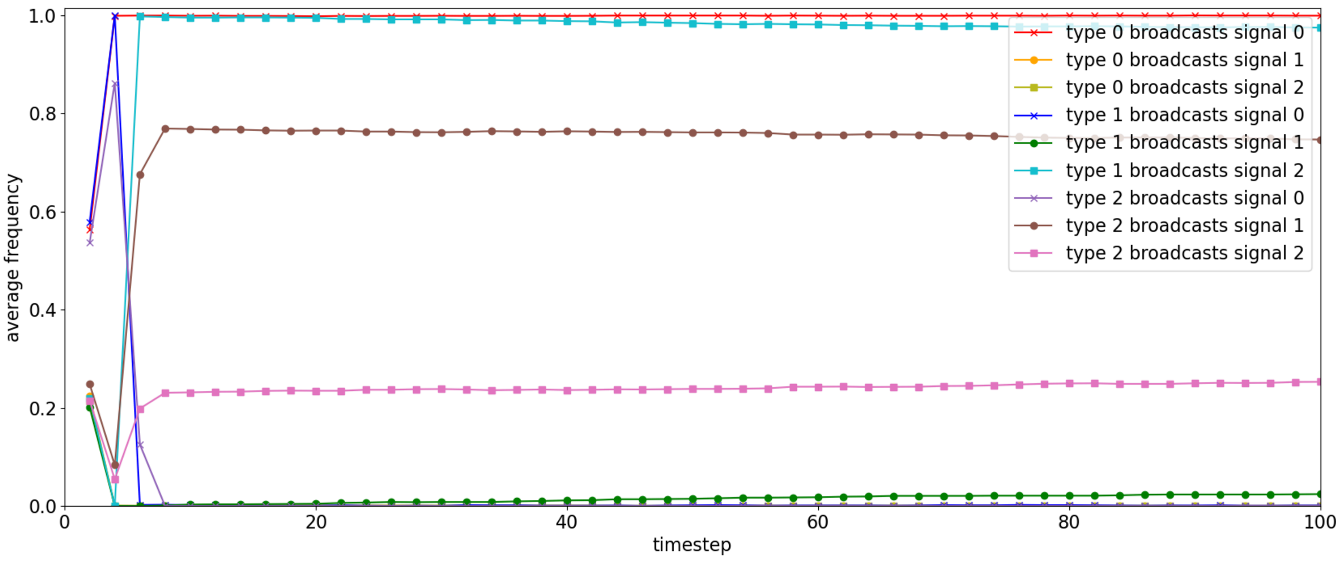

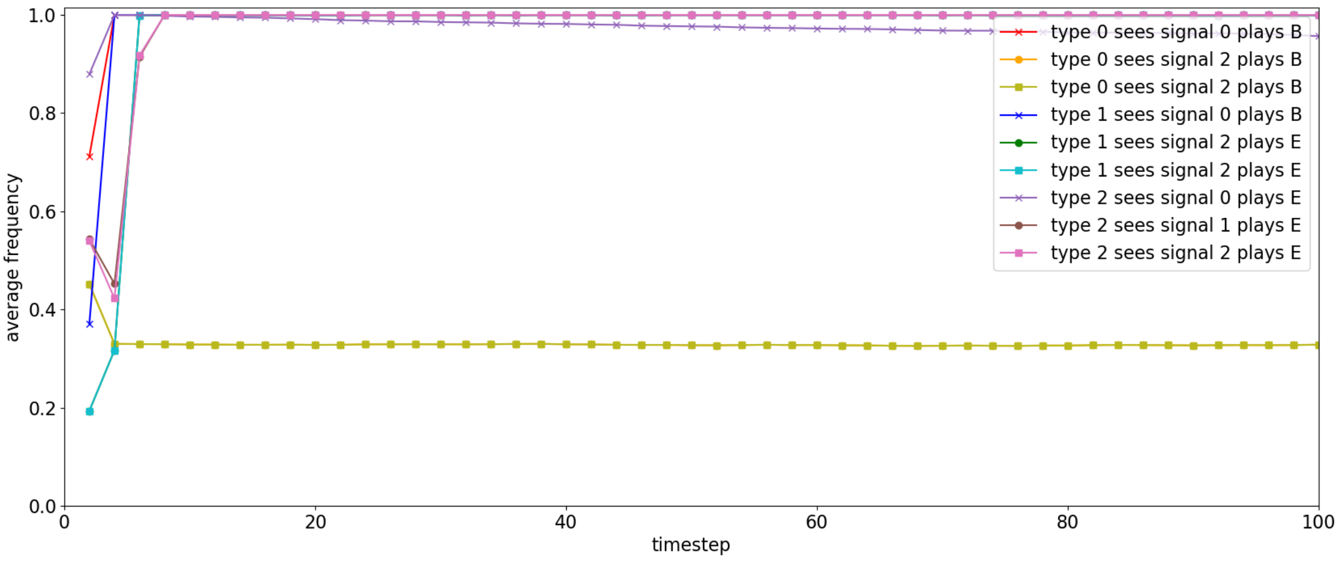

alpha = 0.2 and beta = 0.08 run # 304

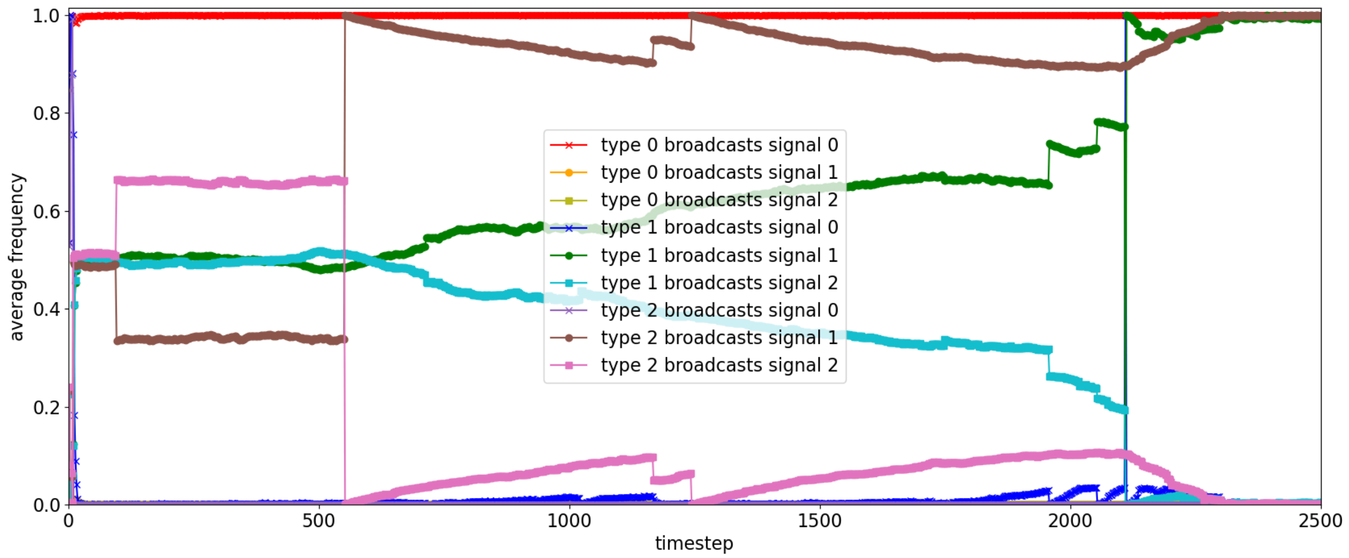

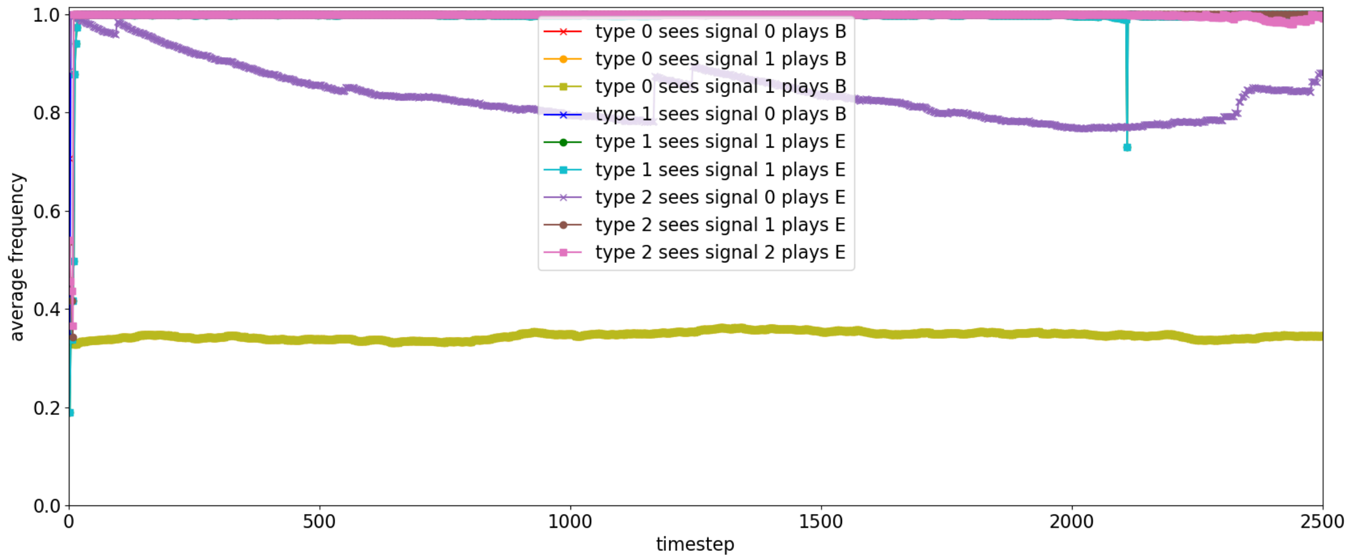

Continued on next page

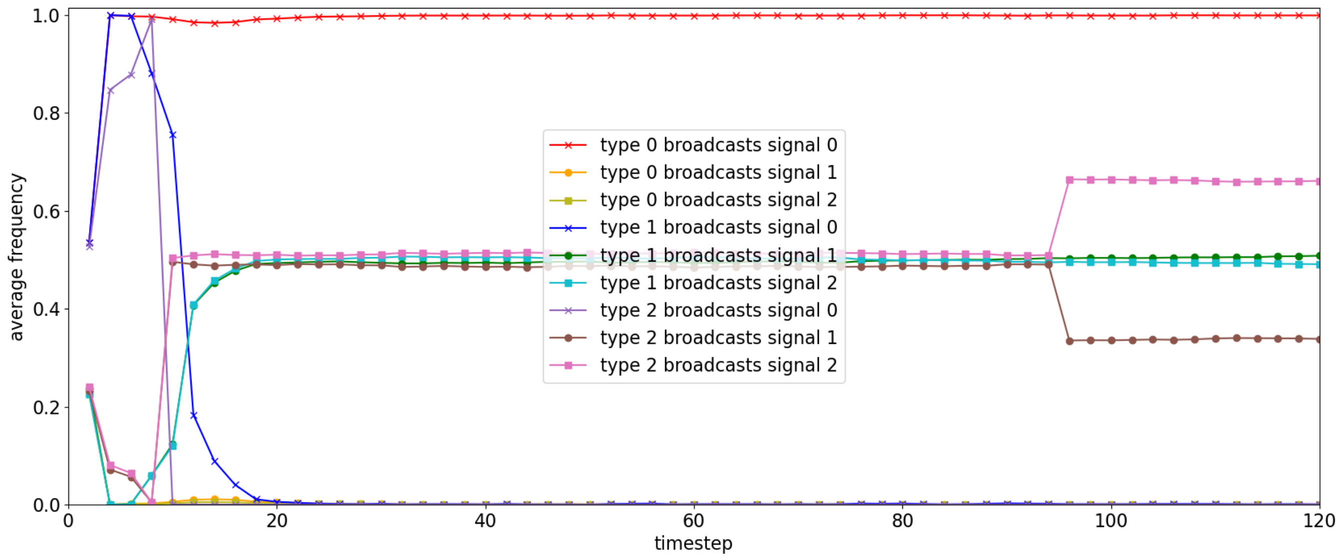

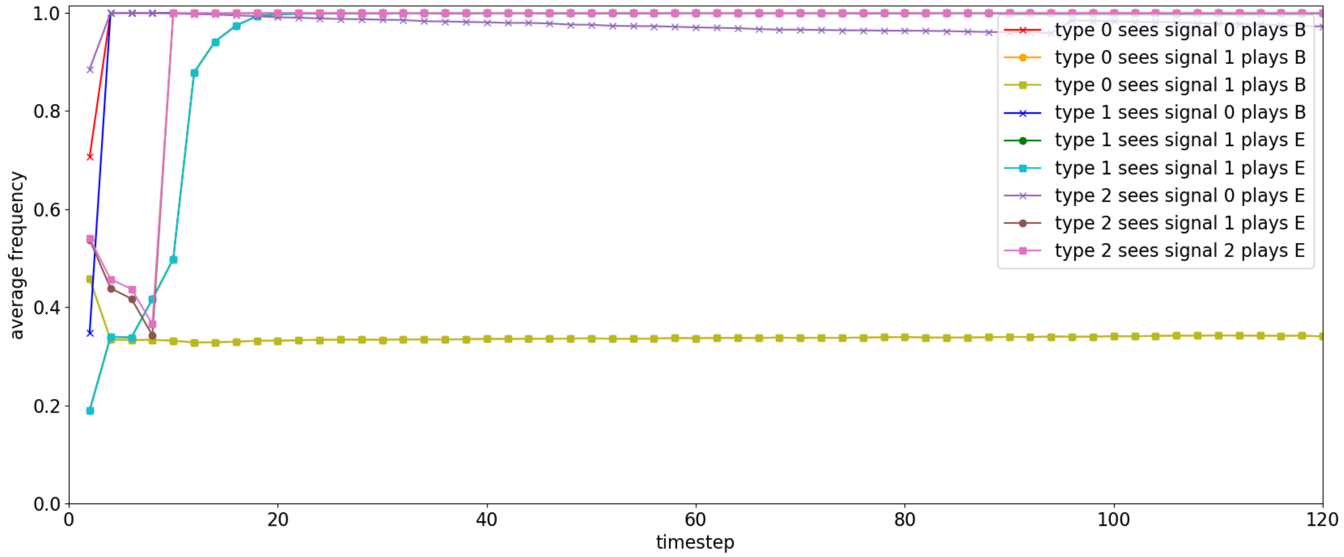

alpha = 0.2 and beta = 0.1 run # 497

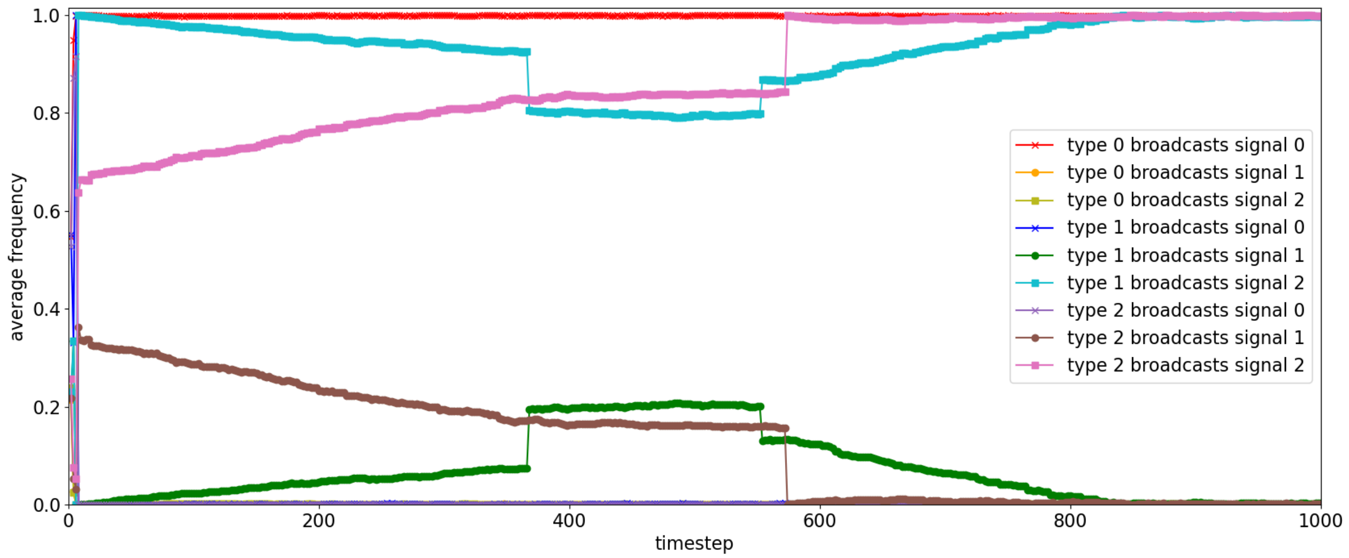

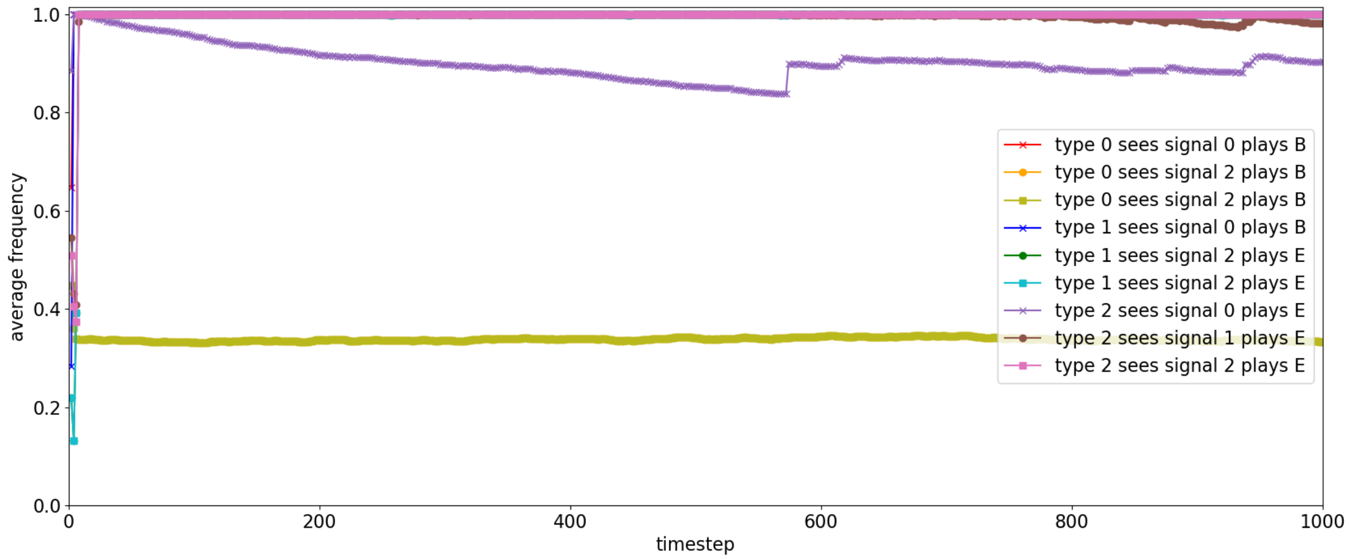

Continued on next page

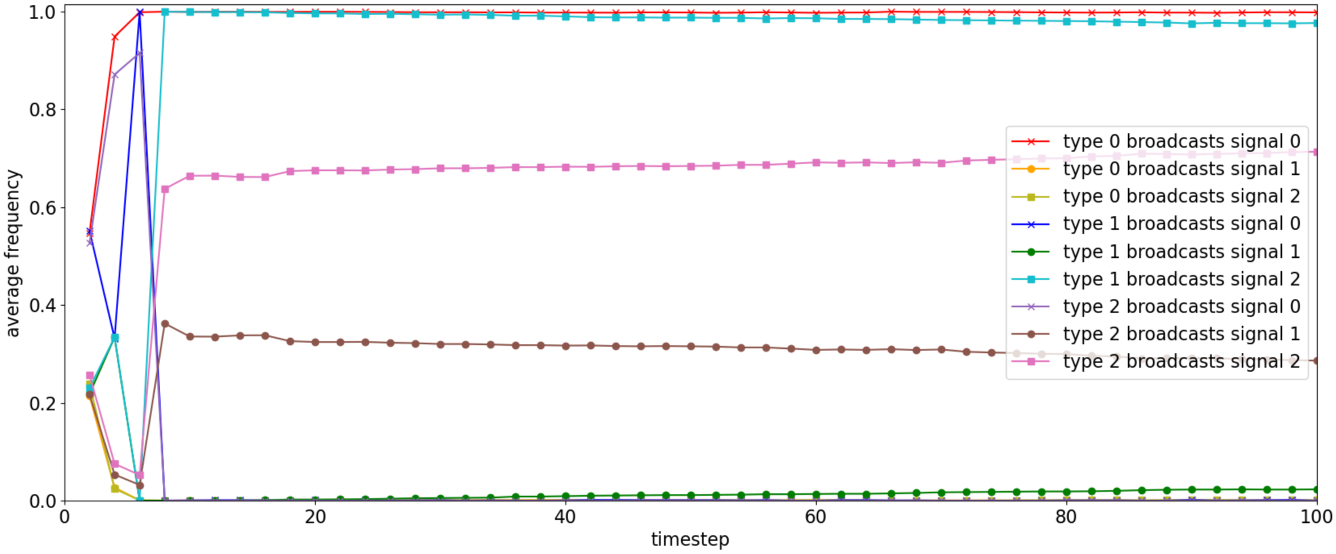
average frequency
1.0
0.8
0.6
0.4
0.2
0.0
0
20
40
60
80
100
timestep
type 0 broadcasts signal 0
type 0 broadcasts signal 1
type 0 broadcasts signal 2
type 1 broadcasts signal 0
type 1 broadcasts signal 1
type 1 broadcasts signal 2
type 2 broadcasts signal 0
type 2 broadcasts signal 1
type 2 broadcasts signal 2

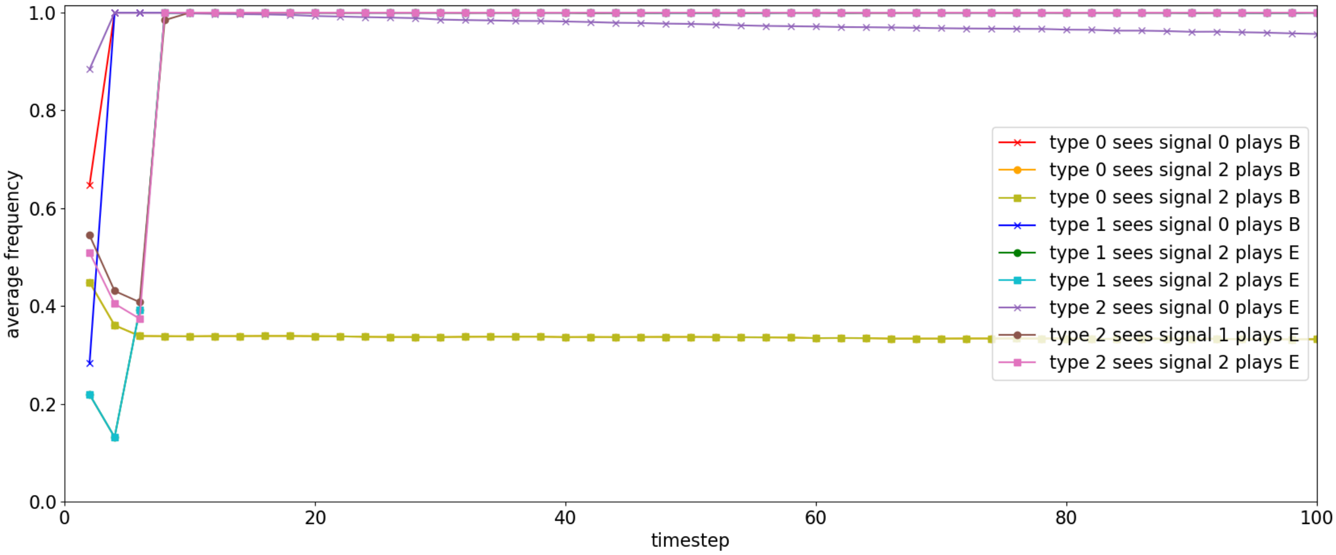
average frequency
1.0
0.8
0.6
0.4
0.2
0.0
0
20
40
60
80
100
timestep
type 0 sees signal 0 plays B
type 0 sees signal 2 plays B
type 0 sees signal 2 plays B
type 1 sees signal 0 plays B
type 1 sees signal 2 plays E
type 1 sees signal 2 plays E
type 2 sees signal 0 plays E
type 2 sees signal 1 plays E
type 2 sees signal 2 plays E

outcomes (xv):

(note: don't forget that agents who signal 0 treat all received signals as 0)

alpha = 0.3 and beta = 0, Run # 226

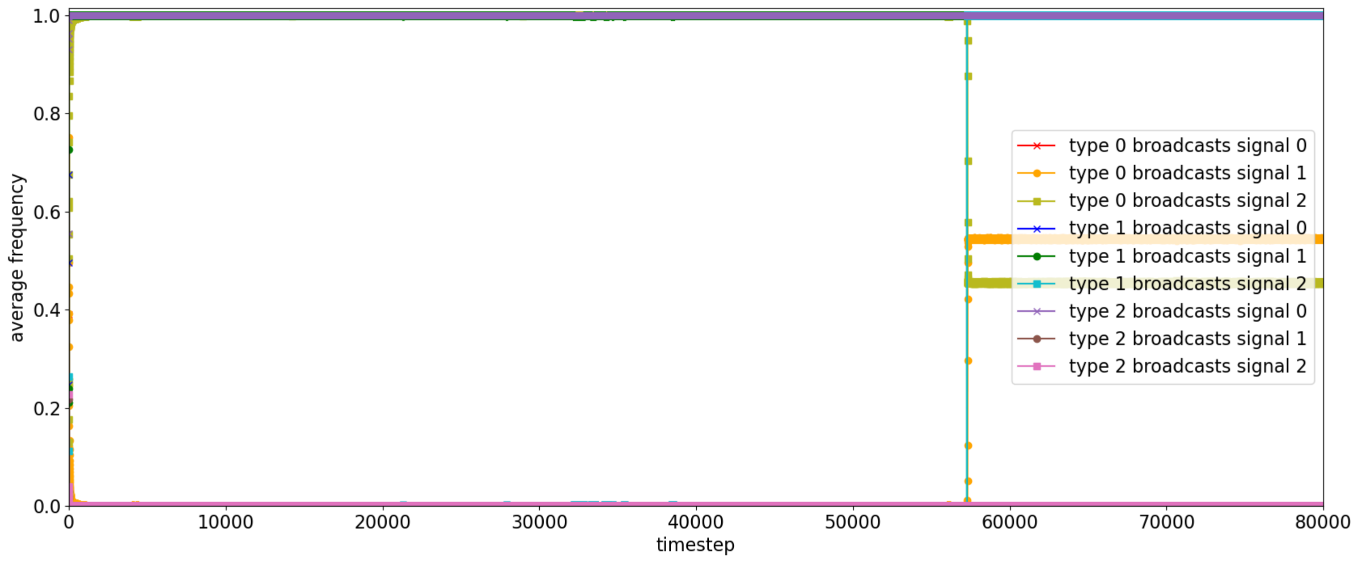

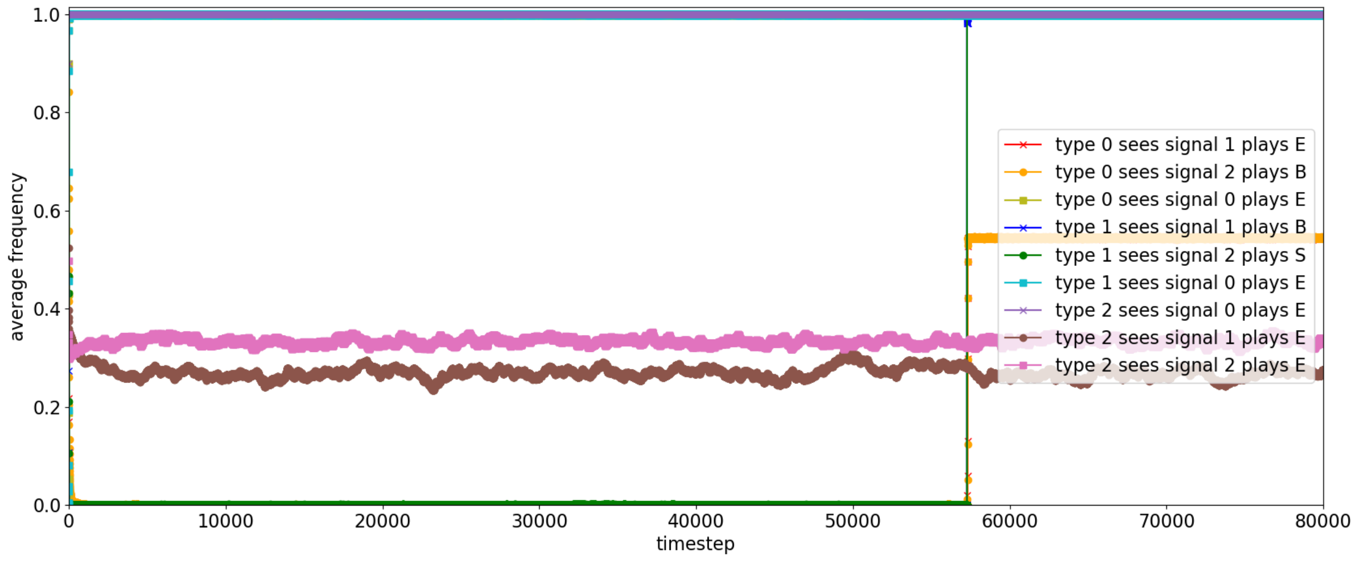

Continued on next page

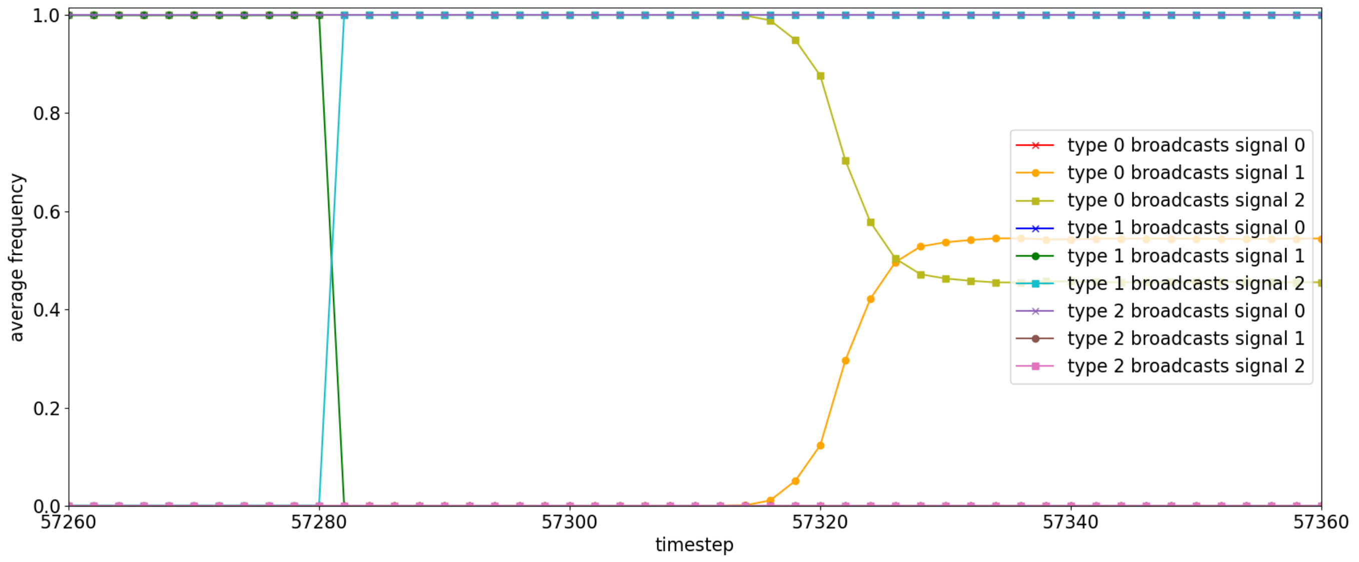
average frequency
timestep
1.0
0.8
0.6
0.4
0.2
0.0
57260
57280
57300
57320
57340
57360
type 0 broadcasts signal 0
type 0 broadcasts signal 1
type 0 broadcasts signal 2
type 1 broadcasts signal 0
type 1 broadcasts signal 1
type 1 broadcasts signal 2
type 2 broadcasts signal 0
type 2 broadcasts signal 1
type 2 broadcasts signal 2

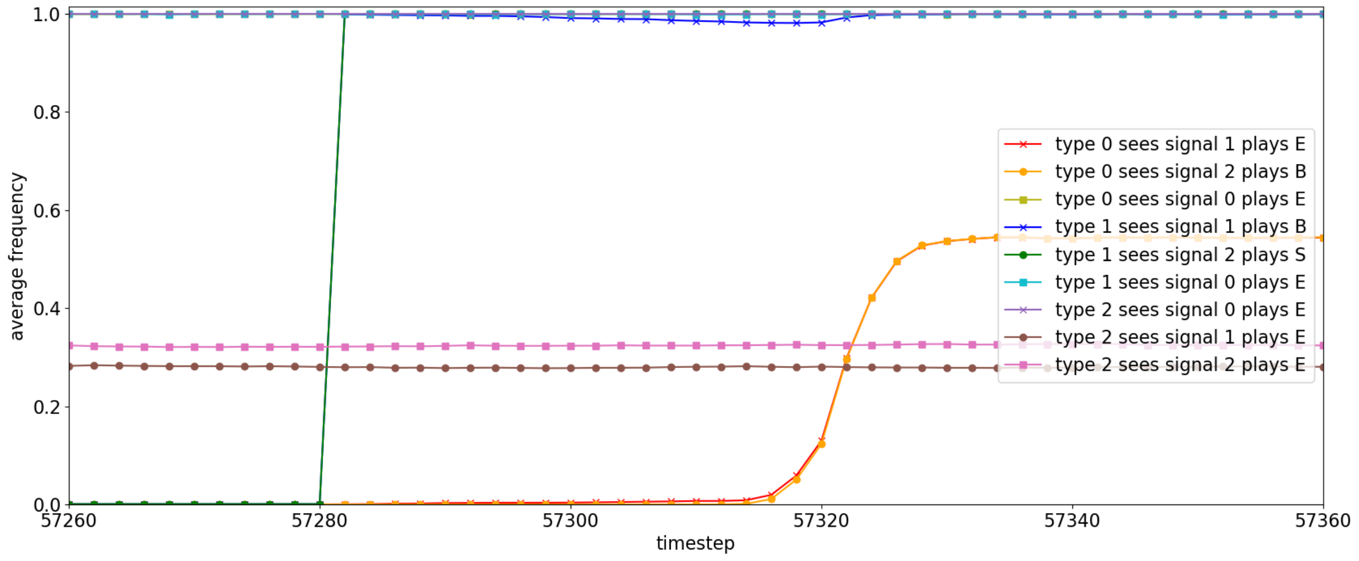
average frequency
timestep
1.0
0.8
0.6
0.4
0.2
0.0
57260
57280
57300
57320
57340
57360
type 0 sees signal 1 plays E
type 0 sees signal 2 plays B
type 0 sees signal 0 plays E
type 1 sees signal 1 plays B
type 1 sees signal 2 plays S
type 1 sees signal 0 plays E
type 2 sees signal 0 plays E
type 2 sees signal 1 plays E
type 2 sees signal 2 plays E

First type 0 sends signal 2 and type 1 sends 1. Additionally, type 0 plays E on signal 1 and type 1 plays B on signal 1. So, type 1 can unilaterally benefit from switching to both signaling 2 and playing S on signal 2 since they were already failing to coordinate with the type 0s all of the time. But now signal 2 is noisy because both types 0 and 1 are using it. Now, it just so happens that the mutation leading to type 0s signaling 1 co-occurs with the mutations to play E on signal 1 and B on signal 2. This leaves type 0's with two symbiotic strategy profiles that depend on each other:

	broadcast signal	play when sees 0	play when sees 1	play when sees 2
first profile	2	E	B	S
second profile	1	E	E	B
hypothetical profile	1	E	B	B

Since the second profile plays B (type 0's preferred action) with agents who signal 2, further increasing the proportion of agents who have the second profile decreases its benefit since this entails that there are fewer agents who signal 2. Likewise, the first profile plays B with agents who signal 1, so further increasing the proportion of agents with the first profile decreases the benefit of the first profile because that entails there being fewer agents who signal 1. Holding all else fixed, one can check that the first and second strategy profiles have equal expected utility when there are 1500 agents with the first profile and 1800 agents with the second profile (see spreadsheet named "a3b0_run226").

But why does the second profile not mutate to playing B on signal 1? Well holding the final state of the simulation fixed, with 1500 agents with the first profile and 1800 agents with the second profile (in reality there were only 1797 agents with the second profile due to mutation), one can calculate that it would require 501 type 0 agents simultaneously switching from the second profile to the hypothetical profile for the hypothetical profile to have greater expected utility than the second profile (see spreadsheet named "a3b0_run226").

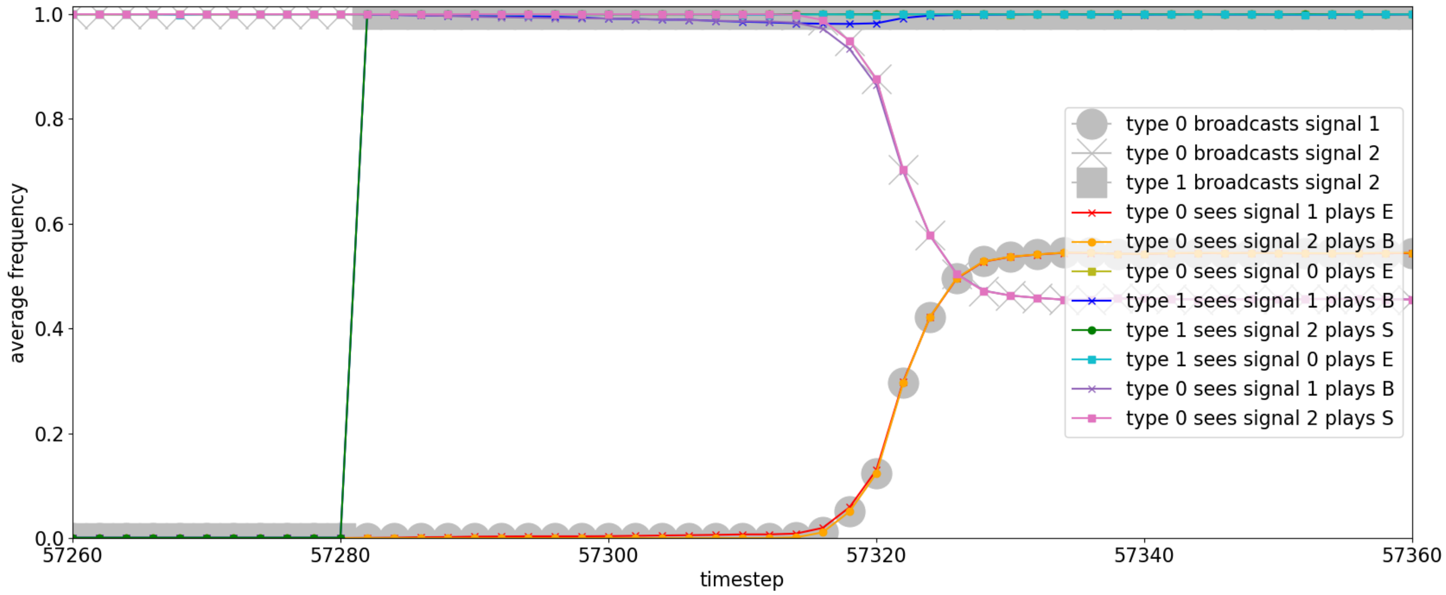

alpha = 0.4 and beta = 0, Run # 237 (looks like similar explanation to above)

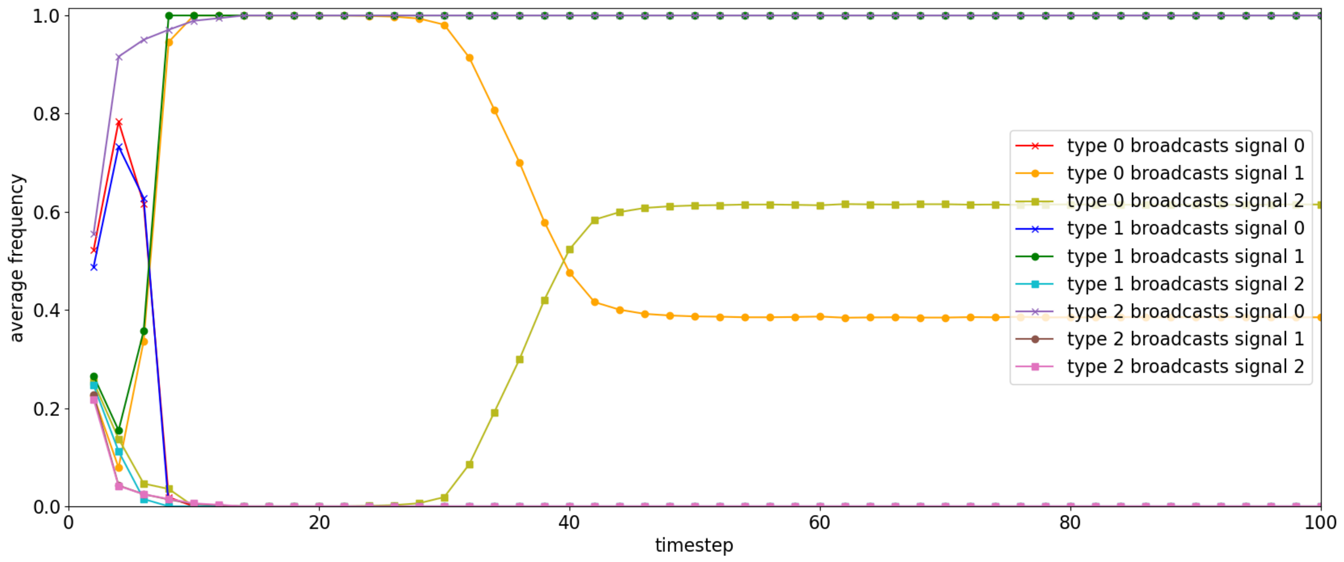

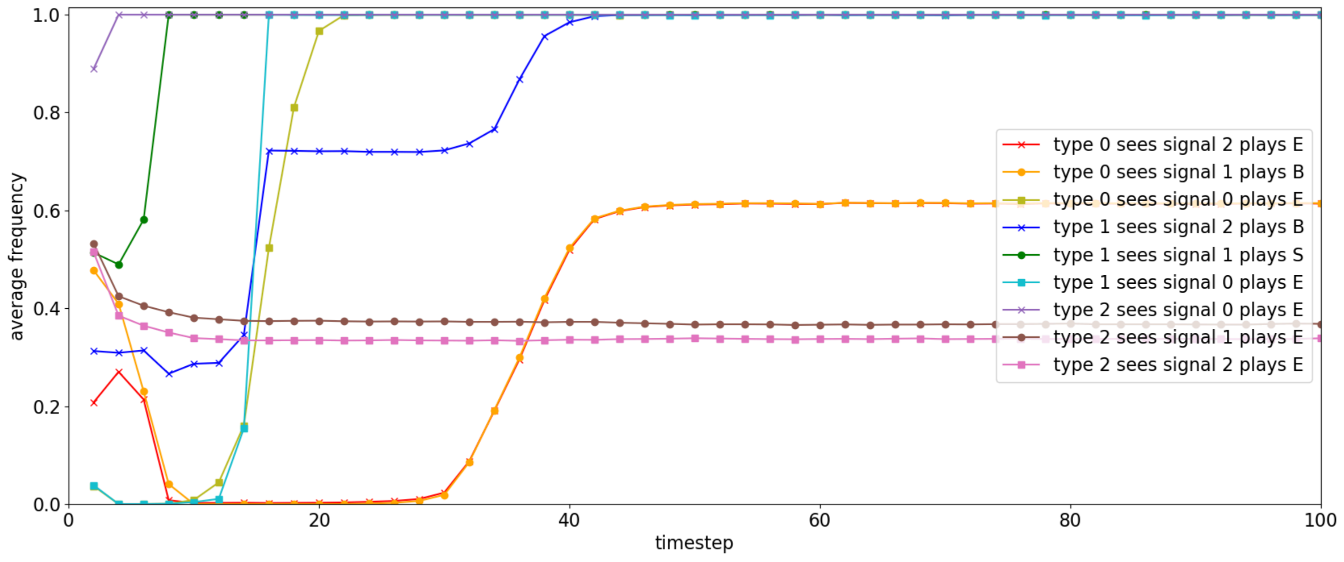

outcomes (xvi):

(note: don't forget that agents who signal 0 treat all received signals as 0)

alpha = 0.3 and beta = 0.04, Run # 882

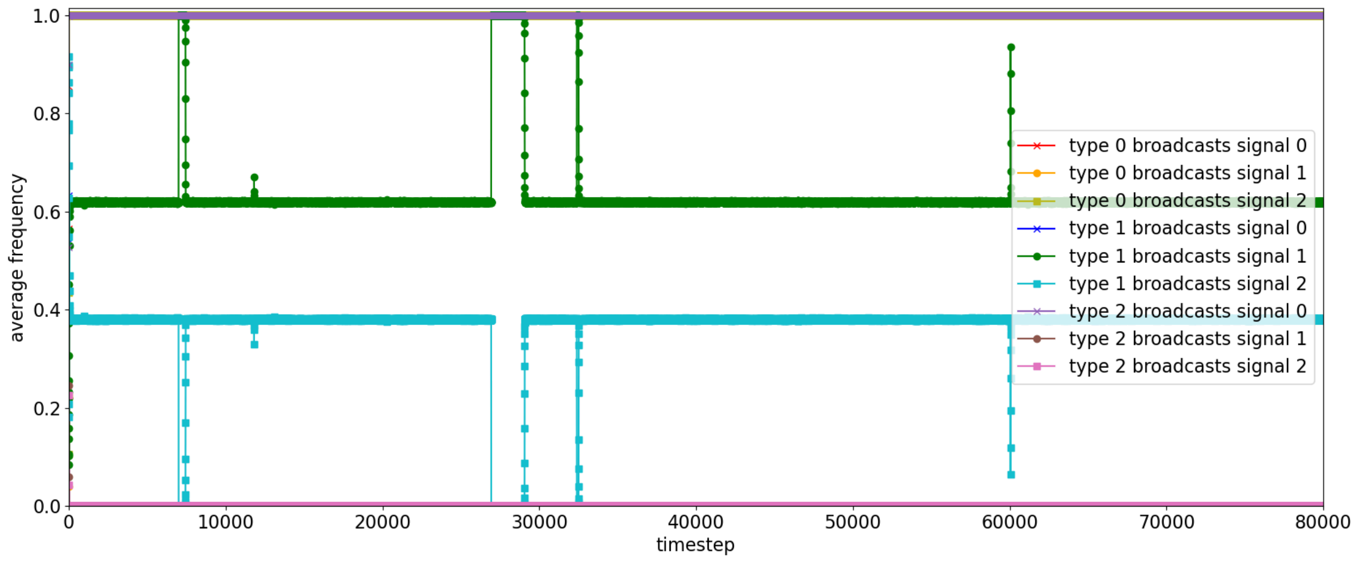

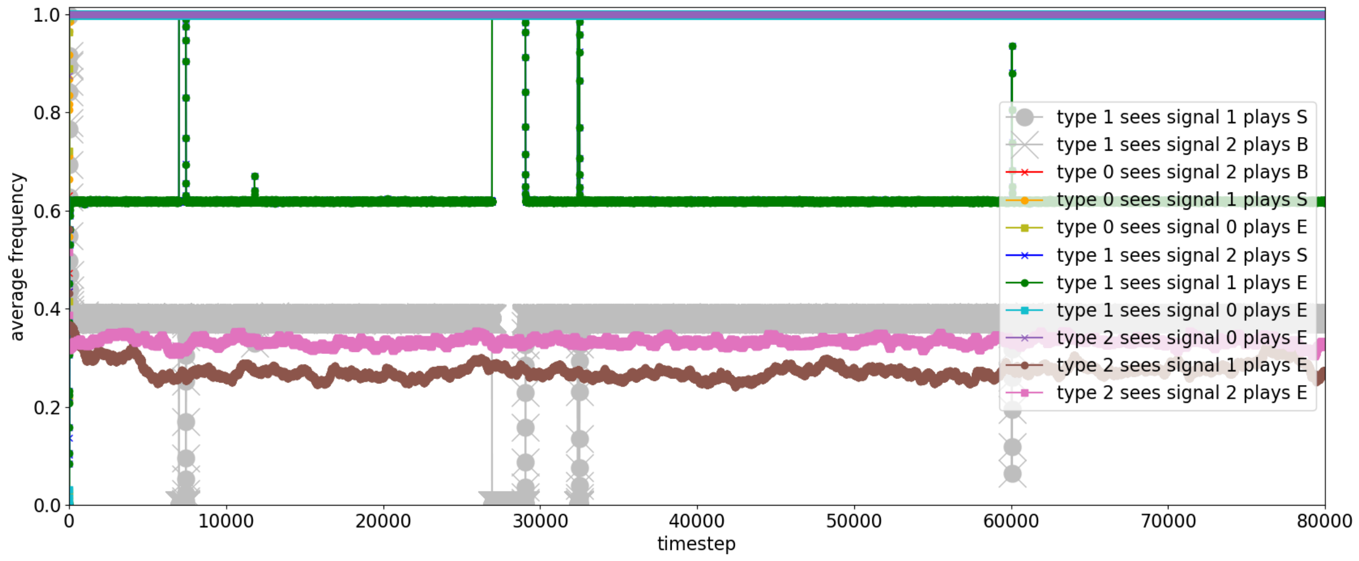

Continued on next page

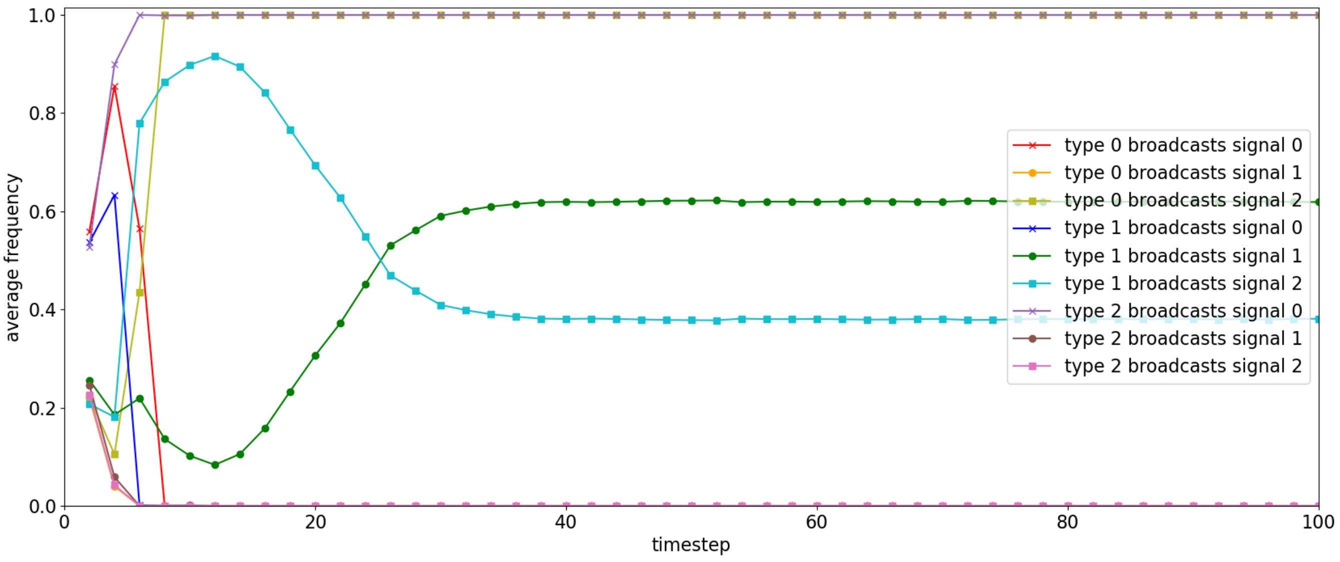

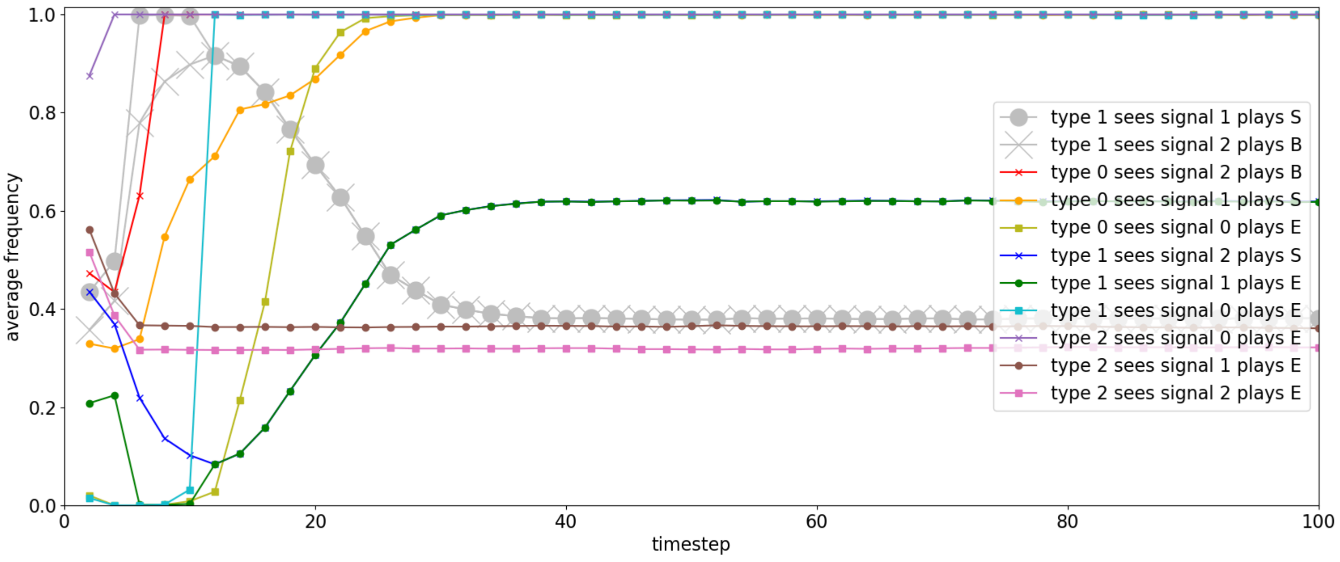

Continued on next page

	broadcast signal	play when sees 0	play when sees 1	play when sees 2	number of agents with this profile
minority profile	2	E	S	B	1100
dominant profile	1	E	E	S	1800
hypothetical profile					0
	B	S	E		
type 1 coordination preferences:	1	1.3	0.5		
total number of agents in population who		signal	play on signal 0	play on signal 1	play on signal 2
type 0	3700	2	E	S	B
type 1	2900		E		
type 2	3400	0	E	E	E

Confirmed that for this distribution of agents the two strategy profiles employed by type 1 agents have equal expected utility and for each profile increasing its proportion lowers the expected utility for that profile (see spreadsheet titled "a3b2_run882").

alpha = 0.4 and beta = 0.02 run # 86

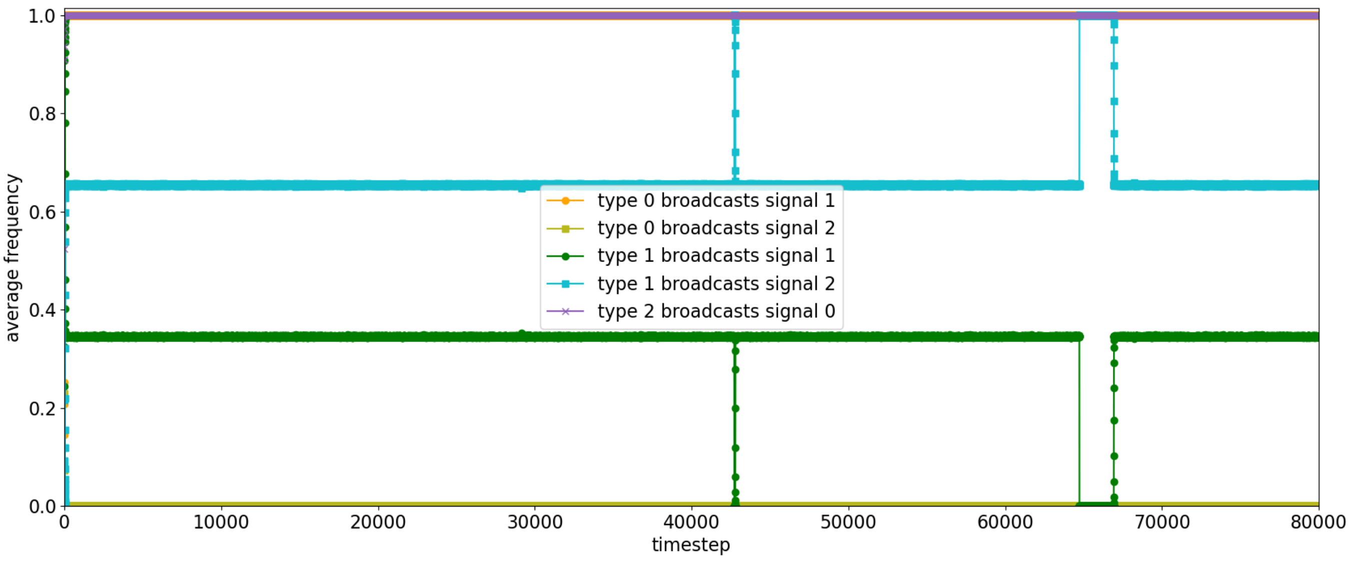

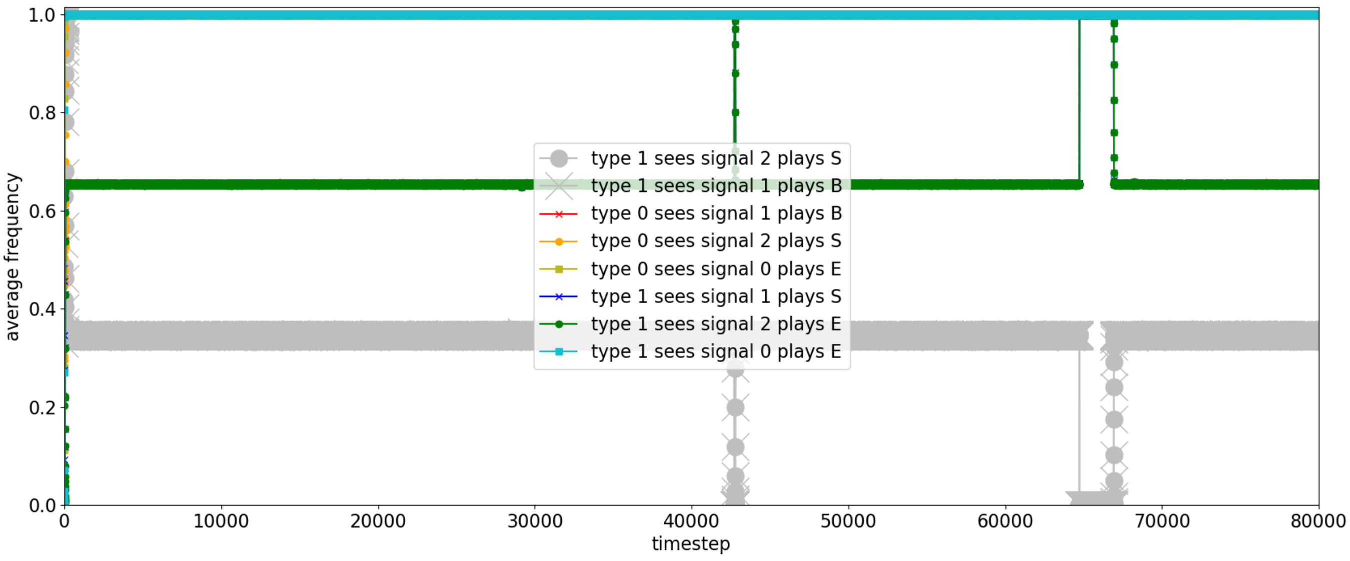

Continued on the next page

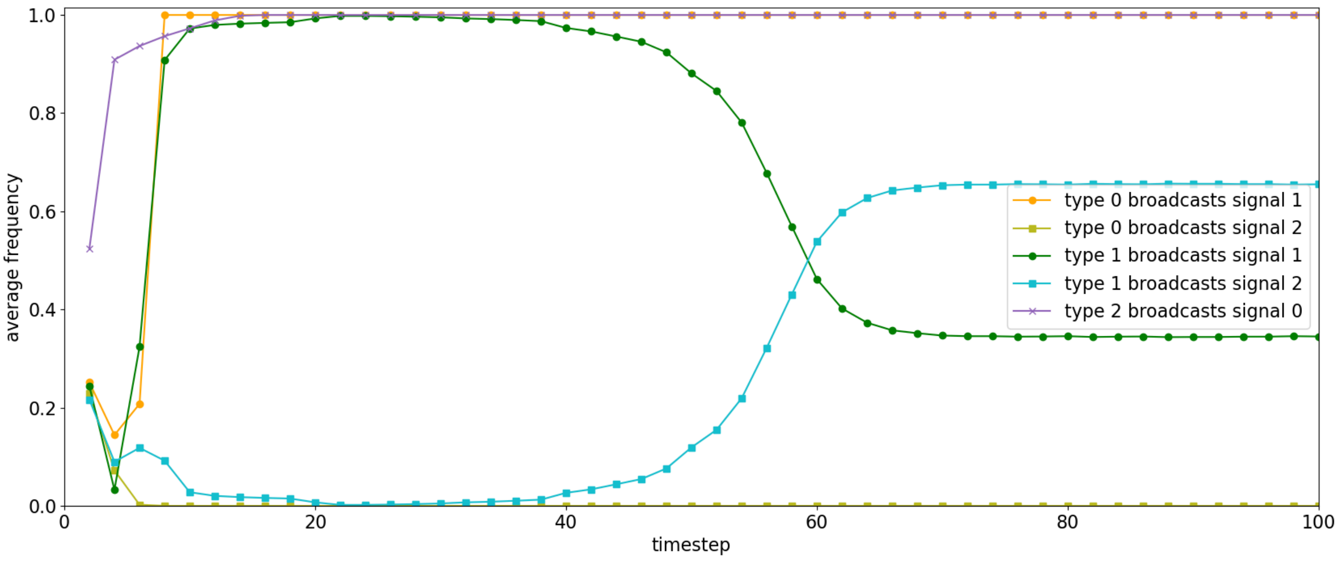

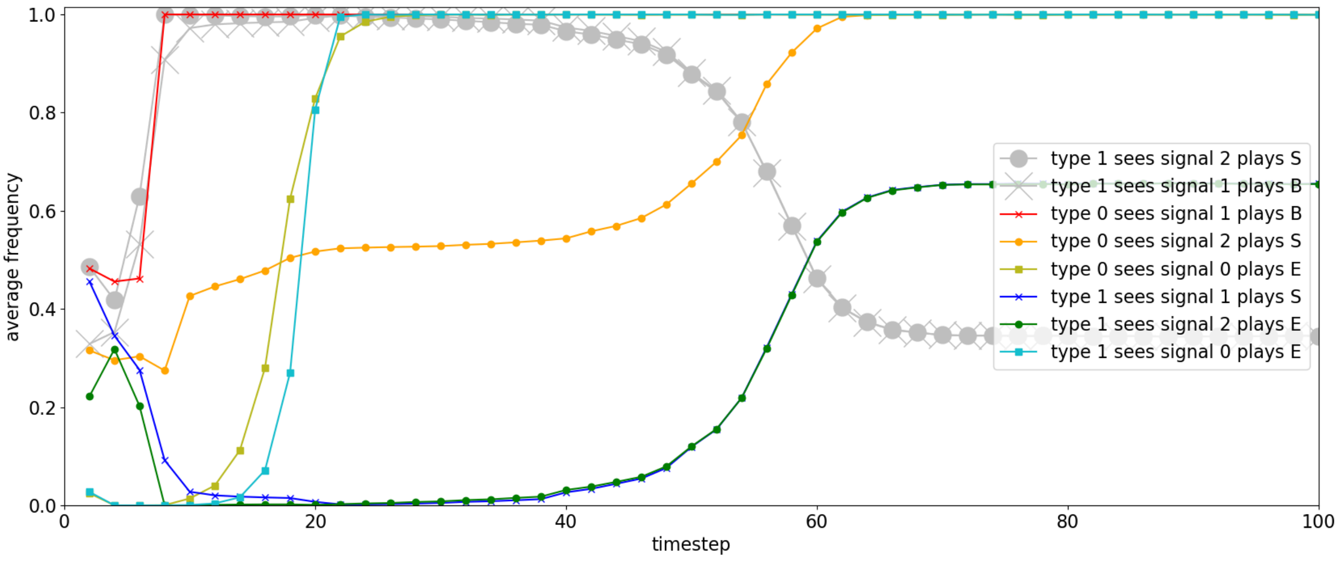

Continued on next page

	broadcast signal	play when sees 0	play when sees 2	play when sees 1	number of agents with this profile
minority profile	1	E	S	B	1068
dominant profile	2	E	E	S	2031
hypothetical profile					0

	B	S	E
type 1 coordination preferences:	1	1.4	0.5

total number of agents in population who		signal	play on signal 0	play on signal 2	play on signal 1
type 0	3501	1	E	S	B
type 1	3099		E		
type 2	3400	0	E	E	E

EU for minority	9112.4
EU for dominant	9112.1

See spreadsheet named “a4b1_run86”

alpha = 0.4 and beta = 0.02 run # 179

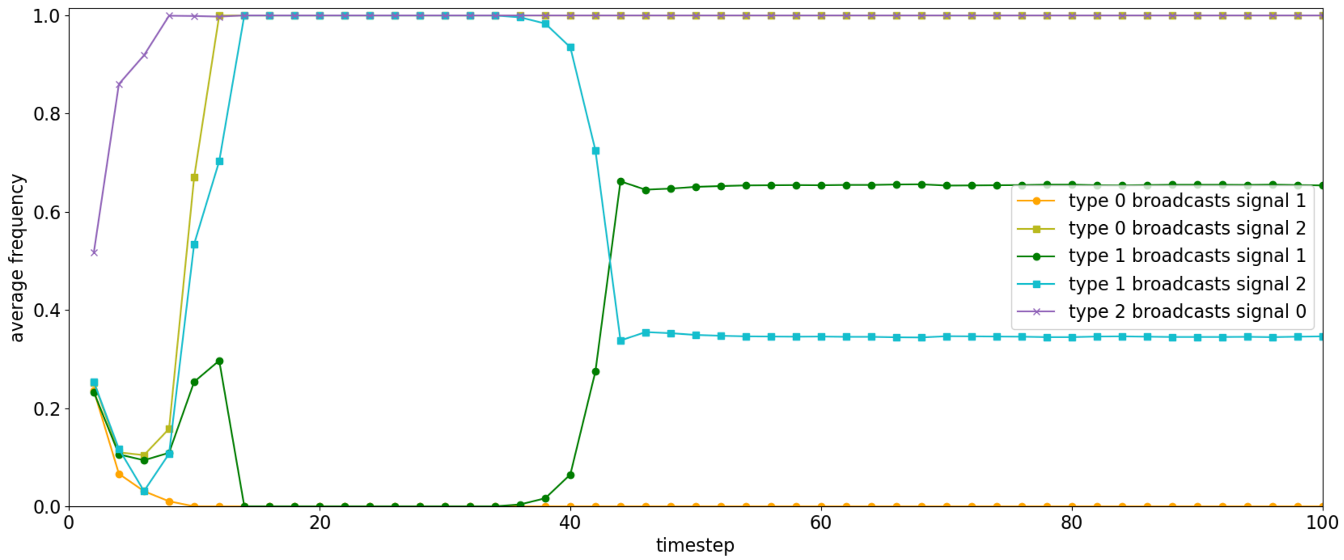

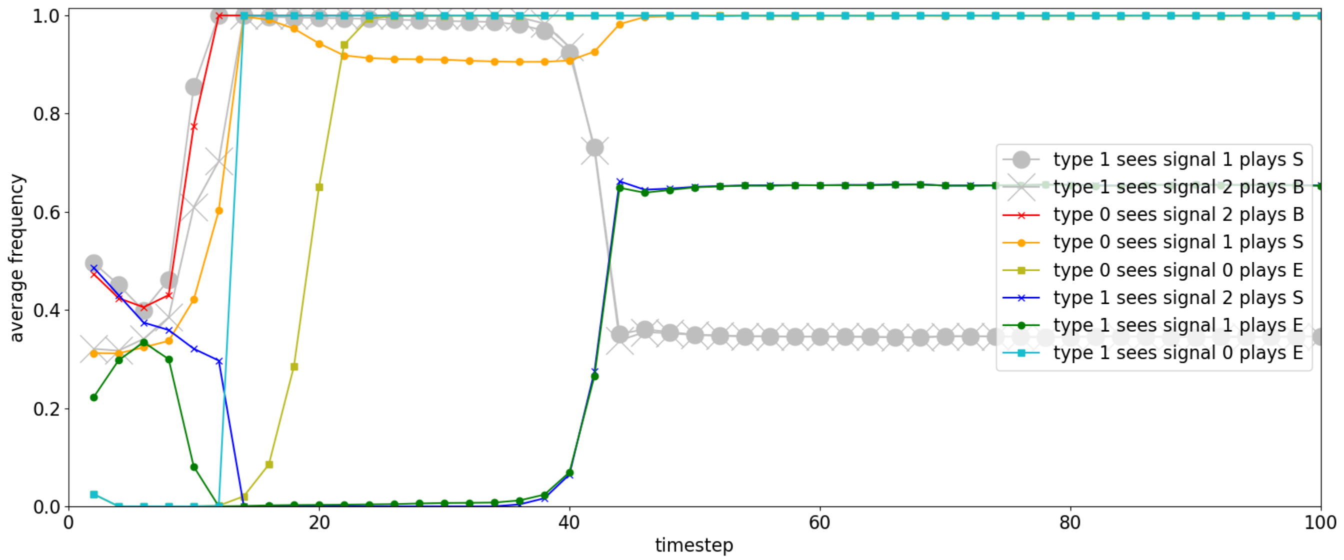

alpha = 0.5 and beta = 0 run # 3

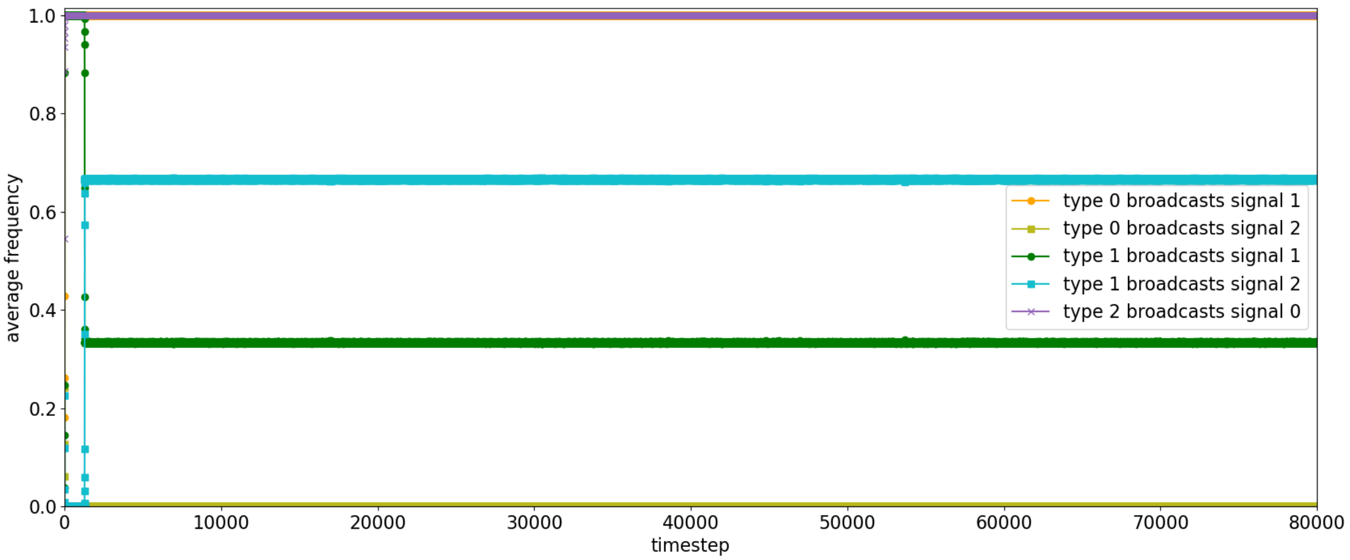

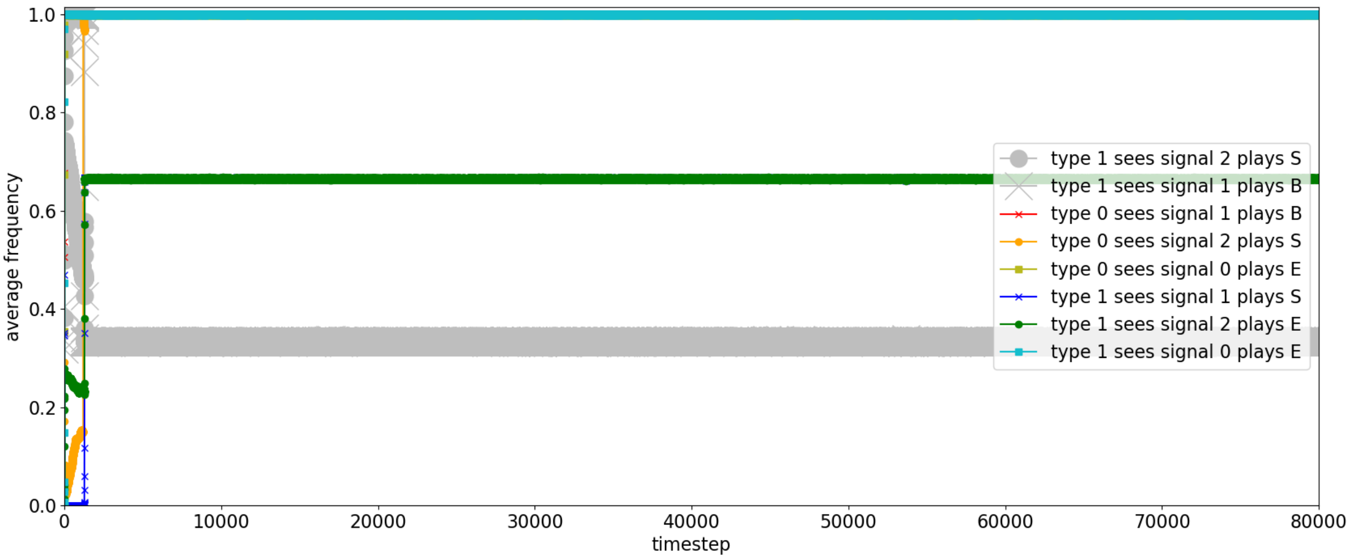

Continued

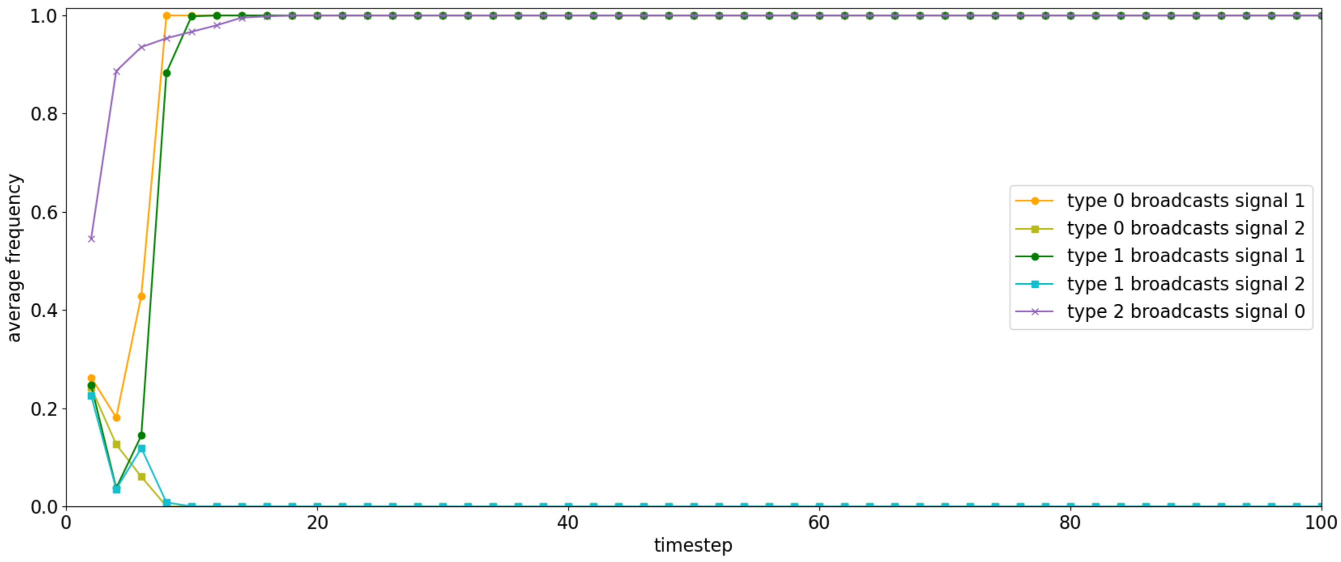
average frequency
timestep
type 0 broadcasts signal 1
type 0 broadcasts signal 2
type 1 broadcasts signal 1
type 1 broadcasts signal 2
type 2 broadcasts signal 0
0.0
0.2
0.4
0.6
0.8
1.0
0
20
40
60
80
100

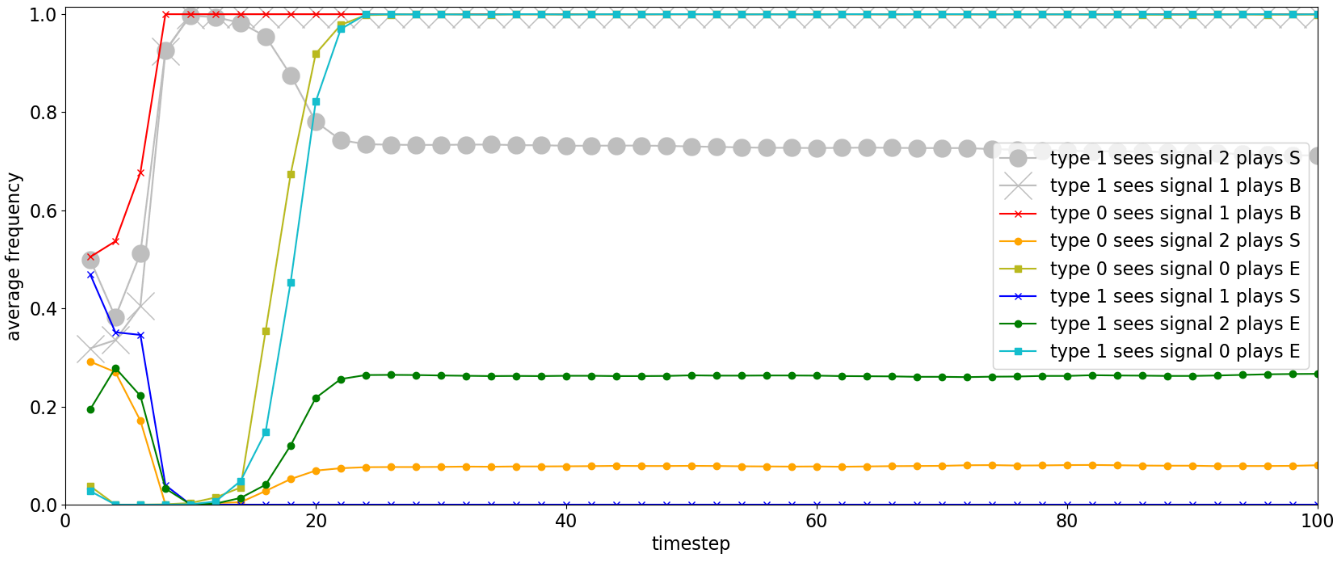
average frequency
timestep
type 1 sees signal 2 plays S
type 1 sees signal 1 plays B
type 0 sees signal 1 plays B
type 0 sees signal 2 plays S
type 0 sees signal 0 plays E
type 1 sees signal 1 plays S
type 1 sees signal 2 plays E
type 1 sees signal 0 plays E
0.0
0.2
0.4
0.6
0.8
1.0
0
20
40
60
80
100

Continued

	broadcast signal	play when sees 0	play when sees 2	play when sees 1	number of agents with this profile
minority profile	1	E	S	B	1101
dominant profile	2	E	E	S	2199
hypothetical profile					0

	B	S	E
type 1 coordination preferences:	1	1.5	0.5

total number of agents in population who		signal	play on signal 0	play on signal 2	play on signal 1
type 0	3300	1	E	S	B
type 1	3300		E		
type 2	3400	0	E	E	E

EU for minority	9399.5
EU for dominant	9401

See spreadsheet titled “a5b0_run3”

alpha = 0.5 and beta = 0.02 run # 794

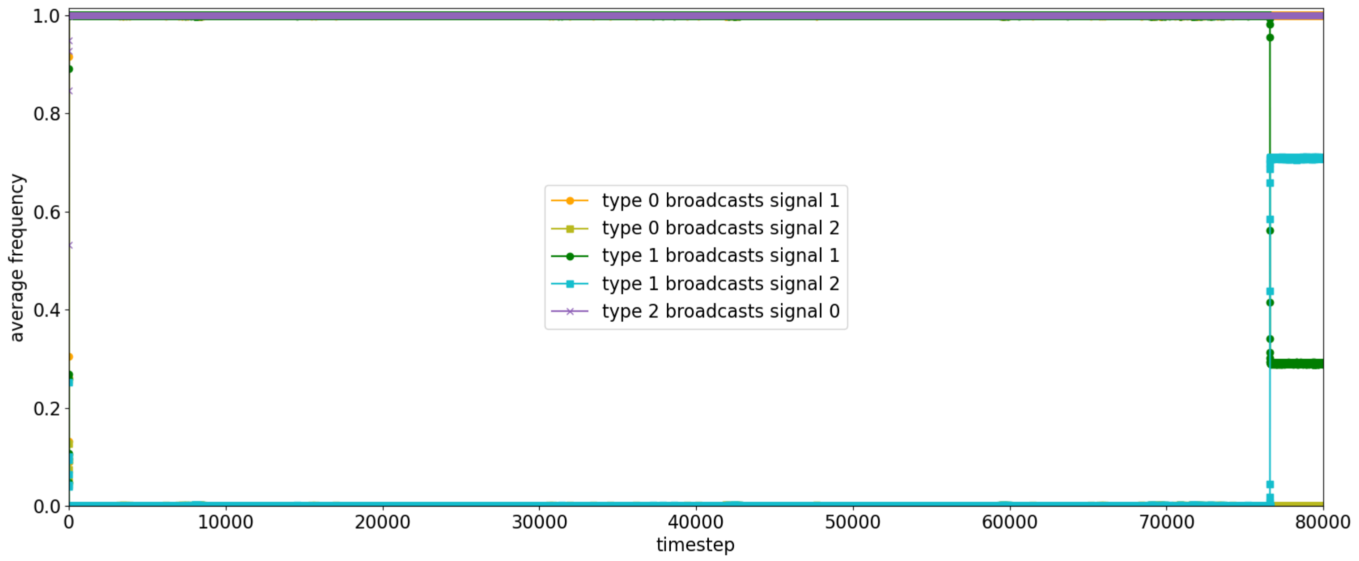

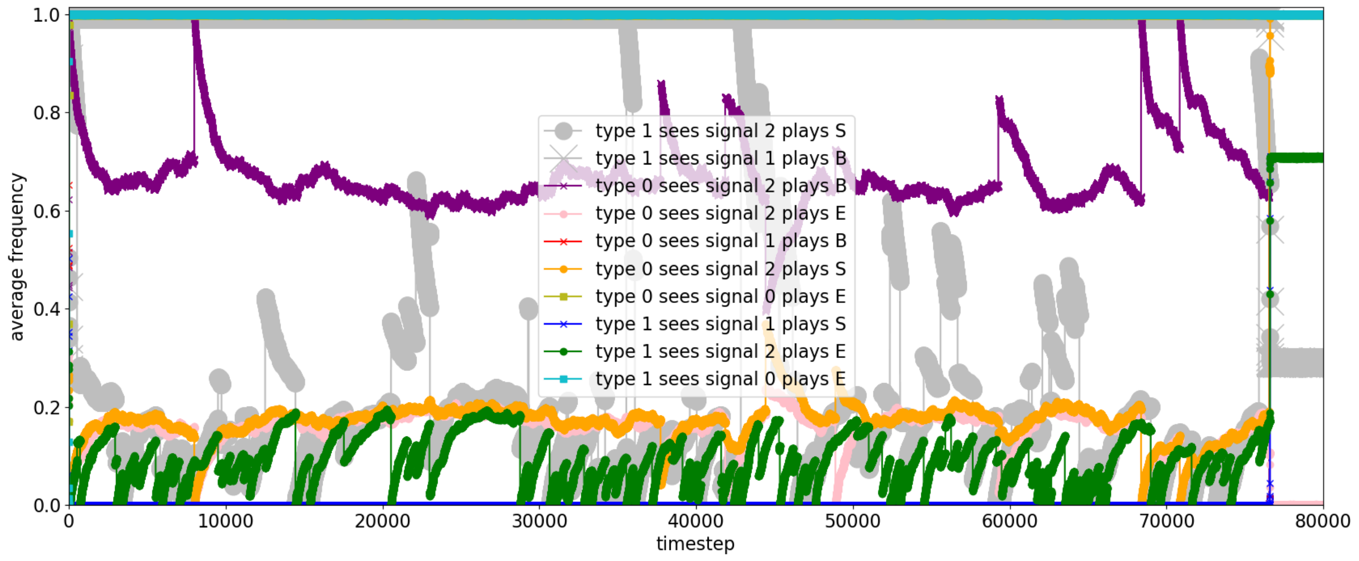

Continued

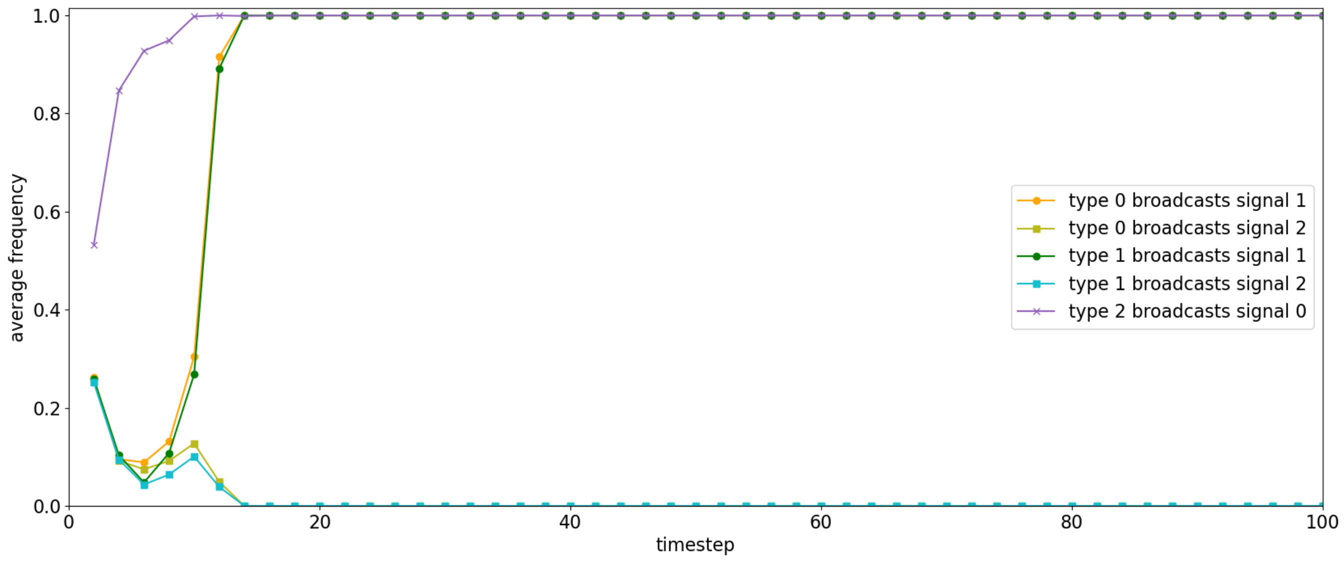
average frequency
timestep
type 0 broadcasts signal 1
type 0 broadcasts signal 2
type 1 broadcasts signal 1
type 1 broadcasts signal 2
type 2 broadcasts signal 0

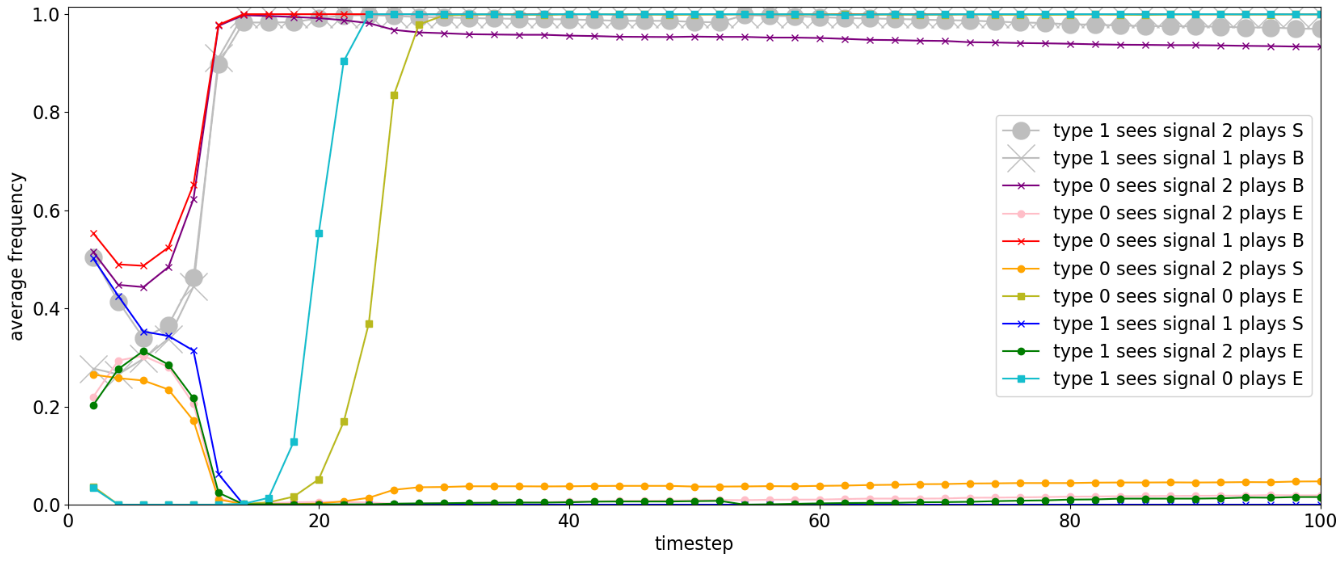
average frequency
timestep
type 1 sees signal 2 plays S
type 1 sees signal 1 plays B
type 0 sees signal 2 plays B
type 0 sees signal 2 plays E
type 0 sees signal 1 plays B
type 0 sees signal 2 plays S
type 0 sees signal 0 plays E
type 1 sees signal 1 plays S
type 1 sees signal 2 plays E
type 1 sees signal 0 plays E

Continued

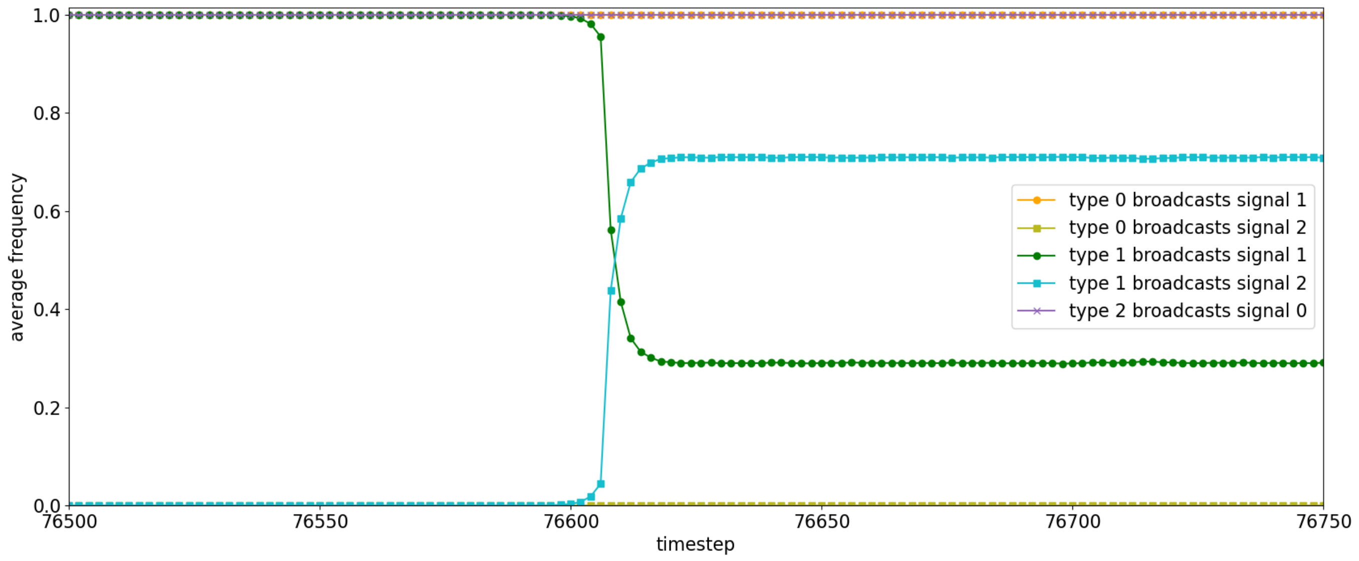
average frequency
1.0
0.8
0.6
0.4
0.2
0.0
76500
76550
76600
76650
76700
76750
timestep
type 0 broadcasts signal 1
type 0 broadcasts signal 2
type 1 broadcasts signal 1
type 1 broadcasts signal 2
type 2 broadcasts signal 0

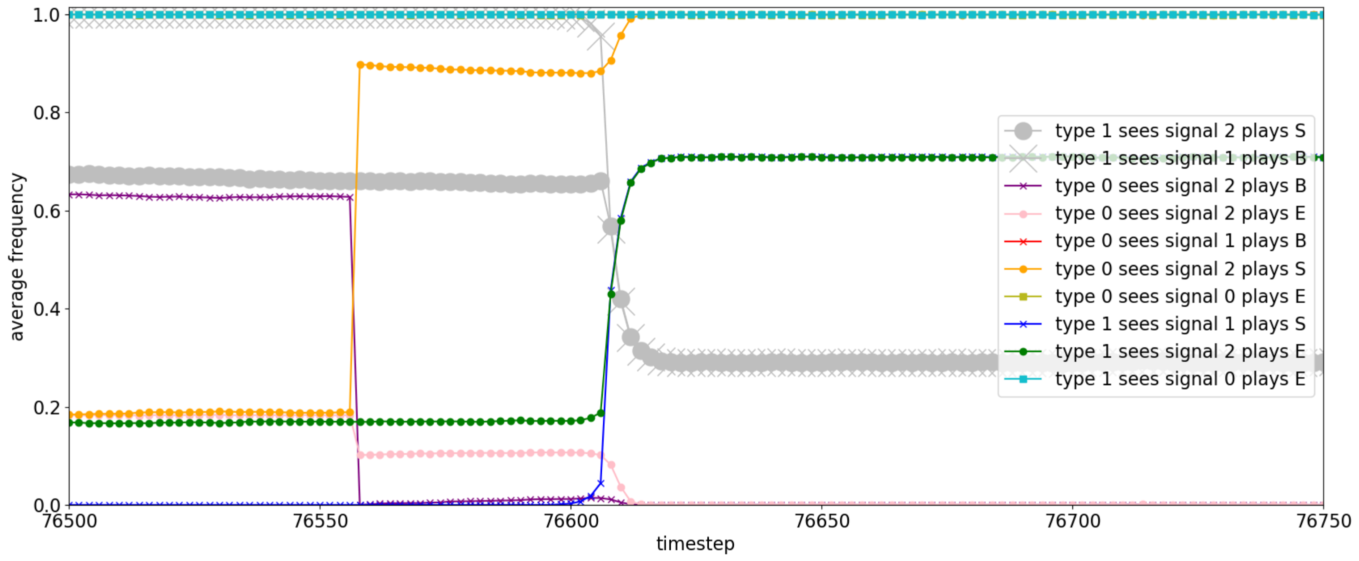
average frequency
1.0
0.8
0.6
0.4
0.2
0.0
76500
76550
76600
76650
76700
76750
timestep
type 1 sees signal 2 plays S
type 1 sees signal 1 plays B
type 0 sees signal 2 plays B
type 0 sees signal 2 plays E
type 0 sees signal 1 plays B
type 0 sees signal 2 plays S
type 0 sees signal 0 plays E
type 1 sees signal 1 plays S
type 1 sees signal 2 plays E
type 1 sees signal 0 plays E

Continued

	broadcast signal	play when sees 0	play when sees 2	play when sees 1	number of agents with this profile
minority profile	1	E	S	B	898
dominant profile	2	E	E	S	2201
hypothetical profile					0
	B	S	E		
type 1 coordination preferences:	1	1.5	0.5		
total number of agents in population who		signal	play on signal 0	play on signal 2	play on signal 1
type 0	3501	1	E	S	B
type 1	3099		E		
type 2	3400	0	E	E	E
EU for minority	9400.5				
EU for dominant	9399				

alpha = 0.5 and beta = 0.04 run # 254

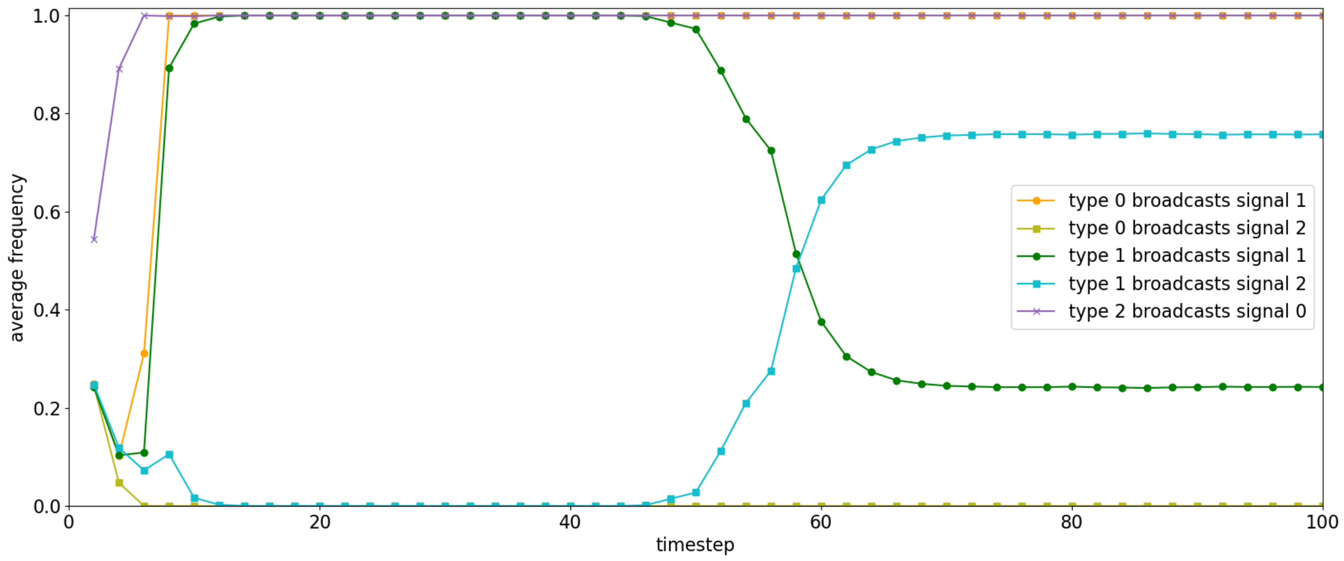

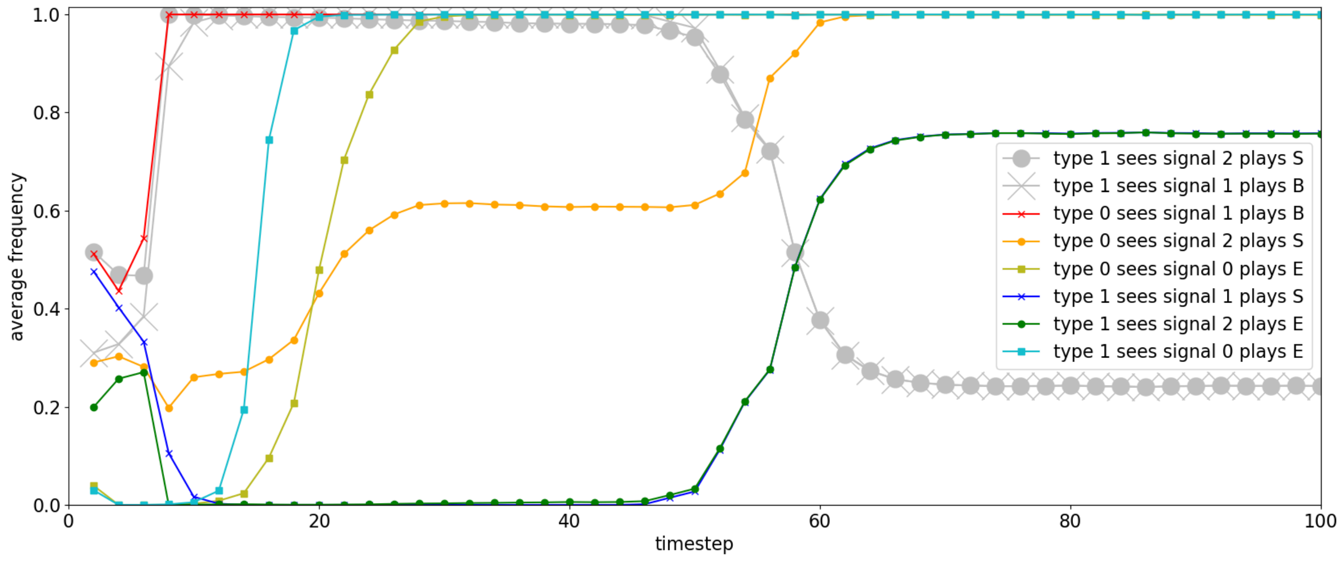

Continued

	broadcast signal	play when sees 0	play when sees 2	play when sees 1	number of agents with this profile
minority profile	1	E	S	B	700
dominant profile	2	E	E	S	2200
hypothetical profile					0
	B	S	E		
type 1 coordination preferences:	1	1.5	0.5		
total number of agents in population who		signal	play on signal 0	play on signal 2	play on signal 1
type 0	3700	1	E	S	B
type 1	2900		E		
type 2	3400	0	E	E	E
EU for minority	9400				
EU for dominant	9400				

See “a5b1_run794”

outcomes (xvii):

(note: don't forget that agents who signal 0 treat all received signals as 0)

alpha = 0.3 and beta = 0.06, Run # 59

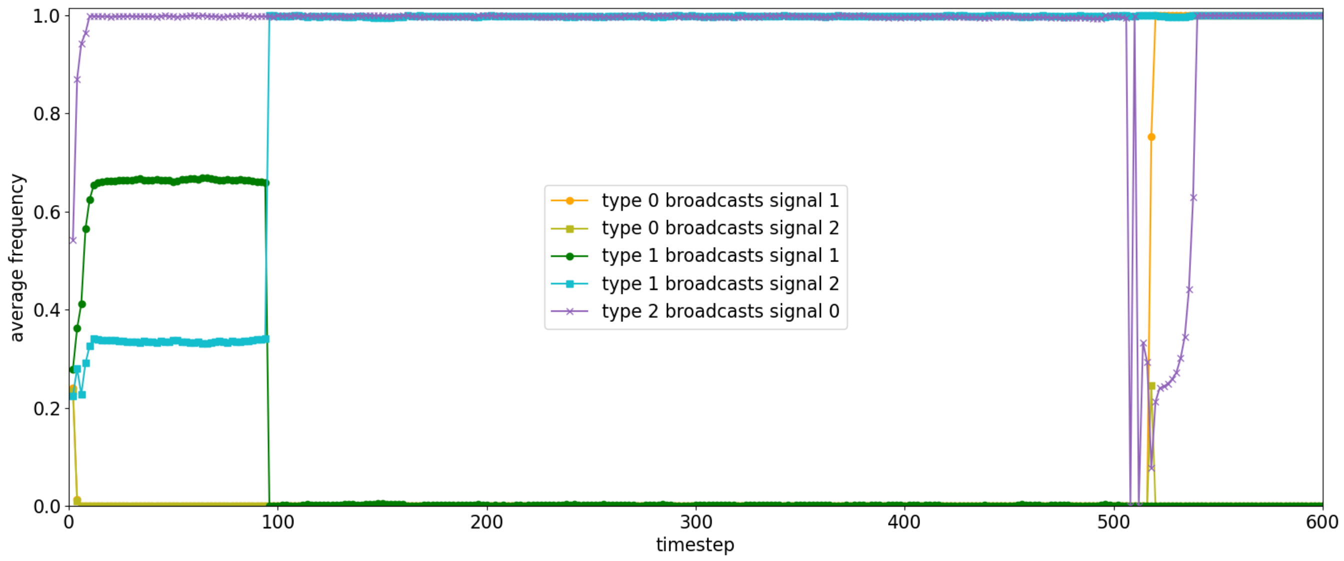

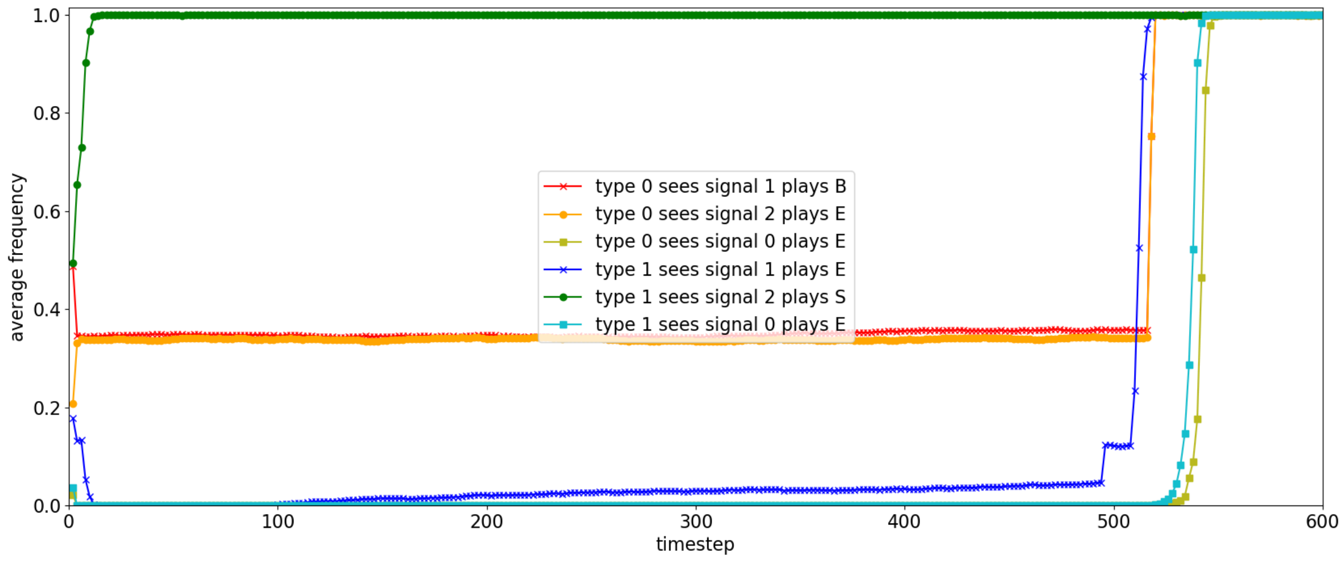

Continued

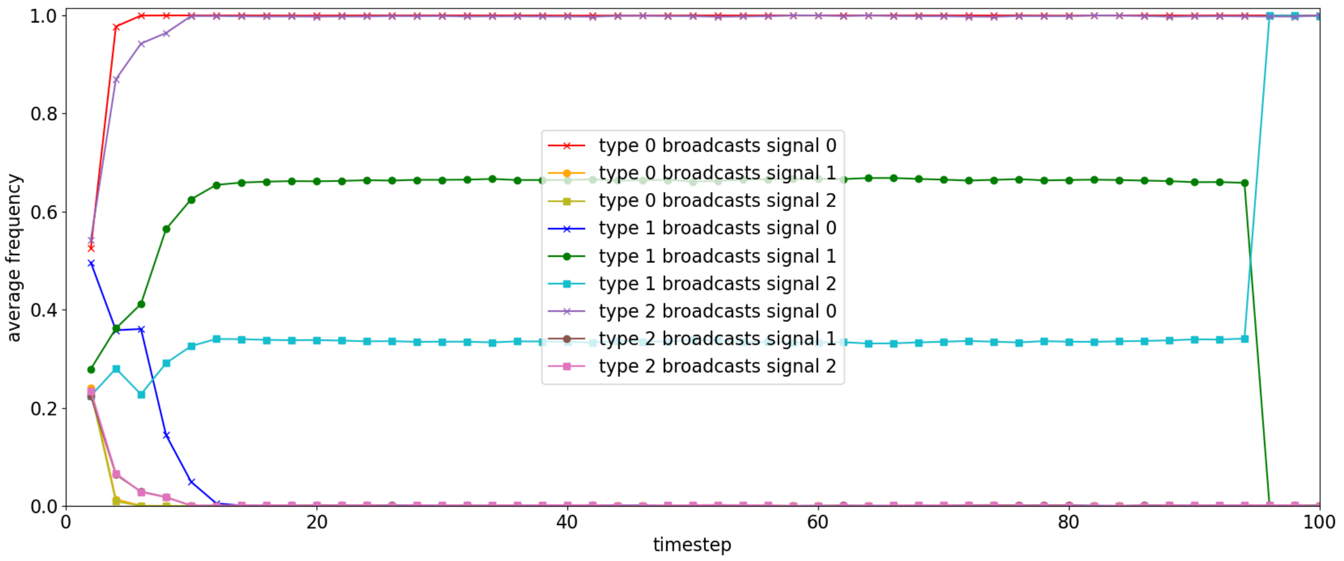
average frequency
timestep
type 0 broadcasts signal 0
type 0 broadcasts signal 1
type 0 broadcasts signal 2
type 1 broadcasts signal 0
type 1 broadcasts signal 1
type 1 broadcasts signal 2
type 2 broadcasts signal 0
type 2 broadcasts signal 1
type 2 broadcasts signal 2

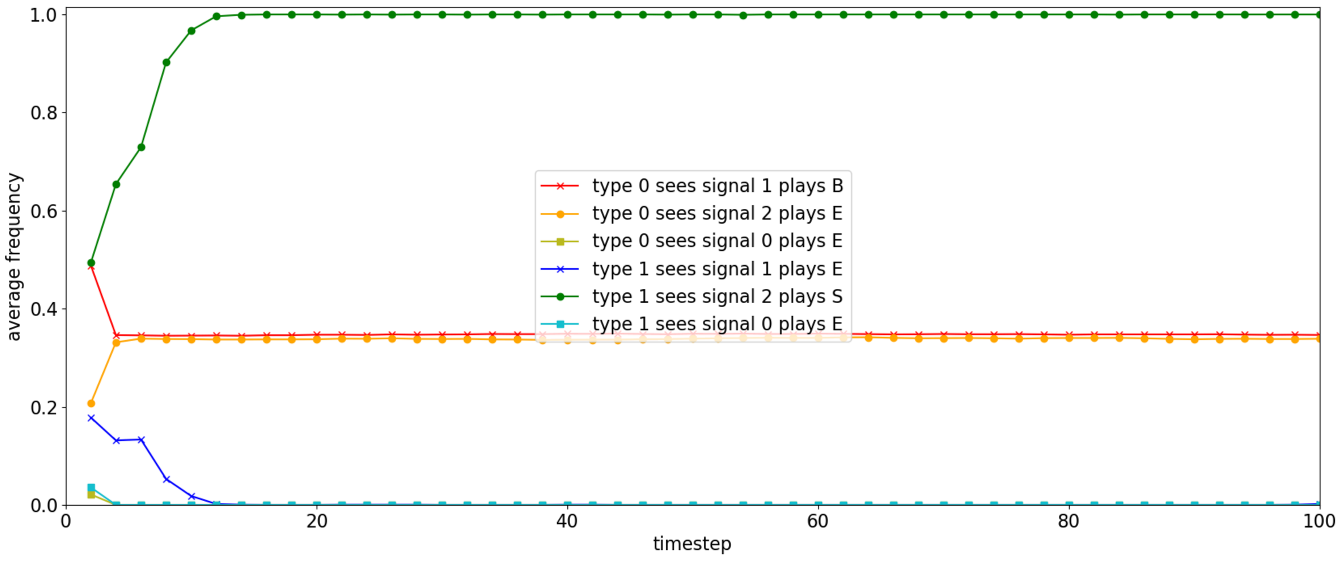
average frequency
timestep
type 0 sees signal 1 plays B
type 0 sees signal 2 plays E
type 0 sees signal 0 plays E
type 1 sees signal 1 plays E
type 1 sees signal 2 plays S
type 1 sees signal 0 plays E

Continued

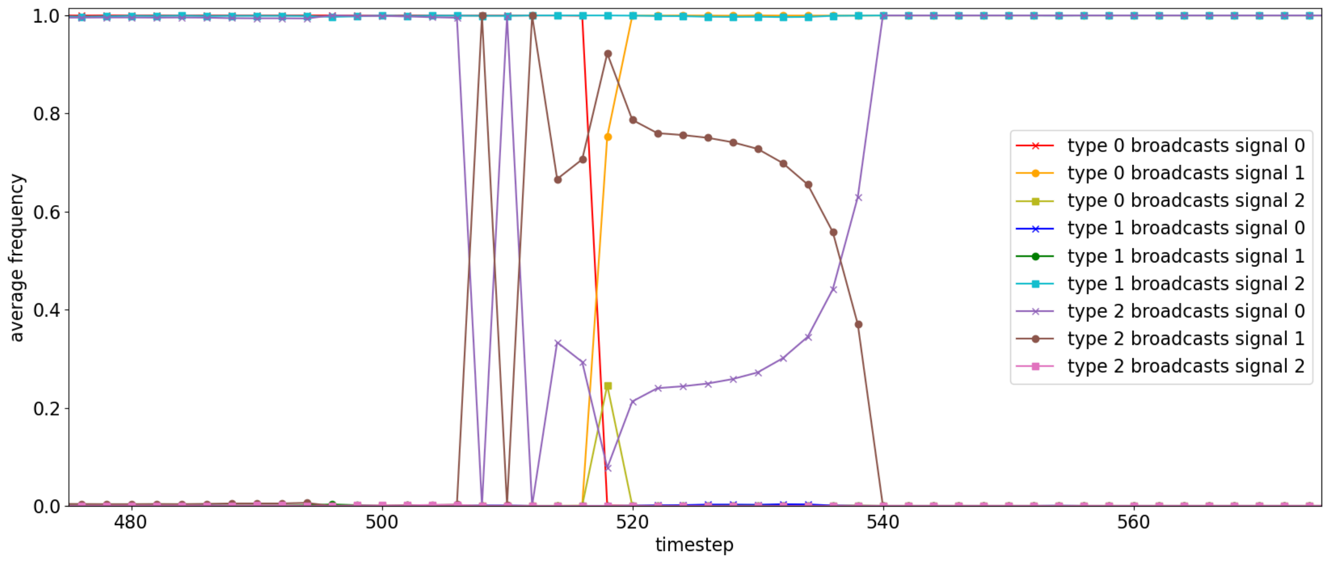
average frequency
timestep
type 0 broadcasts signal 0
type 0 broadcasts signal 1
type 0 broadcasts signal 2
type 1 broadcasts signal 0
type 1 broadcasts signal 1
type 1 broadcasts signal 2
type 2 broadcasts signal 0
type 2 broadcasts signal 1
type 2 broadcasts signal 2

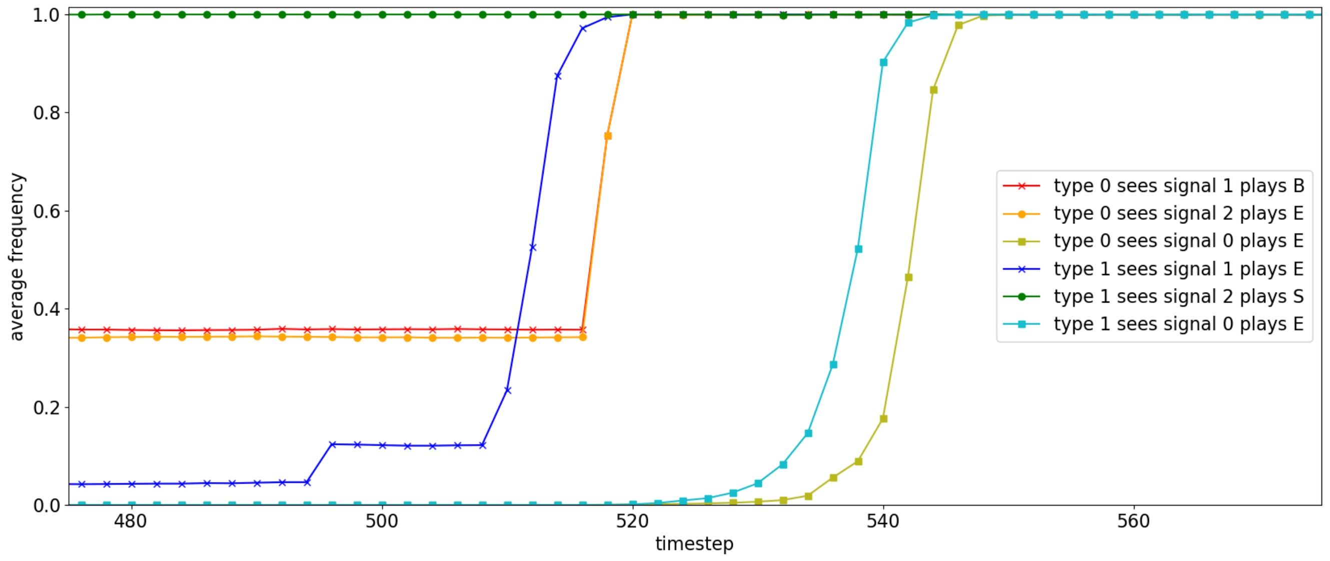
average frequency
timestep
type 0 sees signal 1 plays B
type 0 sees signal 2 plays E
type 0 sees signal 0 plays E
type 1 sees signal 1 plays E
type 1 sees signal 2 plays S
type 1 sees signal 0 plays E

alpha = 0.4 and beta = 0.08 run # 264

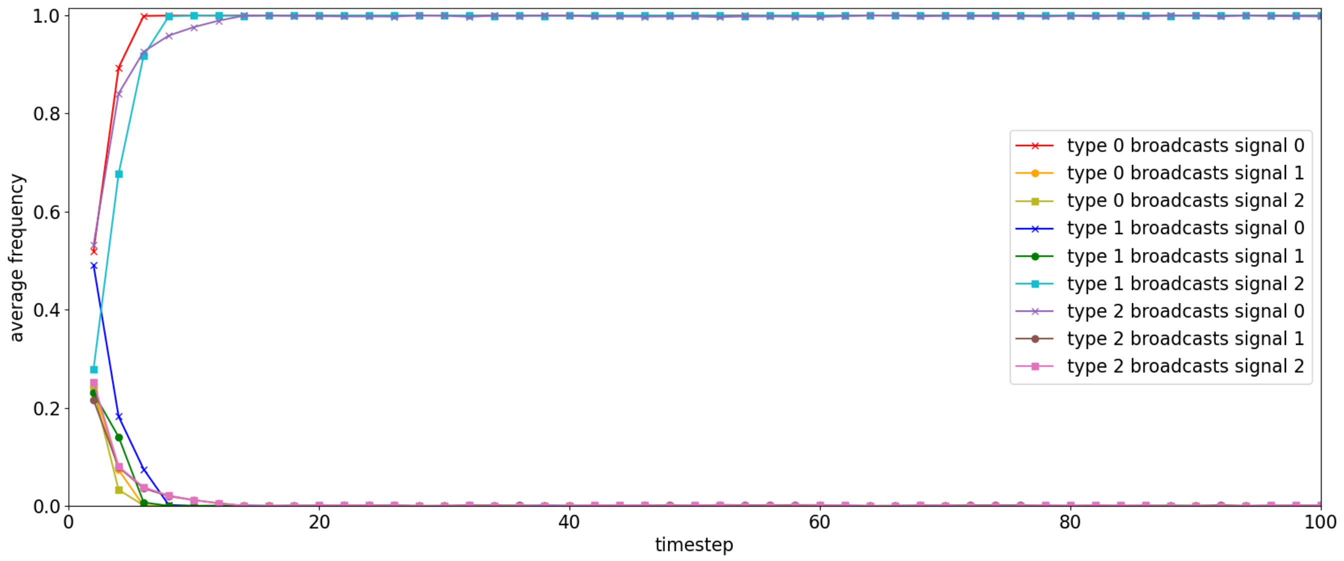

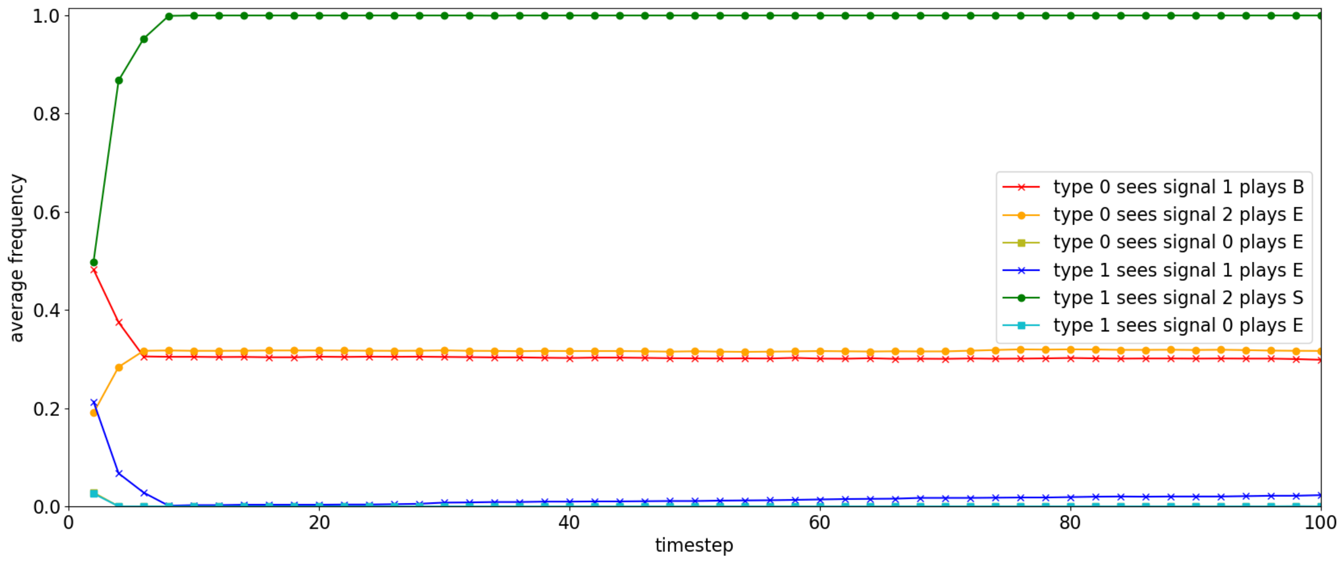

Continued

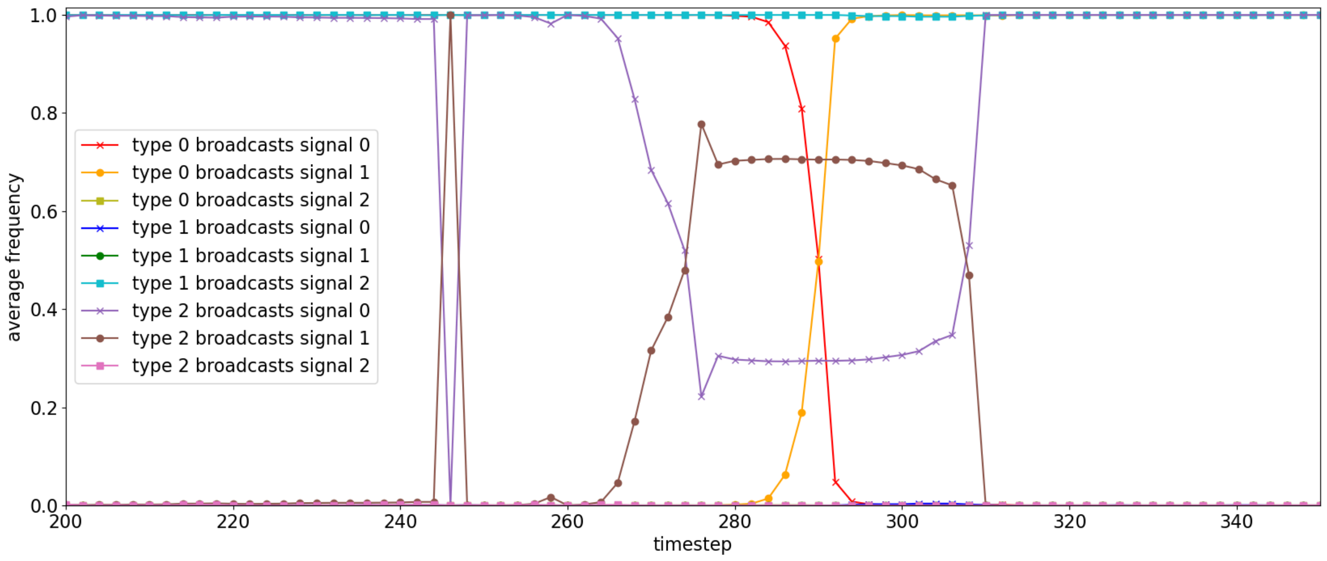
average frequency
timestep
type 0 broadcasts signal 0
type 0 broadcasts signal 1
type 0 broadcasts signal 2
type 1 broadcasts signal 0
type 1 broadcasts signal 1
type 1 broadcasts signal 2
type 2 broadcasts signal 0
type 2 broadcasts signal 1
type 2 broadcasts signal 2
0.0
0.2
0.4
0.6
0.8
1.0
200
220
240
260
280
300
320
340

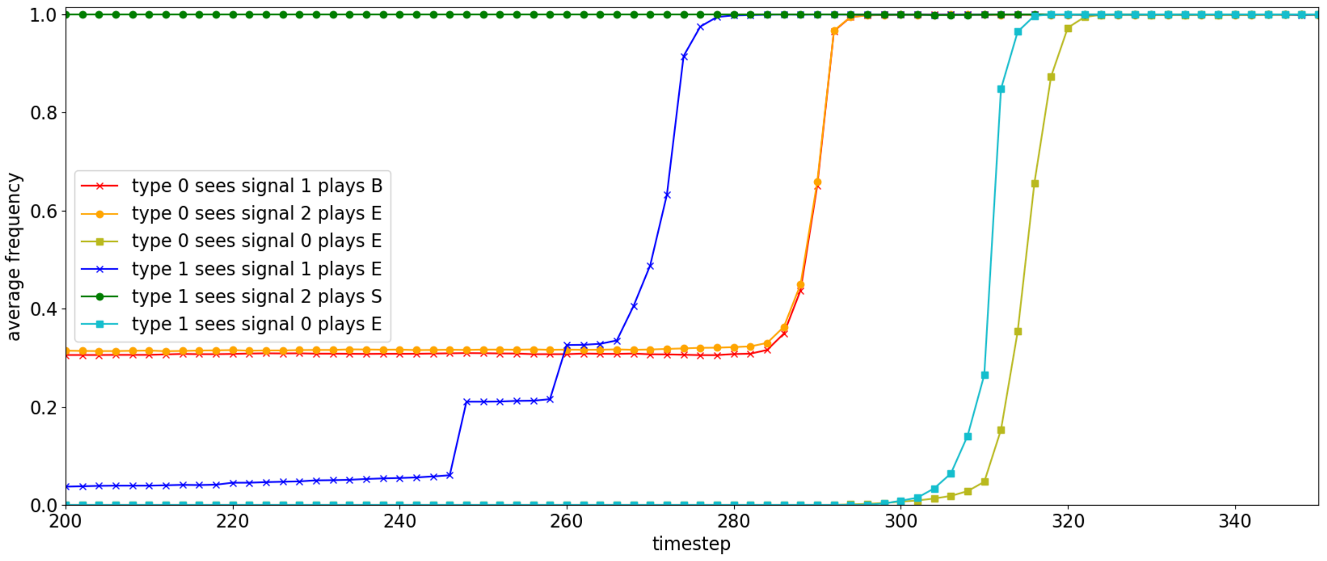
average frequency
timestep
type 0 sees signal 1 plays B
type 0 sees signal 2 plays E
type 0 sees signal 0 plays E
type 1 sees signal 1 plays E
type 1 sees signal 2 plays S
type 1 sees signal 0 plays E
0.0
0.2
0.4
0.6
0.8
1.0
200
220
240
260
280
300
320
340

alpha = 0.5 and beta = 0.06 run # 876

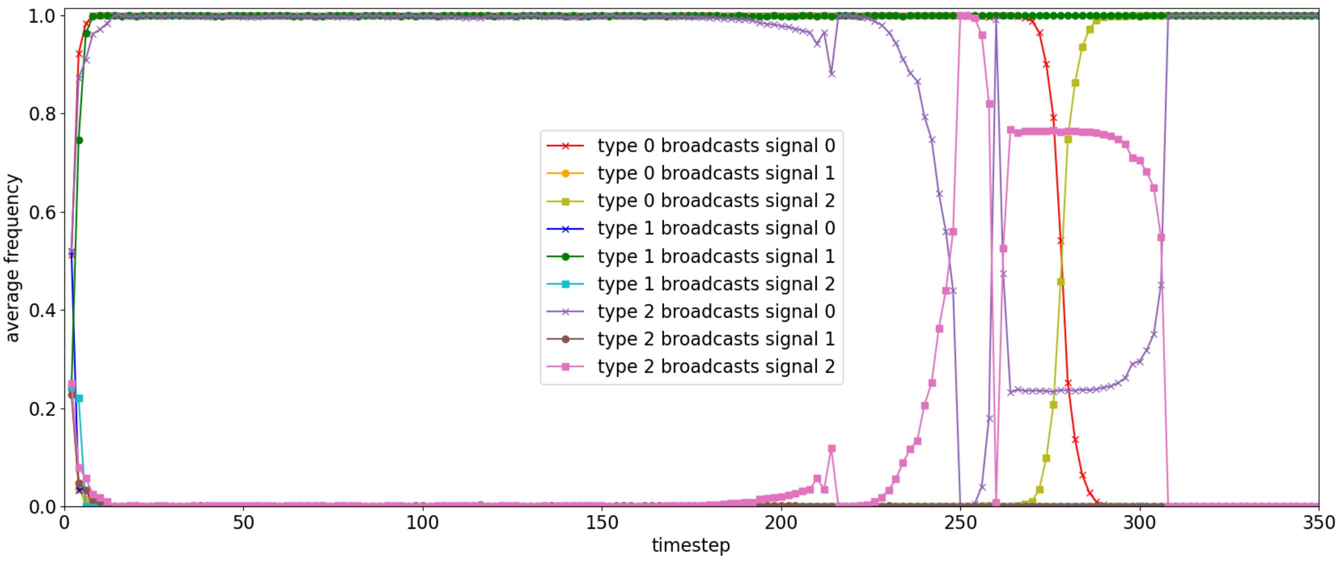

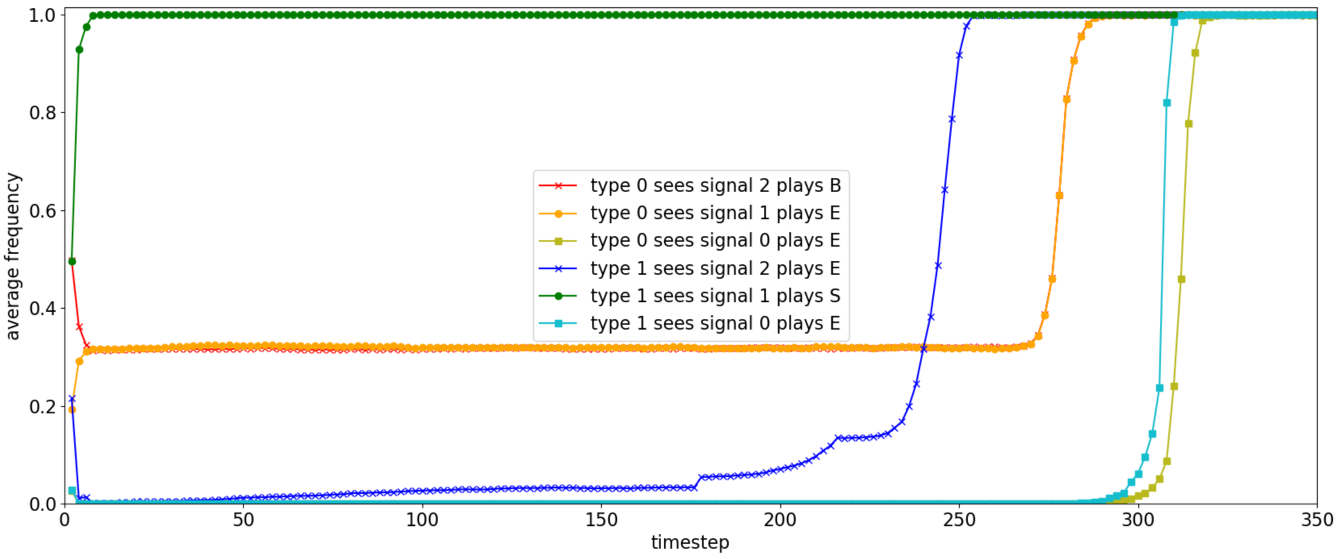

alpha = 0.5 and beta = 0.1 run # 196

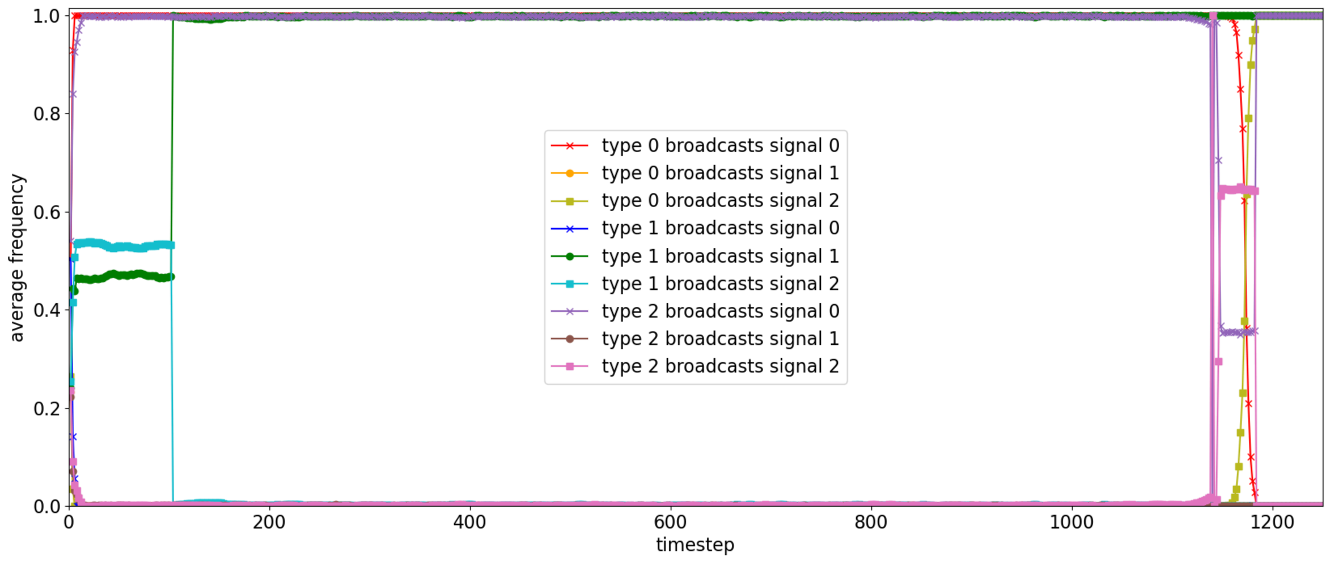

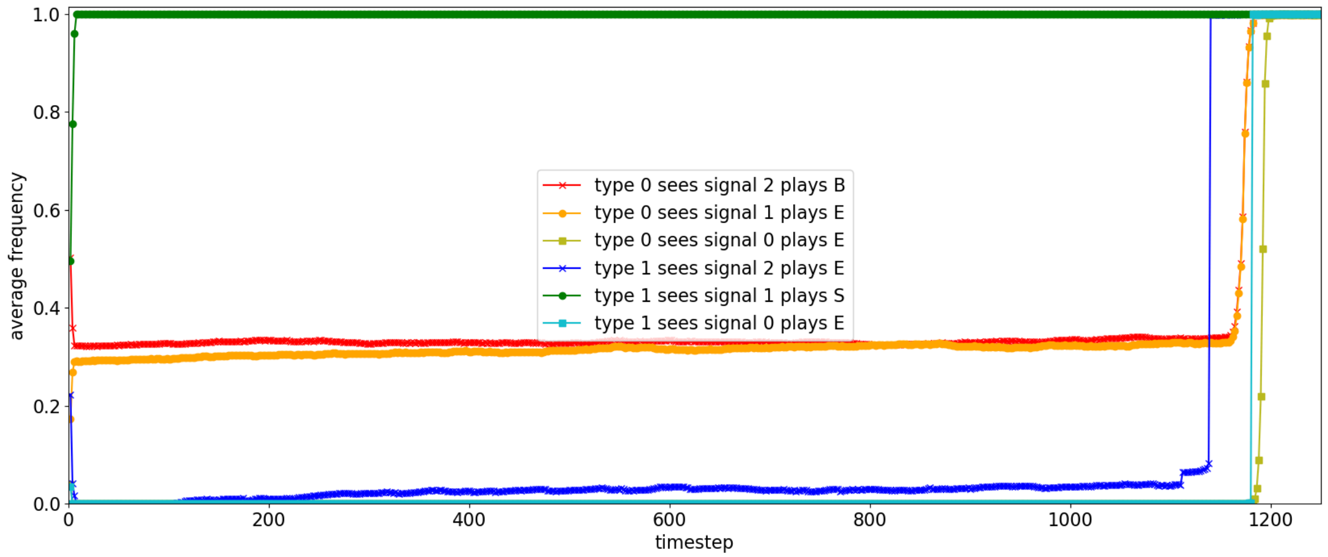

Continued

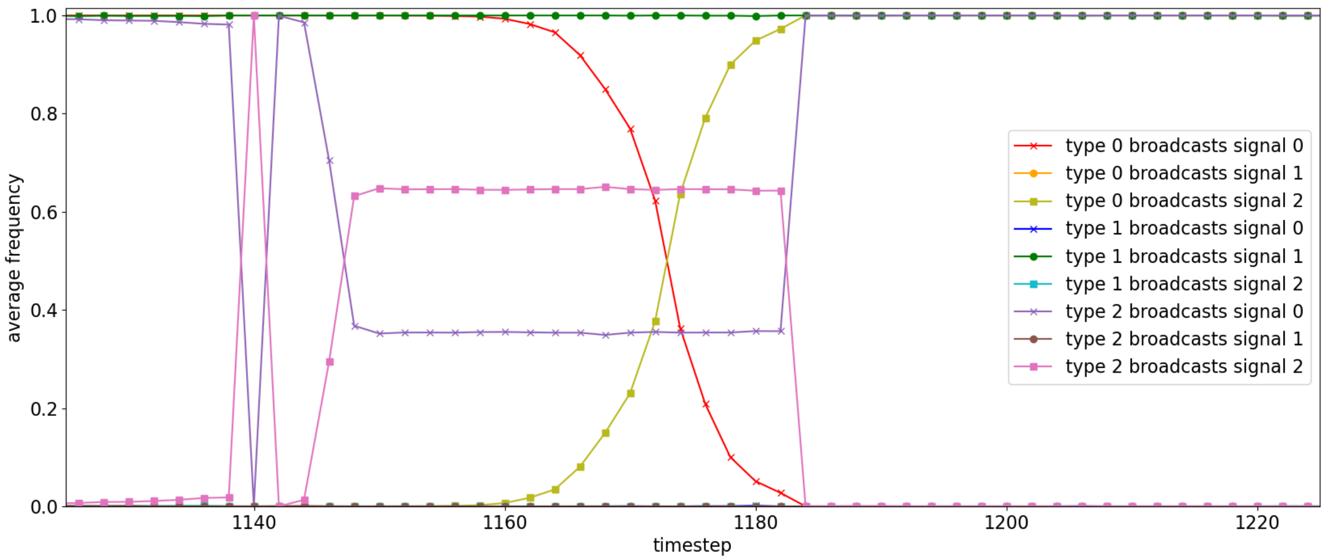

average frequency
timestep
type 0 broadcasts signal 0
type 0 broadcasts signal 1
type 0 broadcasts signal 2
type 1 broadcasts signal 0
type 1 broadcasts signal 1
type 1 broadcasts signal 2
type 2 broadcasts signal 0
type 2 broadcasts signal 1
type 2 broadcasts signal 2

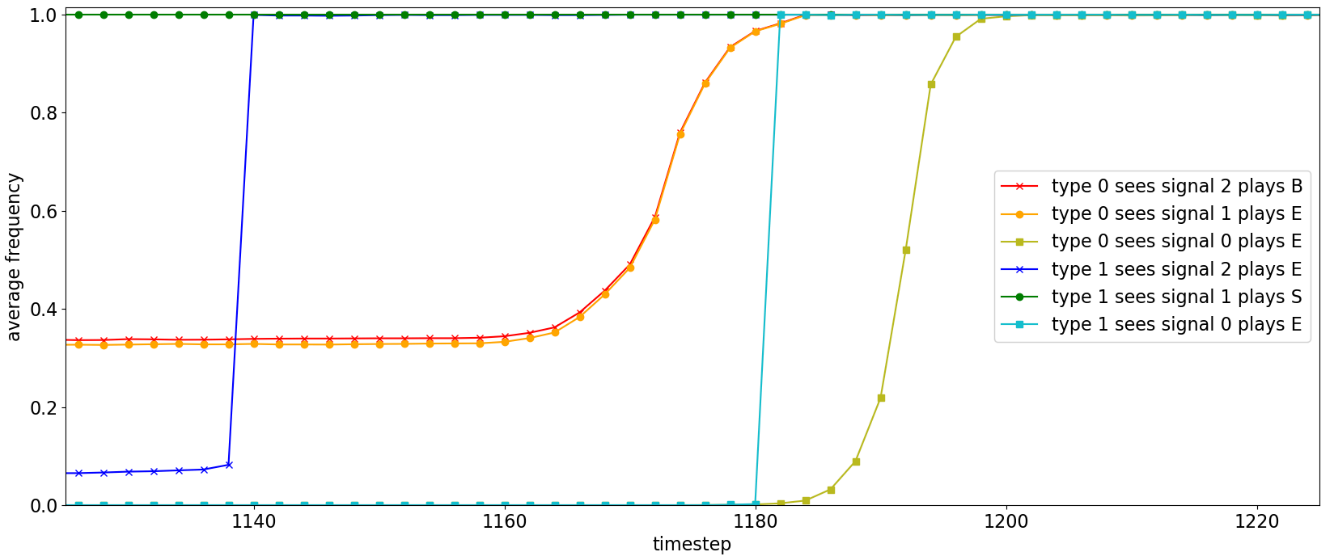

average frequency
timestep
type 0 sees signal 2 plays B
type 0 sees signal 1 plays E
type 0 sees signal 0 plays E
type 1 sees signal 2 plays E
type 1 sees signal 1 plays S
type 1 sees signal 0 plays E

alpha = 0.5 and beta = 0.1 run # 222

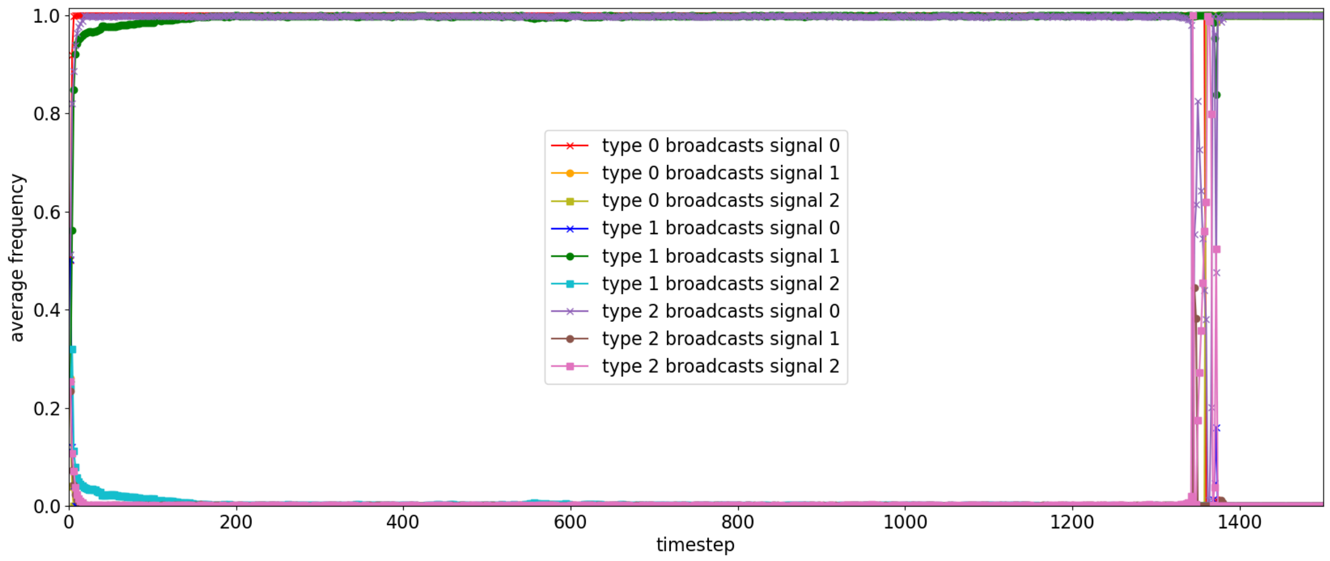

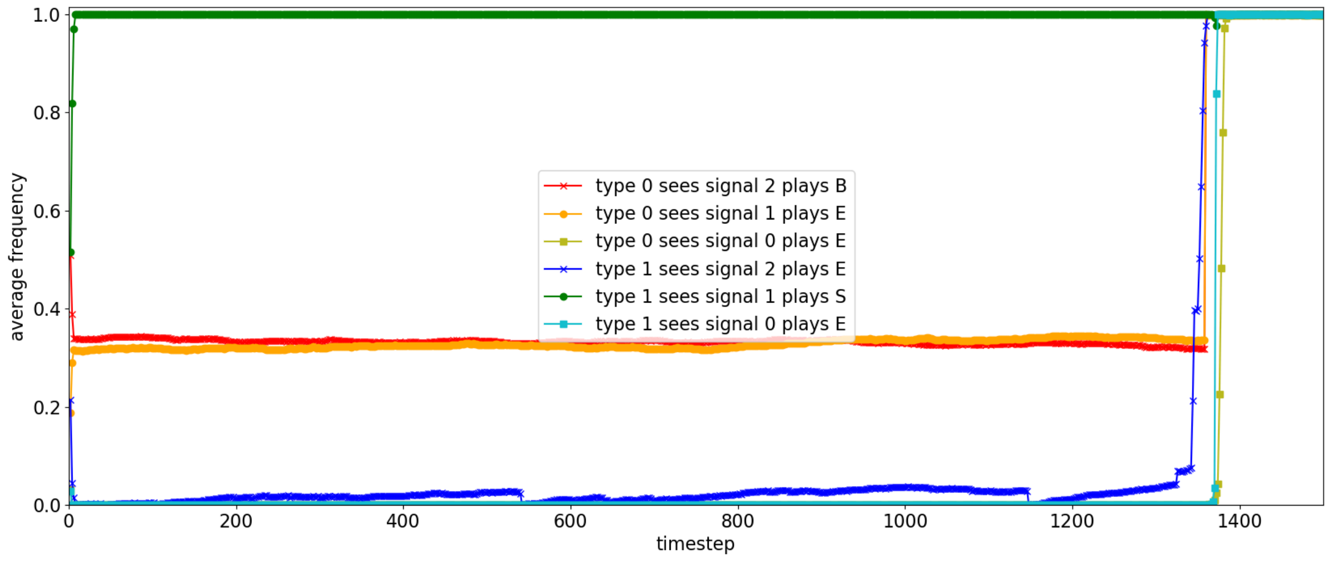

Continued

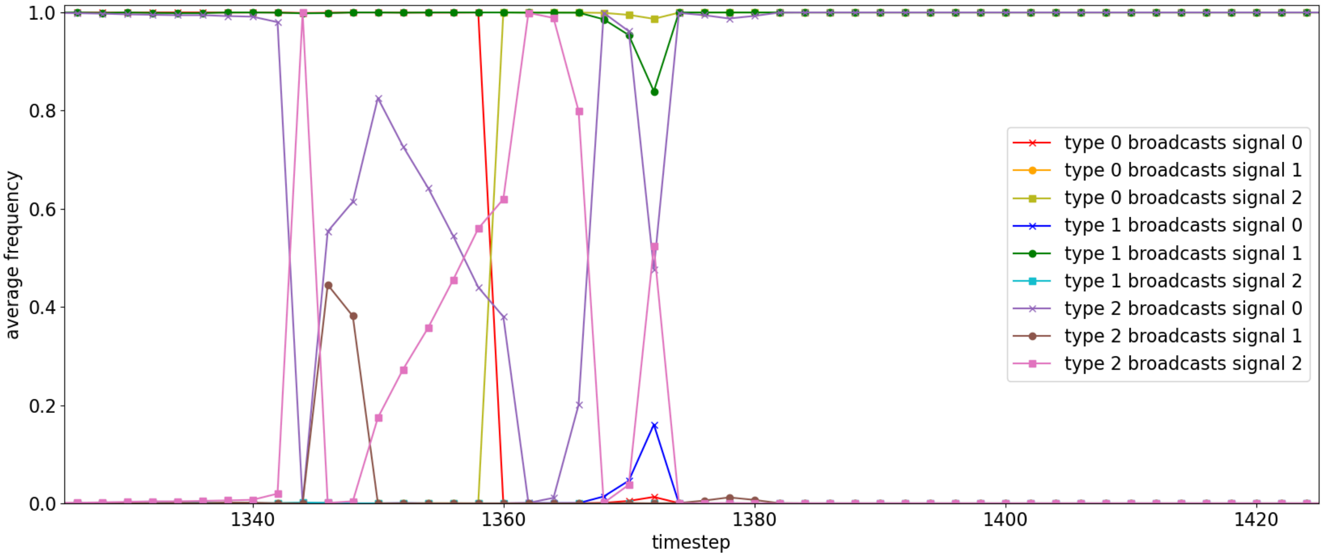

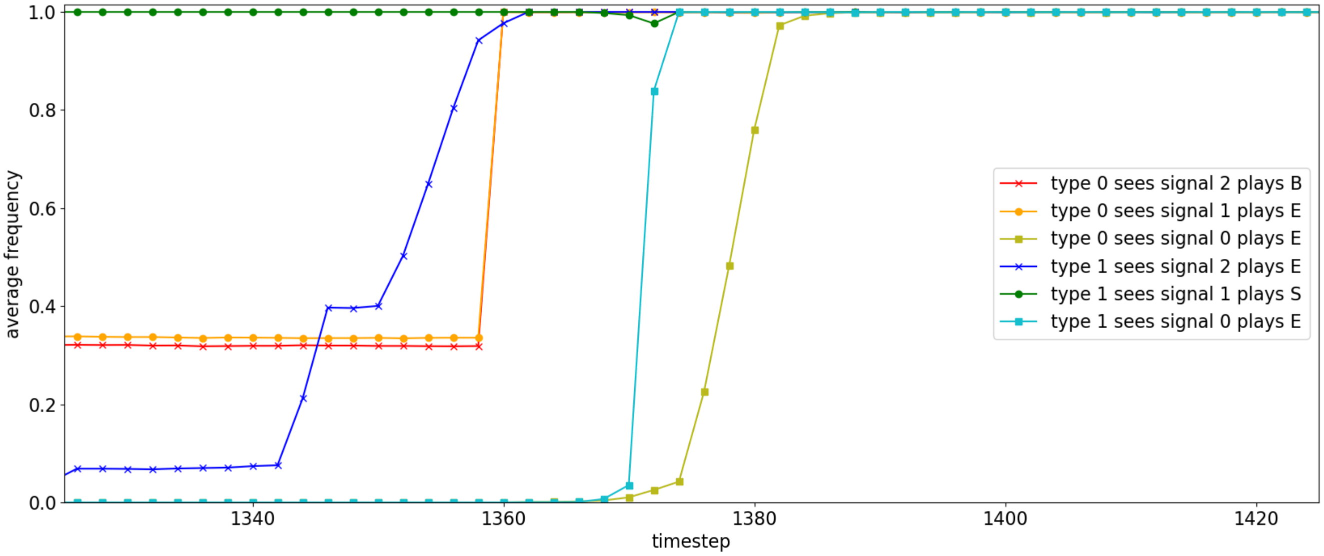

Finally done!
